# Parameterization of the miniPEG-Modified γPNA Backbone: Toward Induced γPNA Duplex Dissociation

**DOI:** 10.1021/acs.jctc.2c01163

**Published:** 2023-05-17

**Authors:** Angel Tamez, Lennart Nilsson, Mihaela-Rita Mihailescu, Jeffrey D. Evanseck

**Affiliations:** † Center for Computational Sciences and the Department of Chemistry and Biochemistry, Duquesne University, Pittsburgh, Pennsylvania 15282, United States; ‡ Department of Biosciences and Nutrition, Karolinska Institute, Solnavägen 1, 171 77 Solna, Sweden

## Abstract

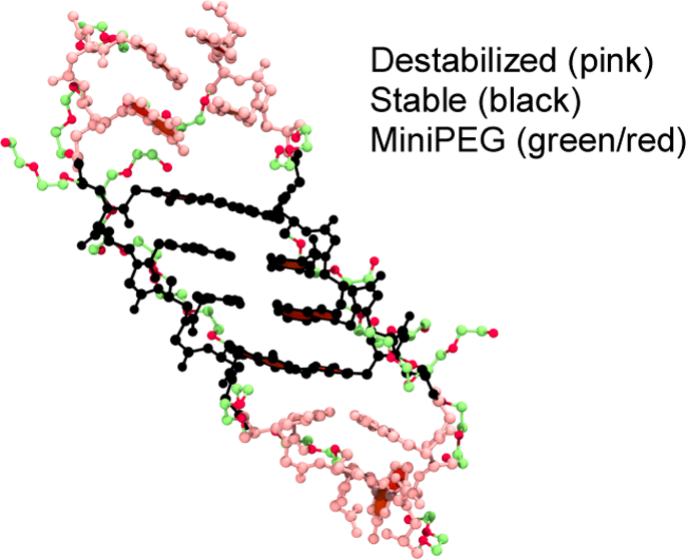

γ-Modified peptide nucleic acids (γPNAs) serve as potential therapeutic agents against genetic diseases. Miniature poly(ethylene glycol) (miniPEG) has been reported to increase solubility and binding affinity toward genetic targets, yet details of γPNA structure and dynamics are not understood. Within our work, we parameterized missing torsional and electrostatic terms for the miniPEG substituent on the γ-carbon atom of the γPNA backbone in the CHARMM force field. Microsecond timescale molecular dynamics simulations were carried out on six miniPEG-modified γPNA duplexes from NMR structures (PDB ID: 2KVJ). Three NMR models for the γPNA duplex (PDB ID: 2KVJ) were simulated as a reference for structural and dynamic changes captured for the miniPEG-modified γPNA duplex. Principal component analysis performed on the γPNA backbone atoms identified a single isotropic conformational substate (CS) for the NMR simulations, whereas four anisotropic CSs were identified for the ensemble of miniPEG-modified γPNA simulations. The NMR structures were found to have a 23° helical bend toward the major groove, consistent with our simulated CS structure of 19.0°. However, a significant difference between simulated methyl- and miniPEG-modified γPNAs involved the opportunistic invasion of miniPEG through the minor and major groves. Specifically, hydrogen bond fractional analysis showed that the invasion was particularly prone to affect the second G–C base pair, reducing the Watson–Crick base pair hydrogen bond by 60% over the six simulations, whereas the A–T base pairs decreased by only 20%. Ultimately, the invasion led to base stack reshuffling, where the well-ordered base stacking was reduced to segmented nucleobase stacking interactions. Our 6 μs timescale simulations indicate that duplex dissociation suggests the onset toward γPNA single strands, consistent with the experimental observation of decreased aggregation. To complement the insight of miniPEG-modified γPNA structure and dynamics, the new miniPEG force field parameters allow for further exploration of such modified γPNA single strands as potential therapeutic agents against genetic diseases.

## Introduction

**Figure 1:**
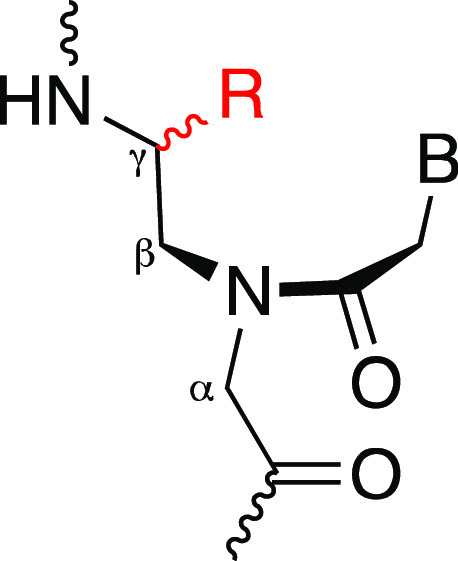
Chemical structure of a γPNA monomer. The Greek lettering identifies the carbon atom position labeling with R designating modifications at the γ-position and B designating nucleobases attached to the γPNA monomer.

Peptide nucleic acids (PNAs) were introduced in 1991 as nucleic acid analogues composed of a 2-aminoethylglycine backbone that replaces the sugar phosphate backbone and a base covalently bonded through a carboxymethyl linker (Figure 1).^1−4^

**Figure 2:**
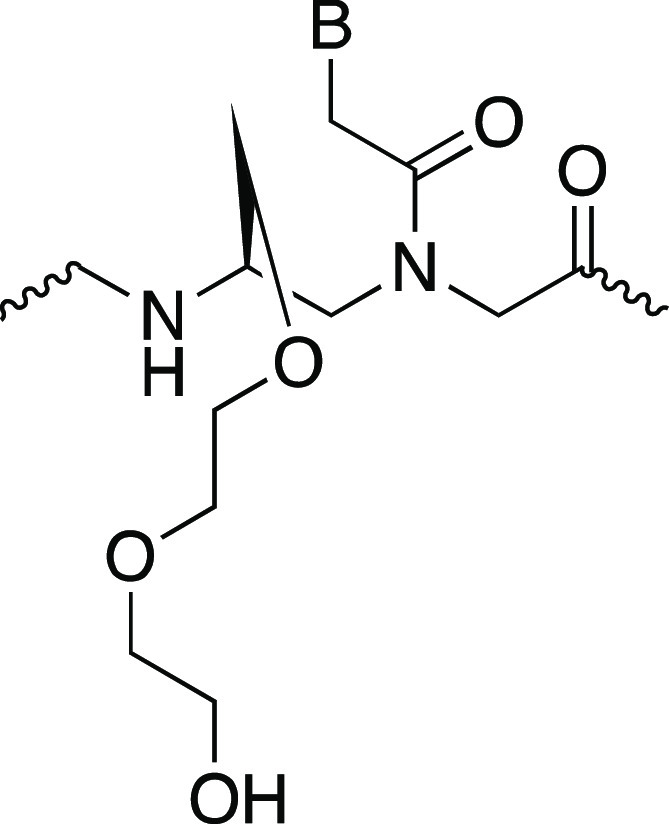
Chemical structure of an (*R*-)miniPEG-modified γPNA monomer with B representing the nucleobase.

PNAs have been reported to be stronger in binding to a complementary DNA or RNA strand due to a lack of electron repulsion from the neutral backbone.^5−7^ Moreover, PNAs are discriminatory in binding, allowing for high sequence specificity and affinity to a genetic target, and inherently capable of evading degradation by nucleases or proteases.^8,9^ Unfortunately, the neutrality of the backbone of PNAs limits their incorporation into therapeutic design, as it has been reported that single-stranded PNAs suffer from aggregation in solution.^10,11^ A way to prevent aggregation was to introduce chirality into the PNA backbone at positions α, β, or γ (Figure 1). Modifications yielding αPNA^12−16^ and γPNA^7,17,18^ are the most common, greatly reducing its limitations (poor membrane permeability, low aqueous solubility, and ambiguity in RNA binding orientation),^7,9^ while also exploring a new frontier of preferential preorganization.^9^ The αPNA modifications have been assessed, discovering a loss of binding affinity toward the intended genetic target relative to nonmodified PNAs.^19−21^ On the other hand, γPNA modifications deliver improved binding affinity compared to nonmodified PNAs,^7,18,22^ but the structure and dynamics of the promising miniature poly(ethylene glycol) (miniPEG)-modified γPNAs have not been studied. Within our work, we focused on establishing torsional and electrostatic parameters for γPNAs modified with the miniPEG substituent for the CHARMM force field (Figure 2).^11^

MiniPEG has been incorporated into γPNA design, introducing chirality and improvements in aqueous solubility and biocompatibility.^7,9,17,23,24^ The (*R*-)miniPEG modification, referred to as miniPEG herein, at the γ-position preorganizes the γPNA to a canonical right-handed (RH) helix to complement the interaction with DNA or RNA sequences.^17,25,26^ The preorganization into a canonical RH helix reduced the aggregation reported in single-stranded PNAs, and coupled with the inherent increase in binding of PNA molecules, allowed for increasing in affinity toward DNA or RNA targets.^22,23,27,28^ The miniPEG modification has a pharmaceutical interest, yet the structural implications of this modification are not well characterized in the literature.

We used computation to determine the overall structure and dynamics adopted when miniPEG is introduced onto the γPNA backbone. Previously, several molecular dynamics (MD) simulations have been carried out for single-stranded PNA, PNA/PNA, PNA/DNA, and PNA/RNA duplexes, and PNA-containing triplexes.^5,11,29−55^ Most recently, backbone torsional parameters for the nonmodified PNAs were improved for the CHARMM and AMBER force fields.^11^ Specifically, the CHARMM force field was reported to outperform AMBER in base pairing, distinguishing between B-like PNA/DNA duplexes and A-like PNA/RNA duplexes, and reproduction of torsional angles seen in experiment.^11^ The CHARMM force field has yet to be extended for the miniPEG-modified γPNA backbone, posing potential challenges in predicting the capabilities of this PNA group for synthetic design. Previous MD simulations of miniPEG-modified γPNAs replace the modification with a methyl group for ease of modeling,^56,57^ neglecting the structural and dynamic advantages that could be explored.

Within our work, the CHARMM force field was extended to include parameters for the covalent bond between miniPEG and the γPNA backbone. An initial parameterization process was adopted^11,50,51^ but was extended in our work by using the CHARMM General Force Field (CGenFF) protocol.^58−60^ A model compound that defined the backbone between two γPNA residues was modified with a methoxy methyl group, representing the covalent bond between miniPEG and the γPNA backbone for parameter development. The parameters were utilized for structural and dynamic differences against methyl-modified γPNA duplexes (PDB ID: 2KVJ)^10^ with and without the miniPEG-modified γPNA up to the microsecond timescale. The development of these parameters enables the exploration of miniPEG-modified γPNAs against genetic targets.

## Methods

### Force Field Optimization

**Figure 3:**
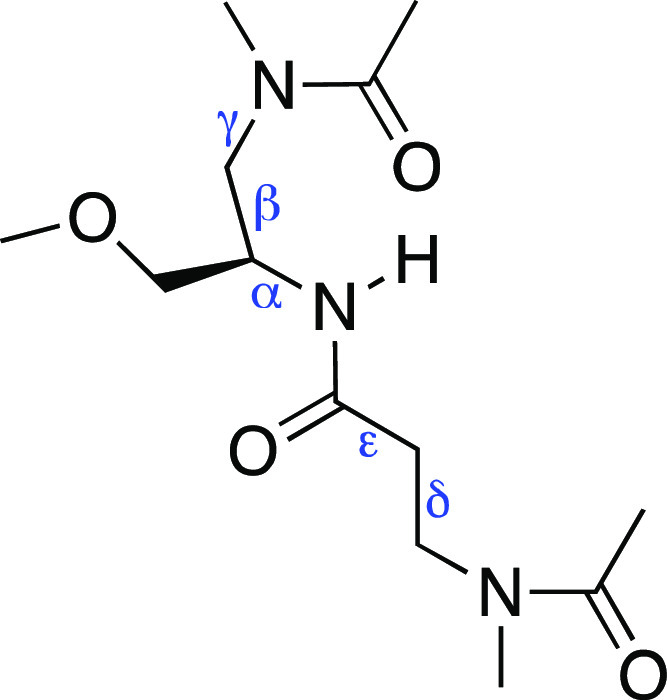
Chemical structure of the model compound used for parameter development. The Greek lettering represents the torsions for the backbone.

Resources at the Center for Computational Sciences at Duquesne University^61^ were used for all parameterization methods and MD simulations. We adapted the PNA backbone model compound reported by Jasiński et al.^11^ and attached a methoxy methyl group to the γ-carbon for parameter development. The model compound represented the backbone between two γPNA residues, a nucleobase was not included within our study and was replaced by methyl groups. The methoxy methyl group replaced a hydrogen atom on the γ-carbon as an (*R-*) stereoisomer (Figure 3). The Set 1 torsional values for the backbone torsions α, β, γ, δ, and ε were used from Jasiński et al.^11^ as −120, 60, 60, 75, and −20°, respectively (Figure 3).

Quantum mechanical (QM) target data was developed employing the CGenFF protocol.^58−60^ The model compound was geometry-optimized at the MP2/6-31+G(d) level of theory using Gaussian 16.^62^ The geometry-optimized structure was parsed through the CGenFF web server (https://cgenff.umaryland.edu/initguess/),^58−60^ deriving initial definitions for the covalent bond defining the miniPEG attachment. Subsequently, a one-dimensional potential energy surface (PES) was established by rotating the dihedral angle for the covalent bond between the γ-carbon and methyl methoxy every 15° from 0 to 360°, constraining the angle at that geometry allowing for all other degrees of freedom to minimize at the same level of theory. The relative energetics generated from the PES became our target for the CHARMM force field.^58−60^ The partial charges for the carbon atoms defining the covalent bond were modified in the CHARMM force field such that the hydrogen atom partial charge of 0.09*e* was aggregated onto each carbon atom.^63^ To maintain neutrality over the miniPEG-modified PNA residues, the partial charge on atom N2′ was reduced by 0.03, becoming −0.36*e* from the Jasiński et al. definition.^11^

The geometries for each QM scan point were extracted and minimized with a harmonic restraint force constant of 10^4^ kcal/mol·radian^2^ on the target torsion.^64^ The torsional terms in eq 1 for the multiplicities (*n*), offsets (δ), and force constants (*k*_χ_) were evaluated against the QM torsional relative energetics.
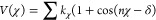
1The CHARMM torsional terms were modified iteratively until convergence with a pairwise error analysis within 0.2 kcal/mol of the PES defined by QM.^63^

### Molecular Dynamics Simulation

We utilized the methyl-modified right-handed γPNA duplex p(^+^NH_3_-CCGTACGG-CO_2_^–^) NMR structure (PDB ID: 2KVJ)^10^ as starting coordinates to assess our parameters. Six randomly chosen NMR models were extracted from the 11 available depositions, mutated to miniPEG-modified duplexes, and simulated, herein referred to as miniPEG Models 1, 2, 4, 8, 9, and 10. Three methyl-modified controls were simulated “as is” from the six randomly chosen structures to baseline the differences found for the miniPEG-modified duplexes, herein referred to as NMR Models 1, 4, and 9. The γPNA models were defined by parameters from the ether force field and CgenFF force field for the diethylene glycol,^58,65,66^ and Jasiński et al. parameters for the PNA backbone and bases.^11^ Each γPNA model was capped with NT (−NH_3_^+^) and CT (−CO_2_^–^) terminal groups and solvated in a cubic box of water defined by the TIP3P water model^67^ with padding of 15 Å in each direction from the atom with the largest coordinate in that direction. The solvated systems were then minimized for 1000 steps, followed by 100 ns of equilibration and 1 μs MD production runs using the nanoscale molecular dynamics (NAMD) engine.^68^ The Langevin piston and temperature control were used to maintain the temperature at 310 K and 1 atm.^69^ All MD simulations were performed within an isobaric/isothermal (*NPT*) ensemble with a 2.0 fs time step. Short-range electrostatics were cut off at 12 Å, with the electrostatics beyond that calculated using the particle mesh Ewald method for electrostatics.^70^ Finally, SHAKE was applied to constrain all bonds connected to hydrogen atoms.^71^

### Simulation Analysis

Visual molecular dynamics (VMD)^72^ was used to visualize the MD simulations and perform classical analyses, root-mean-square deviation (RMSD) and root-mean-square fluctuation (RMSF), on the backbone atoms for the central six base pairs of each γPNA duplex. The terminal base pairs were ignored as they were subject to fraying. We utilized the first frame following the equilibrated structure for the NMR and miniPEG-modified Model 1 simulations as our reference for RMSD. The averaged coordinates for the backbone atoms where the RMSD remained steady in the simulation were used as our reference for the RMSF. Hydrogen bond occupancies were calculated for polar atoms within 3.5 Å at a donor-hydrogen-acceptor angle of 30° from linearity. The python package MDTraj^73^ was used to determine probability distributions for the backbone torsional sampling of the miniPEG-modified simulations. Base stacking interactions were determined using the 3DNA-DSSR software.^74,75^ Base stacking is computed from planar projections of the ring and exocyclic atom in consecutive bases or base pairs, regardless of backbone connectivity.^74^

### Multivariate Statistical Analysis

Principal component analysis (PCA) was carried out on the γPNA backbone for the central six base pairs. The covariance matrix **C** for PCA was defined by atomic coordinates for backbone atoms, whose entries *c*_*ij*_ are given by
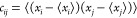
2The dimensionality of the data was reduced, as judged by the scree plot, determining conformational substates (CS) sampled throughout the simulations.^76^ The principal components (PCs) were projected yielding two-dimensional (2D) data points capturing the reduced functional dynamics.^77^ The 2D data points were clustered using the KMeans algorithm encoded in the Scikit-Learn python module,^78^ deriving the CSs and the centroid of each CS as a representative structure. The algorithm places random initial centroids (K) over the 2D data points. Through an iterative process, K clusters are formed by assigning each data point to its closest centroid and a new centroid for each cluster is recomputed.^79^ When Kmeans is applied on MD simulations, “blocky” clusters of similar size are produced.^79,80^ Helical parameters were computed for the 11 NMR models deposited in the PDB database^81^ and stable CS structures using 3DNA-DSSR.^74,75^

### Helical Bending Analysis

Helical bending is frequently reported in the literature, most often used with DNA–protein complexes.^82^ Current programs that measure helical parameters of DNA or RNA, such as 3DNA-DSSR,^74,75^ do not directly compute PNA bending. Thus, the helical bend for our PNA simulations was estimated by deriving the angle between the normal vectors for the geometric centers of atoms N1 and N3 on the second and second-to-last G–C base pairs (G2–C15 and C7–G10) for stable duplexes and simulations.

## Results and Discussion

Parameterization and application of the miniPEG-modified γPNA backbone parameters were performed in a three-step process. First, the CHARMM force field was optimized to match QM torsional relative energetics derived by a small γPNA model, defining the covalent bond between minPEG and γPNA. Within our second step, we performed MD simulations on three right-handed methyl-modified γPNA duplexes (PDB ID: 2KVJ)^10^ to set a baseline for overall structure and dynamics. Finally, six miniPEG-modified γPNA duplexes were simulated to determine the structural and dynamic differences between the methyl-^10^ and miniPEG-modified γPNA duplexes. Our analyses focused on the central six base pairs of the duplexes as the terminal base pairs were subject to fraying.

### Extending the CHARMM Force Field to Include the miniPEG-Modified γPNA Backbone

**Figure 4:**
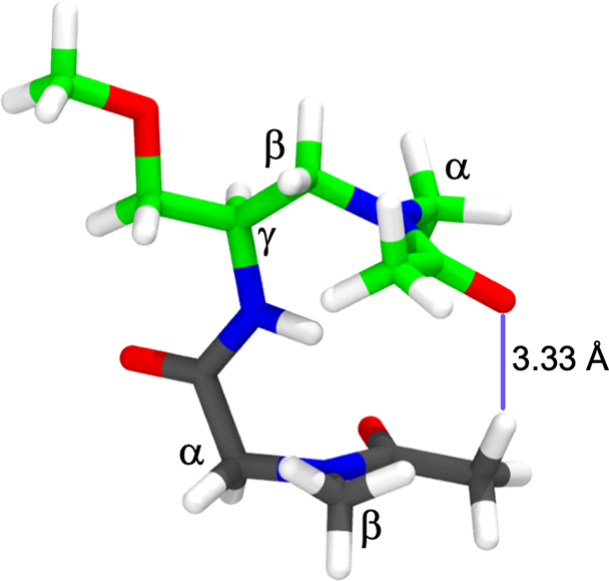
Model compound used for torsional parameterization represented in licorice. The carbon atoms are colored according to residue number; the first residue carbon atoms are colored in green, and the second are colored in gray. The remaining atoms are colored as follows: red for oxygen, blue for nitrogen, and white for hydrogen. The Greek lettering identifies the carbon atom position labeling. The solid blue line indicates the noncovalent interaction between C···O.

The current CHARMM force field^11^ does not have parameters defining the miniPEG-modified γPNA backbone. To develop torsional parameters for the modified backbone, we built a model compound incorporating the entire PNA backbone between two residues, not including the bases, which were replaced with a methyl group adapted from the backbone torsional values defined by Jasiński et al.,^11^ as described in the Methods section. A methoxy methyl group was attached to the γ-carbon, representing the immediate binding atoms used in miniPEG (Figure 4).

The geometry of the model compound was optimized at the MP2/6-31+G(d) level of theory, determining a noncovalent interaction between the carbonyl of one side chain and an α-hydrogen on the methyl group in the subsequent PNA side at a C···O distance of 3.33 Å (Figure 4). The optimized structure was used to rotate the dihedral linkage involving the backbone nitrogen, γ-carbon, methylene carbon, and methoxy methyl oxygen (∠NC^γ^CO) for our QM torsional parameter dataset.

**Figure 5:**
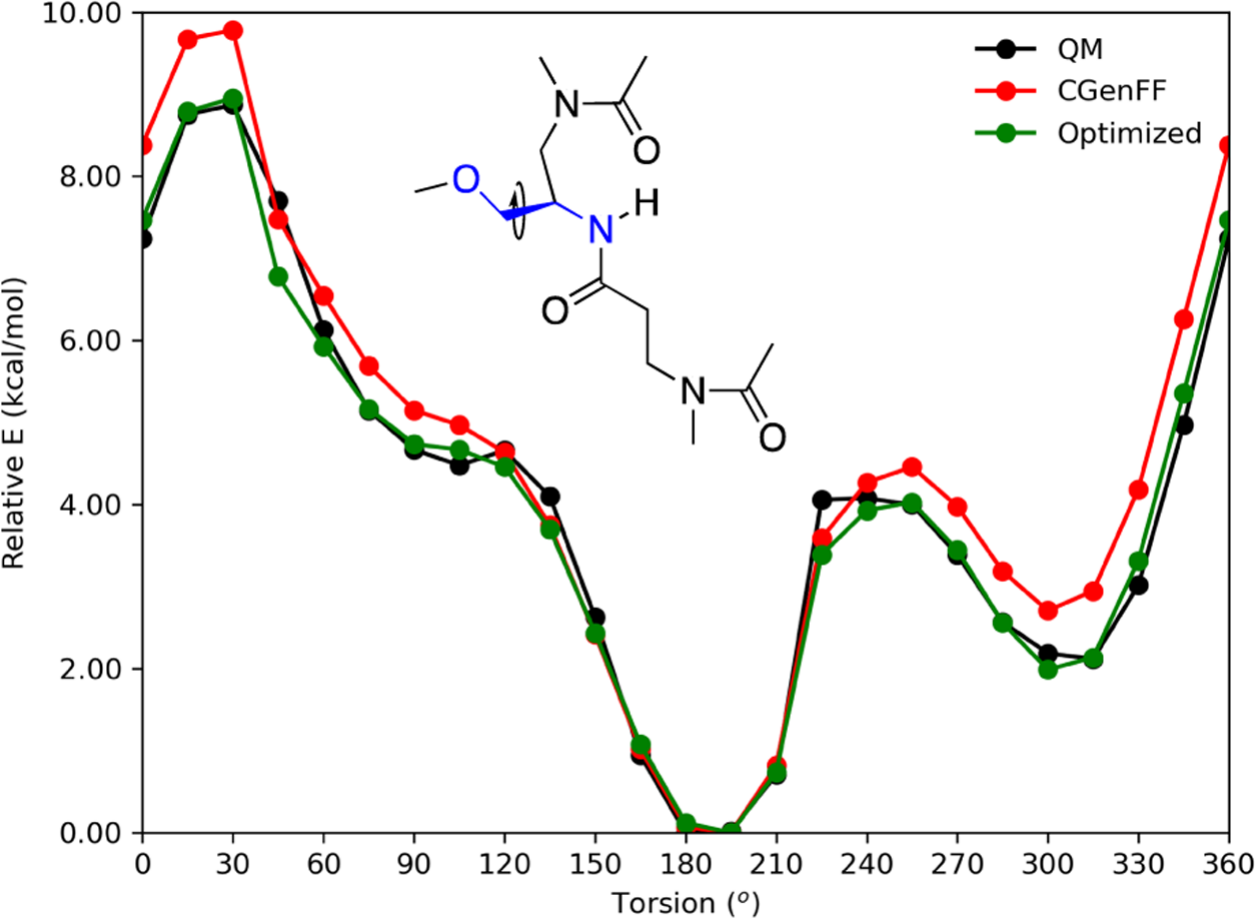
PES for the optimization of the miniPEG linkage to the PNA backbone. The chemical structure of the model compound is shown with the blue atoms detailing the dihedral optimized.

A one-dimensional PES was established by rotating the ∠NC^γ^CO dihedral angle every 15° from 0 to 360°, constraining the dihedral angle at that scan point allowing all other degrees of freedom to minimize (Figure 5). The initial torsional scan was nonperiodic, and the final structure at 360° was a different conformer and lower in relative energy (data not shown). We therefore used the ending geometry as starting coordinates for the PES and rotated the ∠NC^γ^CO dihedral angle backward from 360 to 0°, establishing the QM relative energetic target. Figure 5 details the PES of the torsional scan revealing two minima at 180 and 315° (black curve) corresponding to noncovalent interactions. The minimum at 180° corresponds to two interactions, one hydrogen bond between the methoxy side chain carbonyl and the hydrogen atom on the peptide bond nitrogen at 2.85 Å and the carboxymethyl side chain carbonyl with the α-hydrogen of the subsequent side chain at 3.39 Å. The second minimum corresponds to the hydrogen bond between the hydrogen atom on the peptide nitrogen and the methoxy side chain carbonyl at 2.84 Å.

Coordinates for each QM minimized structure from our QM scan were extracted and used as coordinates for CHARMM minimization. A harmonic force constant restraint of 10^4^ kcal/mol·radian^2^ was placed on the linkage torsion at each dihedral angle, allowing all other degrees of freedom to minimize using the initial parameters for the linkage defined by CgenFF^58−60^ and those from Jasiński et al.^11^ Initially, the parameters derived from the CgenFF web server^58−60^ did well at approximating the QM relative energetics, but the pairwise error was calculated to be 0.53 kcal/mol, a ca. 0.3 kcal/mol difference from our target. Assessing the PES computed from the CHARMM minimizations, the amplitude needed to be fine-tuned. We modified the *k*_χ_ iteratively from 1.80 to 2.40 kcal/mol from the CgenFF-derived parameters to match our QM target data (green curve). Our optimization showed agreement with our QM dataset, lowering the pairwise error from 0.53 to 0.20 kcal/mol. The optimized parameters are tabulated in Table S1 and were used in our MD simulation protocol.

### Baseline Structure and Dynamics of the γPNA Duplex

To determine the perturbation on structure and dynamics the miniPEG-modified γPNA undertakes, three MD simulations were performed on NMR Models 1, 4, and 9 from the 11 deposited for the methyl-modified γPNA duplex (PDB: 2KVJ).^10^ The partial charge assignments for these γPNA models were modified for the covalent bond between the methyl group and the γ-carbon. The γ-carbon atom partial charge was modified from the Jasiński et al.^11^ extension from −0.02 to 0.07*e* by adding the 0.09*e* partial charge from the substituted hydrogen atom onto the γ-carbon, while describing the methyl group modification as a terminal methyl group, with a net zero charge. With these partial charge changes, the NMR γPNA duplexes were solvated in a box of water molecules and simulated for 1 μs at 310 K. The MD simulations were analyzed using RMSD and RMSF on the backbone atoms of the central six residues. A full description of the analysis is provided in the Supplementary Information. For brevity, the RMSD and RMSF quantified for the simulation were low, the RMSD remained steady at ca. 1 Å, and the fluctuations captured by the RMSF were below 1 Å. Agreement with experiment was further verified by PCA, as given in Figure S4.

### Structure and Dynamics of the miniPEG-Modified γPNA Duplex

There is no crystal or NMR structure of the miniPEG-modified γPNA. For initial coordinates, we used six randomly chosen NMR duplexes from the methyl-modified γPNA depositions in the PDB database.^10,81^ The methyl-modified γPNA duplex Models 1, 2, 4, 8, 9, and 10 were mutated to miniPEG-modified duplexes and simulated for 1 μs each to determine the perturbation in structure and dynamics against the NMR simulations.

**Figure 6:**
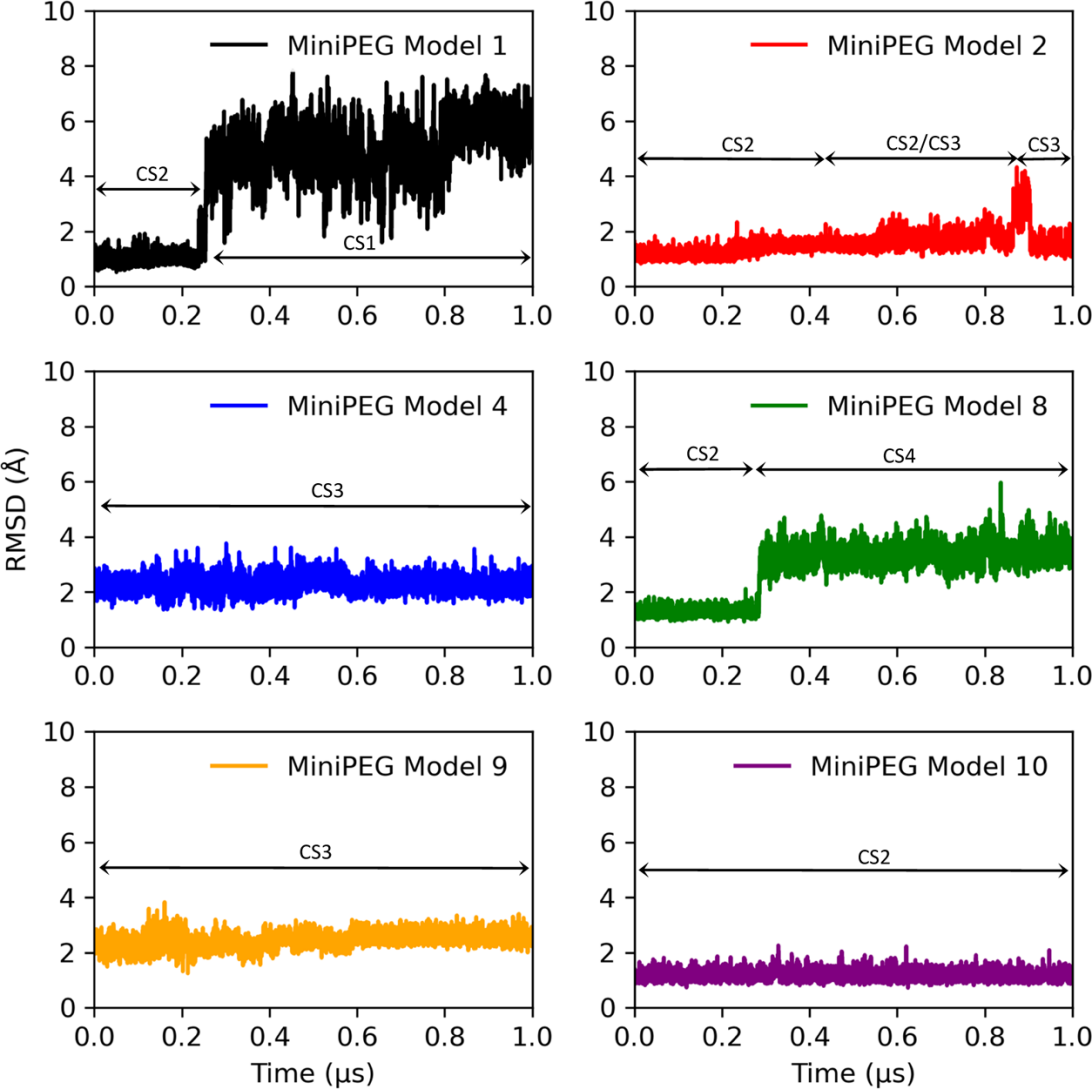
RMSD for the miniPEG-modified γPNA duplexes for the backbone atoms on the central six residues in each simulation.

The RMSD for the backbone atoms was quantified by using the first frame after equilibration of miniPEG Model 1 as the reference point (Figure 6). In five of the six MD simulations, structural variances were seen, where the miniPEG modification was seen to invade the base pairs via the major and minor grooves, leading to deviations captured in the backbone RMSD. The miniPEG Model 10 simulation was consistent with the methyl-modified γPNA simulations, computing a lack of backbone deviations and a steady RMSD at ca. 1 Å. In the miniPEG Model 1 simulation, the RMSD ranged from 1 to 8 Å. The RMSD stayed consistent at ca. 1.0 Å up to 0.25 μs and then rose sharply to ca. 4.0 Å up to 0.8 μs. The backbone became distorted by the miniPEG modification invading the two A–T base pairs via the minor groove. The invasion propagated changes on the backbone, raising the RMSD to ca. 6.0 Å for the remainder of the simulation. In the case of miniPEG Model 8 simulation, the backbone atoms RMSD remained consistent at ca. 1 Å up to 0.28 μs, before rising sharply up to ca. 3.5 Å for the remainder of the simulation. Unlike in the miniPEG Model 1 simulation, in the miniPEG Model 8 simulation, the miniPEG modification increased fraying at the second and second-to-last G–C base pairs by disrupting the WC hydrogen bonds from the major groove. The fraying was primarily consistent for the second base pair. The nucleobases swung out during the simulation and transiently WC base-paired. Although miniPEG Model 4 and 9 simulations remained consistent at an RMSD of ca. 2 Å, the changes in the backbone were localized to the second and second to last G–C base pair. WC hydrogen bonding was disrupted due to the miniPEG modifications invading through the major and minor grooves, leading to significant deformation on the backbone at the terminal ends of the duplex.

**Figure 7:**
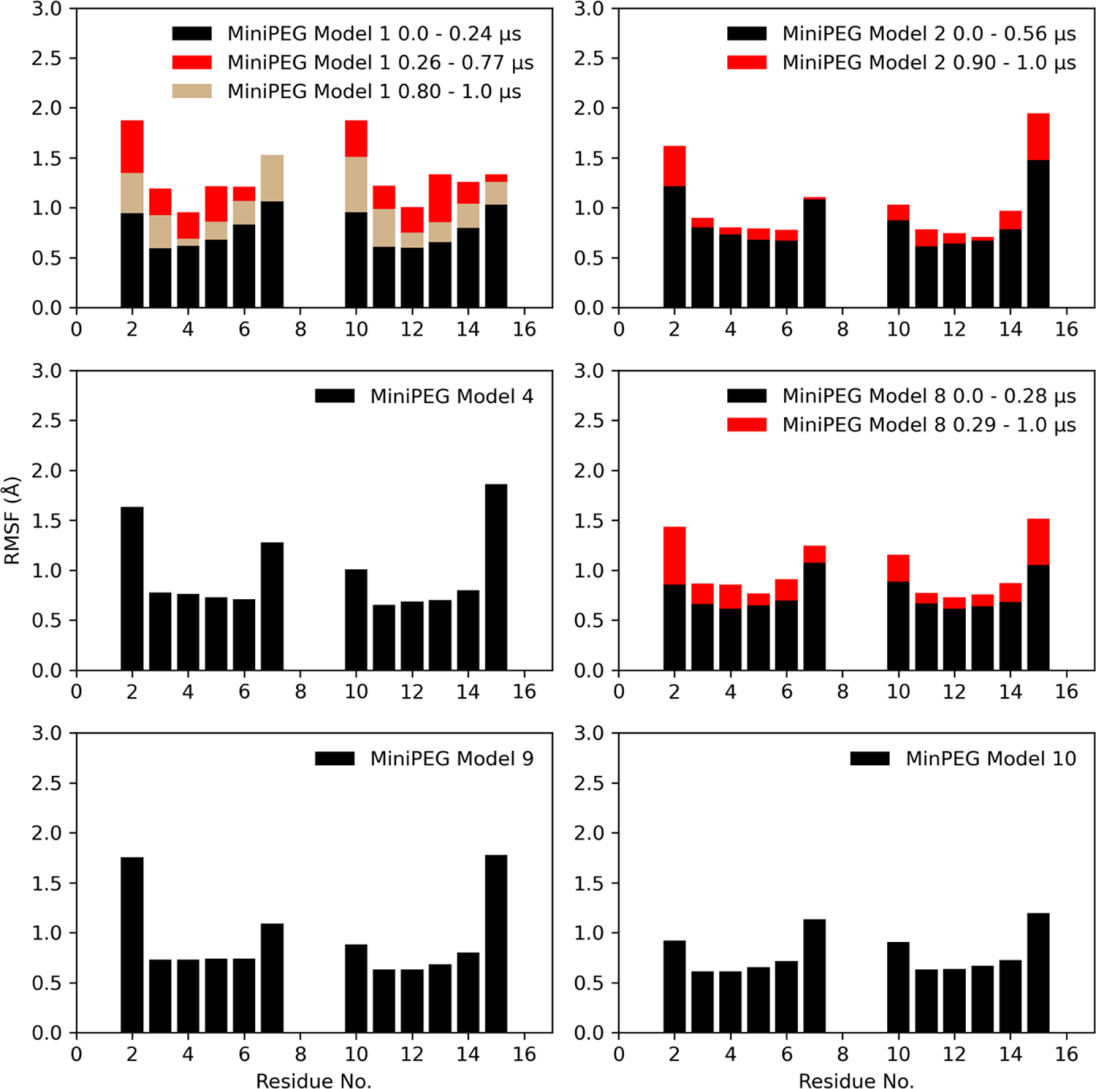
RMSF computed for the methyl-modified γPNA duplex (left) and the miniPEG-modified γPNA duplex (right) for the backbone atoms on the central six residues.

The time intervals that remained steady in the RMSD were used to compute the averaged coordinates of the backbone atoms for our RMSF analysis. The RMSD remained steady for the duration of the miniPEG Model 4, 9, and 10 simulations; thus, the average position for the backbone atoms was computed from the full simulation and used as reference structures for RMSF measurements (Figure 7). The PNA residues closest to the terminal residues tended to have the highest fluctuation, while the central four base pairs had low fluctuation.

Within the first 0.24 and 0.28 μs for miniPEG Model 1 and 8 simulations, the local backbone fluctuations were low, corresponding to slight changes in the backbone atoms, as captured by the RMSD. In the miniPEG Model 1 simulation, the largest fluctuations are seen between 0.26 to 0.77 μs, due to the invasion at the A–T base pairs by miniPEG propagating changes along the backbone, whereas in the miniPEG Model 8 simulation, the fluctuations between 0.29 and 1.0 μs increase primarily for the γPNA residues 2 to 8 due to miniPEG disrupting WC hydrogen bonding through the major grove on the G2–C15 and C7–G10 base pairs. The simulations reveal that there is no specific area of disruption, rather, the miniPEG modification opportunistically disrupts WC hydrogen bonding, leading to changes propagated throughout the backbone.

### Canonical WC Hydrogen Bonding Is Affected by the miniPEG Modification

**Figure 8:**
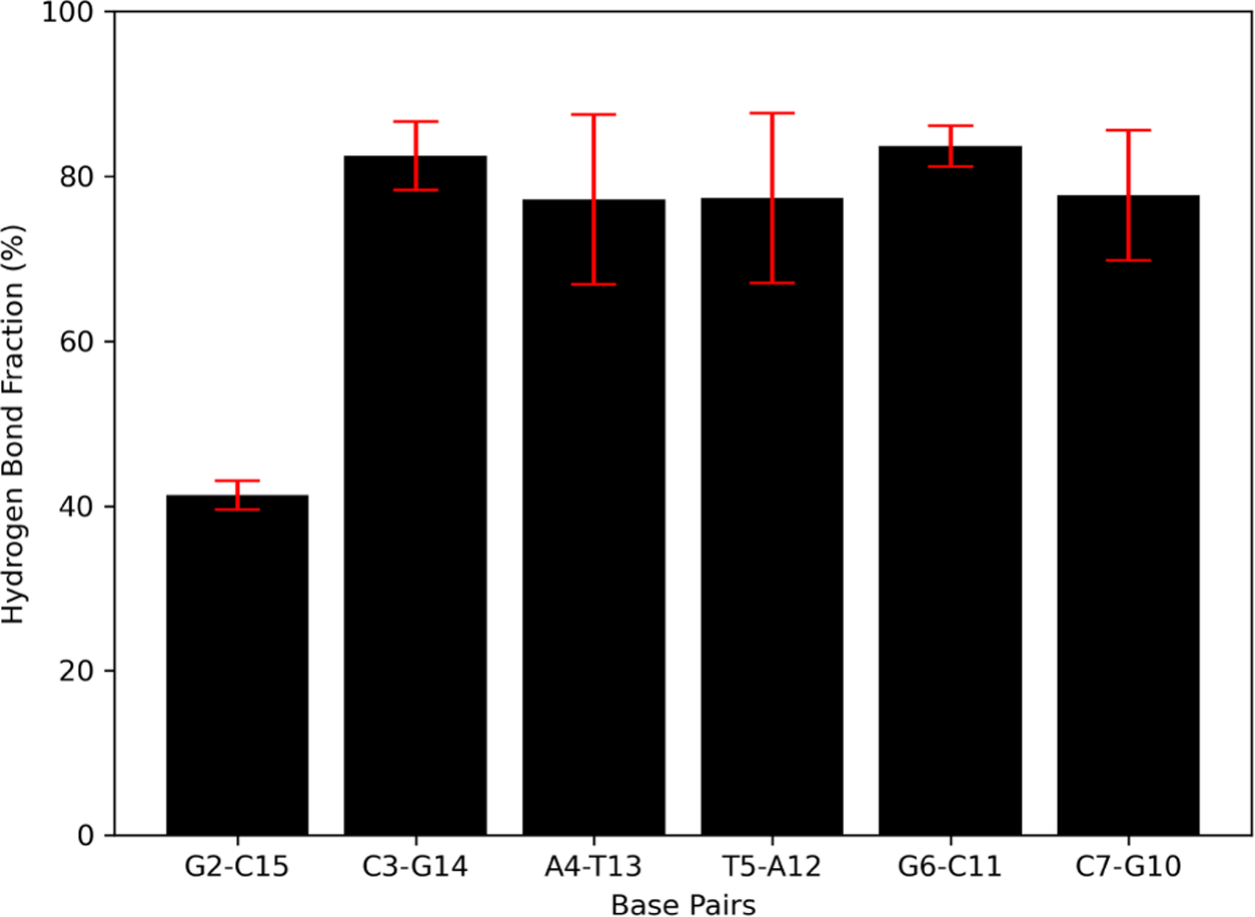
Hydrogen bonding fractions for the central six WC base pairing nucleobases on the miniPEG-modified γPNA duplex. The red bar lines indicate standard deviation for the hydrogen bond fraction analysis across all six 1 μs simulations.

Base pairing between nucleobases plays a significant role in maintaining the overall shape of oligonucleotides.^83^ Upon visualization of the trajectory, the miniPEG modifications were found to invade several areas on the duplexes and disrupt WC hydrogen bonding. Hydrogen bond fractions were assessed across all simulations to determine the WC base pairs most affected by the miniPEG modification (Figure 8). In particular, the G2–C15 WC hydrogen bond saw an ca. 60% reduction in hydrogen bonding. The miniPEG modification opportunistically invaded through both the major and minor grooves in miniPEG Model 2, 4, 8, and 9 simulations due to the fraying of the base G1–C16 base pair. While the C7–G10 did not have a significant reduction in hydrogen bonding, it was variable at 8% due to the same opportunistic invasion of miniPEG. Although disruption was caused at the C8–G9 terminal end, the base pairs leveraged base stacking interactions to maintain WC base pairing and stability.

Hydrogen bonding was most variable in base pairs A4–T13 and T5–A12 at 10%. In a majority of the simulations, A4–T13 and T5–A12 remained as base pairs, but in the miniPEG Model 1 simulation, the miniPEG modifications invaded through the minor groove in the backbone and disrupted the WC base pairing. The A–T base pairs did not reform WC hydrogen bonding for the remainder of the Model 1 simulation. The C3–G14 and G6–C11 WC base pairs remained relatively unperturbed in all simulations, occurring ca. 80% of the simulation with little variance. An interesting case in the minPEG Model 10 simulation is that there was no disruption. The miniPEG modification was extended out to solvent throughout the simulation. Our simulations reveal that the miniPEG modification is opportunistic, inducing duplex dissociation to monomers when possible.

### Torsional Sampling Is Restricted by the miniPEG Modification

**Figure 9:**
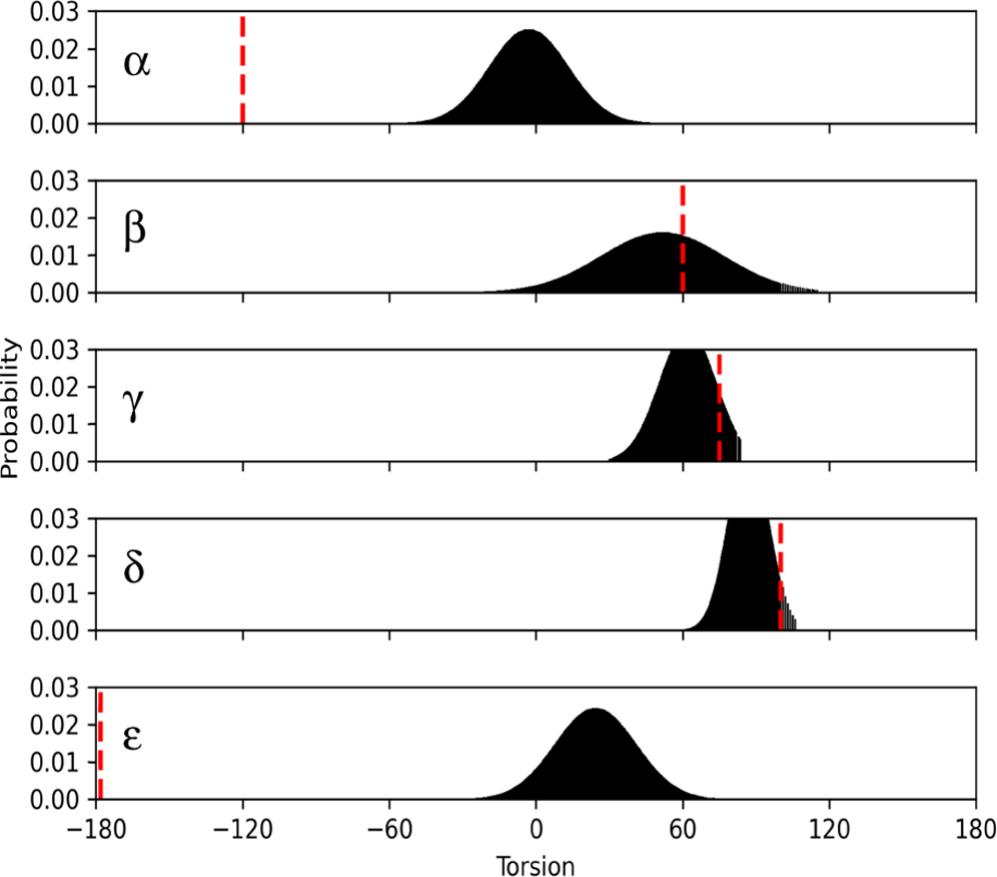
Probability distribution for the backbone torsion values of the miniPEG-modified γPNA. The red dashed line indicates the average torsional value from NMR observations.^10^ See Figure 3 for the chemical structure and torsional definitions.

Developing and optimizing backbone parameters for the miniPEG-modified γPNA revealed the backbone atoms were dynamic. The sampling of the backbone torsions (Figure 9) was determined against those reported for the NMR models^10^ and the CHARMM force field.^11^ The sampling pattern is in good agreement with the β, γ, and δ torsions (Figure 9).

The departures from the current force field^11^ and those reported for the methyl-modified γPNA^10^ are with the α and ε torsions (Figure 9). The miniPEG modification restricted the torsional sampling for the α torsion between −45 and 45°. The CHARMM force field samples the α torsion at ∼90 or ca. −100°, and the values captured for the nonsimulated methyl-modified γPNA NMR models report a distribution steadily at −120°.^10^ The ε torsion, in our simulation samples, is between −30 and ∼60°, with the highest probability being at ∼30°, whereas the CHARMM force field samples 0 and 180°, with a higher propensity for sampling 180°^11^ and the methyl-modified γPNA NMR observations reported ε to primarily be −180°.^10^ We conclude that the differences from the CHARMM force field and the methyl-modified γPNA for the α and ε torsions are due to the proximity to γ-carbon atom. In the case of the α torsion, the γ-carbon atom comprises part of that torsion, influencing the sampling. For the ε torsion, the terminal hydroxyl group on miniPEG transiently hydrogen bonds to the peptide carbonyl and nitrogen groups throughout the simulation, developing a new orientation pattern for ε. Parameterization of the miniPEG-modified backbone captured new variability that characterizes the potential preorganization of α and ε torsions induced by the miniPEG modification.

### Conformational Hierarchy Identifies Duplex Dissociation Induced by the miniPEG Modification

To gain a deeper understanding on the dynamics of the γPNA duplex, PCA was applied on three indices of selectivity over all six simulations: heavy atoms, nucleobase and backbone heavy atoms, and backbone heavy atoms for the central six base pairs. The structures of each simulation were concatenated and aligned against the first frame after equilibration on miniPEG Model 1. The variances were assessed via scree plots (Figures S6 and 10),^76^ computing the backbone atoms retained the largest variance and were used for structural analysis. The dimensions with the most variance consolidated to the first two dimensions on the scree plot, with the largest variance seen in PC1.

**Figure 10:**
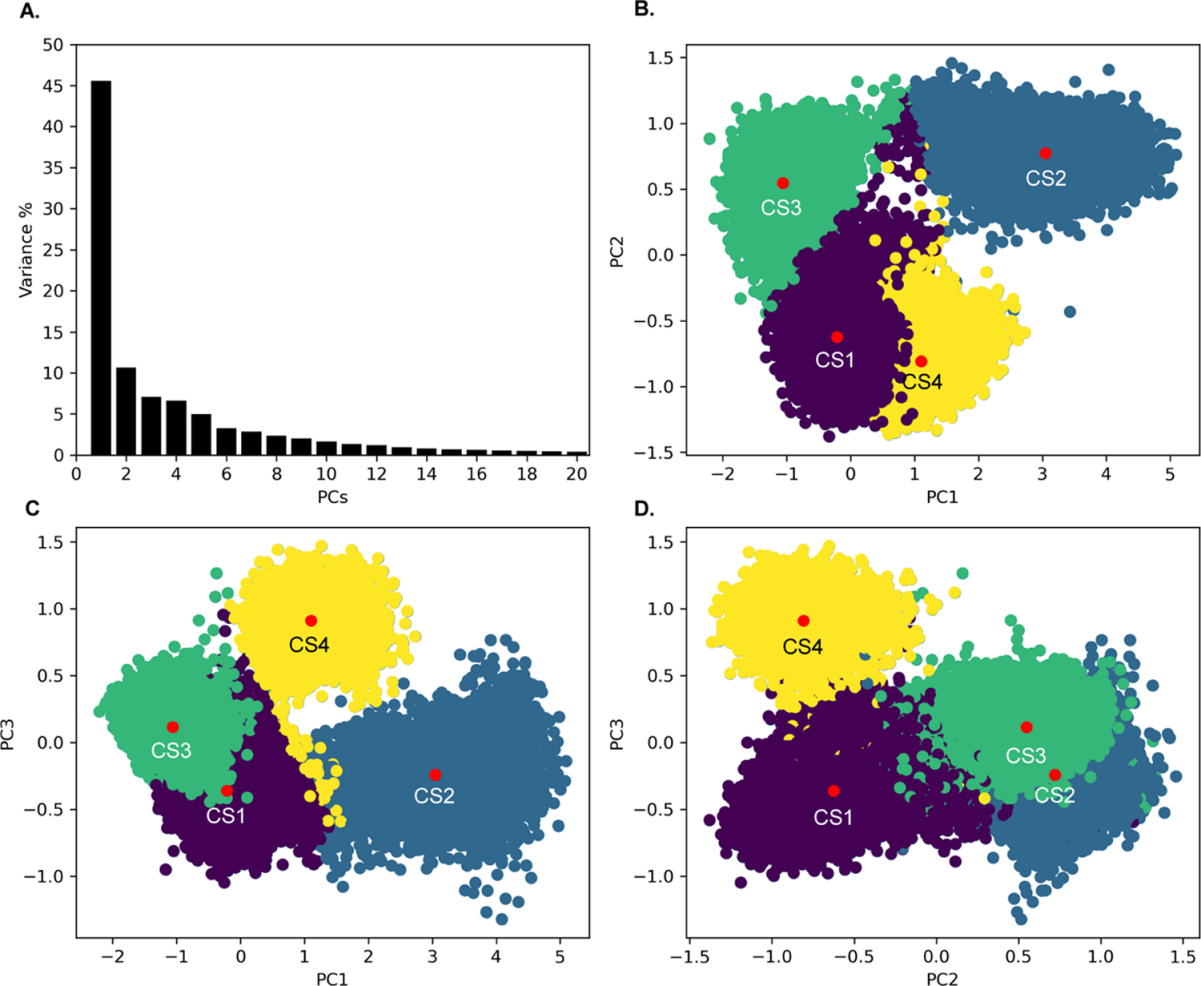
(A) Scree plot of the PCs captured for the six 1 μs MD simulations of miniPEG-modified γPNA duplex backbone atom coordinates. (B–D) 2D projections for the reduced dimensionality of the backbone coordinates (top right and bottom row).

The scree plot for the miniPEG-modified γPNA simulations is markedly different from the methyl-modified γPNA duplex (Figure S4), where the variance across the first three PCs on the NMR model simulations is captured within the first PC for the miniPEG-modified γPNA. Projecting PC1 and PC2, PC1 and PC3, and PC2 and PC3 onto a 2D plane determined four well-defined anisotropic clusters (Figure 10), correlating to four different CSs of the backbone dynamics across the six simulations. The distributions were clustered using the KMeans clustering algorithm, and the coordinates relating to the centroid of each cluster for the miniPEG-modified γPNA duplex were extracted for further analysis. The centroids corresponded to conformations from the miniPEG Model 1, 2, 4, and 8 simulations representing CS 1 to 4, respectively. The CSs demonstrate the predominant conformations adopted by the backbone atoms due to the instability induced by the miniPEG modification.

**Figure 11:**
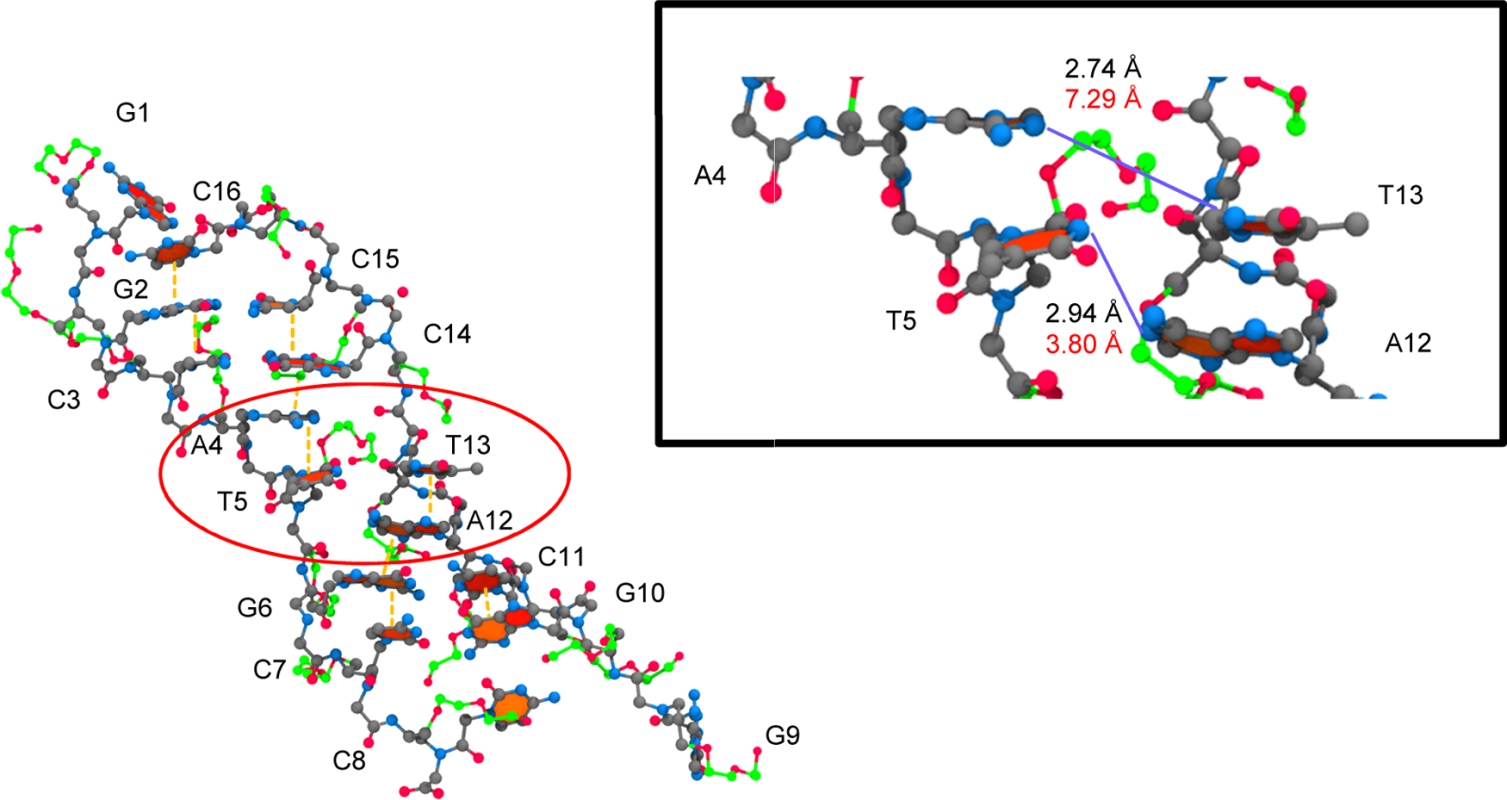
MiniPEG-modified CS1 for the 6 μs simulation of the miniPEG-modified γPNA. The γPNA duplex is represented in CPK, with the carbon atoms for the backbone atoms and nucleobases in gray and the carbon atoms associated with the miniPEG modification in green. All other atoms are colored as follows: blue for nitrogen and red for oxygen. Encircled in red are the areas of destabilization found in duplex. The nucleobases encircled represent destabilization of WC base pairing. The orange dashed line represents base stacking interactions. The blue solid line represents a hydrogen bond.

Figure 11 shows CS1 extracted from the miniPEG Model 1 simulation and the destabilization encircled for the γPNA duplex. In this CS, the miniPEG modification on T5 invades the duplex through the minor groove, disrupting the WC base pairing for the A–T base pairs as shown in the increase in distance between N3 and N1 atoms from 2.74 to 7.29 Å. The miniPEG modification presented an initial case of WC hydrogen bond abstraction. The terminal hydroxyl group (O13′) on the miniPEG modification of A4 hydrogen-bonded with an N2 hydrogen atom on G2. In an attempt for CS1 to stabilize, four base stacking interactions were computed by 3DNA-DSSR^74,75^ between C16–G2–C3, C15–G14–A4–T5, T13–A12–G6–C7, and C11–G10. Although the A–T base pairs became disordered, they intercalated into base stacking interactions between G14–A4 and A12–G6, to stabilize the duplex. Once the miniPEG modification disrupted the A–T base pairs in the Model 1 simulation, the WC base pairs did not reform for the remainder of the simulation.

**Figure 12:**
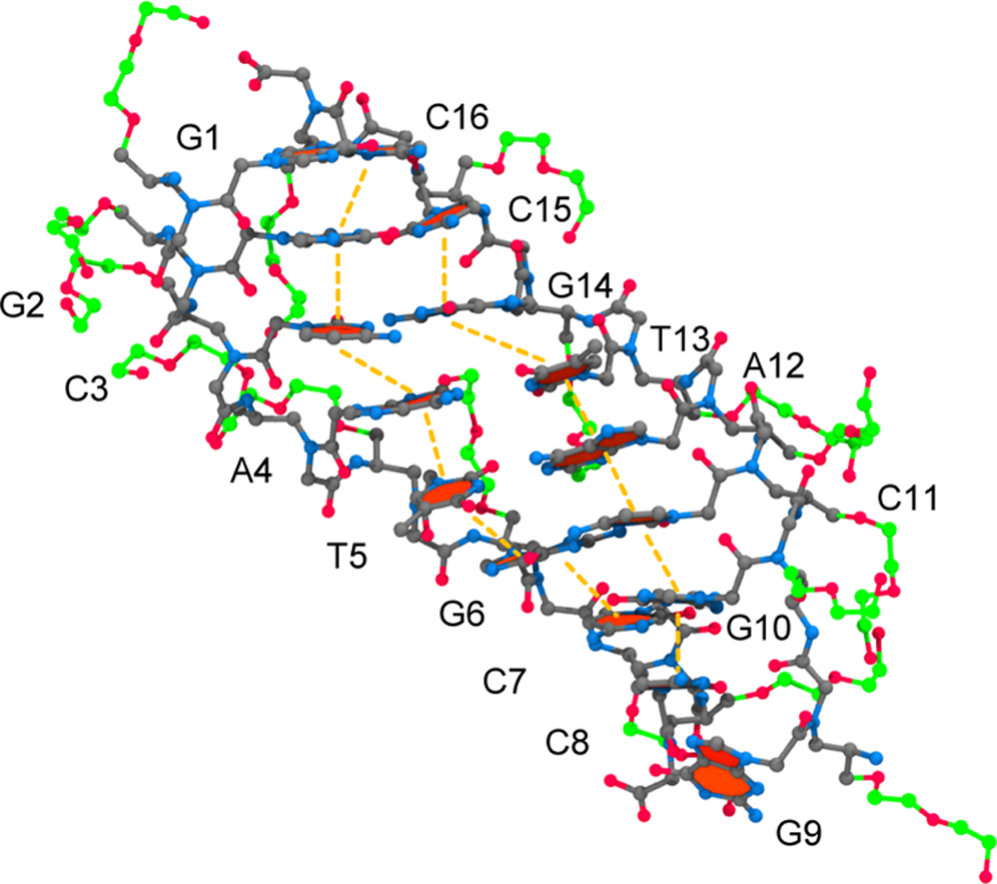
MiniPEG-modified CS2 for the 6 μs simulation of the miniPEG-modified γPNA. The γPNA duplex is represented in CPK, with the carbon atoms for the backbone atoms and nucleobases in gray and the carbon atoms associated with the miniPEG modification in green. All other atoms are colored as follows: blue for nitrogen and red for oxygen. Encircled in red are the areas of destabilization found in duplex. The orange dashed line represents base stacking interactions.

In the miniPEG-modified CS2 (Figure 12) and methyl-modified CS (Figure S5), stacking and WC base pairs are relatively unaffected for the central six base pairs. In CS2, the stacking interactions were found to be well ordered in two base stacks, C16–G2–C3–A4–T5–G6–C7 and C15–G14–T13–A12–C11–G10–C8–G9, where C16 and C8 intercalate between the strands to stabilize the terminal ends. CS2 and the methyl-modified CS were used to calculate helical parameters (*x*-displacement, *y*-displacement, helical rise, inclination, tip, and helical twist) and base step parameters (shift, slide, rise, tilt, roll, and twist) against the A- and B-forms of DNA^83^ and computed averaged NMR helical parameters using 3DNA-DSSR^10,74,75^ (Table 1). The methyl-modified γPNA NMR duplexes were resolved in a general P-form duplex with A-like features in the base region.^10^ In general, PNA duplexes prefer P-form helices, where the helical parameters are characterized by a small twist angle, a large *x*-displacement, and a wide, deep major groove.^10,11,84−87^ The methyl-modified CS structure was determined to have a larger helical rise and helical twist at 2.69 Å and 21.9°, respectively, whereas in CS2, the helical rise was found to be 3.02 Å and the helical twist at 26.1°. The computed helical rise and twists for the CS2 and CS structures were inconsistent with the NMR models and deviated toward A-DNA at 2.9 Å and 32.7°,^83^ especially seen for CS2. P-form duplexes are characterized by a large *x*-displacement, as seen in the nonsimulated NMR models at −9.81 ± 3.83 Å, but the *x*-displacements for the CSs progressively deviated toward A-DNA, at −4.1°,^83^ with the increase in the size of the modification.

**Table 1:** Averaged Helical and Base Step Parameters Comparison between the NMR Model Used, the Methyl-Modified γPNA CS, and the miniPEG-Modified γPNA CS1

	nonsimulated methyl-modified γPNA structures	methyl-modified γPNA CS	miniPEG-modified γPNA CS2
Helical Parameters			
*x*-displacement (Å)	–9.81 ± 3.83	–7.37	–3.67
*y*-displacement (Å)	–0.24 ± 2.69	0.85	0.03
helical rise (Å)	2.50 ± 0.62	2.69	3.02
inclination (deg)	17.4 ± 9.85	16.7	5.33
tip (deg)	–1.50 ± 11.9	–5.25	3.76
helical twist (deg)	17.5 ± 3.13	22.0	26.1
Base Step Parameters			
shift (Å)	0.14 ± 0.44	–0.12	–0.25
slide (Å)	–2.09 ± 0.32	–1.75	–1.37
rise (Å)	3.35 ± 0.14	3.42	3.32
tilt (deg)	0.12 ± 2.81	2.02	–2.44
roll (deg)	5.14 ± 2.96	6.22	2.03
twist (deg)	16.3 ± 3.25	20.3	24.1

The NMR models were resolved to have a helical bend toward the major groove at 23.2 ± 0.94°. The NMR Model 1, 4, and 9 simulations were consistent with the NMR models computing a helical bend of 18.7 ± 0.56°, while the CS had a 19.0° bend. For the miniPEG-modified CS2 structure, we compared the helical bend against the miniPEG Model 10 simulation. The RMSD for the backbone atoms was low relative to all other simulations, retaining base stacking and WC interactions well through the entirety of the simulation. The helical bend for the miniPEG Model 10 simulation was found to be 7.65 ± 0.50°, a reduction of over half than that seen from all three NMR simulations. For CS2, the helical bend was computed to be 6.26°, lower than the expected value seen from the Model 10 simulation. We suspect the deviations from the P-form duplex was due, in part, as the γPNA duplex was resolved as having A-DNA-like features in the base pairing region and that the CHARMM force field captures base geometries for PNA/A-DNA systems well, deviating toward the A-DNA.^5,11^ The miniPEG modification led to the deviation toward the A-DNA form as a potential method for stabilization.

**Figure 13:**
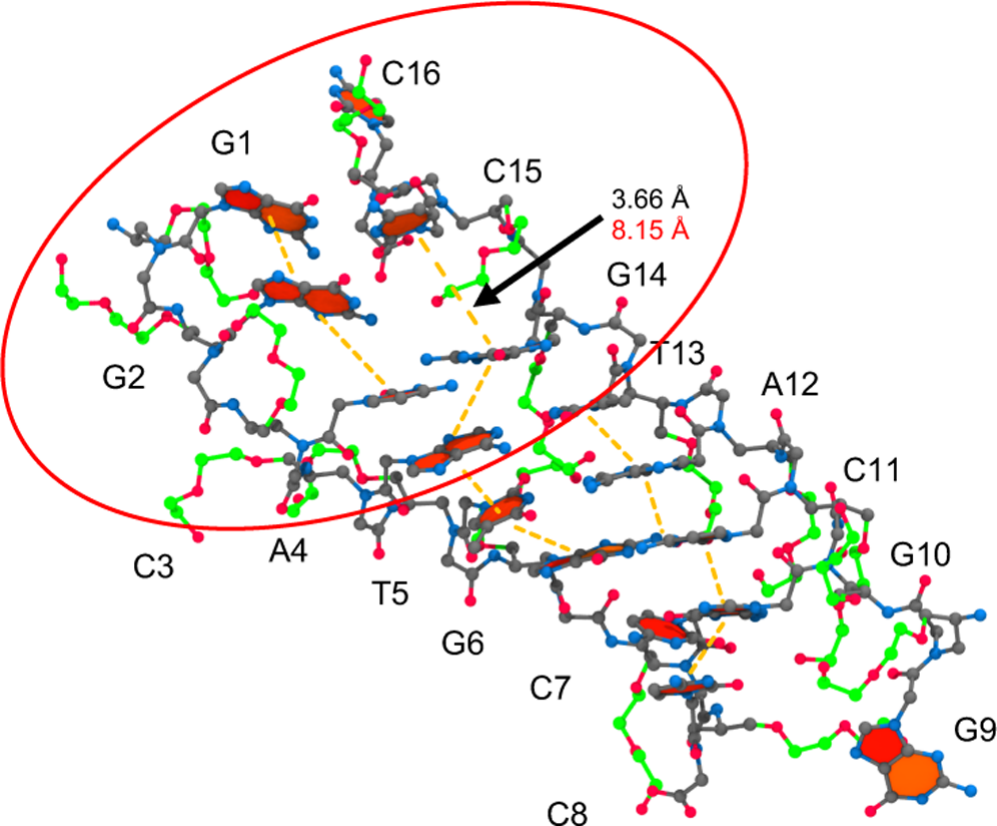
MiniPEG-modified CS3 for the 6 μs simulation of the miniPEG-modified γPNA. The γPNA duplex is represented in CPK, with the carbon atoms for the backbone atoms and nucleobases in gray and the carbon atoms associated with the miniPEG modification in green. All other atoms are colored as follows: blue for nitrogen and red for oxygen. Encircled in red are the areas of destabilization found in duplex. The orange dashed line represents base stacking interactions.

Transitioning from CS2 to CS3, the flexibility of the miniPEG modification is exemplified (Figure 13). The destabilization of the duplex occurs through the major groove on the second and second-to-last base pairs as opposed to the minor groove at the A–T base pairs as seen in CS1. The miniPEG modification on C15 transitions from the minor groove to the major groove, causing C15 to base pair reshuffle to WC base pair with G1. In the process, miniPEG replaced a missing hydrogen bond on G2 by hydrogen bonding with O13' on C15, increasing the base stacking distance from 3.66 Å to 8.15 Å. On G10, the miniPEG modification sterically hinders G9 from WC base pairing to C8. The invasion of miniPEG on the terminal ends of the duplex led to a loss of sequential base stacking interactions into three stacks: G1–G2–C3, G14–A4–T5–G6, and T13–A12–C11–G10–C8. Similarly, to CS1, there are intercalating base stack interactions between G14–A4 and G10–C7 to stabilize the dissociating duplex. The C7–G10 base pair retains the canonical WC base pair hydrogen bonds, with an average donor-hydrogen-acceptor angle of 158° and a dipole−π T-stacking interaction occurs between the N1 hydrogen atom on G10 to the aromatic ring of C7 at a hydrogen−π cloud distance of 3.28 Å. The dipole−π T-stacking interaction causes C7 to adopt a gauche(-) conformation for the carbonyl methyl side chain, connecting the backbone and the nucleobases, as opposed to the trans conformation seen for the remaining nucleobases. The simultaneous T-stack between C7 and G10, base stack between C8 and G10, and WC base paring between G10 and C7 pinch the terminal end of the duplex, preventing further invasion by the miniPEG modifications.

**Figure 14:**
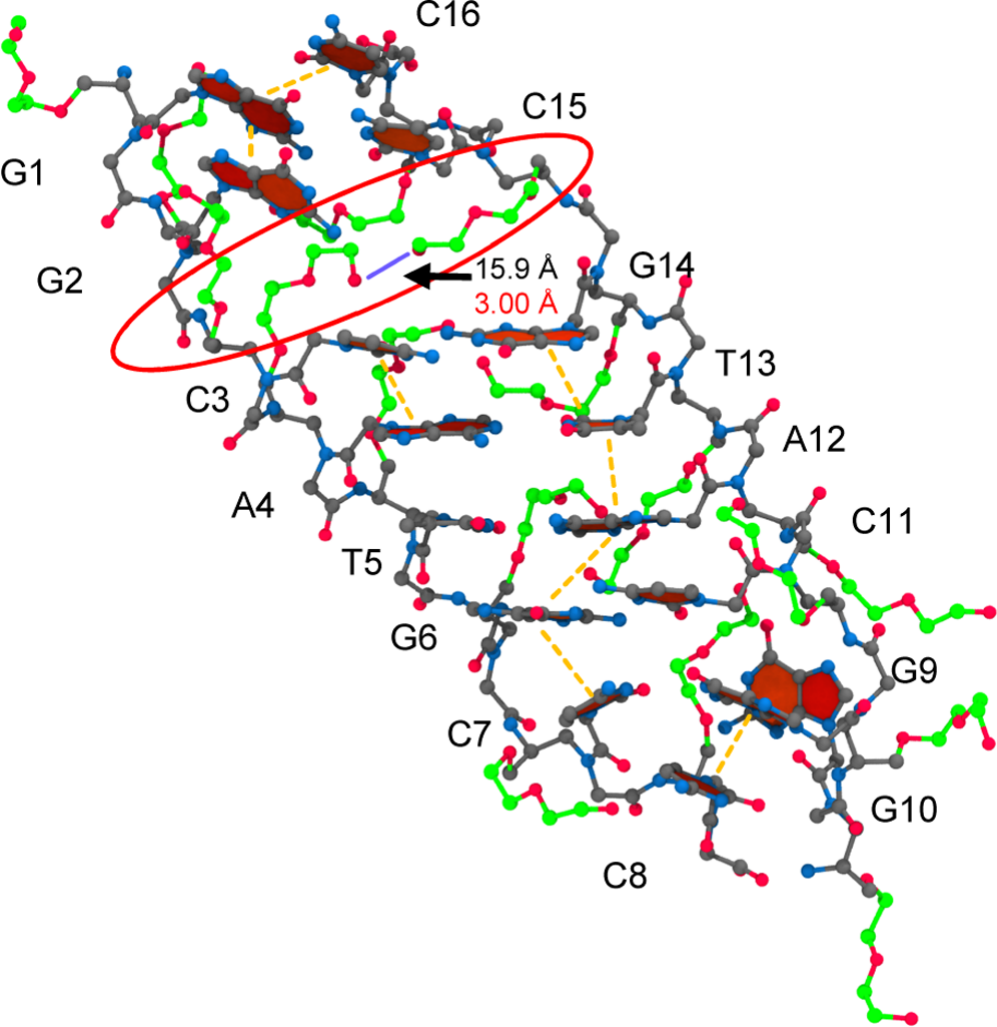
CS4 for the 6 μs simulation of the miniPEG-modified γPNA. The γPNA duplex is represented in CPK, with the carbon atoms for the backbone atoms and nucleobases in gray and the carbon atoms associated with the miniPEG modification in green. All other atoms are colored as follows: blue for nitrogen and red for oxygen. Encircled in red are the areas of destabilization found in duplex. The orange dashed line represents base stacking interactions. The blue line represents a hydrogen bond.

In the final CS, CS4, the duplex is the most destabilized. In this conformation, the miniPEG modifications on C15 and A4 cooperatively invade the duplex from the major and minor grooves, forming a hydrogen bond with the terminal hydroxyl groups with a distance of 3.00 Å, as shown in Figure 14. The miniPEG modification on C15 once again caused base pair reshuffling, forming WC base pairs with C15 and G1. The destabilization on the terminal end on G9 swings out to solvent, where the miniPEG modification on C8 sterically hinders its WC base pairing with C9. The cooperative destabilization caused a significant loss of sequential base stacking interactions forming four stacks: C16–G1–G2, C3–A4, G14–T13–A12–G6–C1, and G10–C8. The base stacks showed three intercalated base stacking interactions between C16–G1, A12–G6, and G10–C8, stabilizing the disrupted duplex. The fraying at the terminal end seen in G9 allowed for the retention of the WC base pair between G10 and C7 and allowed for the intercalated base stacking interaction with C8. We suspect with further simulation, the cooperative miniPEG invasion would further continue, further dissociating the duplex into single strands, consistent with experiment.^17^ In culmination, the destabilized CS duplexes, variable hydrogen bonding, and large RMSDs suggest that miniPEG-modified duplexes are unstable and must exist as single strands in solution.

## Conclusions

The miniPEG modification is widely reported in making the γPNA more soluble, capable of invading any sequence of B-DNA.^17,23,88^ Moreover, application of the miniPEG modification onto the γPNA has been reported to aid in bonding to the RNA CAG repeats reported for Huntington’s Disease.^57^ Yet, molecular modeling of miniPEG-modified γPNAs to its genetic targets often replaces the miniPEG modification with a methyl modification for ease of computational modeling, with the assumption that the miniPEG modification can be overlooked in binding to the genetic target.^56,57,88^ From our simulations of the methyl-modified γPNA, the assumption would be correct, methyl-modified γPNAs do not have overall large changes in structure and have no effect on binding. However, the lack of inclusion of the miniPEG modification in computational studies left open questions on the implication it poses on structure and dynamics. To establish the structural and dynamic differences, we established parameters for the CHARMM force field using QM as our reference data. Our parameterization methodology was based on fitting torsional parameters to match our QM relative energetic PES, optimizing the electrostatic charges at the γ-position carbon in coordination with established precedent.^63^ Our parameters were optimized to be within 0.2 kcal/mol of our QM target data and used in MD simulations.

We performed six 1 μs long simulations on the miniPEG-modified γPNA duplex and three 1 μs long simulations on the methyl-modified γPNA duplexes. Good agreement between our simulated NMR duplexes and the methyl-modified NMR models was computed, but structure and dynamics for the miniPEG-modified duplex were computed to be different. Investigating the changes, it became evident that the miniPEG modification invaded the duplexes at two points, extending the fraying from the terminal ends and at A–T base pairs, causing large structural changes on the backbone atoms. Through hydrogen fraction data of the simulation, we deduced that the G2–C15 base pair was the most affected, reducing WC hydrogen bonding by ca. 60%. The WC base pairing at the A–T base pairs was found to be reduced only ca. 20%, but were variable, showing that they were impaired by the miniPEG modification. The invasion propagated changes on the backbone atoms altering the backbone torsional sampling from the current CHARMM force field and NMR observations. A new sampling pattern was determined for the α and ε torsions restricting the γPNA backbone to new conformations in the duplex at ∼0 and 30°, respectively. Four CSs were computed using PCA on the backbone atoms, demonstrating the onset of duplex dissociation as a consequence of the miniPEG modification, reducing stable base stacking. With a longer simulation time, the miniPEG modifications on the γPNA duplexes would continue to invade the duplex, leading to preference as single-stranded γPNA molecules. To complement the insight of miniPEG-modified γPNA structure and dynamics, the new miniPEG force field parameters allow for further exploration of such modified γPNA monomers as potential therapeutic agents against genetic diseases.

## Supporting Information Available

Optimized parameters used in the miniPEG-modified γPNA simulations, analysis of the three 1 μs miniPEG-modified γPNA simulations, topology and parameters for the methyl- and miniPEG-modified γPNA nucleobases, Gaussian archive for geometry-optimized model compound, starting coordinates for all miniPEG-modified γPNA simulations, Gaussian input decks for geometry optimization and dihedral scanning of the model compound, and NAMD example input decks for miniPEG-modified γPNA equilibration and the first 200 ns simulations (PDF)
