# Supplementary material for: Parameterization of the miniPEG-Modified γPNA Backbone: Toward Induced γPNA Duplex Dissociation
Source: J Chem Theory Comput. 2023 May 17;19(11):3346–58. doi: 10.1021/acs.jctc.2c01163 (PMC10269335; doi:10.1021/acs.jctc.2c01163)
Supplement: Supplementary file 1 — ct2c01163_si_001.pdf [file ct2c01163_si_001.pdf]

# Supplementary Information for: Parameterization of the miniPEG-modified γPNA backbone: Towards induced γPNA duplex dissociation

**Authors:** Angel Tamez,[1] Lennart Nilsson,[2] Mihaela-Rita Mihailescu,[1] Jeffrey D. Evanseck[1]*

**Addresses**:

1. Center for Computational Sciences and the Department of Chemistry and Biochemistry at Duquesne University, Pittsburgh, PA, 15282, USA
2. Department of Biosciences and Nutrition, Karolinska Institute, Solnavägen 1, 171 77 Solna, Sweden

# Table of Contents


Table S1. Optimized parameters for the bond, electrostatic, angle, and torsional parameters on the methyl- and miniPEG-modified γPNA backbone. .......... 3

Methyl-modified γPNA Simulation .......... 3

Figure S1. RMSD comparison using the NMR structure and the frame immediately following equilibration for NMR Model 1. .......... 4

Figure S2. RMSD comparison for NMR Models 1, 4, and 9 using the first frame following equilibration of NMR Model 1 as the reference. .......... 4

Figure S3. RMSF comparison for NMR Models 1, 4, and 9. .......... 5

Figure S4. A. Scree plot of the PCs captured for the three one-microsecond MD simulations of methyl-modified γPNA duplex backbone atom coordinates. B.-D. 2D projections for the reduced dimensionality of the backbone coordinates (top right and bottom row). .......... 6

Figure S5. Representative structure of the methyl–modified γPNA duplex. The duplex is represented in licorice and the atoms are colored as follows: grey for carbon, red for oxygen, blue for nitrogen and white for hydrogen. .......... 7

Figure S6. Scree plot assessments for all heavy atoms (left) and nucleobase and backbone heavy atoms (right) for all six miniPEG-modified γPNA simulations. .......... 9

Topology and Parameters for the methyl-modified γPNA backbone. Current Nomenclature remained the same from the most recent force field improvements .......... 9

Table S2. Estimated parameters used for methyl-modified γPNA simulations. .......... 9

RESI GPN .......... 11

RESI CPN .......... 13

RESI TPN .......... 15

RESI UPN .......... 17

RESI APN .......... 19

Topology and Parameters for the left-handed miniPEG-modified γPNA nucleobases. Current Nomenclature remained the same from the most recent force fieldimprovements .......... 21

Table S3. Estimated parameters for the miniPEG-modified γPNA. .......... 21

RESI GGPN .......... 25

RESI GCPN .......... 28

RESI GTPN .......... 31

RESI GUPN .......... 34

RESI GAPN .......... 37

Archive for the optimized model compound used for our QM model during the parameterization procedure. .......... 40

Coordinates for the miniPEG-modified γPNA MD starting structures........................................ 41
Model 1 ........................................................................................................................ 41
Model 2 ........................................................................................................................ 53
Model 4 ........................................................................................................................ 65
Model 8 ........................................................................................................................ 77
Model 9 ........................................................................................................................ 89
Model 10 ...................................................................................................................... 101
Input deck for model compound optimization. ............................................................ 113
Input deck for model compound dihedral scan. ........................................................... 114
NAMD configuration file for minimization and equilibration of miniPEG-modified 2KVJ. .... 115
NAMD configuration file for a 200-nanosecond production run of miniPEG-modified 2KVJ. 117

**Table S1.** Optimized parameters for the bond, electrostatic, angle, and torsional parameters on the methyl- and miniPEG-modified γPNA backbone.

| Electrostatic | | | |
|---|---|---|---|
| Atom Type | $q_i$ ($e$) | | |
| CG311 | 0.07 | | |
| CG321 | 0.08 | | |
| **Torsional** | | | |
| Atom Type | $k_\chi$ (kcal/mol) | $n$ | $\delta$ (°) |
| NH1-CG311-CG321-OC30A | 2.40 | 1 | 180.0 |

## Methyl-modified γPNA Simulation

Three NMR duplexes were selected and underwent one microsecond long MD simulations at 310 K and 1 atm using the *NPT* ensemble. The NMR Model 1 simulation was analyzed two ways, due to the availability of experimental observation. First, the RMSD of the central six base pair backbone atoms were compared against the NMR model starting coordinates and the frame immediately following equilibration structure to determine changes induced by the current parameters in the CHARMM force field.[1] The RMSD on the backbone of the γPNA duplex over the one microsecond simulation shows low RMSD using both references, with average RMSD of 1.01 ± 0.20 Å and 0.93 ± 0.19 Å against the NMR model and the structure immediately after equilibration, respectively (Figure S1).

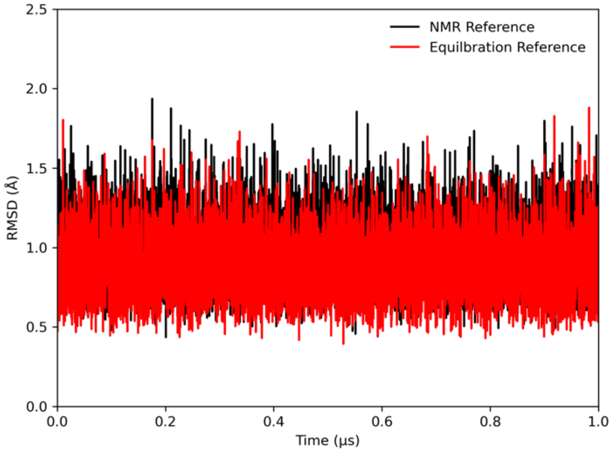


**Figure S1.** RMSD comparison using the NMR structure and the frame 4 immediately following equilibration for NMR Model 1.

Due to the low RMSD calculation from using the frame immediately following equilibration, we utilized the frame following equilibration for NMR Model 1 as our reference point for the RMSD analysis of NMR Models 4 and 9 simulations.

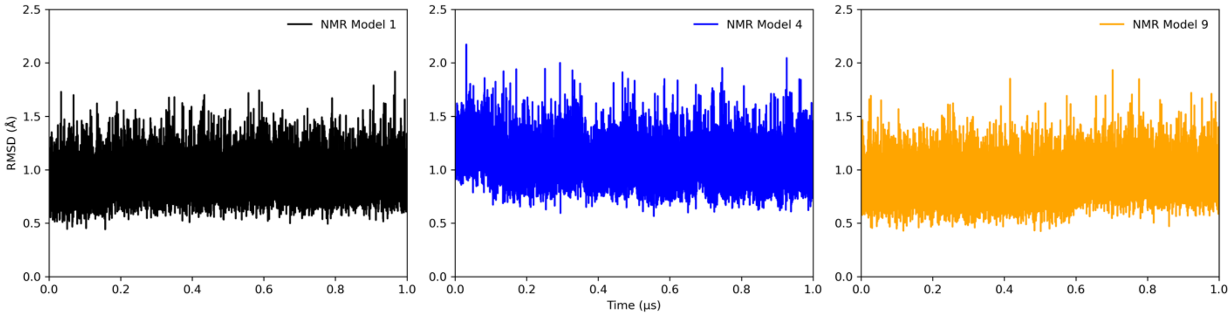


**Figure S2.** RMSD comparison for NMR Models 1, 4, and 9 using the first frame following equilibration of NMR Model 1 as the reference.

In all three cases, the RMSD was low (Figure S2), computing an average of 1.08 ± 0.19 Å and 0.92 ± 0.19 Å for the Model 4 and 9 simulations, respectively. The averaged position for the backbone atoms were used from the full simulations as the reference, due to the steady state of the RMSDs (Figure S3).

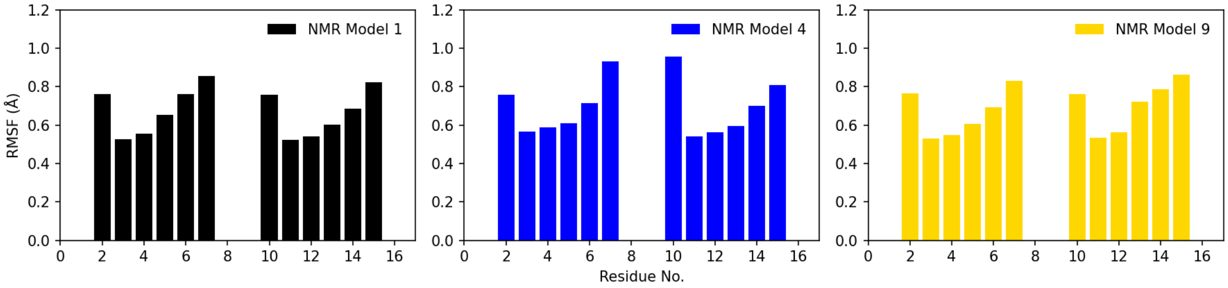


**Figure S3.** RMSF comparison for NMR Models 1, 4, and 9.

The fluctuation was low as well, under 1 Å, in each simulation, further validating the CHARMM force field preserved the backbone atoms for the γPNA duplex.

To further investigate the dynamics of the backbone, PCA was employed on the backbone atom coordinates for the central six base pairs on the γPNA duplex. The python package MDTraj[2] was used to handle the trajectory and determine a conformational hierarchy with Scikit Learn.[3] All NMR models (PDB ID: 2KVJ)[4] and three one microsecond simulation structures were concatenated for analysis. The variance for the coordinates of the backbone atoms were evaluated across 20 PCs, with the first three PCs capturing over 50%, and the following PCs capturing less than 10% (Figure S4).

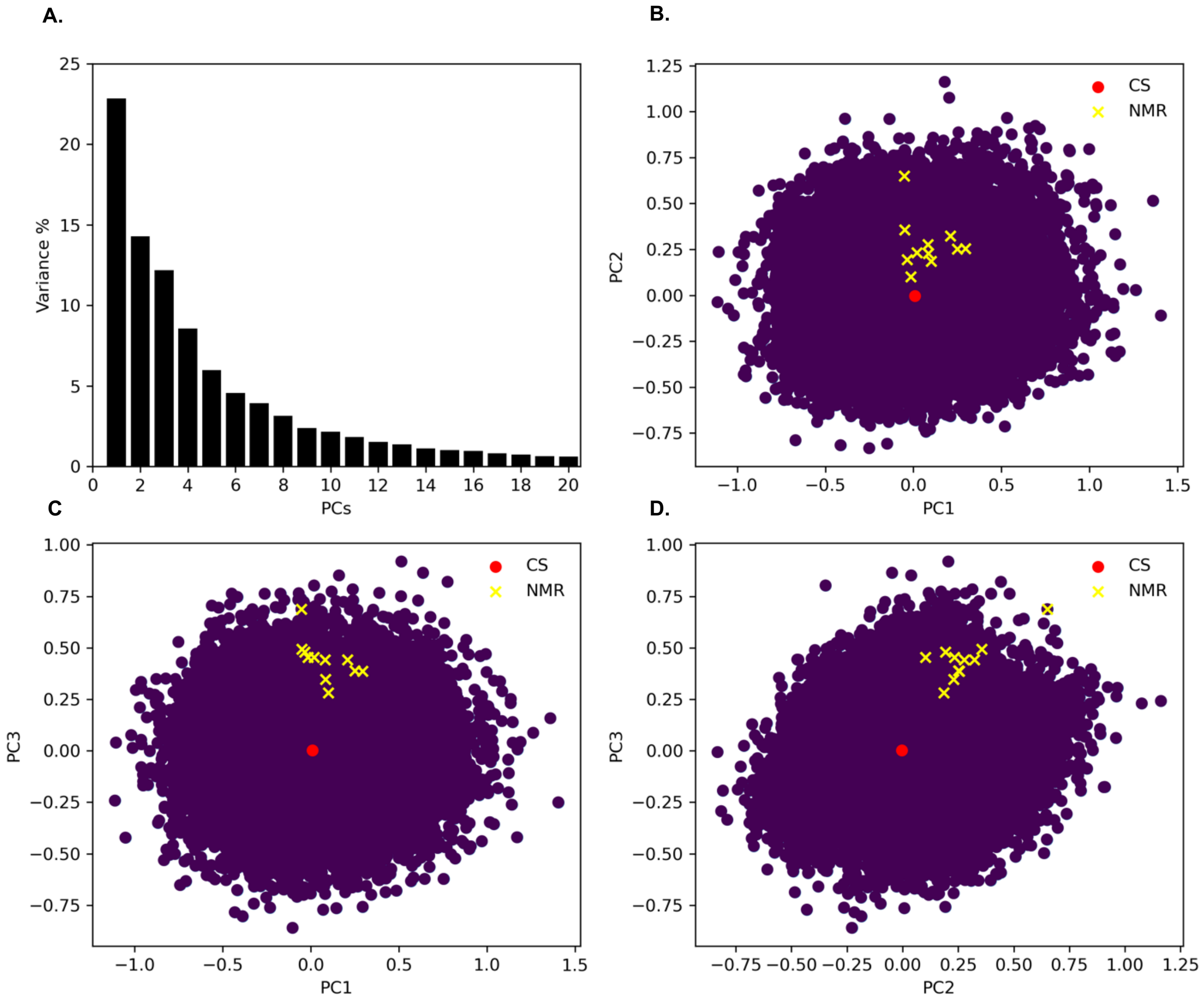


**Figure S4. A.** Scree plot of the PCs captured for the three one-microsecond MD simulations of methyl-modified γPNA duplex backbone atom coordinates. **B.-D.** 2D projections for the reduced dimensionality of the backbone coordinates (top right and bottom row).

Little correlation in the backbone atoms across the PCs was determined (Figure S4). The reduced dimensionality for the Cartesian coordinates corresponding to PCs one and two, one and three, and two and three were projected onto a 2D plane, revealing an isotropic distribution of the functional dynamics for the backbone where the NMR backbone coordinates were revisited across all three simulations (Figure S4). The 2D data points for the PC projections were clustered using the KMeans clustering algorithm encoded in Scikit-learn,[3] deriving the centroid of the distribution as the representative structure, CS, for the methyl-modified γPNA simulation (Figure S5).

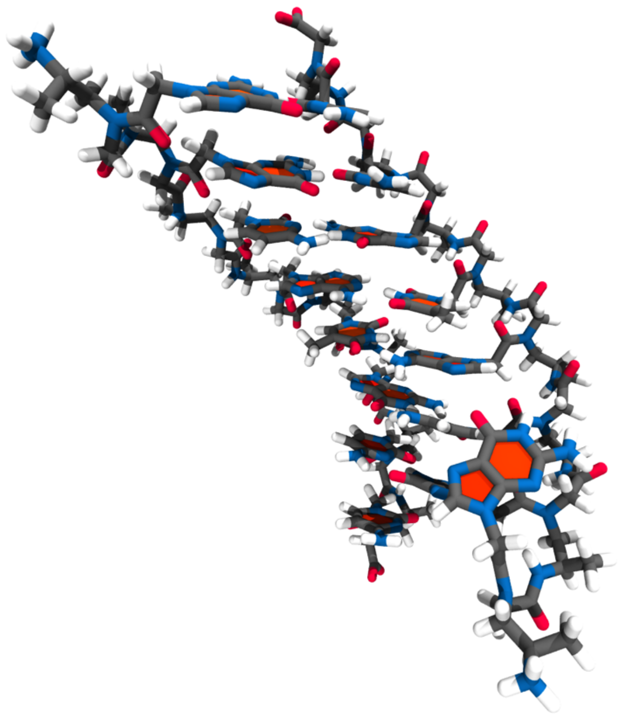

**Figure S5.** Representative structure of the methyl–modified γPNA duplex. The duplex is represented in licorice and the atoms are colored as follows: grey for carbon, red for oxygen, blue for nitrogen and white for hydrogen.

The helical bend was measured between the non-simulated NMR models, simulated NMR models 1, 4, and 9, and the CS. The non-simulated NMR models were computed to have a helical bend at $23.2 \pm 0.94^{o}$, whereas the simulated NMR helical bend was found to be $18.7 \pm 0.56^{o}$, and the helical bend for the CS structure was consistent with our MD simulations at $19.0^{o}$. The marginal helical bend discrepancies between our simulated structures and the non-simulated structures are suspected to arise from the methods used to resolve the NMR structures. The structures deposited into the PDB database[5] were simulated using Nuclear Overhauser Effects (NOEs) restraints at 300 K, whereas our simulations were not restrained and performed at 310 K. The structural deviations are minor and well within the standard deviation captured in the RMSD analysis.

Base pairing between the nucleosides plays a significant role in maintaining the overall shape of oligonucleotides.[6] There is limited availability on the NOEs, as such a direct comparison on NOE violations cannot be determined.[4] Two amino-imino resonances were reported for the G2-

C15 and G6-C11 hydrogen bonds found in the 2D $^1$H NMR, representing canonical Watson-Crick (WC) base pairing.[4] Within our simulations, G2 and C15 have a hydrogen bond fraction of 85.9% ± 5.47%, while in the CS structure, a hydrogen bond between G2-N2 and C15-O2 was calculated. In the case for G6 and C11, the WC base pairing computed a hydrogen bond fraction of 87.8% ± 3.19%, while in the CS structure, the G-C base pairs retained all three canonical hydrogen bonds. The base stacking interactions were well-ordered, found to be at two stacks G1-G2-C3-A4-T5-G6-C7-C8 and C16-C15-G14-T13-A12-C11-G10 and represented a stable duplex. Overall The current CHARMM force field did well in maintaining the overall geometry for the methyl-modified duplex against experimental observation. Our findings engendered confidence in progression towards defining the structural and dynamics differences with the miniPEG-modified duplex using our optimized parameters and the current CHARMM force field.[1]

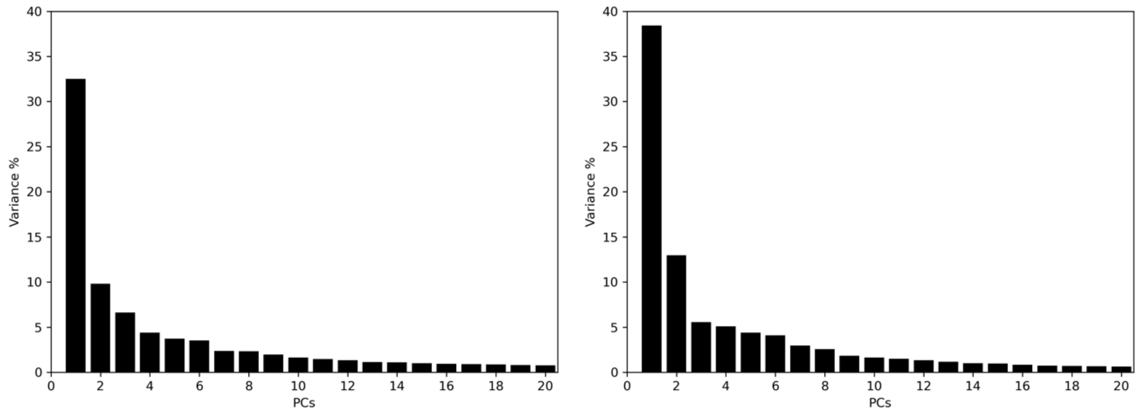


**Figure S6.** Scree plot assessments for all heavy atoms (left) and nucleobase and backbone heavy atoms (right) for all six miniPEG-modified γPNA simulations.

## Topology and Parameters for the methyl-modified γPNA backbone. Current Nomenclature remained the same from the most recent force field improvements.[1]

**Table S2.** Estimated parameters used for methyl-modified γPNA simulations.

| | | | | | | |
|---|---|---|---|---|---|---|
| **Bond** | | | | | | |
| Atom Types | | $k_b$ (kcal/mol) | $b_0$ (Å) | | | |
| CT2 | HB1 | 330 | 1.08 | | | |
| **Angle** | | | | | | |
| Atom Types | | | $k_\theta$ (kcal/mol) | $\theta_0$ (º) | | |
| CT2P | CT2 | CT3 | 58.35 | 113.6 | | |
| HC | NH3 | H | 51.5 | 107.5 | | |
| H | NH3 | CT1 | 35 | 117 | | |
| CT2 | CT2 | CT3 | 58 | 115 | | |
| NH1 | CT2 | CT3 | 70 | 113.5 | | |
| HB2 | CT2 | CT3 | 50 | 109.5 | | |
| NH3 | CT2 | CT2P | 67.7 | 110 | | |
| CT2P | CT2 | HB1 | 35 | 111 | | |
| NH1 | CT2 | HB1 | 48 | 108 | | |
| HB1 | CT2 | CT3 | 35 | 111 | | |
| **Dihedral** | | | | | | |
| Atom Types | | | | $k_\chi$ (kcal/mol) | $n$ | $\delta$ (º) |
| NH3 | CT2 | CT2P | N | 0.4 | 1 | 0 |
| NH3 | CT2 | CT2P | HB2 | 0.15 | 3 | 0 |
| C | NH1 | CT2 | HB1 | 0 | 1 | 0 |
| HB2 | CT2 | CT2 | HB1 | 0.2 | 2 | 0 |
| HB2 | CT2P | CT2 | HB1 | 0.2 | 2 | 0 |

| | | | | | | |
|---|---|---|---|---|---|---|
| H | NH1 | CT2 | HB1 | 0 | 1 | 0 |
| C | NH1 | CT2 | CT3 | 0.96 | 2 | 0 |
| C | NH1 | CT2 | CT3 | 0.17 | 3 | 0 |
| C | NH1 | CT2 | CT3 | 0.41 | 4 | 0 |
| C | NH1 | CT2 | CT3 | 0.27 | 5 | 0 |
| N | CT2P | CT2 | CT3 | 1.59 | 1 | 180 |
| N | CT2P | CT2 | CT3 | 0.72 | 3 | 0 |
| N | CT2P | CT2 | CT3 | 0.03 | 4 | 0 |
| HB2 | CT2P | CT2 | CT3 | 0.19 | 3 | 0 |
| N | CT2P | CT2 | HB1 | 0.15 | 3 | 0 |

```
RESI GPN            0.00 !
GROUP                    !
ATOM C      C       0.51 !
ATOM O1'    O      -0.51 !
GROUP                    !
ATOM C2'    CT2    -0.03 !
ATOM H2'    HB2     0.09 !   \   H1'
ATOM H2''   HB2     0.09 !    \ /
ATOM N2'    N      -0.33 !     N   C6M
ATOM C5'    CT2P    0.00 ! mj   \  /
ATOM H5'    HB2     0.09 !       C6'      Base
ATOM H5''   HB2     0.09 !       |        /
GROUP                    !       C5'     C4'
ATOM N      NH1    -0.47 !        \     /
ATOM H1'    H       0.31 !         N2'-C3'
ATOM C6'    CT2     0.07 !        /     \\
ATOM H6'    HB2     0.09 !       C2'     O3'
                         !       |
GROUP                    !       C
ATOM C3'    CC      0.43 !      // \
ATOM O3'    O      -0.54 !     O1'  \
ATOM C4'    CT2    -0.07 !
ATOM H4'    HA2     0.09 !
ATOM H4''   HA2     0.09 !
GROUP                    !
ATOM C6M    CT3    -0.27 !
ATOM H6M    HA3     0.09 !
ATOM H6M'   HA3     0.09 !
ATOM H6M''  HA3     0.09 !
!BASE ATOMS STARTS HERE. !
GROUP                    !
ATOM N9     NN2B   -0.02 !
ATOM C4     CN5     0.26 !
ATOM N2     NN1    -0.68 !
ATOM H21    HN1     0.32 !
ATOM H22    HN1     0.35 !
ATOM N3     NN3G   -0.74 !
ATOM C2     CN2     0.75
ATOM N1     NN2G   -0.34
ATOM H1     HN2     0.26
ATOM C6     CN1     0.54
ATOM O6     ON1    -0.51
ATOM C5     CN5G    0.00
ATOM N7     NN4    -0.60
ATOM C8     CN4     0.25
ATOM H8     HN3     0.16
BOND +N   C     C   O1'   C   C2'   C2' H2'  C2' H2''  C2' N2'
BOND N2'  C5'   C5' H5'   C5' H5''  C6' C5'  C6' H6'   C6' C6M
BOND C6'  N     N   H1'   C6M H6M   C6M H6M' C6M H6M''
BOND N2'  C3'   C3' O3'   C3' C4'   C4' H4'  C4' H4''
BOND C4'  N9        N9   C4        N9   C8        C4   N3
BOND C4   C5        N3   C2        C2   N2        C2   N1        N2   H21
BOND N2   H22       N1   H1        N1   C6        C6   O6        C6   C5
BOND C5   N7        N7   C8        C8   H8
IMPR C2   N3   N1   N2        C6   N1   C5   O6        N2   H21  C2   H22
!impropers for PNA backbone.
IMPR N    -C   C6'  H1'       C    O1'  C2'  +N
IMPR C3'  O3'  C4'  N2'       N2'  C2'  C5'  C3'
```

```
DONO H21  N2
DONO H22  N2
DONO H1   N1
DONO H1'  N1
ACCE O6   C6
ACCE N3
ACCE N7
ACCE O1'  C
ACCE O3'  C3'

!PNA backbone
IC O1'  C    C2'  N2'  1.23  119.76   172.34  115.72  1.46
IC C    C2'  N2'  C3'  1.49  115.72   -85.69  115.69  1.36
IC C2'  N2'  C5'  C6'  1.46  116.43    79.13  110.77  1.55
IC H2'  C2'  N2'  C3'  1.08  106.87    35.19  115.69  1.23
IC H2'' C2'  N2'  C5'  1.08  106.87   -18.93  118.21  1.47
IC N2'  C5'  C6'  N    1.47  113.26    70.08  111.16  1.44
IC C5'  C6'  N    H1'  1.53  111.16    67.33  115.23  1.00
IC H5'  C5'  C6'  N    1.08  109.06  -170.24  111.16  1.44
IC H5'' C5'  C6'  N    1.08  109.06   -49.59  111.16  1.44
IC C6'  C5'  N2'  C3'  1.53  113.26  -108.76  125.61  1.36
IC H6'  C6'  C5'  N2'  1.08  109.44   -49.50  113.26  1.47
!IC H6'' C6'  C5'  N2'  1.08  109.44  -170.33  113.26  1.47
IC N    C6'  C5'  N2'  1.44  111.16    70.00  113.26  1.47
IC H1'  N    C6'  C5'  1.00  115.23    67.33  111.16  1.53
IC C3'  N2'  C2'  H2'' 1.36  115.69   153.43  106.87  1.08
IC O3'  C3'  N2'  C2'  1.23  120.78     6.15  115.69  1.46
IC C4'  C3'  N2'  C2'  1.52  119.02  -173.47  116.54  1.46
IC H4'  C4'  C3'  O3'  1.11  109.30  -113.00  119.96  1.24
IC H4'' C4'  C3'  N2'  1.11  109.30   -52.69  119.02  1.36
IC C6M  C6'  C5'  N2'  1.52  108.80  -176.38  109.07  1.48
IC H6M   C6M  C6'  C5'  1.08  109.47   -67.99  108.80  1.48
IC H6M'  C6M  C6'  C5'  1.08  109.47   176.01  108.80  1.48
IC H6M'' C6M  C6'  C5'  1.08  109.47    52.01  108.80  1.48
!To connect with previous
IC N    -C  -C2' -H2'' 1.35  118.45   110.49  108.45  1.08
IC C6'  N   -C   -O1'  1.45  122.56     6.77  121.77  1.23
IC C5'  C6'  N   -C    1.52  113.10  -115.86  122.56  1.35
!Adopted from GUA, first three lines adjusted to PNA
BILD O3'  C3'  C4'  N9     1.23    120.40   10.91  109.15   1.46
BILD H4'  C4'  N9   C4     1.11    109.86 -150.27  124.65   1.36
BILD C4'  C4   *N9  C8     1.46    124.65  177.35  107.10   1.374
BILD C4   N9   C8   N7     1.377   106.0     0.0   113.5    1.304
BILD C8   N9   C4   C5     1.374   106.0     0.0   105.6    1.377
BILD N9   C5   *C4  N3     1.377   105.6   180.0   128.4    1.355
BILD C5   C4   N3   C2     1.377   128.4     0.0   111.8    1.327
BILD C4   N3   C2   N1     1.355   111.8     0.0   124.0    1.375
BILD N1   N3   *C2  N2     1.375   124.0   180.0   119.7    1.341
BILD N3   C2   N2   H21    1.327   119.7   180.0   127.0    1.01
BILD H21  C2   *N2  H22    1.01    127.0  -180.0   116.5    1.01
BILD N3   C2   N1   C6     1.327   124.0     0.0   124.9    1.393
BILD C6   C2   *N1  H1     1.393   124.9   180.0   117.4    1.03
BILD C5   N1   *C6  O6     1.415   111.7   180.0   120.0    1.239
BILD N9   N7   *C8  H8     0.0       0.0   180.0     0.0    0.0
!PATCHING FIRST NT LAST CT
```

**RESI CPN** 0.00

```
GROUP                   !
ATOM C      C       0.51 !
ATOM O1'    O      -0.51 !
GROUP                    !
ATOM C2'    CT2    -0.03 !
ATOM H2'    HB2     0.09 !   \   H1'
ATOM H2''   HB2     0.09 !    \ /
ATOM N2'    N      -0.33 !     N   C6M
ATOM C5'    CT2P    0.00 ! mj   \  /
ATOM H5'    HB2     0.09 !       C6'      Base
ATOM H5''   HB2     0.09 !       |         /
GROUP                    !       C5'     C4'
ATOM N      NH1    -0.47 !        \     /
ATOM H1'    H       0.31 !         N2'-C3'
ATOM C6'    CT2     0.07 !        /     \\
ATOM H6'    HB2     0.09 !       C2'     O3'
                         !       |
GROUP                    !       C
ATOM C3'    CC      0.43 !      // \
ATOM O3'    O      -0.54 !     O1'  \
ATOM C4'    CT2    -0.07 !
ATOM H4'    HA2     0.09 !
ATOM H4''   HA2     0.09 !
GROUP                    !
ATOM C6M    CT3    -0.27 !
ATOM H6M    HA3     0.09 !
ATOM H6M'   HA3     0.09 !
ATOM H6M''  HA3     0.09 !
!BASE ATOMS STARTS HERE.
GROUP
ATOM N1     NN2    -0.13
ATOM C6     CN3     0.05
ATOM H6     HN3     0.17
ATOM C5     CN3    -0.13
ATOM H5     HN3     0.07
ATOM C2     CN1     0.52
ATOM O2     ON1C   -0.49
ATOM N3     NN3    -0.66
ATOM C4     CN2     0.65
ATOM N4     NN1    -0.75
ATOM H41    HN1     0.37
ATOM H42    HN1     0.33
BOND +N   C     C   O1'   C   C2'   C2' H2'  C2' H2''  C2' N2'
BOND N2'  C5'   C5' H5'   C5' H5''  C6' C5'  C6' H6'   C6' C6M
BOND C6'  N     N   H1'   C6M H6M   C6M H6M' C6M H6M''
BOND N2'  C3'   C3' O3'   C3' C4'   C4' H4'  C4' H4''
BOND C4'  N1    N1   C2        N1   C6        C2   O2
BOND C2   N3    N3   C4        C4   N4        N4   H41        N4   H42
BOND C4   C5    C5   C6        C5   H5        C6   H6
IMPR C2   N1   N3   O2        C4   N3   C5   N4
!impropers for PNA backbone.
IMPR N    -C   C6'  H1'       C    O1'  C2'  +N
IMPR C3'  O3'  C4'  N2'       N2'  C2'  C5'  C3'
DONO H42  N4
DONO H41  N4
ACCE O2
ACCE N3
```

```
DONO H1'  N
ACCE O1'  C
ACCE O3'  C3'

!PNA backbone
IC O1'  C    C2'  N2'  1.23  119.76   172.34  115.72  1.46
IC C    C2'  N2'  C3'  1.49  115.72   -85.69  115.69  1.36
IC C2'  N2'  C5'  C6'  1.46  116.43    79.13  110.77  1.55
IC H2'  C2'  N2'  C3'  1.08  106.87    35.19  115.69  1.23
IC H2'' C2'  N2'  C5'  1.08  106.87   -18.93  118.21  1.47
IC N2'  C5'  C6'  N    1.47  113.26    70.08  111.16  1.44
IC C5'  C6'  N    H1'  1.53  111.16    67.33  115.23  1.00
IC H5'  C5'  C6'  N    1.08  109.06  -170.24  111.16  1.44
IC H5'' C5'  C6'  N    1.08  109.06   -49.59  111.16  1.44
IC C6'  C5'  N2'  C3'  1.53  113.26  -108.76  125.61  1.36
IC H6'  C6'  C5'  N2'  1.08  109.44   -49.50  113.26  1.47
!IC H6'' C6'  C5'  N2'  1.08  109.44  -170.33  113.26  1.47
IC N    C6'  C5'  N2'  1.44  111.16    70.00  113.26  1.47
IC H1'  N    C6'  C5'  1.00  115.23    67.33  111.16  1.53
IC C3'  N2'  C2'  H2'' 1.36  115.69   153.43  106.87  1.08
IC O3'  C3'  N2'  C2'  1.23  120.78     6.15  115.69  1.46
IC C4'  C3'  N2'  C2'  1.52  119.02  -173.47  116.54  1.46
IC H4'  C4'  C3'  O3'  1.11  109.30  -113.00  119.96  1.24
IC H4'' C4'  C3'  N2'  1.11  109.30   -52.69  119.02  1.36
IC C6M  C6'  C5'  N2'  1.52  108.80  -176.38  109.07  1.48
IC H6M   C6M  C6'  C5'  1.08  109.47   -67.99  108.80  1.48
IC H6M'  C6M  C6'  C5'  1.08  109.47   176.01  108.80  1.48
IC H6M'' C6M  C6'  C5'  1.08  109.47    52.01  108.80  1.48
!To connect with previous
IC N    -C  -C2' -H2'' 1.35  118.45   110.49  108.45  1.08
IC C6'  N   -C   -O1'  1.45  122.56     6.77  121.77  1.23
IC C5'  C6'  N   -C    1.52  113.10  -115.86  122.56  1.35
!Adopted from CYT, first three lines adjusted to PNA
BILD N2'  C3'  C4'  N1     1.36    118.87 -175.00  110.85   1.47
BILD H4'  C4'  N1   C2     1.11    109.43 -158.84  117.57   1.40
BILD C4'  C2   *N1  C6     1.47    117.57  173.53  120.6    1.364
BILD C2   N1   C6   C5     1.399   120.6     0.0   121.0    1.337
BILD C6   N1   C2   N3     1.364   120.6     0.0   118.9    1.356
BILD N1   N3   *C2  O2     1.399   118.9   180.0   121.9    1.237
BILD N1   C2   N3   C4     1.399   118.9     0.0   120.0    1.334
BILD C5   N3   *C4  N4     1.426   121.8   180.00  118.9    1.337
BILD N3   C4   N4   H41    1.337   117.9     0.00  118.9    1.01
BILD H41  C4   *N4  H42    1.01    118.9   180.00  120.7    1.01
BILD C6   C4   *C5  H5     0.0       0.0   180.0     0.0    0.0
BILD N1   C5   *C6  H6     0.0       0.0   180.0     0.0    0.0
!PATCHING FIRST NT LAST CT
```

```
RESI TPN           0.00  !
GROUP                    !
ATOM C      C       0.51 !
ATOM O1'    O      -0.51 !
GROUP                    !
ATOM C2'    CT2    -0.03 !
ATOM H2'    HB2     0.09 !   \   H1'
ATOM H2''   HB2     0.09 !    \ /
ATOM N2'    N      -0.33 !     N   C6M
ATOM C5'    CT2P    0.00 ! mj   \  /
ATOM H5'    HB2     0.09 !       C6'      Base
ATOM H5''   HB2     0.09 !       |        /
GROUP                    !       C5'     C4'
ATOM N      NH1    -0.47 !        \     /
ATOM H1'    H       0.31 !         N2'-C3'
ATOM C6'    CT2     0.07 !        /     \\
ATOM H6'    HB2     0.09 !       C2'     O3'
                         !       |
GROUP                    !       C
ATOM C3'    CC      0.43 !      // \
ATOM O3'    O      -0.54 !     O1'  \
ATOM C4'    CT2    -0.07 !
ATOM H4'    HA2     0.09 !
ATOM H4''   HA2     0.09 !
GROUP                    !
ATOM C6M    CT3    -0.27 !
ATOM H6M    HA3     0.09 !
ATOM H6M'   HA3     0.09 !
ATOM H6M''  HA3     0.09 !
!BASE ATOMS STARTS HERE.
GROUP
ATOM N1     NN2B   -0.34
ATOM C6     CN3     0.17
ATOM H6     HN3     0.17
ATOM C2     CN1T    0.51
ATOM O2     ON1    -0.41
ATOM N3     NN2U   -0.46
ATOM H3     HN2     0.36
ATOM C4     CN1     0.50
ATOM O4     ON1    -0.45
ATOM C5     CN3T   -0.15
ATOM C5M    CN9    -0.11
ATOM H51    HN9     0.07
ATOM H52    HN9     0.07
ATOM H53    HN9     0.07
BOND +N  C   C  O1'   C  C2'   C2' H2'  C2' H2''  C2' N2'
BOND N2' C5'   C5' H5'   C5' H5''  C6' C5'  C6' H6'   C6' C6M
BOND C6' N      N  H1'    C6M H6M   C6M H6M' C6M H6M''
BOND N2' C3'   C3' O3'   C3' C4'   C4' H4'  C4' H4''
BOND C4'  N1         N1   C2         N1   C6         C2   O2
BOND C2   N3         N3   H3         N3   C4         C4   O4         C4   C5
BOND C5   C5M        C5   C6         C6   H6         C5M  H51        C5M  H52
BOND C5M  H53
IMPR C2   N1   N3   O2        C4   N3   C5   O4         C5   C4   C6   C5M
!impropers for PNA backbone.
IMPR N    -C   C6'  H1'       C    O1'  C2'  +N
IMPR C3'  O3'  C4'  N2'       N2'  C2'  C5'  C3'
DONO H3   N3
```

```
ACCE O2   C2
ACCE O4   C4
DONO H1'  N
ACCE O1'  C
ACCE O3'  C3'

!PNA backbone
IC O1'  C    C2'  N2'  1.23  119.76   172.34  115.72  1.46
IC C    C2'  N2'  C3'  1.49  115.72   -85.69  115.69  1.36
IC C2'  N2'  C5'  C6'  1.46  116.43    79.13  110.77  1.55
IC H2'  C2'  N2'  C3'  1.08  106.87    35.19  115.69  1.23
IC H2'' C2'  N2'  C5'  1.08  106.87   -18.93  118.21  1.47
IC N2'  C5'  C6'  N    1.47  113.26    70.08  111.16  1.44
IC C5'  C6'  N    H1'  1.53  111.16    67.33  115.23  1.00
IC H5'  C5'  C6'  N    1.08  109.06  -170.24  111.16  1.44
IC H5'' C5'  C6'  N    1.08  109.06   -49.59  111.16  1.44
IC C6'  C5'  N2'  C3'  1.53  113.26  -108.76  125.61  1.36
IC H6'  C6'  C5'  N2'  1.08  109.44   -49.50  113.26  1.47
!IC H6'' C6'  C5'  N2'  1.08  109.44  -170.33  113.26  1.47
IC N    C6'  C5'  N2'  1.44  111.16    70.00  113.26  1.47
IC H1'  N    C6'  C5'  1.00  115.23    67.33  111.16  1.53
IC C3'  N2'  C2'  H2'' 1.36  115.69   153.43  106.87  1.08
IC O3'  C3'  N2'  C2'  1.23  120.78     6.15  115.69  1.46
IC C4'  C3'  N2'  C2'  1.52  119.02  -173.47  116.54  1.46
IC H4'  C4'  C3'  O3'  1.11  109.30  -113.00  119.96  1.24
IC H4'' C4'  C3'  N2'  1.11  109.30   -52.69  119.02  1.36
IC  C6M  C6'  C5'  N2'  1.52  108.80  -176.38  109.07  1.48
IC H6M   C6M  C6'  C5'  1.08  109.47   -67.99  108.80  1.48
IC H6M'  C6M  C6'  C5'  1.08  109.47   176.01  108.80  1.48
IC H6M'' C6M  C6'  C5'  1.08  109.47    52.01  108.80  1.48
!To connect with previous
IC N    -C  -C2' -H2'' 1.35  118.45   110.49  108.45  1.08
IC C6'  N   -C   -O1'  1.45  122.56     6.77  121.77  1.23
IC C5'  C6'  N   -C    1.52  113.10  -115.86  122.56  1.35
!Adopted from THY, first three lines adjusted to PNA
BILD N2'  C3'  C4'  N1     1.37    115.53 -173.29  119.60   1.48
BILD H4'  C4'  N1   C2     1.11    109.65 -150.45  117.24   1.39
BILD C4'  C2   *N1  C6     1.48    117.24  174.09  121.34   1.3704
BILD C2   N1   C6   C5     1.3746  122.08   -0.02  121.23   1.3432
BILD C6   N1   C2   N3     1.3704  122.08    0.06  115.38   1.3813
BILD N1   N3   *C2  O2     1.3746  115.38 -179.95  121.70   1.2191
BILD N1   C2   N3   C4     1.3746  115.38   -0.07  126.46   1.3795
BILD C5   N3   *C4  O4     1.4439  114.07  179.98  120.59   1.2327
BILD C2   C4   *N3  H3     1.3813  126.46  180.00  116.77   1.0900
BILD C4   C6   *C5  C5M    1.4439  120.78 -179.94  121.63   1.5000
BILD N1   C5   *C6  H6     0.0       0.0   180.0     0.0    0.0
BILD C6   C5   C5M  H51    0.0       0.0     0.0     0.0    0.0
BILD C5   H51  *C5M H52    0.0       0.0   115.0     0.0    0.0
BILD H51  H52  *C5M H53    0.0       0.0  -115.0     0.0    0.0
!PATCHING FIRST NT LAST CT
```

```
RESI UPN            0.00

GROUP                      !
ATOM C       C       0.51 !
ATOM O1'     O      -0.51 !
GROUP                      !
ATOM C2'     CT2    -0.03 !
ATOM H2'     HB2     0.09 !   \   H1'
ATOM H2''    HB2     0.09 !    \ /
ATOM N2'     N      -0.33 !     N   C6M
ATOM C5'     CT2P    0.00 ! mj   \  /
ATOM H5'     HB2     0.09 !       C6'      Base
ATOM H5''    HB2     0.09 !       |        /
GROUP                      !       C5'     C4'
ATOM N       NH1    -0.47 !        \     /
ATOM H1'     H       0.31 !         N2'-C3'
ATOM C6'     CT2     0.07 !        /     \\
ATOM H6'     HB2     0.09 !       C2'     O3'
                           !       |
GROUP                      !       C
ATOM C3'     CC      0.43 !      // \
ATOM O3'     O      -0.54 !     O1'  \
ATOM C4'     CT2    -0.07 !
ATOM H4'     HA2     0.09 !
ATOM H4''    HA2     0.09 !
GROUP                      !
ATOM C6M     CT3    -0.27 !
ATOM H6M     HA3     0.09 !
ATOM H6M'    HA3     0.09 !
ATOM H6M''   HA3     0.09 !
!BASE ATOMS STARTS HERE.
GROUP
ATOM N1      NN2B   -0.34  !
ATOM C6      CN3     0.20  !
ATOM H6      HN3     0.14  !
ATOM C2      CN1T    0.55  !
ATOM O2      ON1    -0.45  !
ATOM N3      NN2U   -0.46  !
ATOM H3      HN2     0.36  !
ATOM C4      CN1     0.53  !
ATOM O4      ON1    -0.48  !
ATOM C5      CN3    -0.15  !
ATOM H5      HN3     0.10  !
BOND +N  C   C  O1'   C  C2'   C2' H2'  C2' H2''  C2' N2'
BOND N2' C5'   C5' H5'   C5' H5''  C6' C5'  C6' H6'   C6' C6M
BOND C6' N     N  H1'    C6M H6M   C6M H6M' C6M H6M''
BOND N2' C3'   C3' O3'   C3' C4'   C4' H4'  C4' H4''
BOND C4'  N1        N1   C2        N1   C6        C2   O2
BOND C2   N3        N3   H3        N3   C4        C4   O4        C4   C5
BOND C5   H5        C5   C6        C6   H6
IMPR C2   N1   N3   O2        C4   N3   C5   O4
!impropers for PNA backbone.
IMPR N    -C   C6'  H1'       C    O1'  C2'  +N
IMPR C3'  O3'  C4'  N2'       N2'  C2'  C5'  C3'
DONO H3   N3
ACCE O2   C2
ACCE O4   C4
DONO H1'  N
ACCE O1'  C
```

```
ACCE O3'  C3'

!PNA backbone
IC O1'  C    C2'  N2'  1.23  119.76   172.34  115.72  1.46
IC C    C2'  N2'  C3'  1.49  115.72   -85.69  115.69  1.36
IC C2'  N2'  C5'  C6'  1.46  116.43    79.13  110.77  1.55
IC H2'  C2'  N2'  C3'  1.08  106.87    35.19  115.69  1.23
IC H2'' C2'  N2'  C5'  1.08  106.87   -18.93  118.21  1.47
IC N2'  C5'  C6'  N    1.47  113.26    70.08  111.16  1.44
IC C5'  C6'  N    H1'  1.53  111.16    67.33  115.23  1.00
IC H5'  C5'  C6'  N    1.08  109.06  -170.24  111.16  1.44
IC H5'' C5'  C6'  N    1.08  109.06   -49.59  111.16  1.44
IC C6'  C5'  N2'  C3'  1.53  113.26  -108.76  125.61  1.36
IC H6'  C6'  C5'  N2'  1.08  109.44   -49.50  113.26  1.47
!IC H6'' C6'  C5'  N2'  1.08  109.44  -170.33  113.26  1.47
IC N    C6'  C5'  N2'  1.44  111.16    70.00  113.26  1.47
IC H1'  N    C6'  C5'  1.00  115.23    67.33  111.16  1.53
IC C3'  N2'  C2'  H2'' 1.36  115.69   153.43  106.87  1.08
IC O3'  C3'  N2'  C2'  1.23  120.78     6.15  115.69  1.46
IC C4'  C3'  N2'  C2'  1.52  119.02  -173.47  116.54  1.46
IC H4'  C4'  C3'  O3'  1.11  109.30  -113.00  119.96  1.24
IC H4'' C4'  C3'  N2'  1.11  109.30   -52.69  119.02  1.36
IC C6M  C6'  C5'  N2'  1.52  108.80  -176.38  109.07  1.48
IC H6M   C6M  C6'  C5'  1.08  109.47   -67.99  108.80  1.48
IC H6M'  C6M  C6'  C5'  1.08  109.47   176.01  108.80  1.48
IC H6M'' C6M  C6'  C5'  1.08  109.47    52.01  108.80  1.48
!To connect with previous
IC N    -C  -C2' -H2'' 1.35  118.45   110.49  108.45  1.08
IC C6'  N   -C   -O1'  1.45  122.56     6.77  121.77  1.23
IC C5'  C6'  N   -C    1.52  113.10  -115.86  122.56  1.35
!Adopted from URA, first three lines adjusted to PNA
BILD N2'  C3'  C4'  N1     1.37    115.53 -173.29  119.60   1.48
BILD H4'  C4'  N1   C2     1.11    109.65 -150.45  117.24   1.39
BILD C4'  C2   *N1  C6     1.48    117.24  174.09  121.34   1.3704
BILD C2   N1   C6   C5     1.379   121.3     0.0   122.8    1.338
BILD C6   N1   C2   N3     1.380   121.3     0.0   114.8    1.373
BILD N1   N3   *C2  O2     1.379   114.8  -180.0   122.0    1.218
BILD N1   C2   N3   C4     1.379   114.8     0.0   127.0    1.383
BILD C5   N3   *C4  O4     1.440   114.7   180.0   119.8    1.227
BILD C2   C4   *N3  H3     1.373   127.0   180.0   116.5    1.03
BILD C6   C4   *C5  H5     0.0       0.0   180.0     0.0    0.0
BILD N1   C5   *C6  H6     0.0       0.0   180.0     0.0    0.0
!PATCHING FIRST NT LAST CT
```

```
RESI APN          0.00
GROUP                    !
ATOM C      C       0.51 !
ATOM O1'    O      -0.51 !
GROUP                    !
ATOM C2'    CT2    -0.03 !
ATOM H2'    HB2     0.09 !    \   H1'
ATOM H2''   HB2     0.09 !     \ /
ATOM N2'    N      -0.33 !      N   C6M
ATOM C5'    CT2P    0.00 ! mj    \  /
ATOM H5'    HB2     0.09 !        C6'       Base
ATOM H5''   HB2     0.09 !        |        /
GROUP                    !        C5'     C4'
ATOM N      NH1    -0.47 !         \     /
ATOM H1'    H       0.31 !          N2'-C3'
ATOM C6'    CT2     0.07 !         /     \\
ATOM H6'    HB1     0.09 !        C2'     O3'
                         !        |
GROUP                    !        C
ATOM C3'    CC      0.43 !       // \
ATOM O3'    O      -0.54 !      O1'  \
ATOM C4'    CT2    -0.07 !
ATOM H4'    HA2     0.09 !
ATOM H4''   HA2     0.09 !
GROUP                    !
ATOM C6M    CT3    -0.27 !
ATOM H6M    HA3     0.09 !
ATOM H6M'   HA3     0.09 !
ATOM H6M''  HA3     0.09 !
!BASE ATOMS STARTS HERE.
GROUP
ATOM N9     NN2    -0.05  !
ATOM C5     CN5     0.28  !
ATOM N7     NN4    -0.71  !
ATOM C8     CN4     0.34  !
ATOM H8     HN3     0.12  !
ATOM N1     NN3A   -0.74  !
ATOM C2     CN4     0.50  !
ATOM H2     HN3     0.13  !
ATOM N3     NN3A   -0.75
ATOM C4     CN5     0.43
ATOM C6     CN2     0.46
ATOM N6     NN1    -0.77
ATOM H61    HN1     0.38
ATOM H62    HN1     0.38
BOND +N   C     C   O1'  C   C2'   C2' H2'  C2' H2''  C2' N2'
BOND N2'  C5'   C5' H5'  C5' H5''  C6' C5'  C6' H6'   C6' C6M
BOND C6'  N     N   H1'  C6M H6M   C6M H6M' C6M H6M''
BOND N2'  C3'   C3' O3'  C3' C4'   C4' H4'  C4' H4''
BOND C4'  N9        N9   C4        N9   C8        C4   N3
BOND C4   C5        N3   C2        C2   N1        N1   C6        C6   N6
BOND N6   H61       N6   H62       C6   C5        C5   N7        N7   C8
BOND C8   H8        C2   H2
IMPR N6   C6   H61  H62       C6   N1   C5   N6
!impropers for PNA backbone.
IMPR N    -C   C6'  H1'      C    O1'  C2'  +N
IMPR C3'  O3'  C4'  N2'      N2'  C2'  C5'  C3'
DONO H61  N6
```

```
DONO H62  N6
ACCE N3
ACCE N7
ACCE N1
DONO H1'  N
ACCE O1'  C
ACCE O3'  C3'

!PNA backbone
IC O1'  C    C2'  N2'  1.23  119.76   172.34  115.72  1.46
IC C    C2'  N2'  C3'  1.49  115.72   -85.69  115.69  1.36
IC C2'  N2'  C5'  C6'  1.46  116.43    79.13  110.77  1.55
IC H2'  C2'  N2'  C3'  1.08  106.87    35.19  115.69  1.23
IC H2'' C2'  N2'  C5'  1.08  106.87   -18.93  118.21  1.47
IC N2'  C5'  C6'  N    1.47  113.26    70.08  111.16  1.44
IC C5'  C6'  N    H1'  1.53  111.16    67.33  115.23  1.00
IC H5'  C5'  C6'  N    1.08  109.06  -170.24  111.16  1.44
IC H5'' C5'  C6'  N    1.08  109.06   -49.59  111.16  1.44
IC C6'  C5'  N2'  C3'  1.53  113.26  -108.76  125.61  1.36
IC H6'  C6'  C5'  N2'  1.08  109.44   -49.50  113.26  1.47
!IC H6'' C6'  C5'  N2'  1.08  109.44  -170.33  113.26  1.47
IC N    C6'  C5'  N2'  1.44  111.16    70.00  113.26  1.47
IC H1'  N    C6'  C5'  1.00  115.23    67.33  111.16  1.53
IC C3'  N2'  C2'  H2'' 1.36  115.69   153.43  106.87  1.08
IC O3'  C3'  N2'  C2'  1.23  120.78     6.15  115.69  1.46
IC C4'  C3'  N2'  C2'  1.52  119.02  -173.47  116.54  1.46
IC H4'  C4'  C3'  O3'  1.11  109.30  -113.00  119.96  1.24
IC H4'' C4'  C3'  N2'  1.11  109.30   -52.69  119.02  1.36
IC  C6M  C6'  C5'  N2'  1.52  108.80  -176.38  109.07  1.48
IC H6M   C6M  C6'  C5'  1.08  109.47   -67.99  108.80  1.48
IC H6M'  C6M  C6'  C5'  1.08  109.47   176.01  108.80  1.48
IC H6M'' C6M  C6'  C5'  1.08  109.47    52.01  108.80  1.48
!To connect with previous
IC N    -C  -C2' -H2'' 1.35  118.45   110.49  108.45  1.08
IC C6'  N   -C   -O1'  1.45  122.56     6.77  121.77  1.23
IC C5'  C6'  N   -C    1.52  113.10  -115.86  122.56  1.35
!Adopted from ADE, first three lines adjusted to PNA
BILD O3'  C3'  C4'  N9     1.23    120.40   10.91  109.15   1.46
BILD H4'  C4'  N9   C4     1.11    109.86 -150.27  124.65   1.36
BILD C4'  C4   *N9  C8     1.46    124.65  177.35  107.10   1.374
BILD C4   N9   C8   N7     1.376   106.0     0.0   113.6    1.312
BILD C8   N9   C4   C5     1.367   106.0     0.0   105.6    1.382
BILD C8   N7   C5   C6     0.0       0.0   180.0     0.0    0.0
BILD N7   C5   C6   N1     0.0       0.0   180.0     0.0    0.0
BILD C5   C6   N1   C2     0.0       0.0     0.0     0.0    0.0
BILD N9   C5   *C4  N3     1.376   105.6  -180.0   126.9    1.342
BILD C5   N1   *C6  N6     1.409   117.6  -180.0   121.2    1.337
BILD N1   C6   N6   H61    1.337   121.2     0.0   119.0    1.01
BILD H61  C6   *N6  H62    1.01    119.0   180.0   119.00   1.01
BILD C5   N1   *C6  N6     1.409   117.6  -180.0   119.0    1.337
BILD N1   C6   N6   H61    1.337   119.0     0.0   119.0    1.01
BILD H61  C6   *N6  H62    1.01    119.0   180.0   121.00   1.01
BILD N9   N7   *C8   H8    0.0       0.0   180.0     0.0    0.0
BILD N1   N3   *C2   H2    0.0       0.0   180.0     0.0    0.0
!PATCHING FIRST NT LAST CT
```

## Topology and Parameters for the left-handed miniPEG-modified γPNA nucleobases. Current Nomenclature remained the same from the most recent force fieldimprovements.[1]

**Table S3.** Estimated parameters for the miniPEG-modified γPNA.

| Bonds | | | |
|---|---|---|---|
| Atom Types | | $k_b$ (kcal/mol) | $b_0$ (Å) |
| CT2 | CG321 | 225 | 1.53 |
| CG321 | HB2 | 330 | 1.08 |
| NH1 | CG311 | 320 | 1.43 |
| CG311 | CT2P | 225 | 1.53 |
| CG311 | HB2 | 330 | 1.08 |
| CG321 | HCA2A | 309 | 1.111 |
| CG321 | OC30A | 225 | 1.53 |
| CC32A | OG311 | 360 | 1.415 |

| Angles | | | | | | |
|---|---|---|---|---|---|---|
| Atom Types | | | $k_\theta$ (kcal/mol) | $\theta_0$ (º) | $k_{UB}$ (kcal/mol) | $s_0$ (Å) |
| HB2 | CG321 | HCA2A | 35.5 | 109.5 | 5.4 | 1.802 |
| CT2 | CG321 | HCA2A | 26.5 | 110.1 | 22.53 | 2.179 |
| CG311 | NH1 | C | 50 | 120 | | |
| CG311 | NH1 | H | 35 | 117 | | |
| NH1 | CG311 | CT2P | 70 | 113.5 | | |
| NH1 | CG311 | HB2 | 48 | 108 | | |
| NH1 | CG311 | CG321 | 70 | 117 | | |
| CT2P | CG311 | HB2 | 50 | 109.5 | | |
| CT2P | CG311 | CG321 | 58.35 | 113.6 | | |
| HB2 | CG311 | CG321 | 50 | 109.5 | | |
| CG311 | CT2P | N | 70 | 113.5 | | |
| CG311 | CT2P | HB2 | 50 | 109.5 | | |
| CT2P | N | CT2P | 35 | 117 | | |
| CG311 | CG321 | HCA2A | 26.5 | 110.1 | | |
| HCA2A | CG321 | HCA2A | 35.5 | 109 | | |
| HCA2A | CG321 | OC30A | 60 | 109.5 | | |
| CG321 | OC30A | CC32A | 95 | 109.5 | | |
| C | CT2 | N | 43.7 | 110 | | |
| CG311 | CG321 | OC30A | 75.7 | 110.1 | | |
| CC | N | CT2 | 50 | 120 | | |
| CG321 | OC30A | HCA2A | 60 | 109.5 | | |
| CC32A | CC32A | OC30A | 45 | 111.5 | | |
| HCA2A | CC32A | OG311 | 60 | 109.5 | | |
| CC32A | OG311 | HGP1 | 50 | 106 | | |

| | | | | |
|---|---|---|---|---|
| CC32A | OC30A | HCA2A | 60 | 109.5 |
| CG321 | OC30A | CC32A | 95 | 109.7 |
| HCA2A | OC30A | CC32A | 60 | 109.5 |
| CC32A | CC32A | OG311 | 45 | 109 |
| CC32A | OG311 | HGP1 | 50 | 106 |
| CG321 | OC30A | HCA2A | 60 | 109.5 |
| CG321 | OC30A | CC32A | 95 | 109.7 |
| NH3 | CT2 | CT2P | 67.7 | 110 |
| NH3 | CT2 | CG321 | 67.7 | 110 |
| HB2 | CT2 | CG321 | 50 | 109.5 |
| CT2P | CT2 | CG321 | 58.35 | 113.6 |
| CT2 | CG321 | HB2 | 50 | 109.5 |
| CT2 | CG321 | OC30A | 75.5 | 110 |
| HB2 | CG321 | OC30A | 60 | 109.5 |

| **Dihedrals** | | | | | | |
|---|---|---|---|---|---|---|
| Atom Types | | | | $k_\chi$ (kcal/mol) | $n$ | $\delta$ (º) |
| NH3 | CT2 | CG321 | HB2 | 0.19 | 3 | 0 |
| NH3 | CT2 | CG321 | HCA2A | 0.19 | 3 | 0 |
| NH3 | CT2 | CG321 | OC30A | 0.19 | 3 | 0 |
| CT2 | CG321 | OC30A | CC32A | 0.57 | 1 | 0 |
| CT2 | CG321 | OC30A | CC32A | 0.29 | 2 | 0 |
| CT2 | CG321 | OC30A | CC32A | 0.43 | 3 | 0 |
| HB2 | CT2 | CG321 | HB2 | 0.2 | 3 | 0 |
| HB2 | CT2 | CG321 | HCA2A | 0.2 | 3 | 0 |
| HB2 | CT2 | CG321 | OC30A | 0.19 | 3 | 0 |
| HB2 | CG321 | CT2 | CT2P | 0.19 | 3 | 0 |
| HB2 | CG321 | OC30A | CC32A | 0.284 | 3 | 0 |
| CT2P | CT2 | CG321 | HCA2A | 0.19 | 3 | 0 |
| CT2P | CT2 | CG321 | OC30A | 0.2 | 3 | 0 |
| CG321 | OC30A | CC32A | HCA2A | 0.284 | 3 | 0 |
| CG321 | OC30A | CC32A | CC32A | 0.57 | 1 | 0 |
| CG321 | OC30A | CC32A | CC32A | 0.29 | 2 | 0 |
| CG321 | OC30A | CC32A | CC32A | 0.43 | 3 | 0 |
| HCA2A | CC32A | OG311 | HGP1 | 0.18 | 3 | 0 |
| NH3 | CT2 | CT2P | N | 0.4 | 1 | 0 |
| NH3 | CT2 | CT2P | HB2 | 0.15 | 3 | 0 |
| N | CT2P | CT2 | CG321 | 0.19 | 3 | 0 |
| HB2 | CT2P | CT2 | CG321 | 0.19 | 3 | 0 |
| HCA2A | CG321 | OC30A | CC32A | 0.284 | 3 | 0 |
| OC30A | CC32A | CC32A | OG311 | 0.59 | 1 | 180 |

| | | | | | | |
|---|---|---|---|---|---|---|
| OC30A | CC32A | CC32A | OG311 | 1.16 | 2 | 0 |
| HCA2A | CC32A | CC32A | OG311 | 0.19 | 3 | 0 |
| CT2 | N | CT2P | CG311 | 0.73 | 1 | 0 |
| CT2 | N | CT2P | CG311 | 1.52 | 3 | 0 |
| CT2 | N | CT2P | CG311 | 0.48 | 4 | 0 |
| CT2 | N | CT2P | CG311 | 0.31 | 5 | 0 |
| NH1 | C | CT2 | N | 0.68 | 1 | 0 |
| NH1 | C | CT2 | N | 0.71 | 2 | 0 |
| NH1 | C | CT2 | N | 0.33 | 3 | 0 |
| O | C | CT2 | N | 1 | 2 | 180 |
| O | C | CT2 | N | 0.2 | 3 | 0 |
| CT2 | CC | N | CT2 | 2.6 | 2 | 180 |
| CT2 | CC | N | CT2P | 2.6 | 2 | 180 |
| CG321 | CG311 | CT2P | N | 0.35 | 3 | 0 |
| CT2P | CG311 | CG321 | OC30A | 0.2 | 3 | 180 |
| NH1 | CG311 | CT2P | N | 1.59 | 1 | 1800 |
| NH1 | CG311 | CT2P | N | 0.72 | 3 | 0 |
| NH1 | CG311 | CT2P | N | 0.03 | 4 | 0 |
| NH1 | CG311 | CG321 | OC30A | 2.4 | 1 | 180 |
| HB2 | CG311 | CG321 | OC30A | 0.195 | 3 | 0 |
| C | CT2 | N | CC | 0.3 | 2 | 0 |
| C | CT2 | N | CC | 0.5 | 3 | 180 |
| C | CT2 | N | CT2P | 0.62 | 4 | 180 |
| CG311 | CT2P | N | CC | 0.6 | 1 | 0 |
| CG311 | CT2P | N | CC | 0.45 | 2 | 0 |
| HB2 | CT2 | N | CC | 0.1 | 3 | 180 |
| HB2 | CT2P | N | CC | 0.1 | 3 | 180 |
| CG311 | CG321 | OC30A | CC32A | 0.57 | 1 | 0 |
| CG311 | CG321 | OC30A | CC32A | 0.29 | 2 | 0 |
| CG311 | CG321 | OC30A | CC32A | 0.43 | 3 | 0 |
| NH1 | CG311 | CT2P | HB2 | 0.15 | 3 | 0 |
| !NH1 | CG311 | CT2P | HB2 | 0.15 | 3 | 0 |
| CG311 | NH1 | C | CT2 | 1.6 | 1 | 0 |
| CG311 | NH1 | C | CT2 | 2.5 | 2 | 180 |
| CG311 | NH1 | C | O | 2.5 | 2 | 180 |
| CG311 | CT2P | N | CT2P | 0.73 | 1 | 0 |
| CG311 | CT2P | N | CT2P | 1.52 | 3 | 0 |
| CG311 | CT2P | N | CT2P | 0.48 | 4 | 0 |
| CG311 | CT2P | N | CT2P | 0.31 | 5 | 0 |
| CT2 | CG311 | NH1 | C | 1.8 | 1 | 0 |

| | | | | | | |
|---|---|---|---|---|---|---|
| CT2 | CG311 | NH1 | H | 0 | 1 | 0 |
| CT2P | CG311 | CG321 | HCA2A | 0.2 | 3 | 0 |
| CT2P | N | CT2P | HB2 | 0.1 | 3 | 180 |
| N | CT2P | CG311 | HB2 | 0.15 | 3 | 0 |
| C | NH1 | CG311 | HB2 | 0 | 1 | 0 |
| C | NH1 | CG311 | CG321 | 1.8 | 1 | 0 |
| H | NH1 | CG311 | HB2 | 0 | 1 | 0 |
| H | NH1 | CG311 | CG321 | 0 | 1 | 0 |
| HB2 | CG311 | CT2P | HB2 | 0.1 | 3 | 0 |
| HB2 | CG311 | CT2P | HCA2A | 0.1 | 3 | 0 |
| NH1 | CG311 | CG321 | HCA2A | 0.2 | 3 | 0 |
| CT2P | CG311 | NH1 | C | 1.8 | 1 | 0 |
| CT2P | CG311 | NH1 | H | 0 | 1 | 0 |
| C | NH1 | CG311 | CG321 | 0.2 | 3 | 0 |
| HB2 | CG311 | CG321 | HCA2A | 0.2 | 3 | 0 |
| CG321 | OC30A | CC33A | HCA3A | 0.284 | 3 | 0 |
| HCA2A | CG321 | OC30A | CC33A | 0.284 | 3 | 0 |
| HB2 | CT2P | CG311 | CG321 | 0.2 | 3 | 0 |
| CC32A | CC32A | OG311 | HGP1 | 0.24 | 3 | 0 |
| C | NH1 | CT2 | CT3 | 0.96 | 2 | 0 |
| C | NH1 | CT2 | CT3 | 0.17 | 3 | 0 |
| C | NH1 | CT2 | CT3 | 0.41 | 4 | 0 |
| C | NH1 | CT2 | CT3 | 0.27 | 5 | 0 |
| N | CT2P | CT2 | CT3 | 1.59 | 1 | 180 |
| N | CT2P | CT2 | CT3 | 0.72 | 3 | 0 |
| N | CT2P | CT2 | CT3 | 0.03 | 4 | 0 |
| HB2 | CT2P | CT2 | CT3 | 0.19 | 3 | 0 |

```
RESI GGPN          0.00 !

GROUP                   !
ATOM C      C       0.51 !
ATOM O1'    O      -0.51 !
GROUP                   !
ATOM C2'    CT2    -0.03 !
ATOM H2'    HB2     0.09 !   \   H1'
ATOM H2''   HB2     0.09 !    \ /
ATOM N2'    N      -0.36 !     N   C6''-O7'-C8'-C9'-O10'-C11'-C12'-O13'
ATOM C5'    CT2P    0.01 ! mj   \ /
ATOM H5'    HB2     0.09 !       C6'      Base
ATOM H5''   HB2     0.09 !       |        /
GROUP                   !       C5'     C4'
ATOM N      NH1    -0.47 !        \     /
ATOM H1'    H       0.31 !         N2'-C3'
GROUP ! MINIPEG STARTS HERE        /
ATOM C6'    CG311   0.07 !       C2'
ATOM H6'    HB2     0.09 !       |
ATOM C6''   CG321   0.08 !       C
ATOM H6''   HCA2A   0.09 !     // \
ATOM H6'''  HCA2A   0.09 !    O1'  \
ATOM O7'    OC30A  -0.34 !
ATOM C8'    CC32A  -0.01 !
ATOM H8'    HCA2A   0.09 !
ATOM H8''   HCA2A   0.09 !
ATOM C9'    CC32A  -0.01 !
ATOM H9'    HCA2A   0.09 !
ATOM H9''   HCA2A   0.09 !
ATOM O10'   OC30A  -0.34 !
ATOM C11'   CC32A  -0.01 !
ATOM H11'   HCA2A   0.09 !
ATOM H11''  HCA2A   0.09 !
ATOM C12'   CC32A  -0.01 !
ATOM H12'   HCA2A   0.09 !
ATOM H12''  HCA2A   0.09 !
ATOM O13'   OG311  -0.67 !
ATOM H13'   HGP1    0.43 !
! MINIPEG ENDS HERE!
GROUP                   !
ATOM C3'    CC      0.43 !
ATOM O3'    O      -0.57 !
ATOM C4'    CT2    -0.07 !
ATOM H4'    HA2     0.09 !
ATOM H4''   HA2     0.09 !
!BASE ATOMS STARTS HERE. !
GROUP                   !
ATOM N9     NN2B   -0.02 !
ATOM C4     CN5     0.26 !
ATOM N2     NN1    -0.68 !
ATOM H21    HN1     0.32 !
ATOM H22    HN1     0.35 !
ATOM N3     NN3G   -0.74 !
ATOM C2     CN2     0.75
ATOM N1     NN2G   -0.34
ATOM H1     HN2     0.26
ATOM C6     CN1     0.54
ATOM O6     ON1    -0.51
ATOM C5     CN5G    0.00
```

```
ATOM N7     NN4    -0.60
ATOM C8     CN4     0.25
ATOM H8     HN3     0.16
BOND +N   C     C   O1'   C   C2'   C2' H2'  C2' H2''  C2' N2'
BOND N2'  C5'   C5' H5'   C5' H5''  C6' C5'  C6' H6'
BOND C6'  N     N   H1'
! GLYCOL STARTS HERE
BOND C6' C6''  C6'' O7'  C6'' H6''  C6'' H6'''  O7' C8'  C8' H8'
BOND C8' H8''  C8' C9'  C9' O10'  C9' H9'  C9' H9''  O10' C11'
BOND C11' H11'  C11' H11'' C11' C12'  C12' H12'  C12'  H12''
BOND C12' O13'  O13' H13'
! GLYCOL ENDS HERE
BOND N2'  C3'   C3' O3'   C3' C4'   C4' H4'  C4' H4''
BOND C4'  N9        N9   C4        N9   C8        C4   N3
BOND C4   C5        N3   C2        C2   N2        C2   N1        N2   H21
BOND N2   H22       N1   H1        N1   C6        C6   O6        C6   C5
BOND C5   N7        N7   C8        C8   H8
IMPR C2   N3   N1   N2       C6   N1   C5   O6        N2   H21  C2   H22
!impropers for PNA backbone.
IMPR N    -C   C6'  H1'       C    O1'  C2'  +N
IMPR C3'  O3'  C4'  N2'       N2'  C2'  C5'  C3'
DONO H21  N2
DONO H22  N2
DONO H1   N1
DONO H1'  N1
ACCE O6   C6
ACCE N3
ACCE N7
ACCE O1'  C
ACCE O3'  C3'
ACCE O7'
ACCE O10'
DONO H13' O13'

!PNA backbone
IC O1'  C    C2'  N2'  1.23  119.76   -172.34  115.72  1.46
IC C    C2'  N2'  C3'  1.49  115.72   85.69  115.69  1.36
IC C2'  N2'  C5'  C6'  1.46  116.43   -79.13  110.77  1.55
IC H2'  C2'  N2'  C3'  1.08  106.87   -35.19  115.69  1.23
IC H2'' C2'  N2'  C5'  1.08  106.87   18.93  118.21  1.47
IC N2'  C5'  C6'  N    1.47  113.26   -70.08  111.16  1.44
IC C5'  C6'  N    H1'  1.53  111.16   -67.33  115.23  1.00
IC H5'  C5'  C6'  N    1.08  109.06  170.24  111.16  1.44
IC H5'' C5'  C6'  N    1.08  109.06   49.59  111.16  1.44
IC C6'  C5'  N2'  C3'  1.53  113.26  108.76  125.61  1.36
IC H6'  C6'  C5'  N2'  1.08  109.44  170.50  113.26  1.47 ! DELTE THIS FOR RIGHT
HANDED HELIX
IC N    C6'  C5'  N2'  1.44  111.16   -70.00  113.26  1.47
IC H1'  N    C6'  C5'  1.00  115.23    -67.33  111.16  1.53
IC C3'  N2'  C2'  H2'' 1.36  115.69   -153.43  106.87  1.08
IC O3'  C3'  N2'  C2'  1.23  120.78     -6.15  115.69  1.46
IC C4'  C3'  N2'  C2'  1.52  119.02  173.47  116.54  1.46
IC H4'  C4'  C3'  O3'  1.11  109.30  113.00  119.96  1.24
IC H4'' C4'  C3'  N2'  1.11  109.30   52.69  119.02  1.36
! Glycol Linkage
IC C6'' C6'  C5'  N2'  1.52  109.29   60.19  112.08  1.46
IC H6'' C6'' C6'  C5'  1.10  109.32   66.67  109.29  1.54
IC H6''' C6'' C6' C5'  1.10  109.32   173.53  109.29  1.54
```

```
IC O7'  C6'' C6'  C5'  1.42  106.39   180.0  106.29  1.54
IC C8'  O7'  C6'' C6'  1.42  111.39   180.0  106.29  1.54
IC H8'  C8'  O7'  C6'' 1.10  109.32   -66.67  109.29  1.42
IC H8'' C8'  O7'  C6'' 1.10  109.32    66.67  109.29  1.42
IC C9'  C8'  O7'  C6'' 1.54  109.5    180.0   111.40  1.42
IC H9'  C9'  C8'  O7'  1.10  109.50   -66.67  106.65  1.42
IC H9'' C9'  C8'  O7'  1.10  109.50    66.67  106.65  1.42
IC O10' C9'  C8'  O7'  1.42  109.50   180.00  109.50  1.42
IC C11' O10' C9'  C8'  1.42  109.50   180.00  109.50  1.54
IC H11' C11' O10' C9'  1.10  109.50   -66.67  109.50  1.42
IC H11'' C11' O10' C9' 1.10  109.50    66.67  109.50  1.42
IC C12' C11' O10' C9'  1.54  109.50   180.00  109.50  1.42
IC H12' C12' C11' O10' 1.10  109.50   -66.67  109.50  1.42
IC H12'' C12' C11' O10' 1.10 109.50    66.67  109.50  1.42
IC O13' C12' C11' O10' 1.42  109.50   180.00  109.50  1.42
IC H13' O13' C12' C11' 0.96  109.5    180.00  109.50  1.54
!To connect with previous
IC N    -C  -C2' -H2'' 1.35  118.45  -110.49  108.45  1.08
IC C6'  N   -C   -O1'  1.45  122.56    -6.77  121.77  1.23
IC C5'  C6'  N   -C    1.52  113.10   115.86  122.56  1.35
!Adopted from GUA, first three lines adjusted to PNA
BILD O3'  C3'  C4'  N9     1.23    120.40   -10.91  109.15   1.46
BILD H4'  C4'  N9   C4     1.11    109.86 150.27  124.65   1.36
BILD C4'  C4   *N9  C8     1.46    124.65  -177.35  107.10   1.374
BILD C4   N9   C8   N7     1.377   106.0     0.0   113.5    1.304
BILD C8   N9   C4   C5     1.374   106.0     0.0   105.6    1.377
BILD N9   C5   *C4  N3     1.377   105.6   180.0   128.4    1.355
BILD C5   C4   N3   C2     1.377   128.4     0.0   111.8    1.327
BILD C4   N3   C2   N1     1.355   111.8     0.0   124.0    1.375
BILD N1   N3   *C2  N2     1.375   124.0   180.0   119.7    1.341
BILD N3   C2   N2   H21    1.327   119.7   180.0   127.0    1.01
BILD H21  C2   *N2  H22    1.01    127.0  -180.0   116.5    1.01
BILD N3   C2   N1   C6     1.327   124.0     0.0   124.9    1.393
BILD C6   C2   *N1  H1     1.393   124.9   180.0   117.4    1.03
BILD C5   N1   *C6  O6     1.415   111.7   180.0   120.0    1.239
BILD N9   N7   *C8  H8     0.0       0.0   180.0     0.0    0.0
!PATCHING FIRST NT LAST CT
```

```
RESI GCPN          0.00
GROUP                   !
ATOM C      C       0.51 !
ATOM O1'    O      -0.51 !
GROUP                    !
ATOM C2'    CT2    -0.03 !
ATOM H2'    HB2     0.09 !   \   H1'
ATOM H2''   HB2     0.09 !    \ /
ATOM N2'    N      -0.36 !     N   C6''-O7'-C8'-C9'-O10'-C11'-C12'-O13'
ATOM C5'    CT2P    0.01 ! mj   \ /
ATOM H5'    HB2     0.09 !       C6'      Base
ATOM H5''   HB2     0.09 !       |        /
GROUP                    !       C5'     C4'
ATOM N      NH1    -0.47 !        \     /
ATOM H1'    H       0.31 !         N2'-C3'
GROUP ! MINIPEG STARTS HERE       / AT
ATOM C6'    CG311   0.07 !       C2'
ATOM H6'    HB2     0.09 !       |
ATOM C6''   CG321   0.08 !       C
ATOM H6''   HCA2A   0.09 !     // \
ATOM H6'''  HCA2A   0.09 !    O1'  \
ATOM O7'    OC30A  -0.34 !
ATOM C8'    CC32A  -0.01 !
ATOM H8'    HCA2A   0.09 !
ATOM H8''   HCA2A   0.09 !
ATOM C9'    CC32A  -0.01 !
ATOM H9'    HCA2A   0.09 !
ATOM H9''   HCA2A   0.09 !
ATOM O10'   OC30A  -0.34 !
ATOM C11'   CC32A  -0.01 !
ATOM H11'   HCA2A   0.09 !
ATOM H11''  HCA2A   0.09 !
ATOM C12'   CC32A  -0.01 !
ATOM H12'   HCA2A   0.09 !
ATOM H12''  HCA2A   0.09 !
ATOM O13'   OG311  -0.67 !
ATOM H13'   HGP1    0.43 !
! MINIPEG ENDS HERE
GROUP                    !
ATOM C3'    CC      0.43 !
ATOM O3'    O      -0.54 !
ATOM C4'    CT2    -0.07 !
ATOM H4'    HA2     0.09 !
ATOM H4''   HA2     0.09 !
!BASE ATOMS STARTS HERE.
GROUP
ATOM N1     NN2    -0.13
ATOM C6     CN3     0.05
ATOM H6     HN3     0.17
ATOM C5     CN3    -0.13
ATOM H5     HN3     0.07
ATOM C2     CN1     0.52
ATOM O2     ON1C   -0.49
ATOM N3     NN3    -0.66
ATOM C4     CN2     0.65
ATOM N4     NN1    -0.75
ATOM H41    HN1     0.37
ATOM H42    HN1     0.33
```

```
BOND +N   C      C   O1'   C   C2'   C2' H2'  C2' H2''  C2' N2'
BOND N2'  C5'    C5' H5'   C5' H5''  C6' C5'  C6' H6'
BOND C6'  N      N   H1'
BOND N2'  C3'    C3' O3'   C3' C4'   C4' H4'  C4' H4''
! GLYCOL STARTS HERE
BOND C6' C6''  C6'' O7'  C6'' H6''  C6'' H6'''  O7' C8'  C8'  H8'
BOND C8' H8''  C8' C9'  C9' O10'  C9' H9'  C9' H9''  O10' C11'
BOND C11' H11'  C11' H11'' C11' C12'  C12' H12'  C12'  H12''
BOND C12' O13'  O13' H13'
! GLYCOL ENDS HERE
BOND C4'  N1    N1   C2         N1   C6         C2   O2
BOND C2   N3    N3   C4         C4   N4         N4   H41        N4   H42
BOND C4   C5    C5   C6         C5   H5         C6   H6
IMPR C2   N1   N3   O2         C4   N3   C5   N4
!impropers for PNA backbone.
IMPR N    -C   C6'  H1'        C    O1'  C2'  +N
IMPR C3'  O3'  C4'  N2'        N2'  C2'  C5'  C3'
DONO H42  N4
DONO H41  N4
ACCE O2
ACCE N3
DONO H1'  N
ACCE O1'  C
ACCE O3'  C3'
ACCE O7'
ACCE O10'
DONO H13' O13'

!PNA backbone
IC O1'  C    C2'  N2'  1.23  119.76   -172.34  115.72  1.46
IC C    C2'  N2'  C3'  1.49  115.72   85.69  115.69  1.36
IC C2'  N2'  C5'  C6'  1.46  116.43   -79.13  110.77  1.55
IC H2'  C2'  N2'  C3'  1.08  106.87   -35.19  115.69  1.23
IC H2'' C2'  N2'  C5'  1.08  106.87   18.93  118.21  1.47
IC N2'  C5'  C6'  N    1.47  113.26   -70.08  111.16  1.44
IC C5'  C6'  N    H1'  1.53  111.16   -67.33  115.23  1.00
IC H5'  C5'  C6'  N    1.08  109.06  170.24  111.16  1.44
IC H5'' C5'  C6'  N    1.08  109.06   49.59  111.16  1.44
IC C6'  C5'  N2'  C3'  1.53  113.26  108.76  125.61  1.36
IC H6'  C6'  C5'  N2'  1.08  109.44  170.50  113.26  1.47
IC N    C6'  C5'  N2'  1.44  111.16   -70.00  113.26  1.47
IC H1'  N    C6'  C5'  1.00  115.23    -67.33  111.16  1.53
IC C3'  N2'  C2'  H2'' 1.36  115.69   -153.43  106.87  1.08
IC O3'  C3'  N2'  C2'  1.23  120.78     -6.15  115.69  1.46
IC C4'  C3'  N2'  C2'  1.52  119.02  173.47  116.54  1.46
IC H4'  C4'  C3'  O3'  1.11  109.30  113.00  119.96  1.24
IC H4'' C4'  C3'  N2'  1.11  109.30   52.69  119.02  1.36
! Glycol linkage
IC C6'' C6'  C5'  N2'  1.52  109.29   60.19  112.08  1.46
IC H6'' C6'' C6'  C5'  1.10  109.32   66.67  109.29  1.54
IC H6''' C6'' C6' C5'  1.10  109.32   173.53  109.29  1.54
IC O7'  C6'' C6'  C5'  1.42  106.39   180.0  106.29  1.54
IC C8'  O7'  C6'' C6'  1.42  111.39   180.0  106.29  1.54
IC H8'  C8'  O7'  C6'' 1.10  109.32   -66.67  109.29  1.42
IC H8'' C8'  O7'  C6'' 1.10  109.32    66.67  109.29  1.42
IC C9'  C8'  O7'  C6'' 1.54  109.5    180.0   111.40  1.42
IC H9'  C9'  C8'  O7'  1.10  109.50   -66.67  106.65  1.42
IC H9'' C9'  C8'  O7'  1.10  109.50    66.67  106.65  1.42
```

```
IC O10' C9'  C8'  O7'  1.42  109.50   180.00  109.50  1.42
IC C11' O10' C9'  C8'  1.42  109.50   180.00  109.50  1.54
IC H11' C11' O10' C9'  1.10  109.50   -66.67  109.50  1.42
IC H11'' C11' O10' C9' 1.10  109.50    66.67  109.50  1.42
IC C12' C11' O10' C9'  1.54  109.50   180.00  109.50  1.42
IC H12' C12' C11' O10' 1.10  109.50   -66.67  109.50  1.42
IC H12'' C12' C11' O10' 1.10 109.50    66.67  109.50  1.42
IC O13' C12' C11' O10' 1.42  109.50   180.00  109.50  1.42
IC H13' O13' C12' C11' 0.96  109.5    180.00  109.50  1.54
! GLYCOL ENDS HERE
!To connect with previous
IC N    -C  -C2' -H2'' 1.35  118.45   -110.49  108.45  1.08
IC C6'  N   -C   -O1'  1.45  122.56    -6.77  121.77  1.23
IC C5'  C6'  N   -C    1.52  113.10   115.86  122.56  1.35
!Adopted from CYT, first three lines adjusted to PNA
BILD N2'  C3'  C4'  N1     1.36    118.87   175.00  110.85   1.47
BILD H4'  C4'  N1   C2     1.11    109.43   158.84  117.57   1.40
BILD C4'  C2   *N1  C6     1.47    117.57  -173.53  120.6    1.364
BILD C2   N1   C6   C5     1.399   120.6     0.0   121.0    1.337
BILD C6   N1   C2   N3     1.364   120.6     0.0   118.9    1.356
BILD N1   N3   *C2  O2     1.399   118.9   180.0   121.9    1.237
BILD N1   C2   N3   C4     1.399   118.9     0.0   120.0    1.334
BILD C5   N3   *C4  N4     1.426   121.8   180.00  118.9    1.337
BILD N3   C4   N4   H41    1.337   117.9     0.00  118.9    1.01
BILD H41  C4   *N4  H42    1.01    118.9   180.00  120.7    1.01
BILD C6   C4   *C5  H5     0.0       0.0   180.0     0.0    0.0
BILD N1   C5   *C6  H6     0.0       0.0   180.0     0.0    0.0
!PATCHING FIRST NT LAST CT
```

```
RESI GTPN            0.00 !

GROUP                     !
ATOM C      C        0.51 !
ATOM O1'    O       -0.51 !
GROUP                     !
ATOM C2'    CT2     -0.03 !
ATOM H2'    HB2      0.09 !   \   H1'
ATOM H2''   HB2      0.09 !    \ /
ATOM N2'    N       -0.36 !     N   C6''-O7'-C8'-C9'-O10'-C11'-C12'-O13'     N
ATOM C5'    CT2P     0.01 ! mj   \ /
ATOM H5'    HB2      0.09 !       C6'      Base
ATOM H5''   HB2      0.09 !       |        /
GROUP                     !       C5'     C4'
ATOM N      NH1     -0.47 !        \     /
ATOM H1'    H        0.31 !         N2'-C3'
GROUP ! MINIPEG STARTS HERE        /
ATOM C6'    CG311    0.07 !       C2'
ATOM H6'    HB2      0.09 !       |
ATOM C6''   CG321    0.08 !       C
ATOM H6''   HCA2A    0.09 !     // \
ATOM H6'''  HCA2A    0.09 !    O1'  \
ATOM O7'    OC30A   -0.34 !
ATOM C8'    CC32A   -0.01 !
ATOM H8'    HCA2A    0.09 !
ATOM H8''   HCA2A    0.09 !
ATOM C9'    CC32A   -0.01 !
ATOM H9'    HCA2A    0.09 !
ATOM H9''   HCA2A    0.09 !
ATOM O10'   OC30A   -0.34 !
ATOM C11'   CC32A   -0.01 !
ATOM H11'   HCA2A    0.09 !
ATOM H11''  HCA2A    0.09 !
ATOM C12'   CC32A   -0.01 !
ATOM H12'   HCA2A    0.09 !
ATOM H12''  HCA2A    0.09 !
ATOM O13'   OG311   -0.67 !
ATOM H13'   HGP1     0.43 !
! MINIPEG ENDS HERE
GROUP                     !
ATOM C3'    CC       0.43 !
ATOM O3'    O       -0.54 !
ATOM C4'    CT2     -0.07 !
ATOM H4'    HA2      0.09 !
ATOM H4''   HA2      0.09 !
!BASE ATOMS STARTS HERE.
GROUP
ATOM N1     NN2B    -0.34
ATOM C6     CN3      0.17
ATOM H6     HN3      0.17
ATOM C2     CN1T     0.51
ATOM O2     ON1     -0.41
ATOM N3     NN2U    -0.46
ATOM H3     HN2      0.36
ATOM C4     CN1      0.50
ATOM O4     ON1     -0.45
ATOM C5     CN3T    -0.15
ATOM C5M    CN9     -0.11
ATOM H51    HN9      0.07
```

```
ATOM H52    HN9     0.07
ATOM H53    HN9     0.07
! GLYCOL STARTS HERE
BOND C6' C6''  C6'' O7'  C6'' H6''  C6'' H6'''  O7' C8'  C8'  H8'
BOND C8' H8''  C8' C9'  C9' O10'  C9' H9'  C9' H9''  O10' C11'
BOND C11' H11'  C11' H11'' C11' C12'  C12' H12'  C12'  H12''
BOND C12' O13'  O13' H13'
! GLYCOL ENDS HERE
BOND +N  C   C  O1'   C  C2'   C2' H2'  C2' H2''  C2' N2'
BOND N2' C5'   C5' H5'   C5' H5''  C6' C5'  C6' H6'
BOND C6' N     N  H1'
BOND N2' C3'   C3' O3'   C3' C4'   C4' H4'  C4' H4''
BOND C4'  N1        N1   C2        N1   C6        C2   O2
BOND C2   N3        N3   H3        N3   C4        C4   O4        C4   C5
BOND C5   C5M       C5   C6        C6   H6        C5M  H51       C5M  H52
BOND C5M  H53
IMPR C2   N1   N3   O2        C4   N3   C5   O4        C5   C4   C6   C5M
!impropers for PNA backbone.
IMPR N    -C   C6'  H1'       C    O1'  C2'  +N
IMPR C3'  O3'  C4'  N2'       N2'  C2'  C5'  C3'
DONO H3   N3
ACCE O2   C2
ACCE O4   C4
DONO H1'  N
ACCE O1'  C
ACCE O3'  C3'
ACCE O7'
ACCE O10'
DONO H13' O13'

!PNA backbone
IC O1'  C    C2'  N2'  1.23  119.76   -172.34  115.72  1.46
IC C    C2'  N2'  C3'  1.49  115.72   85.69  115.69  1.36
IC C2'  N2'  C5'  C6'  1.46  116.43    -79.13  110.77  1.55
IC H2'  C2'  N2'  C3'  1.08  106.87    -35.19  115.69  1.23
IC H2'' C2'  N2'  C5'  1.08  106.87   18.93  118.21  1.47
IC N2'  C5'  C6'  N    1.47  113.26    -70.08  111.16  1.44
IC C5'  C6'  N    H1'  1.53  111.16    -67.33  115.23  1.00
IC H5'  C5'  C6'  N    1.08  109.06  170.24  111.16  1.44
IC H5'' C5'  C6'  N    1.08  109.06   49.59  111.16  1.44
IC C6'  C5'  N2'  C3'  1.53  113.26  108.76  125.61  1.36
!IC H6'  C6'  C5'  N2'  1.08  109.44   -49.50  113.26  1.47 ! replacing this
hydrogen leads to LH helix
IC H6' C6'  C5'  N2'  1.08  109.44  170.33  113.26  1.47
IC N    C6'  C5'  N2'  1.44  111.16    -70.00  113.26  1.47
IC H1'  N    C6'  C5'  1.00  115.23    -67.33  111.16  1.53
IC C3'  N2'  C2'  H2'' 1.36  115.69   -153.43  106.87  1.08
IC O3'  C3'  N2'  C2'  1.23  120.78     -6.15  115.69  1.46
IC C4'  C3'  N2'  C2'  1.52  119.02  173.47  116.54  1.46
IC H4'  C4'  C3'  O3'  1.11  109.30  113.00  119.96  1.24
IC H4'' C4'  C3'  N2'  1.11  109.30   52.69  119.02  1.36
! Glycol linkage
IC C6'' C6'  C5'  N2'  1.52  109.29   60.19  112.08  1.46
IC H6'' C6'' C6'  C5'  1.10  109.32   66.67  109.29  1.54
IC H6''' C6'' C6' C5'  1.10  109.32   173.53  109.29  1.54
IC O7'  C6'' C6'  C5'  1.42  106.39   180.0  106.29  1.54
IC C8'  O7'  C6'' C6'  1.42  111.39   180.0  106.29  1.54
IC H8'  C8'  O7'  C6'' 1.10  109.32   -66.67  109.29  1.42
```

```
IC H8'' C8'  O7'  C6'' 1.10  109.32    66.67  109.29  1.42
IC C9'  C8'  O7'  C6'' 1.54  109.5    180.0   111.40  1.42
IC H9'  C9'  C8'  O7'  1.10  109.50   -66.67  106.65  1.42
IC H9'' C9'  C8'  O7'  1.10  109.50    66.67  106.65  1.42
IC O10' C9'  C8'  O7'  1.42  109.50   180.00  109.50  1.42
IC C11' O10' C9'  C8'  1.42  109.50   180.00  109.50  1.54
IC H11' C11' O10' C9'  1.10  109.50   -66.67  109.50  1.42
IC H11'' C11' O10' C9' 1.10  109.50    66.67  109.50  1.42
IC C12' C11' O10' C9'  1.54  109.50   180.00  109.50  1.42
IC H12' C12' C11' O10' 1.10  109.50   -66.67  109.50  1.42
IC H12'' C12' C11' O10' 1.10 109.50    66.67  109.50  1.42
IC O13' C12' C11' O10' 1.42  109.50   180.00  109.50  1.42
IC H13' O13' C12' C11' 0.96  109.5    180.00  109.50  1.54
! GLYCOL ENDS HERE
!To connect with previous
IC N    -C  -C2' -H2'' 1.35  118.45   110.49  108.45  1.08
IC C6'  N   -C   -O1'  1.45  122.56     6.77  121.77  1.23
IC C5'  C6'  N   -C    1.52  113.10  -115.86  122.56  1.35
!Adopted from THY, first three lines adjusted to PNA
BILD N2'  C3'  C4'  N1     1.37    115.53 -173.29  119.60   1.48
BILD H4'  C4'  N1   C2     1.11    109.65 -150.45  117.24   1.39
BILD C4'  C2   *N1  C6     1.48    117.24  174.09  121.34   1.3704
BILD C2   N1   C6   C5     1.3746  122.08   -0.02  121.23   1.3432
BILD C6   N1   C2   N3     1.3704  122.08    0.06  115.38   1.3813
BILD N1   N3   *C2  O2     1.3746  115.38 -179.95  121.70   1.2191
BILD N1   C2   N3   C4     1.3746  115.38   -0.07  126.46   1.3795
BILD C5   N3   *C4  O4     1.4439  114.07  179.98  120.59   1.2327
BILD C2   C4   *N3  H3     1.3813  126.46  180.00  116.77   1.0900
BILD C4   C6   *C5  C5M    1.4439  120.78 -179.94  121.63   1.5000
BILD N1   C5   *C6  H6     0.0       0.0   180.0     0.0    0.0
BILD C6   C5   C5M  H51    0.0       0.0     0.0     0.0    0.0
BILD C5   H51  *C5M H52    0.0       0.0   115.0     0.0    0.0
BILD H51  H52  *C5M H53    0.0       0.0  -115.0     0.0    0.0
!PATCHING FIRST NT LAST CT
```

```
RESI GUPN             0.00!
GROUP                    !
ATOM C      C       0.51 !
ATOM O1'    O      -0.51 !
GROUP                    !
ATOM C2'    CT2    -0.03 !
ATOM H2'    HB2     0.09 !   \   H1'
ATOM H2''   HB2     0.09 !    \ /
ATOM N2'    N      -0.36 !     N   C6''-O7'-C8'-C9'-O10'-C11'-C12'-O13'
ATOM C5'    CT2P    0.01 ! mj   \ /
ATOM H5'    HB2     0.09 !       C6'      Base
ATOM H5''   HB2     0.09 !       |        /
GROUP                    !       C5'     C4'
ATOM N      NH1    -0.47 !        \     /
ATOM H1'    H       0.31 !         N2'-C3'
                                  /
GROUP                    !       C2'
ATOM C3'    CC      0.43 !       |
ATOM O3'    O      -0.54 !       C
ATOM C4'    CT2    -0.07 !     // \
ATOM H4'    HA2     0.09 !    O1'  \
ATOM H4''   HA2     0.09 !
!BASE ATOMS STARTS HERE.
GROUP
ATOM N1     NN2B   -0.34  !
ATOM C6     CN3     0.20  !
ATOM H6     HN3     0.14  !
ATOM C2     CN1T    0.55  !
ATOM O2     ON1    -0.45  !
ATOM N3     NN2U   -0.46  !
ATOM H3     HN2     0.36  !
ATOM C4     CN1     0.53  !
ATOM O4     ON1    -0.48  !
ATOM C5     CN3    -0.15  !
ATOM H5     HN3     0.10  !
GROUP ! MINIPEG STARTS HERE
ATOM C6'    CG311   0.07 !
ATOM H6'    HB2     0.09 !
ATOM C6''   CG321   0.08 !
ATOM H6''   HCA2A   0.09 !
ATOM H6'''  HCA2A   0.09 !
ATOM O7'    OC30A  -0.34 !
ATOM C8'    CC32A  -0.01 !
ATOM H8'    HCA2A   0.09 !
ATOM H8''   HCA2A   0.09 !
ATOM C9'    CC32A  -0.01 !
ATOM H9'    HCA2A   0.09 !
ATOM H9''   HCA2A   0.09 !
ATOM O10'   OC30A  -0.34 !
ATOM C11'   CC32A  -0.01 !
ATOM H11'   HCA2A   0.09 !
ATOM H11''  HCA2A   0.09 !
ATOM C12'   CC32A  -0.01 !
ATOM H12'   HCA2A   0.09 !
ATOM H12''  HCA2A   0.09 !
ATOM O13'   OG311  -0.67 !
ATOM H13'   HGP1    0.43 !
! MINIPEG ENDS HERE
```

```
! GLYCOL STARTS HERE
BOND C6' C6''  C6'' O7'  C6'' H6''  C6'' H6'''  O7' C8'  C8'  H8'
BOND C8' H8''  C8' C9'  C9' O10'  C9' H9'  C9' H9''  O10' C11'
BOND C11' H11'  C11' H11'' C11' C12'  C12' H12'  C12'  H12''
BOND C12' O13'  O13' H13'
! GLYCOL ENDS HERE
BOND +N  C   C  O1'   C  C2'   C2' H2'  C2' H2''  C2' N2'
BOND N2' C5'   C5' H5'   C5' H5''  C6' C5'  C6' H6''
BOND C6' N     N  H1'
BOND N2' C3'   C3' O3'   C3' C4'   C4' H4'  C4' H4''
BOND C4'  N1        N1   C2        N1   C6        C2   O2
BOND C2   N3        N3   H3        N3   C4        C4   O4        C4   C5
BOND C5   H5        C5   C6        C6   H6
IMPR C2   N1   N3   O2        C4   N3   C5   O4
!impropers for PNA backbone.
IMPR N    -C   C6'  H1'       C    O1'  C2'  +N
IMPR C3'  O3'  C4'  N2'       N2'  C2'  C5'  C3'
DONO H3   N3
ACCE O2   C2
ACCE O4   C4
DONO H1'  N
ACCE O1'  C
ACCE O3'  C3'
ACCE O7'
ACCE O10'
DONO H13' O13'

!PNA backbone
IC O1'  C    C2'  N2'  1.23  119.76   -172.34  115.72  1.46
IC C    C2'  N2'  C3'  1.49  115.72   85.69  115.69  1.36
IC C2'  N2'  C5'  C6'  1.46  116.43   -79.13  110.77  1.55
IC H2'  C2'  N2'  C3'  1.08  106.87    -35.19  115.69  1.23
IC H2'' C2'  N2'  C5'  1.08  106.87   18.93  118.21  1.47
IC N2'  C5'  C6'  N    1.47  113.26    -70.08  111.16  1.44
IC C5'  C6'  N    H1'  1.53  111.16    -67.33  115.23  1.00
IC H5'  C5'  C6'  N    1.08  109.06  170.24  111.16  1.44
IC H5'' C5'  C6'  N    1.08  109.06   49.59  111.16  1.44
IC C6'  C5'  N2'  C3'  1.53  113.26  108.76  125.61  1.36
!IC H6'  C6'  C5'  N2'  1.08  109.44   49.50  113.26  1.47
IC H6' C6'  C5'  N2'  1.08  109.44  170.33  113.26  1.47
IC N    C6'  C5'  N2'  1.44  111.16    -70.00  113.26  1.47
IC H1'  N    C6'  C5'  1.00  115.23    -67.33  111.16  1.53
IC C3'  N2'  C2'  H2'' 1.36  115.69   -153.43  106.87  1.08
IC O3'  C3'  N2'  C2'  1.23  120.78     -6.15  115.69  1.46
IC C4'  C3'  N2'  C2'  1.52  119.02  173.47  116.54  1.46
IC H4'  C4'  C3'  O3'  1.11  109.30  113.00  119.96  1.24
IC H4'' C4'  C3'  N2'  1.11  109.30   52.69  119.02  1.36
! Glycol linkage
IC C6'' C6'  C5'  N2'  1.52  109.29   60.19  112.08  1.46
IC H6'' C6'' C6'  C5'  1.10  109.32   66.67  109.29  1.54
IC H6''' C6'' C6' C5'  1.10  109.32   173.53  109.29  1.54
IC O7'  C6'' C6'  C5'  1.42  106.39   180.0  106.29  1.54
IC C8'  O7'  C6'' C6'  1.42  111.39   180.0  106.29  1.54
IC H8'  C8'  O7'  C6'' 1.10  109.32   -66.67  109.29  1.42
IC H8'' C8'  O7'  C6'' 1.10  109.32    66.67  109.29  1.42
IC C9'  C8'  O7'  C6'' 1.54  109.5    180.0   111.40  1.42
IC H9'  C9'  C8'  O7'  1.10  109.50   -66.67  106.65  1.42
```

```
IC H9'' C9'  C8'  O7'  1.10  109.50    66.67  106.65  1.42
IC O10' C9'  C8'  O7'  1.42  109.50   180.00  109.50  1.42
IC C11' O10' C9'  C8'  1.42  109.50   180.00  109.50  1.54
IC H11' C11' O10' C9'  1.10  109.50   -66.67  109.50  1.42
IC H11'' C11' O10' C9' 1.10  109.50    66.67  109.50  1.42
IC C12' C11' O10' C9'  1.54  109.50   180.00  109.50  1.42
IC H12' C12' C11' O10' 1.10  109.50   -66.67  109.50  1.42
IC H12'' C12' C11' O10' 1.10 109.50    66.67  109.50  1.42
IC O13' C12' C11' O10' 1.42  109.50   180.00  109.50  1.42
IC H13' O13' C12' C11' 0.96  109.5    180.00  109.50  1.54
! GLYCOL ENDS HERE
!To connect with previous
IC N    -C  -C2' -H2'' 1.35  118.45   110.49  108.45  1.08
IC C6'  N   -C   -O1'  1.45  122.56     6.77  121.77  1.23
IC C5'  C6'  N   -C    1.52  113.10  -115.86  122.56  1.35
!Adopted from URA, first three lines adjusted to PNA
BILD N2'  C3'  C4'  N1     1.37    115.53 -173.29  119.60   1.48
BILD H4'  C4'  N1   C2     1.11    109.65 -150.45  117.24   1.39
BILD C4'  C2   *N1  C6     1.48    117.24  174.09  121.34   1.3704
BILD C2   N1   C6   C5     1.379   121.3     0.0   122.8    1.338
BILD C6   N1   C2   N3     1.380   121.3     0.0   114.8    1.373
BILD N1   N3   *C2  O2     1.379   114.8  -180.0   122.0    1.218
BILD N1   C2   N3   C4     1.379   114.8     0.0   127.0    1.383
BILD C5   N3   *C4  O4     1.440   114.7   180.0   119.8    1.227
BILD C2   C4   *N3  H3     1.373   127.0   180.0   116.5    1.03
BILD C6   C4   *C5  H5     0.0       0.0   180.0     0.0    0.0
BILD N1   C5   *C6  H6     0.0       0.0   180.0     0.0    0.0
!PATCHING FIRST NT LAST CT
```

**RESI GAPN** 0.00

```
GROUP                    !
ATOM C       C        0.51 !
ATOM O1'     O       -0.51 !
GROUP                      !   \   H1'
ATOM C2'     CT2     -0.03 !    \ /
ATOM H2'     HB2      0.09 !     N   C6''-O7'-C8'-C9'-O10'-C11'-C12'-O13'
ATOM H2''    HB2      0.09 !      \ /
ATOM N2'     N       -0.36 !      C6'      Base
ATOM C5'     CT2P     0.01 ! mj    |        /
ATOM H5'     HB2      0.09 !      C5'     C4'
ATOM H5''    HB2      0.09 !        \     /
GROUP                      !         N2'-C3'
ATOM N       NH1     -0.47 !        /
ATOM H1'     H        0.31 !       C2'
GROUP ! MINIPEG STARTS HERE         |
ATOM C6'     CG311    0.07 !        C
ATOM H6'     HB2      0.09 !      // \
ATOM C6''    CG321    0.06 !    O1'   \
ATOM H6''    HCA2A    0.09 !
ATOM H6'''   HCA2A    0.09 !
ATOM O7'     OC30A   -0.34 !
ATOM C8'     CC32A   -0.01 !
ATOM H8'     HCA2A    0.09 !
ATOM H8''    HCA2A    0.09 !
ATOM C9'     CC32A   -0.01 !
ATOM H9'     HCA2A    0.09 !
ATOM H9''    HCA2A    0.09 !
ATOM O10'    OC30A   -0.34 !
ATOM C11'    CC32A   -0.01 !
ATOM H11'    HCA2A    0.09 !
ATOM H11''   HCA2A    0.09 !
ATOM C12'    CC32A   -0.01 !
ATOM H12'    HCA2A    0.09 !
ATOM H12''   HCA2A    0.09 !
ATOM O13'    OG311   -0.67 !
ATOM H13'    HGP1     0.43 !
! MINIPEG ENDS HERE
GROUP                      !
ATOM C3'     CC       0.43 !
ATOM O3'     O       -0.54 !
ATOM C4'     CT2     -0.07 !
ATOM H4'     HA2      0.09 !
ATOM H4''    HA2      0.09 !
!BASE ATOMS STARTS HERE.
GROUP
ATOM N9      NN2     -0.05  !
ATOM C5      CN5      0.28  !
ATOM N7      NN4     -0.71  !
ATOM C8      CN4      0.34  !
ATOM H8      HN3      0.12  !
ATOM N1      NN3A    -0.74  !
ATOM C2      CN4      0.50  !
ATOM H2      HN3      0.13  !
ATOM N3      NN3A    -0.75
ATOM C4      CN5      0.43
ATOM C6      CN2      0.46
ATOM N6      NN1     -0.77
```

```
ATOM H61    HN1     0.38
ATOM H62    HN1     0.38
! GLYCOL STARTS HERE
BOND C6' C6''  C6'' O7'  C6'' H6''  C6'' H6'''  O7' C8'  C8'  H8'
BOND C8' H8''  C8' C9'  C9' O10'  C9' H9'  C9' H9''  O10' C11'
BOND C11' H11'  C11' H11'' C11' C12'  C12' H12'  C12'  H12''
BOND C12' O13'  O13' H13'
! GLYCOL ENDS HERE
BOND +N   C     C   O1'  C   C2'   C2' H2'  C2' H2''  C2' N2'
BOND N2'  C5'   C5' H5'  C5' H5''  C6' C5'  C6' H6'
BOND C6'  N     N   H1'
BOND N2'  C3'   C3' O3'  C3' C4'   C4' H4'  C4' H4''
BOND C4'  N9        N9   C4        N9   C8        C4   N3
BOND C4   C5        N3   C2        C2   N1        N1   C6        C6   N6
BOND N6   H61       N6   H62       C6   C5        C5   N7        N7   C8
BOND C8   H8        C2   H2
IMPR N6   C6   H61  H62        C6   N1   C5   N6
!impropers for PNA backbone.
IMPR N    -C   C6'  H1'       C    O1'  C2'  +N
IMPR C3'  O3'  C4'  N2'       N2'  C2'  C5'  C3'
DONO H61  N6
DONO H62  N6
ACCE N3
ACCE N7
ACCE N1
DONO H1'  N
ACCE O1'  C
ACCE O3'  C3'
ACCE O7'
ACCE O10'
DONO H13' O13'

!PNA backbone
IC O1'  C    C2'  N2'  1.23  119.76   -172.34  115.72  1.46
IC C    C2'  N2'  C3'  1.49  115.72   85.69  115.69  1.36
IC C2'  N2'  C5'  C6'  1.46  116.43    -79.13  110.77  1.55
IC H2'  C2'  N2'  C3'  1.08  106.87    -35.19  115.69  1.23
IC H2'' C2'  N2'  C5'  1.08  106.87   18.93  118.21  1.47
IC N2'  C5'  C6'  N    1.47  113.26    -70.08  111.16  1.44
IC C5'  C6'  N    H1'  1.53  111.16    -67.33  115.23  1.00
IC H5'  C5'  C6'  N    1.08  109.06  170.24  111.16  1.44
IC H5'' C5'  C6'  N    1.08  109.06   49.59  111.16  1.44
IC C6'  C5'  N2'  C3'  1.53  113.26  108.76  125.61  1.36
!IC H6'  C6'  C5'  N2'  1.08  109.44   -49.50  113.26  1.47
IC H6' C6'  C5'  N2'  1.08  109.44  170.33  113.26  1.47
IC N    C6'  C5'  N2'  1.44  111.16    -70.00  113.26  1.47
IC H1'  N    C6'  C5'  1.00  115.23    -67.33  111.16  1.53
IC C3'  N2'  C2'  H2'' 1.36  115.69   -153.43  106.87  1.08
IC O3'  C3'  N2'  C2'  1.23  120.78     -6.15  115.69  1.46
IC C4'  C3'  N2'  C2'  1.52  119.02  173.47  116.54  1.46
IC H4'  C4'  C3'  O3'  1.11  109.30  113.00  119.96  1.24
IC H4'' C4'  C3'  N2'  1.11  109.30   52.69  119.02  1.36
! Glycol linkage
IC C6'' C6'  C5'  N2'  1.52  109.29   60.19  112.08  1.46
IC H6'' C6'' C6'  C5'  1.10  109.32   66.67  109.29  1.54
IC H6''' C6'' C6' C5'  1.10  109.32   173.53  109.29  1.54
IC O7'  C6'' C6'  C5'  1.42  106.39   180.0  106.29  1.54
IC C8'  O7'  C6'' C6'  1.42  111.39   180.0  106.29  1.54
```

```
IC H8'  C8'  O7'  C6'' 1.10  109.32   -66.67  109.29  1.42
IC H8'' C8'  O7'  C6'' 1.10  109.32    66.67  109.29  1.42
IC C9'  C8'  O7'  C6'' 1.54  109.5    180.0   111.40  1.42
IC H9'  C9'  C8'  O7'  1.10  109.50   -66.67  106.65  1.42
IC H9'' C9'  C8'  O7'  1.10  109.50    66.67  106.65  1.42
IC O10' C9'  C8'  O7'  1.42  109.50   180.00  109.50  1.42
IC C11' O10' C9'  C8'  1.42  109.50   180.00  109.50  1.54
IC H11' C11' O10' C9'  1.10  109.50   -66.67  109.50  1.42
IC H11'' C11' O10' C9' 1.10  109.50    66.67  109.50  1.42
IC C12' C11' O10' C9'  1.54  109.50   180.00  109.50  1.42
IC H12' C12' C11' O10' 1.10  109.50   -66.67  109.50  1.42
IC H12'' C12' C11' O10' 1.10 109.50    66.67  109.50  1.42
IC O13' C12' C11' O10' 1.42  109.50   180.00  109.50  1.42
IC H13' O13' C12' C11' 0.96  109.5    180.00  109.50  1.54
! GLYCOL ENDS HERE
!To connect with previous
IC N    -C  -C2' -H2'' 1.35  118.45   110.49  108.45  1.08
IC C6'  N   -C   -O1'  1.45  122.56     6.77  121.77  1.23
IC C5'  C6'  N   -C    1.52  113.10  -115.86  122.56  1.35
!Adopted from ADE, first three lines adjusted to PNA
BILD O3'  C3'  C4'  N9     1.23    120.40   10.91  109.15   1.46
BILD H4'  C4'  N9   C4     1.11    109.86 -150.27  124.65   1.36
BILD C4'  C4   *N9  C8     1.46    124.65  177.35  107.10   1.374
BILD C4   N9   C8   N7     1.376   106.0     0.0   113.6    1.312
BILD C8   N9   C4   C5     1.367   106.0     0.0   105.6    1.382
BILD C8   N7   C5   C6     0.0       0.0   180.0     0.0    0.0
BILD N7   C5   C6   N1     0.0       0.0   180.0     0.0    0.0
BILD C5   C6   N1   C2     0.0       0.0     0.0     0.0    0.0
BILD N9   C5   *C4  N3     1.376   105.6  -180.0   126.9    1.342
BILD C5   N1   *C6  N6     1.409   117.6  -180.0   121.2    1.337
BILD N1   C6   N6   H61    1.337   121.2     0.0   119.0    1.01
BILD H61  C6   *N6  H62    1.01    119.0   180.0   119.00   1.01
BILD C5   N1   *C6  N6     1.409   117.6  -180.0   119.0    1.337
BILD N1   C6   N6   H61    1.337   119.0     0.0   119.0    1.01
BILD H61  C6   *N6  H62    1.01    119.0   180.0   121.00   1.01
BILD N9   N7   *C8   H8    0.0       0.0   180.0     0.0    0.0
BILD N1   N3   *C2   H2    0.0       0.0   180.0     0.0    0.0
!PATCHING FIRST NT LAST CT
```

**Archive for the optimized model compound used for our QM model during the parameterization procedure.**

```
1\1\GINC-GPU005\FOpt\RMP2-FC\6-31+G(d)\C12H23N3O4\TAMEZA\22-Dec-2020\0
\\#p opt=(calcfc,noeigen,modredundant) freq mp2/6-31+g(d) pop=(mk,dipo
le) density=all nosym\\Gamma modified dimer optimization. AT15Dec2020\
\0,1\N,0.0544479968,-0.8056089729,-0.2290340167\C,0.0845262529,-1.1232
124912,1.1867805184\C,-1.1204550267,-0.4270494966,1.8547510511\N,-2.29
92045788,-0.4311347604,0.9983288125\C,-2.9083088316,-1.73436792,0.7203
162575\C,1.0176304812,-1.1148421568,-1.1315985462\C,0.7269185507,-0.65
82961701,-2.5624896698\N,-0.613678278,-0.1391804922,-2.7985245253\C,-0
.8072332809,1.3075221356,-2.8550231384\C,-2.8597792258,0.6910469774,0.
4488831908\C,-2.2771002797,2.0437839042,0.8181369506\O,-3.8180137733,0
.6174144987,-0.3438602299\C,-1.6271913665,-1.0619807139,-2.9554790546\
C,-2.9910054564,-0.5498608277,-3.3528030935\O,-1.4036428049,-2.2688605
965,-2.7689034488\O,2.0664052227,-1.71746927,-0.8673754111\H,-1.829445
9705,1.5661172521,-2.5745522748\H,-0.1286405943,1.7822130681,-2.139265
7189\H,-0.5944772261,1.7108233098,-3.8538318461\H,-2.9351484837,0.1651
063947,-4.1795588055\H,-3.4904055472,-0.0703036681,-2.5032109662\H,-3.
5796940831,-1.414912288,-3.66137449\H,0.903018113,-1.5287581915,-3.201
5038542\H,1.4570970425,0.1127548979,-2.8318646829\H,-0.7576257467,-0.3
077293897,-0.5802100821\H,0.005923305,-2.21294357,1.3115468767\H,-0.84
92993915,0.60248228,2.0912035595\H,-1.3465513501,-0.9306024823,2.80025
56873\H,-2.3361478114,2.2318914577,1.8950244269\H,-2.8700492466,2.7943
293298,0.2941393601\H,-1.2289915713,2.1417416937,0.5150949642\H,-2.804
4727818,-2.3606261721,1.6113487162\H,-2.4232594058,-2.230093364,-0.128
9652919\H,-3.9640040621,-1.5872549467,0.4901826307\C,1.3712361013,-0.6
54152906,1.8555328928\H,1.5259514615,0.4178571016,1.6389093984\H,2.232
2077947,-1.2168845496,1.4791004892\O,1.1950912424,-0.8595559814,3.2549
393805\C,2.3932958145,-0.5821567815,3.9762929555\H,2.6959374478,0.4652
665778,3.8429674905\H,2.1750577781,-0.7691357012,5.028211074\H,3.20787
02396,-1.2385066388,3.6445826237\\Version=ES64L-G16RevC.01\HF=-930.635
8294\MP2=-933.4464979\RMSD=7.517e-09\RMSF=1.296e-03\Dipole=0.2166164,1
.9828338,0.8721006\Quadrupole=-7.1946727,-2.9293939,10.1240665,3.88208
46,1.3240634,-8.2626965\PG=C01 [X(C12H23N3O4)]\\@
```

**Coordinates for the miniPEG-modified γPNA MD starting structures.**

Model 1

| | | | | | |
|---|---|---|---|---|---|
| 1 | GGPN | N | 15.5470000000 | 30.3860000000 | 25.0200000000 |
| 1 | GGPN | HT1 | 16.4092228967 | 30.9587546020 | 24.9193826937 |
| 1 | GGPN | HT2 | 15.0018786745 | 30.7211712575 | 25.8398186414 |
| 1 | GGPN | HT3 | 14.9690746261 | 30.4754562621 | 24.1600003050 |
| 1 | GGPN | C6' | 16.1030000000 | 28.2540000000 | 25.3210000000 |
| 1 | GGPN | H6' | 15.8241505289 | 28.1152888244 | 26.3464927586 |
| 1 | GGPN | C | 19.3180000000 | 27.1830000000 | 24.3740000000 |
| 1 | GGPN | O1' | 19.9715626551 | 26.1642417579 | 24.1551678724 |
| 1 | GGPN | C2' | 18.0510000000 | 26.8550000000 | 25.1050000000 |
| 1 | GGPN | H2' | 18.2767498776 | 26.0965353728 | 25.8393868193 |
| 1 | GGPN | H2'' | 17.3377022417 | 26.4828121423 | 24.3851124359 |
| 1 | GGPN | N2' | 17.3920000000 | 27.9260000000 | 25.8150000000 |
| 1 | GGPN | C5' | 18.0921697341 | 26.8845785975 | 25.1210548971 |
| 1 | GGPN | H5' | 18.7769937133 | 27.1749267717 | 24.4476123092 |
| 1 | GGPN | H5'' | 18.0554364503 | 25.9688449988 | 25.5295809135 |
| 1 | GGPN | C6'' | 16.4240000000 | 29.4540000000 | 23.1430000000 |
| 1 | GGPN | H6'' | 17.1007092526 | 30.3225170640 | 23.2912673892 |
| 1 | GGPN | H6''' | 15.4429937507 | 29.8163375390 | 22.7680616471 |
| 1 | GGPN | O7' | 17.0389990733 | 28.5077711218 | 22.1098847838 |
| 1 | GGPN | C8' | 17.2173271654 | 29.1911417830 | 20.8837409786 |
| 1 | GGPN | H8' | 17.8870317615 | 30.0611789612 | 21.0527311468 |
| 1 | GGPN | H8'' | 16.2293162597 | 29.5549994361 | 20.5295254048 |
| 1 | GGPN | C9' | 17.8315392671 | 28.2806743613 | 19.8185214856 |
| 1 | GGPN | H9' | 17.1618346710 | 27.4106371831 | 19.6495313174 |
| 1 | GGPN | H9'' | 18.8195501728 | 27.9168167081 | 20.1727370594 |
| 1 | GGPN | O10' | 18.0098673592 | 28.9640450224 | 18.5923776804 |
| 1 | GGPN | C11' | 18.5786410120 | 28.0889379227 | 17.6369149151 |
| 1 | GGPN | H11' | 17.8991271834 | 27.2214181892 | 17.4965686180 |
| 1 | GGPN | H11'' | 19.5568426852 | 27.7275977142 | 18.0197743601 |
| 1 | GGPN | C12' | 18.7897169820 | 28.7876031411 | 16.2922164926 |
| 1 | GGPN | H12' | 19.4738134177 | 29.6537079248 | 16.4194122453 |
| 1 | GGPN | H12'' | 17.8160979159 | 29.1475283998 | 15.8962065033 |
| 1 | GGPN | O13' | 19.3585702554 | 27.8680001951 | 15.3795493161 |
| 1 | GGPN | H13' | 19.4718530764 | 28.3482113471 | 14.5560420289 |
| 1 | GGPN | C3' | 17.8870000000 | 28.4220000000 | 26.9360000000 |
| 1 | GGPN | O3' | 18.9430000000 | 27.9920000000 | 27.3520000000 |
| 1 | GGPN | C4' | 17.1490000000 | 29.5270000000 | 27.6770000000 |
| 1 | GGPN | H4' | 16.0587921761 | 29.4157459316 | 27.4949497362 |
| 1 | GGPN | H4'' | 17.4856877770 | 30.5100640515 | 27.2841905336 |
| 1 | GGPN | N9 | 17.4070000000 | 29.4660000000 | 29.1070000000 |
| 1 | GGPN | C4 | 18.3860000000 | 30.1340000000 | 29.7710000000 |
| 1 | GGPN | N2 | 20.9970000000 | 32.2760000000 | 29.7970000000 |
| 1 | GGPN | H21 | 21.2358360794 | 32.3977853516 | 28.8336072165 |
| 1 | GGPN | H22 | 21.5196452037 | 32.8059771230 | 30.4648070382 |
| 1 | GGPN | N3 | 19.2940000000 | 30.9710000000 | 29.2340000000 |
| 1 | GGPN | C2 | 20.0670000000 | 31.4800000000 | 30.1580000000 |
| 1 | GGPN | N1 | 20.0050000000 | 31.2520000000 | 31.4520000000 |
| 1 | GGPN | H1 | 20.6522118020 | 31.7122826298 | 32.0717561724 |
| 1 | GGPN | C6 | 19.1030000000 | 30.4180000000 | 32.0050000000 |
| 1 | GGPN | O6 | 19.1410000000 | 30.3080000000 | 33.2160000000 |
| 1 | GGPN | C5 | 18.2250000000 | 29.8070000000 | 31.0810000000 |
| 1 | GGPN | N7 | 17.1700000000 | 28.9200000000 | 31.2520000000 |
| 1 | GGPN | C8 | 16.7310000000 | 28.7500000000 | 30.0430000000 |
| 1 | GGPN | H8 | 15.8986030265 | 28.0987333712 | 29.7765429890 |
| 2 | GGPN | C | 24.6370000000 | 26.6570000000 | 24.4480000000 |
| 2 | GGPN | O1' | 25.1786574884 | 25.7378928269 | 23.8358324007 |
| 2 | GGPN | C2' | 23.1640000000 | 26.7260000000 | 24.4970000000 |
| 2 | GGPN | H2' | 22.7890074624 | 25.8164386725 | 24.9422628125 |
| 2 | GGPN | H2'' | 22.7884391148 | 26.8353176386 | 23.4904296551 |
| 2 | GGPN | N2' | 22.6320000000 | 27.8400000000 | 25.2790000000 |
| 2 | GGPN | C5' | 22.4552768076 | 30.5054068271 | 24.6242865454 |
| 2 | GGPN | H5' | 23.2188844415 | 30.8696859489 | 24.1148159891 |
| 2 | GGPN | H5'' | 21.7344938431 | 31.0073277002 | 25.0758432973 |
| 2 | GGPN | N | 19.8870000000 | 28.3620000000 | 24.4690000000 |
| 2 | GGPN | H1' | 19.2934920549 | 29.1540115480 | 24.5439007507 |
| 2 | GGPN | C6' | 22.2330000000 | 28.9970000000 | 24.4970000000 |
| 2 | GGPN | H6' | 22.6967814670 | 28.5034713782 | 25.3381849302 |
| 2 | GGPN | C6'' | 20.6300000000 | 29.9660000000 | 22.9280000000 |

| | | | | | |
|---|---|---|---|---|---|
| 2 | GGPN | H6'' | 19.8189344186 | 30.4758379528 | 23.4905553888 |
| 2 | GGPN | H6''' | 20.1887917142 | 29.1618496092 | 22.3011761983 |
| 2 | GGPN | O7' | 21.3289662307 | 30.9822240740 | 22.0226631347 |
| 2 | GGPN | C8' | 20.3935065134 | 31.5378098488 | 21.1179728208 |
| 2 | GGPN | H8' | 19.5855377160 | 32.0331836708 | 21.6974707184 |
| 2 | GGPN | H8'' | 19.9553950116 | 30.7191953272 | 20.5080915279 |
| 2 | GGPN | C9' | 21.0505200608 | 32.5621461034 | 20.1906279896 |
| 2 | GGPN | H9' | 21.8584888582 | 32.0667722814 | 19.6111300920 |
| 2 | GGPN | H9'' | 21.4886315625 | 33.3807606250 | 20.8005092825 |
| 2 | GGPN | O10' | 20.1150603434 | 33.1177318782 | 19.2859376757 |
| 2 | GGPN | C11' | 20.7614898967 | 34.0575730970 | 18.4486490061 |
| 2 | GGPN | H11' | 21.5732535776 | 33.5425647457 | 17.8920227951 |
| 2 | GGPN | H11'' | 21.2033962819 | 34.8565530893 | 19.0814019855 |
| 2 | GGPN | C12' | 19.7851983548 | 34.6851645327 | 17.4517117889 |
| 2 | GGPN | H12' | 18.9719456663 | 35.2092805287 | 17.9978131316 |
| 2 | GGPN | H12'' | 19.3418029619 | 33.8952921851 | 16.8084339412 |
| 2 | GGPN | O13' | 20.4844494873 | 35.6130487600 | 16.6440585746 |
| 2 | GGPN | H13' | 19.8354158810 | 35.9785433477 | 16.0384439413 |
| 2 | GGPN | C3' | 22.5970000000 | 27.7770000000 | 26.6130000000 |
| 2 | GGPN | O3' | 23.0200000000 | 26.7950000000 | 27.2020000000 |
| 2 | GGPN | C4' | 22.0740000000 | 28.9620000000 | 27.4010000000 |
| 2 | GGPN | H4' | 21.0555694473 | 29.2107197835 | 27.0338222717 |
| 2 | GGPN | H4'' | 22.7463715783 | 29.8288093049 | 27.2265296724 |
| 2 | GGPN | N9 | 22.0110000000 | 28.6860000000 | 28.8390000000 |
| 2 | GGPN | C4 | 22.7780000000 | 29.2750000000 | 29.7960000000 |
| 2 | GGPN | N2 | 25.3090000000 | 31.3420000000 | 30.6770000000 |
| 2 | GGPN | H21 | 25.7264958450 | 31.6042377017 | 29.8069835592 |
| 2 | GGPN | H22 | 25.7304746234 | 31.7016450774 | 31.5094749607 |
| 2 | GGPN | N3 | 23.7860000000 | 30.1490000000 | 29.5900000000 |
| 2 | GGPN | C2 | 24.2810000000 | 30.5800000000 | 30.7230000000 |
| 2 | GGPN | N1 | 23.8810000000 | 30.2380000000 | 31.9330000000 |
| 2 | GGPN | H1 | 24.3374462056 | 30.6375885344 | 32.7376200124 |
| 2 | GGPN | C6 | 22.8740000000 | 29.3650000000 | 32.1640000000 |
| 2 | GGPN | O6 | 22.6020000000 | 29.1460000000 | 33.3320000000 |
| 2 | GGPN | C5 | 22.2910000000 | 28.8260000000 | 30.9880000000 |
| 2 | GGPN | N7 | 21.2860000000 | 27.8820000000 | 30.7940000000 |
| 2 | GGPN | C8 | 21.1920000000 | 27.8200000000 | 29.5000000000 |
| 2 | GGPN | H8 | 20.5247956599 | 27.1437937422 | 28.9656208495 |
| 3 | GCPN | C | 29.8510000000 | 25.4320000000 | 26.5250000000 |
| 3 | GCPN | O1' | 30.2863219527 | 24.2816164963 | 26.5214514879 |
| 3 | GCPN | C2' | 28.3730000000 | 25.4330000000 | 26.2320000000 |
| 3 | GCPN | H2' | 27.9520844797 | 24.5057791937 | 26.5913418233 |
| 3 | GCPN | H2'' | 28.2356241586 | 25.5205390236 | 25.1645265145 |
| 3 | GCPN | N2' | 27.6020000000 | 26.5230000000 | 26.8540000000 |
| 3 | GCPN | C5' | 27.5888272025 | 28.9368767425 | 26.9485218779 |
| 3 | GCPN | H5' | 28.4852840328 | 29.3963149736 | 26.9518449941 |
| 3 | GCPN | H5'' | 26.7407400012 | 29.3742879047 | 27.2712430490 |
| 3 | GCPN | N | 25.2900000000 | 27.4340000000 | 25.1880000000 |
| 3 | GCPN | H1' | 24.7505261900 | 28.1801186312 | 25.5278282666 |
| 3 | GCPN | C6' | 27.4410000000 | 27.7030000000 | 26.0560000000 |
| 3 | GCPN | H6' | 27.6734609273 | 26.8223863151 | 26.6362906959 |
| 3 | GCPN | C6'' | 26.6550000000 | 28.7550000000 | 24.1590000000 |
| 3 | GCPN | H6'' | 25.7736247523 | 29.3801184157 | 24.4171381801 |
| 3 | GCPN | H6''' | 26.3543224997 | 27.9937995304 | 23.4077360206 |
| 3 | GCPN | O7' | 27.7457031754 | 29.6465521202 | 23.5620063651 |
| 3 | GCPN | C8' | 27.2549298197 | 30.2872478437 | 22.3997346657 |
| 3 | GCPN | H8' | 26.3687933617 | 30.8981591065 | 22.6746459624 |
| 3 | GCPN | H8'' | 26.9494911090 | 29.5118402213 | 21.6652438029 |
| 3 | GCPN | C9' | 28.3151233464 | 31.1914461520 | 21.7678208226 |
| 3 | GCPN | H9' | 29.2012598045 | 30.5805348891 | 21.4929095258 |
| 3 | GCPN | H9'' | 28.6205620571 | 31.9668537743 | 22.5023116854 |
| 3 | GCPN | O10' | 27.8243499907 | 31.8321418755 | 20.6055491232 |
| 3 | GCPN | C11' | 28.8330722085 | 32.6566819082 | 20.0534275589 |
| 3 | GCPN | H11' | 29.7124081459 | 32.0265325184 | 19.8010257560 |
| 3 | GCPN | H11'' | 29.1317103985 | 33.4128514036 | 20.8104279156 |
| 3 | GCPN | C12' | 28.3450323683 | 33.3710604538 | 18.7915319975 |
| 3 | GCPN | H12' | 27.4690747307 | 34.0101020567 | 19.0336646803 |
| 3 | GCPN | H12'' | 28.0497724781 | 32.6237831715 | 18.0242625208 |
| 3 | GCPN | O13' | 29.3912868121 | 34.1780092226 | 18.2851622477 |
| 3 | GCPN | H13' | 29.0406770624 | 34.6034066432 | 17.4992170601 |
| 3 | GCPN | C3' | 27.2270000000 | 26.4750000000 | 28.1340000000 |
| 3 | GCPN | O3' | 27.5870000000 | 25.5550000000 | 28.8430000000 |
| 3 | GCPN | C4' | 26.3770000000 | 27.5810000000 | 28.7260000000 |

| | | | | | |
|---|---|---|---|---|---|
| 3 | GCPN | H4' | 25.4590579292 | 27.6873639154 | 28.1097302855 |
| 3 | GCPN | H4'' | 26.9550841081 | 28.5286004199 | 28.6861064307 |
| 3 | GCPN | N1 | 25.9810000000 | 27.3130000000 | 30.1450000000 |
| 3 | GCPN | C6 | 25.0280000000 | 26.3830000000 | 30.4050000000 |
| 3 | GCPN | H6 | 24.6146213600 | 25.8505137477 | 29.5491419459 |
| 3 | GCPN | C5 | 24.6480000000 | 26.1680000000 | 31.6780000000 |
| 3 | GCPN | H5 | 23.8744879223 | 25.4453167715 | 31.9378226002 |
| 3 | GCPN | C2 | 26.6110000000 | 27.9600000000 | 31.2120000000 |
| 3 | GCPN | O2 | 27.5480000000 | 28.7280000000 | 31.0310000000 |
| 3 | GCPN | N3 | 26.2300000000 | 27.7740000000 | 32.4840000000 |
| 3 | GCPN | C4 | 25.2820000000 | 26.9090000000 | 32.6810000000 |
| 3 | GCPN | N4 | 24.9190000000 | 26.7610000000 | 33.9010000000 |
| 3 | GCPN | H41 | 25.3520192494 | 27.2755726126 | 34.6410738855 |
| 3 | GCPN | H42 | 24.1899577256 | 26.1257348041 | 34.1558244357 |
| 4 | GAPN | C | 34.3260000000 | 24.3040000000 | 29.9790000000 |
| 4 | GAPN | O1' | 35.0655284856 | 23.3314391854 | 29.8371441547 |
| 4 | GAPN | C2' | 33.0540000000 | 24.2930000000 | 29.1590000000 |
| 4 | GAPN | H2' | 32.5792544735 | 23.3292601083 | 29.2685584847 |
| 4 | GAPN | H2'' | 33.3067908132 | 24.4729787716 | 28.1246473513 |
| 4 | GAPN | N2' | 32.0590000000 | 25.3170000000 | 29.5500000000 |
| 4 | GAPN | C5' | 32.3035695222 | 27.9891161039 | 29.5195495741 |
| 4 | GAPN | H5' | 33.2150475689 | 28.3459013858 | 29.6944266875 |
| 4 | GAPN | H5'' | 31.4543853812 | 28.5054147668 | 29.5510449260 |
| 4 | GAPN | N | 30.3520000000 | 26.3610000000 | 27.3140000000 |
| 4 | GAPN | H1' | 29.8529021442 | 27.2099254221 | 27.3015899431 |
| 4 | GAPN | C6' | 32.2320000000 | 26.6090000000 | 28.8630000000 |
| 4 | GAPN | H6' | 32.1231371123 | 25.8607224373 | 29.6340287914 |
| 4 | GAPN | C6'' | 32.0220000000 | 27.9420000000 | 26.7830000000 |
| 4 | GAPN | H6'' | 31.0820107901 | 28.5334350029 | 26.8122507018 |
| 4 | GAPN | H6''' | 31.9973313531 | 27.2618499362 | 25.9049236768 |
| 4 | GAPN | O7' | 33.2111518094 | 28.8950534567 | 26.6469592604 |
| 4 | GAPN | C8' | 33.0828249189 | 29.6511747034 | 25.4578260175 |
| 4 | GAPN | H8' | 32.1336176280 | 30.2267172850 | 25.5000500643 |
| 4 | GAPN | H8'' | 33.0489381910 | 28.9551322183 | 24.5927230392 |
| 4 | GAPN | C9' | 34.2533865476 | 30.6203162942 | 25.2804843965 |
| 4 | GAPN | H9' | 35.2025938385 | 30.0447737127 | 25.2382603497 |
| 4 | GAPN | H9'' | 34.2872732756 | 31.3163587793 | 26.1455873747 |
| 4 | GAPN | O10' | 34.1250596571 | 31.3764375410 | 24.0913511536 |
| 4 | GAPN | C11' | 35.2248307750 | 32.2578562607 | 23.9655356983 |
| 4 | GAPN | H11' | 36.1612832807 | 31.6608132471 | 23.9405765523 |
| 4 | GAPN | H11'' | 35.2459627177 | 32.9323983138 | 24.8479035774 |
| 4 | GAPN | C12' | 35.1273777806 | 33.0976278607 | 22.6903154924 |
| 4 | GAPN | H12' | 34.1970019686 | 33.7045956347 | 22.7074956851 |
| 4 | GAPN | H12'' | 35.1123225315 | 32.4330105680 | 21.8001686601 |
| 4 | GAPN | O13' | 36.2492239163 | 33.9569417180 | 22.6177486778 |
| 4 | GAPN | H13' | 36.1449301144 | 34.4603606744 | 21.8070131122 |
| 4 | GAPN | C3' | 31.1920000000 | 25.0740000000 | 30.5500000000 |
| 4 | GAPN | O3' | 31.1720000000 | 24.0060000000 | 31.1450000000 |
| 4 | GAPN | C4' | 30.2810000000 | 26.1710000000 | 30.9960000000 |
| 4 | GAPN | H4' | 29.6669118747 | 26.5007447540 | 30.1310154351 |
| 4 | GAPN | H4'' | 30.8958821923 | 27.0245203554 | 31.3530112301 |
| 4 | GAPN | N9 | 29.3840000000 | 25.7210000000 | 32.0910000000 |
| 4 | GAPN | C5 | 28.4940000000 | 25.3360000000 | 34.0540000000 |
| 4 | GAPN | N7 | 27.6660000000 | 24.6940000000 | 33.1260000000 |
| 4 | GAPN | C8 | 28.2610000000 | 24.9390000000 | 31.9910000000 |
| 4 | GAPN | H8 | 27.9169552288 | 24.5662866554 | 31.0262826792 |
| 4 | GAPN | N1 | 29.4730000000 | 26.1190000000 | 36.0850000000 |
| 4 | GAPN | C2 | 30.4580000000 | 26.6130000000 | 35.3570000000 |
| 4 | GAPN | H2 | 31.2540954652 | 27.1133755698 | 35.9061879131 |
| 4 | GAPN | N3 | 30.5950000000 | 26.5740000000 | 34.0440000000 |
| 4 | GAPN | C4 | 29.5640000000 | 25.9210000000 | 33.4440000000 |
| 4 | GAPN | C6 | 28.4950000000 | 25.4870000000 | 35.4510000000 |
| 4 | GAPN | N6 | 27.5760000000 | 25.0260000000 | 36.2610000000 |
| 4 | GAPN | H61 | 26.8675255374 | 24.3959981748 | 35.9429408294 |
| 4 | GAPN | H62 | 27.5507569966 | 25.2879455120 | 37.2257524758 |
| 5 | GTPN | C | 37.6640000000 | 22.5730000000 | 34.0530000000 |
| 5 | GTPN | O1' | 38.0740022297 | 21.4145782101 | 33.9995478882 |
| 5 | GTPN | C2' | 36.6200000000 | 22.9390000000 | 33.0240000000 |
| 5 | GTPN | H2' | 36.1267389646 | 22.0329648606 | 32.7051713276 |
| 5 | GTPN | H2'' | 37.1175861056 | 23.4109169563 | 32.1899974226 |
| 5 | GTPN | N2' | 35.5610000000 | 23.8650000000 | 33.4640000000 |
| 5 | GTPN | C5' | 35.3179987573 | 26.3830692198 | 34.0243214507 |
| 5 | GTPN | H5' | 36.0158337997 | 26.7747795573 | 34.6241465742 |

| | | | | | |
|---|---|---|---|---|---|
| 5 | GTPN | H5'' | 34.4275170842 | 26.7970940222 | 33.8350418515 |
| 5 | GTPN | N | 34.3720000000 | 25.0610000000 | 31.0610000000 |
| 5 | GTPN | H1' | 33.6632370406 | 25.7438683969 | 31.1065198204 |
| 5 | GTPN | C6' | 35.7720000000 | 25.2690000000 | 33.0790000000 |
| 5 | GTPN | H6' | 35.7319063105 | 24.3267087365 | 33.6050565628 |
| 5 | GTPN | C6'' | 35.6800000000 | 27.0100000000 | 31.3180000000 |
| 5 | GTPN | H6'' | 34.6384902720 | 27.3830028102 | 31.2162303041 |
| 5 | GTPN | H6''' | 36.0085933511 | 26.5779034852 | 30.3486962848 |
| 5 | GTPN | O7' | 36.5982221400 | 28.1776566417 | 31.6845324934 |
| 5 | GTPN | C8' | 36.5392426617 | 29.1662840968 | 30.6739078469 |
| 5 | GTPN | H8' | 35.4902179241 | 29.5191996208 | 30.5789112833 |
| 5 | GTPN | H8'' | 36.8603210031 | 28.7141002958 | 29.7113772639 |
| 5 | GTPN | C9' | 37.4445570776 | 30.3558970737 | 30.9996788919 |
| 5 | GTPN | H9' | 38.4935818152 | 30.0029815496 | 31.0946754555 |
| 5 | GTPN | H9'' | 37.1234787362 | 30.8080808746 | 31.9622094749 |
| 5 | GTPN | O10' | 37.3855775993 | 31.3445245288 | 29.9890542454 |
| 5 | GTPN | C11' | 38.2347830425 | 32.4244161288 | 30.3280369109 |
| 5 | GTPN | H11' | 39.2734323389 | 32.0443415404 | 30.4318519419 |
| 5 | GTPN | H11'' | 37.9033292599 | 32.8494408654 | 31.2993859612 |
| 5 | GTPN | C12' | 38.2023939908 | 33.5197986212 | 29.2603346375 |
| 5 | GTPN | H12' | 37.1686729339 | 33.9123990607 | 29.1526784221 |
| 5 | GTPN | H12'' | 38.5387760129 | 33.1072997357 | 28.2851444027 |
| 5 | GTPN | O13' | 39.0667866562 | 34.5697239326 | 29.6511122730 |
| 5 | GTPN | H13' | 39.8788624660 | 34.4423394519 | 29.1552230987 |
| 5 | GTPN | C3' | 34.4810000000 | 23.4020000000 | 34.1320000000 |
| 5 | GTPN | O3' | 34.4020000000 | 22.2290000000 | 34.4840000000 |
| 5 | GTPN | C4' | 33.3350000000 | 24.3560000000 | 34.4390000000 |
| 5 | GTPN | H4' | 32.9223195295 | 24.7262894844 | 33.4766544624 |
| 5 | GTPN | H4'' | 33.7405480654 | 25.2136865587 | 35.0164365250 |
| 5 | GTPN | N1 | 32.2350000000 | 23.7290000000 | 35.2220000000 |
| 5 | GTPN | C6 | 31.2350000000 | 23.0600000000 | 34.5610000000 |
| 5 | GTPN | H6 | 31.3210432912 | 23.0203043104 | 33.4756654938 |
| 5 | GTPN | C2 | 32.2490000000 | 23.8120000000 | 36.6190000000 |
| 5 | GTPN | O2 | 33.1440000000 | 24.3320000000 | 37.2700000000 |
| 5 | GTPN | N3 | 31.1980000000 | 23.2880000000 | 37.2830000000 |
| 5 | GTPN | H3 | 31.1846717354 | 23.4118646296 | 38.2843873571 |
| 5 | GTPN | C4 | 30.1690000000 | 22.6150000000 | 36.7160000000 |
| 5 | GTPN | O4 | 29.3000000000 | 22.1860000000 | 37.4660000000 |
| 5 | GTPN | C5 | 30.2190000000 | 22.4970000000 | 35.2600000000 |
| 5 | GTPN | C5M | 29.1196657976 | 21.7387160282 | 34.5770141073 |
| 5 | GTPN | H51 | 29.2872192091 | 21.7297315895 | 33.4787581562 |
| 5 | GTPN | H52 | 28.1414872513 | 22.2236384615 | 34.7826133923 |
| 5 | GTPN | H53 | 29.1009959630 | 20.6894214336 | 34.9415499652 |
| 6 | GGPN | C | 39.6640000000 | 19.5420000000 | 38.2570000000 |
| 6 | GGPN | O1' | 40.3608069550 | 18.5456277501 | 38.0709827775 |
| 6 | GGPN | C2' | 39.1450000000 | 20.2470000000 | 37.0230000000 |
| 6 | GGPN | H2' | 38.7249519050 | 19.5105341045 | 36.3541560405 |
| 6 | GGPN | H2'' | 39.9660497965 | 20.7628063567 | 36.5476099564 |
| 6 | GGPN | N2' | 38.1030000000 | 21.2400000000 | 37.2800000000 |
| 6 | GGPN | C5' | 38.5816730989 | 23.7602014648 | 38.3895588510 |
| 6 | GGPN | H5' | 39.3909712225 | 23.8276783876 | 38.9471635029 |
| 6 | GGPN | H5'' | 37.8410588411 | 24.4043606905 | 38.3059589186 |
| 6 | GGPN | N | 37.7860000000 | 23.3040000000 | 35.1460000000 |
| 6 | GGPN | H1' | 37.5322299556 | 24.2644606033 | 35.1080787268 |
| 6 | GGPN | C6' | 38.5820000000 | 22.6230000000 | 37.3660000000 |
| 6 | GGPN | H6' | 37.9340811859 | 21.8347060630 | 37.7196236920 |
| 6 | GGPN | C6'' | 39.4620000000 | 24.6060000000 | 36.1920000000 |
| 6 | GGPN | H6'' | 38.6348978293 | 25.3215815376 | 35.9968699328 |
| 6 | GGPN | H6''' | 39.9125767517 | 24.2973491543 | 35.2245570161 |
| 6 | GGPN | O7' | 40.5291194571 | 25.2926021050 | 37.0468295817 |
| 6 | GGPN | C8' | 41.0278492652 | 26.4282564751 | 36.3658136273 |
| 6 | GGPN | H8' | 40.1863969757 | 27.1265380604 | 36.1698863794 |
| 6 | GGPN | H8'' | 41.4640758981 | 26.1023056771 | 35.3975734626 |
| 6 | GGPN | C9' | 42.1004323044 | 27.1477665533 | 37.1860397374 |
| 6 | GGPN | H9' | 42.9418845939 | 26.4494849679 | 37.3819669852 |
| 6 | GGPN | H9'' | 41.6642056715 | 27.4737173512 | 38.1542799020 |
| 6 | GGPN | O10' | 42.5991621126 | 28.2834209233 | 36.5050237829 |
| 6 | GGPN | C11' | 43.5860732444 | 28.9184156806 | 37.2956014680 |
| 6 | GGPN | H11' | 44.4080194927 | 28.1968859779 | 37.4900902017 |
| 6 | GGPN | H11'' | 43.1303405703 | 29.2211183612 | 38.2624031184 |
| 6 | GGPN | C12' | 44.1546077036 | 30.1548582070 | 36.5964030360 |
| 6 | GGPN | H12' | 43.3417269349 | 30.8870152134 | 36.4028180079 |
| 6 | GGPN | H12'' | 44.6194058573 | 29.8627828302 | 35.6305050911 |

| | | | | | |
|---|---|---|---|---|---|
| 6 | GGPN | O13' | 45.1328863826 | 30.7465497972 | 37.4301277437 |
| 6 | GGPN | H13' | 45.8083134430 | 30.0771669236 | 37.5617516571 |
| 6 | GGPN | C3' | 36.8300000000 | 20.8410000000 | 37.4650000000 |
| 6 | GGPN | O3' | 36.5260000000 | 19.6490000000 | 37.4420000000 |
| 6 | GGPN | C4' | 35.7550000000 | 21.8910000000 | 37.7310000000 |
| 6 | GGPN | H4' | 35.6461906162 | 22.5270481364 | 36.8268052214 |
| 6 | GGPN | H4'' | 36.0779186763 | 22.5236221087 | 38.5850946713 |
| 6 | GGPN | N9 | 34.4550000000 | 21.2750000000 | 38.0490000000 |
| 6 | GGPN | C4 | 33.9080000000 | 21.1390000000 | 39.2960000000 |
| 6 | GGPN | N2 | 34.1630000000 | 21.4230000000 | 42.6770000000 |
| 6 | GGPN | H21 | 35.0073815426 | 21.9372237273 | 42.8273122374 |
| 6 | GGPN | H22 | 33.6883815699 | 21.0822554423 | 43.4885605290 |
| 6 | GGPN | N3 | 34.4750000000 | 21.5070000000 | 40.4700000000 |
| 6 | GGPN | C2 | 33.7090000000 | 21.2100000000 | 41.4960000000 |
| 6 | GGPN | N1 | 32.5160000000 | 20.6420000000 | 41.4400000000 |
| 6 | GGPN | H1 | 32.0195155599 | 20.4442316526 | 42.2942243651 |
| 6 | GGPN | C6 | 31.9070000000 | 20.3020000000 | 40.2740000000 |
| 6 | GGPN | O6 | 30.7900000000 | 19.8160000000 | 40.3800000000 |
| 6 | GGPN | C5 | 32.6930000000 | 20.5410000000 | 39.1080000000 |
| 6 | GGPN | N7 | 32.4980000000 | 20.2290000000 | 37.7590000000 |
| 6 | GGPN | C8 | 33.5900000000 | 20.6550000000 | 37.1920000000 |
| 6 | GGPN | H8 | 33.8172988374 | 20.5399437128 | 36.1322300605 |
| 7 | GCPN | C | 39.0560000000 | 15.7400000000 | 42.8750000000 |
| 7 | GCPN | O1' | 39.5959483166 | 14.6417107077 | 42.9979489557 |
| 7 | GCPN | C2' | 39.2880000000 | 16.4700000000 | 41.5760000000 |
| 7 | GCPN | H2' | 39.1876651341 | 15.7684855831 | 40.7611392327 |
| 7 | GCPN | H2'' | 40.2814444956 | 16.8932000971 | 41.5885437031 |
| 7 | GCPN | N2' | 38.3500000000 | 17.5720000000 | 41.3180000000 |
| 7 | GCPN | C5' | 38.0915135455 | 19.8158311642 | 42.6418642246 |
| 7 | GCPN | H5' | 38.3644334424 | 19.7965021731 | 43.6014891095 |
| 7 | GCPN | H5'' | 37.5234285123 | 20.5170377245 | 42.2160384918 |
| 7 | GCPN | N | 39.1410000000 | 19.8150000000 | 39.4410000000 |
| 7 | GCPN | H1' | 38.7041654859 | 20.6902795758 | 39.4537367165 |
| 7 | GCPN | C6' | 38.8590000000 | 18.8910000000 | 41.6950000000 |
| 7 | GCPN | H6' | 38.3197483616 | 17.9598607338 | 41.6031132554 |
| 7 | GCPN | C6'' | 40.4230000000 | 20.7320000000 | 41.1900000000 |
| 7 | GCPN | H6'' | 39.8611072079 | 21.5952084675 | 40.7736127851 |
| 7 | GCPN | H6''' | 41.1722311010 | 20.3909354719 | 40.4439715650 |
| 7 | GCPN | O7' | 41.1469373516 | 21.1713678083 | 42.4642725925 |
| 7 | GCPN | C8' | 42.0409767971 | 22.2265885721 | 42.1652280982 |
| 7 | GCPN | H8' | 41.4638469395 | 23.0754741189 | 41.7405622645 |
| 7 | GCPN | H8'' | 42.7749708326 | 21.8712011233 | 41.4109210444 |
| 7 | GCPN | C9' | 42.7886675363 | 22.6988652692 | 43.4137525392 |
| 7 | GCPN | H9' | 43.3657973939 | 21.8499797224 | 43.8384183729 |
| 7 | GCPN | H9'' | 42.0546735008 | 23.0542527181 | 44.1680595930 |
| 7 | GCPN | O10' | 43.6827069818 | 23.7540860330 | 43.1147080449 |
| 7 | GCPN | C11' | 44.3522307416 | 24.1604294636 | 44.2932019785 |
| 7 | GCPN | H11' | 44.9088314610 | 23.2923539964 | 44.7063208923 |
| 7 | GCPN | H11'' | 43.5977075678 | 24.4966269921 | 45.0359621124 |
| 7 | GCPN | C12' | 45.3317345532 | 25.3026738192 | 44.0161638669 |
| 7 | GCPN | H12' | 44.7845538650 | 26.1794828916 | 43.6086060989 |
| 7 | GCPN | H12'' | 46.0956777581 | 24.9752098960 | 43.2789648788 |
| 7 | GCPN | O13' | 45.9696558468 | 25.6660872106 | 45.2257969229 |
| 7 | GCPN | H13' | 46.6792517238 | 26.2646586209 | 44.9812852502 |
| 7 | GCPN | C3' | 37.1250000000 | 17.3100000000 | 40.8420000000 |
| 7 | GCPN | O3' | 36.8040000000 | 16.1830000000 | 40.4960000000 |
| 7 | GCPN | C4' | 36.0960000000 | 18.4120000000 | 40.7830000000 |
| 7 | GCPN | H4' | 36.2635846429 | 19.0081767929 | 39.8607875917 |
| 7 | GCPN | H4'' | 36.2244168543 | 19.0684606242 | 41.6698797367 |
| 7 | GCPN | N1 | 34.7060000000 | 17.8690000000 | 40.7710000000 |
| 7 | GCPN | C6 | 34.1060000000 | 17.5860000000 | 39.5890000000 |
| 7 | GCPN | H6 | 34.6883407402 | 17.7631648964 | 38.6855245472 |
| 7 | GCPN | C5 | 32.8490000000 | 17.1110000000 | 39.5920000000 |
| 7 | GCPN | H5 | 32.3166465757 | 16.8747028034 | 38.6706621721 |
| 7 | GCPN | C2 | 34.0510000000 | 17.5490000000 | 41.9620000000 |
| 7 | GCPN | O2 | 34.6080000000 | 17.6660000000 | 43.0450000000 |
| 7 | GCPN | N3 | 32.7900000000 | 17.1030000000 | 41.9830000000 |
| 7 | GCPN | C4 | 32.2270000000 | 16.9230000000 | 40.8290000000 |
| 7 | GCPN | N4 | 31.0110000000 | 16.5340000000 | 40.8700000000 |
| 7 | GCPN | H41 | 30.5374323492 | 16.3902059469 | 41.7389401305 |
| 7 | GCPN | H42 | 30.4897714796 | 16.3595574490 | 40.0346015167 |
| 8 | GCPN | C | 36.6170000000 | 11.7170000000 | 46.2000000000 |
| 8 | GCPN | C2' | 37.4520000000 | 12.6630000000 | 45.4770000000 |

| | | | | | |
|---|---|---|---|---|---|
| 8 | GCPN | H2' | 38.0756679674 | 12.1194245812 | 44.7828706086 |
| 8 | GCPN | H2'' | 38.0607512371 | 13.2047132301 | 46.1856750237 |
| 8 | GCPN | N2' | 36.7080000000 | 13.6390000000 | 44.7140000000 |
| 8 | GCPN | C5' | 35.6193544854 | 15.8441825478 | 45.9357244686 |
| 8 | GCPN | H5' | 35.5327340491 | 15.7868735602 | 46.9178711133 |
| 8 | GCPN | H5'' | 35.2073570346 | 16.5061906612 | 45.3295868523 |
| 8 | GCPN | N | 38.0520000000 | 16.1050000000 | 43.6360000000 |
| 8 | GCPN | H1' | 37.7485401080 | 17.0419096355 | 43.5369811659 |
| 8 | GCPN | C6' | 36.7000000000 | 14.9580000000 | 45.3130000000 |
| 8 | GCPN | H6' | 36.2261181660 | 14.1150218379 | 44.8322925401 |
| 8 | GCPN | C6'' | 37.9660000000 | 17.0050000000 | 45.7730000000 |
| 8 | GCPN | H6'' | 37.6106177635 | 17.8208247758 | 45.1078954352 |
| 8 | GCPN | H6''' | 39.0461304029 | 16.8258630894 | 45.5846945015 |
| 8 | GCPN | O7' | 37.7733380234 | 17.4258696781 | 47.2313038356 |
| 8 | GCPN | C8' | 38.5075663444 | 18.6080107711 | 47.4875775345 |
| 8 | GCPN | H8' | 38.1466139515 | 19.4084191652 | 46.8070730253 |
| 8 | GCPN | H8'' | 39.5821265909 | 18.4134574788 | 47.2838720916 |
| 8 | GCPN | C9' | 38.3436535502 | 19.0665851627 | 48.9380057896 |
| 8 | GCPN | H9' | 38.7046059432 | 18.2661767686 | 49.6185102989 |
| 8 | GCPN | H9'' | 37.2690933037 | 19.2611384550 | 49.1417112325 |
| 8 | GCPN | O10' | 39.0778818713 | 20.2487262557 | 49.1942794886 |
| 8 | GCPN | C11' | 38.8997010236 | 20.6379619384 | 50.5429722515 |
| 8 | GCPN | H11' | 39.2533526721 | 19.8169218160 | 51.2024040095 |
| 8 | GCPN | H11'' | 37.8178400326 | 20.8118835024 | 50.7256049432 |
| 8 | GCPN | C12' | 39.6778532440 | 21.9152133803 | 50.8654747725 |
| 8 | GCPN | H12' | 39.3274152503 | 22.7456276888 | 50.2159291788 |
| 8 | GCPN | H12'' | 40.7629278898 | 21.7506660024 | 50.6927282452 |
| 8 | GCPN | O13' | 39.4630415337 | 22.2553877563 | 52.2220743166 |
| 8 | GCPN | H13' | 39.6843990445 | 23.1862878761 | 52.2997004684 |
| 8 | GCPN | C3' | 36.1120000000 | 13.2860000000 | 43.5840000000 |
| 8 | GCPN | O3' | 36.1190000000 | 12.1330000000 | 43.1840000000 |
| 8 | GCPN | C4' | 35.4270000000 | 14.3190000000 | 42.7520000000 |
| 8 | GCPN | H4' | 36.0159707536 | 14.4677211485 | 41.8221267390 |
| 8 | GCPN | H4'' | 35.4021256298 | 15.2705735208 | 43.3243267515 |
| 8 | GCPN | N1 | 34.0420000000 | 13.9430000000 | 42.3870000000 |
| 8 | GCPN | C6 | 33.7270000000 | 13.8320000000 | 41.0790000000 |
| 8 | GCPN | H6 | 34.5319527781 | 13.9915726147 | 40.3625155278 |
| 8 | GCPN | C5 | 32.4650000000 | 13.5370000000 | 40.7360000000 |
| 8 | GCPN | H5 | 32.1547045532 | 13.4288337626 | 39.6967177603 |
| 8 | GCPN | C2 | 33.0600000000 | 13.7180000000 | 43.3520000000 |
| 8 | GCPN | O2 | 33.3070000000 | 13.7660000000 | 44.5480000000 |
| 8 | GCPN | N3 | 31.7990000000 | 13.4370000000 | 43.0280000000 |
| 8 | GCPN | C4 | 31.5420000000 | 13.3690000000 | 41.7650000000 |
| 8 | GCPN | N4 | 30.3260000000 | 13.1150000000 | 41.4870000000 |
| 8 | GCPN | H41 | 29.6143410831 | 13.1347916457 | 42.1892463077 |
| 8 | GCPN | H42 | 30.0394302199 | 12.8866967679 | 40.5565402237 |
| 8 | GCPN | OT1 | 35.3665525611 | 11.8327642273 | 46.0971416204 |
| 8 | GCPN | OT2 | 37.1881586157 | 10.8316462796 | 46.8910330133 |
| 9 | GGPN | N | 24.7220000000 | 11.4840000000 | 50.9880000000 |
| 9 | GGPN | HT1 | 24.8531605171 | 10.4523662714 | 50.9766523687 |
| 9 | GGPN | HT2 | 24.6756379684 | 11.8383266104 | 50.0113204972 |
| 9 | GGPN | HT3 | 23.8369272764 | 11.7155102577 | 51.4826199658 |
| 9 | GGPN | C6' | 26.5140000000 | 12.4840000000 | 52.0930000000 |
| 9 | GGPN | H6' | 27.4296357108 | 12.2408147438 | 51.5754700771 |
| 9 | GGPN | C | 27.0960000000 | 15.7510000000 | 52.5570000000 |
| 9 | GGPN | O1' | 26.7300181023 | 16.9244357108 | 52.5122129116 |
| 9 | GGPN | C2' | 26.0340000000 | 14.7890000000 | 52.1530000000 |
| 9 | GGPN | H2' | 25.3114868093 | 15.3080036120 | 51.5408101231 |
| 9 | GGPN | H2'' | 25.5629231462 | 14.3994735468 | 53.0432371850 |
| 9 | GGPN | N2' | 26.5040000000 | 13.6540000000 | 51.3880000000 |
| 9 | GGPN | C5' | 26.1169367191 | 14.8679209925 | 52.0459770821 |
| 9 | GGPN | H5' | 26.8592210803 | 15.4526736084 | 52.3728091505 |
| 9 | GGPN | H5'' | 25.1704257410 | 15.1623586975 | 51.9149644805 |
| 9 | GGPN | C6'' | 25.1690000000 | 11.0500000000 | 53.0930000000 |
| 9 | GGPN | H6'' | 25.2038099694 | 10.0737894840 | 52.5638102266 |
| 9 | GGPN | H6''' | 24.1282021609 | 11.4380722885 | 53.0734992658 |
| 9 | GGPN | O7' | 25.6042008184 | 10.8502319480 | 54.5461321251 |
| 9 | GGPN | C8' | 24.7375054827 | 9.9296070247 | 55.1813596007 |
| 9 | GGPN | H8' | 24.7769554878 | 8.9640102980 | 54.6335519037 |
| 9 | GGPN | H8'' | 23.7013476793 | 10.3282931025 | 55.1432409430 |
| 9 | GGPN | C9' | 25.1363309323 | 9.6989727030 | 56.6403476303 |
| 9 | GGPN | H9' | 25.0968809273 | 10.6645694297 | 57.1881553273 |
| 9 | GGPN | H9'' | 26.1724887358 | 9.3002866252 | 56.6784662880 |

| | | | | | |
|---|---|---|---|---|---|
| 9 | GGPN | O10' | 24.2696355967 | 8.7783477796 | 57.2755751059 |
| 9 | GGPN | C11' | 24.6721252424 | 8.5935949734 | 58.6194848817 |
| 9 | GGPN | H11' | 24.6387701255 | 9.5737049940 | 59.1413070719 |
| 9 | GGPN | H11'' | 25.7143779340 | 8.2094221894 | 58.6316180327 |
| 9 | GGPN | C12' | 23.7605136546 | 7.6031706359 | 59.3467564573 |
| 9 | GGPN | H12' | 23.7912690385 | 6.6164955616 | 58.8370206498 |
| 9 | GGPN | H12'' | 22.7156612300 | 7.9807783661 | 59.3467096890 |
| 9 | GGPN | O13' | 24.2090612714 | 7.4583617900 | 60.6809455120 |
| 9 | GGPN | H13' | 23.6107015657 | 6.8320099018 | 61.0947679070 |
| 9 | GGPN | C3' | 26.8650000000 | 13.6780000000 | 50.1370000000 |
| 9 | GGPN | O3' | 26.8820000000 | 14.7420000000 | 49.6070000000 |
| 9 | GGPN | C4' | 27.2860000000 | 12.4290000000 | 49.3820000000 |
| 9 | GGPN | H4' | 26.6100255713 | 11.5951612057 | 49.6678590264 |
| 9 | GGPN | H4'' | 28.3265775178 | 12.1708423524 | 49.6726836849 |
| 9 | GGPN | N9 | 27.2260000000 | 12.6200000000 | 47.9390000000 |
| 9 | GGPN | C4 | 28.2880000000 | 12.8790000000 | 47.1350000000 |
| 9 | GGPN | N2 | 31.6000000000 | 13.3920000000 | 46.7200000000 |
| 9 | GGPN | H21 | 31.9729220103 | 13.3842072084 | 47.6478299664 |
| 9 | GGPN | H22 | 32.2461935229 | 13.5446790963 | 45.9722547061 |
| 9 | GGPN | N3 | 29.5690000000 | 13.0210000000 | 47.5270000000 |
| 9 | GGPN | C2 | 30.3550000000 | 13.2150000000 | 46.5000000000 |
| 9 | GGPN | N1 | 30.0010000000 | 13.2670000000 | 45.2330000000 |
| 9 | GGPN | H1 | 30.7008266428 | 13.4210266195 | 44.5249260709 |
| 9 | GGPN | C6 | 28.7240000000 | 13.1220000000 | 44.8230000000 |
| 9 | GGPN | O6 | 28.5350000000 | 13.1660000000 | 43.6210000000 |
| 9 | GGPN | C5 | 27.7930000000 | 12.9180000000 | 45.8690000000 |
| 9 | GGPN | N7 | 26.4170000000 | 12.7260000000 | 45.8650000000 |
| 9 | GGPN | C8 | 26.1390000000 | 12.5670000000 | 47.1230000000 |
| 9 | GGPN | H8 | 25.1350692286 | 12.4044352340 | 47.5150705176 |
| 10 | GGPN | C | 30.2900000000 | 20.2100000000 | 51.9720000000 |
| 10 | GGPN | O1' | 29.9241694848 | 21.3373033493 | 52.3010215688 |
| 10 | GGPN | C2' | 29.2520000000 | 19.1180000000 | 52.0680000000 |
| 10 | GGPN | H2' | 28.3280063104 | 19.4764256293 | 51.6390466120 |
| 10 | GGPN | H2'' | 29.1102790327 | 18.8619323102 | 53.1075089152 |
| 10 | GGPN | N2' | 29.6100000000 | 17.8890000000 | 51.3630000000 |
| 10 | GGPN | C5' | 31.6190666255 | 16.1476381773 | 52.2260279873 |
| 10 | GGPN | H5' | 32.3044039201 | 16.5518179375 | 52.8059818890 |
| 10 | GGPN | H5'' | 31.6322547175 | 15.2689069364 | 51.7821220353 |
| 10 | GGPN | N | 28.3590000000 | 15.4240000000 | 52.5360000000 |
| 10 | GGPN | H1' | 28.6137652229 | 14.4728440259 | 52.6761821831 |
| 10 | GGPN | C6' | 30.2780000000 | 16.8830000000 | 52.1850000000 |
| 10 | GGPN | H6' | 30.1642339440 | 17.3480768472 | 51.2170037980 |
| 10 | GGPN | C6'' | 30.1220000000 | 15.3020000000 | 54.0480000000 |
| 10 | GGPN | H6'' | 30.0183139882 | 14.2688795559 | 53.6528518761 |
| 10 | GGPN | H6''' | 29.2341949217 | 15.5449448127 | 54.6701017938 |
| 10 | GGPN | O7' | 31.3803320613 | 15.3845296021 | 54.9144232732 |
| 10 | GGPN | C8' | 31.2812957457 | 14.4679455360 | 55.9878712031 |
| 10 | GGPN | H8' | 31.1674914636 | 13.4431721214 | 55.5744529544 |
| 10 | GGPN | H8'' | 30.3833723971 | 14.7192373782 | 56.5917028721 |
| 10 | GGPN | C9' | 32.5212821001 | 14.5164404342 | 56.8828472283 |
| 10 | GGPN | H9' | 32.6350863823 | 15.5412138488 | 57.2962654770 |
| 10 | GGPN | H9'' | 33.4192054487 | 14.2651485920 | 56.2790155594 |
| 10 | GGPN | O10' | 32.4222457846 | 13.5998563682 | 57.9562951582 |
| 10 | GGPN | C11' | 33.5859973314 | 13.6761827648 | 58.7575951135 |
| 10 | GGPN | H11' | 33.6858222067 | 14.7119885227 | 59.1463984580 |
| 10 | GGPN | H11'' | 34.4699412732 | 13.4359232659 | 58.1291485403 |
| 10 | GGPN | C12' | 33.5221516916 | 12.6994948376 | 59.9335646824 |
| 10 | GGPN | H12' | 33.4289733178 | 11.6587696394 | 59.5560556867 |
| 10 | GGPN | H12'' | 32.6448542513 | 12.9348348962 | 60.5733056044 |
| 10 | GGPN | O13' | 34.7075595423 | 12.8194070231 | 60.6968826341 |
| 10 | GGPN | H13' | 34.6223168768 | 12.1918764915 | 61.4183666047 |
| 10 | GGPN | C3' | 29.4220000000 | 17.8010000000 | 50.0360000000 |
| 10 | GGPN | O3' | 28.9700000000 | 18.7460000000 | 49.3940000000 |
| 10 | GGPN | C4' | 29.8180000000 | 16.5190000000 | 49.3160000000 |
| 10 | GGPN | H4' | 29.3146760597 | 15.6613860574 | 49.8111197897 |
| 10 | GGPN | H4'' | 30.9186088801 | 16.3907261505 | 49.3946522863 |
| 10 | GGPN | N9 | 29.4350000000 | 16.5510000000 | 47.8970000000 |
| 10 | GGPN | C4 | 30.2730000000 | 16.6900000000 | 46.8270000000 |
| 10 | GGPN | N2 | 33.3710000000 | 17.2090000000 | 45.5440000000 |
| 10 | GGPN | H21 | 33.9436344918 | 17.3855859421 | 46.3445667641 |
| 10 | GGPN | H22 | 33.8137104516 | 17.2607647279 | 44.6488308154 |
| 10 | GGPN | N3 | 31.6110000000 | 16.8780000000 | 46.8660000000 |
| 10 | GGPN | C2 | 32.1260000000 | 16.9290000000 | 45.6600000000 |

| | | | | | |
|---|---|---|---|---|---|
| 10 | GGPN | N1 | 31.4690000000 | 16.8170000000 | 44.5210000000 |
| 10 | GGPN | H1 | 31.9702345325 | 16.8800904302 | 43.6492510460 |
| 10 | GGPN | C6 | 30.1320000000 | 16.6180000000 | 44.4550000000 |
| 10 | GGPN | O6 | 29.6590000000 | 16.5120000000 | 43.3350000000 |
| 10 | GGPN | C5 | 29.4820000000 | 16.5770000000 | 45.7190000000 |
| 10 | GGPN | N7 | 28.1400000000 | 16.4620000000 | 46.0770000000 |
| 10 | GGPN | C8 | 28.1770000000 | 16.4870000000 | 47.3760000000 |
| 10 | GGPN | H8 | 27.2944481278 | 16.4601914239 | 48.0150331839 |
| 11 | GCPN | C | 32.3330000000 | 24.6900000000 | 49.5040000000 |
| 11 | GCPN | O1' | 31.6848444234 | 25.7059691493 | 49.7501727773 |
| 11 | GCPN | C2' | 31.6140000000 | 23.4870000000 | 50.0280000000 |
| 11 | GCPN | H2' | 30.5516212049 | 23.6476046498 | 49.9201157708 |
| 11 | GCPN | H2'' | 31.8719289533 | 23.3588040323 | 51.0687305192 |
| 11 | GCPN | N2' | 31.9280000000 | 22.2220000000 | 49.3490000000 |
| 11 | GCPN | C5' | 34.0486172195 | 20.6108902538 | 49.2956131523 |
| 11 | GCPN | H5' | 34.9340612379 | 21.0558983494 | 49.2052799764 |
| 11 | GCPN | H5'' | 33.8607232801 | 19.6450482953 | 49.1471255223 |
| 11 | GCPN | N | 31.3640000000 | 20.0170000000 | 51.2290000000 |
| 11 | GCPN | H1' | 31.6207858044 | 19.0714427403 | 51.1149889759 |
| 11 | GCPN | C6' | 32.9570000000 | 21.4120000000 | 50.0080000000 |
| 11 | GCPN | H6' | 32.4028513010 | 21.9771544566 | 49.2733058486 |
| 11 | GCPN | C6'' | 33.6110000000 | 19.9750000000 | 51.9170000000 |
| 11 | GCPN | H6'' | 33.3737957938 | 18.9148840778 | 51.6843649274 |
| 11 | GCPN | H6''' | 33.0500796222 | 20.2830308295 | 52.8251387662 |
| 11 | GCPN | O7' | 35.1116313827 | 20.1069659020 | 52.1845639251 |
| 11 | GCPN | C8' | 35.4758167798 | 19.2831835328 | 53.2758826708 |
| 11 | GCPN | H8' | 35.2217708716 | 18.2300597834 | 53.0300826314 |
| 11 | GCPN | H8'' | 34.8980547000 | 19.5982065351 | 54.1708564703 |
| 11 | GCPN | C9' | 36.9719685692 | 19.3838821667 | 53.5796747186 |
| 11 | GCPN | H9' | 37.2260144773 | 20.4370059162 | 53.8254747580 |
| 11 | GCPN | H9'' | 37.5497306489 | 19.0688591645 | 52.6847009191 |
| 11 | GCPN | O10' | 37.3361539662 | 18.5600997976 | 54.6709934643 |
| 11 | GCPN | C11' | 38.7239927940 | 18.6821466939 | 54.9184463754 |
| 11 | GCPN | H11' | 38.9550351245 | 19.7444840296 | 55.1466687253 |
| 11 | GCPN | H11'' | 39.2787512961 | 18.3763372780 | 54.0058948865 |
| 11 | GCPN | C12' | 39.1632195577 | 17.8062739700 | 56.0935322538 |
| 11 | GCPN | H12' | 38.9429415684 | 16.7398472976 | 55.8732688908 |
| 11 | GCPN | H12'' | 38.6192253968 | 18.1079940492 | 57.0140427296 |
| 11 | GCPN | O13' | 40.5545396214 | 17.9682775954 | 56.2940523419 |
| 11 | GCPN | H13' | 40.7824733086 | 17.4034762393 | 57.0361073684 |
| 11 | GCPN | C3' | 31.3300000000 | 21.9180000000 | 48.1830000000 |
| 11 | GCPN | O3' | 30.6040000000 | 22.7210000000 | 47.6090000000 |
| 11 | GCPN | C4' | 31.5740000000 | 20.5640000000 | 47.5550000000 |
| 11 | GCPN | H4' | 31.2088374480 | 19.7822607181 | 48.2544191670 |
| 11 | GCPN | H4'' | 32.6675068562 | 20.4385548827 | 47.4061596712 |
| 11 | GCPN | N1 | 30.8830000000 | 20.3950000000 | 46.2360000000 |
| 11 | GCPN | C6 | 29.5360000000 | 20.2340000000 | 46.2000000000 |
| 11 | GCPN | H6 | 29.0098934738 | 20.2799604248 | 47.1528940222 |
| 11 | GCPN | C5 | 28.9290000000 | 20.0300000000 | 45.0160000000 |
| 11 | GCPN | H5 | 27.8530612540 | 19.8752524064 | 44.9353228592 |
| 11 | GCPN | C2 | 31.5930000000 | 20.4460000000 | 45.0350000000 |
| 11 | GCPN | O2 | 32.7900000000 | 20.7030000000 | 45.0130000000 |
| 11 | GCPN | N3 | 31.0090000000 | 20.2230000000 | 43.8490000000 |
| 11 | GCPN | C4 | 29.7280000000 | 20.0210000000 | 43.8670000000 |
| 11 | GCPN | N4 | 29.1960000000 | 19.7860000000 | 42.7250000000 |
| 11 | GCPN | H41 | 29.7416658687 | 19.7731293708 | 41.8870960039 |
| 11 | GCPN | H42 | 28.2165381316 | 19.6072502158 | 42.6317103275 |
| 12 | GAPN | C | 33.3580000000 | 28.9460000000 | 45.5530000000 |
| 12 | GAPN | O1' | 33.0457397844 | 30.1322037587 | 45.6441822382 |
| 12 | GAPN | C2' | 32.7120000000 | 28.0090000000 | 46.5460000000 |
| 12 | GAPN | H2' | 31.6495879109 | 28.2016459396 | 46.5653587167 |
| 12 | GAPN | H2'' | 33.1441925915 | 28.1831364200 | 47.5202139068 |
| 12 | GAPN | N2' | 32.8910000000 | 26.5740000000 | 46.2380000000 |
| 12 | GAPN | C5' | 35.0749751800 | 25.2295632591 | 45.8730618984 |
| 12 | GAPN | H5' | 35.8544339944 | 25.7381816612 | 45.5076017994 |
| 12 | GAPN | H5'' | 34.9888603421 | 24.2333750627 | 45.8712960951 |
| 12 | GAPN | N | 33.1350000000 | 24.6300000000 | 48.5200000000 |
| 12 | GAPN | H1' | 33.4947528936 | 23.7284423027 | 48.3918673262 |
| 12 | GAPN | C6' | 34.1230000000 | 25.9990000000 | 46.7910000000 |
| 12 | GAPN | H6' | 33.3194141875 | 26.4081032297 | 46.1967439728 |
| 12 | GAPN | C6'' | 35.3390000000 | 25.2900000000 | 48.8170000000 |
| 12 | GAPN | H6'' | 35.3773817961 | 24.1805350997 | 48.8599552520 |
| 12 | GAPN | H6''' | 34.8150680351 | 25.6716328194 | 49.7192651319 |

| | | | | | |
|---|---|---|---|---|---|
| 12 | GAPN | O7' | 36.7659571738 | 25.8415020023 | 48.7937886728 |
| 12 | GAPN | C8' | 37.4553781966 | 25.4300167738 | 49.9589511335 |
| 12 | GAPN | H8' | 37.4743480071 | 24.3197615370 | 49.9905750161 |
| 12 | GAPN | H8'' | 36.9120342460 | 25.8108592567 | 50.8498848959 |
| 12 | GAPN | C9' | 38.8904536057 | 25.9601997351 | 49.9780455793 |
| 12 | GAPN | H9' | 38.8714837952 | 27.0704549719 | 49.9464216968 |
| 12 | GAPN | H9'' | 39.4337975562 | 25.5793572522 | 49.0871118169 |
| 12 | GAPN | O10' | 39.5798746285 | 25.5487145066 | 51.1432080401 |
| 12 | GAPN | C11' | 40.8995768513 | 26.0587637440 | 51.1217413551 |
| 12 | GAPN | H11' | 40.8542278265 | 27.1677248635 | 51.0751010737 |
| 12 | GAPN | H11'' | 41.4165415875 | 25.6766271438 | 50.2157911938 |
| 12 | GAPN | C12' | 41.6838851614 | 25.6374597960 | 52.3660337075 |
| 12 | GAPN | H12' | 41.7414890891 | 24.5292457645 | 52.4193969645 |
| 12 | GAPN | H12'' | 41.1791753281 | 26.0203434842 | 53.2787068444 |
| 12 | GAPN | O13' | 42.9910007817 | 26.1740541021 | 52.2902689023 |
| 12 | GAPN | H13' | 43.4422234961 | 25.8851543360 | 53.0868459464 |
| 12 | GAPN | C3' | 32.0480000000 | 25.9470000000 | 45.4000000000 |
| 12 | GAPN | O3' | 31.1080000000 | 26.5270000000 | 44.8780000000 |
| 12 | GAPN | C4' | 32.3120000000 | 24.5190000000 | 45.0470000000 |
| 12 | GAPN | H4' | 32.3082742993 | 23.9117653576 | 45.9772377788 |
| 12 | GAPN | H4'' | 33.3115637402 | 24.4456457120 | 44.5678532641 |
| 12 | GAPN | N9 | 31.2800000000 | 23.9980000000 | 44.1150000000 |
| 12 | GAPN | C5 | 30.1770000000 | 23.4110000000 | 42.3180000000 |
| 12 | GAPN | N7 | 29.2990000000 | 23.2140000000 | 43.3900000000 |
| 12 | GAPN | C8 | 30.0040000000 | 23.5990000000 | 44.4170000000 |
| 12 | GAPN | H8 | 29.6355784126 | 23.6131486900 | 45.4426908238 |
| 12 | GAPN | N1 | 31.1060000000 | 23.5740000000 | 40.1240000000 |
| 12 | GAPN | C2 | 32.1880000000 | 24.0900000000 | 40.6800000000 |
| 12 | GAPN | H2 | 32.9864513963 | 24.3761810146 | 39.9971213932 |
| 12 | GAPN | N3 | 32.4190000000 | 24.3010000000 | 41.9640000000 |
| 12 | GAPN | C4 | 31.3660000000 | 23.9280000000 | 42.7390000000 |
| 12 | GAPN | C6 | 30.1070000000 | 23.2320000000 | 40.9240000000 |
| 12 | GAPN | N6 | 29.0780000000 | 22.7470000000 | 40.2770000000 |
| 12 | GAPN | H61 | 28.1979339820 | 22.6013771977 | 40.7289710205 |
| 12 | GAPN | H62 | 29.1364044139 | 22.5053478965 | 39.3083962702 |
| 13 | GTPN | C | 33.5270000000 | 32.2680000000 | 41.0180000000 |
| 13 | GTPN | O1' | 32.9034472295 | 33.3279252699 | 41.0433054249 |
| 13 | GTPN | C2' | 33.3050000000 | 31.3550000000 | 42.2010000000 |
| 13 | GTPN | H2' | 32.3407337899 | 31.5846273866 | 42.6291395928 |
| 13 | GTPN | H2'' | 34.0897134529 | 31.5364082648 | 42.9201365725 |
| 13 | GTPN | N2' | 33.3110000000 | 29.9070000000 | 41.9250000000 |
| 13 | GTPN | C5' | 35.0680963071 | 28.1081623715 | 41.3038606021 |
| 13 | GTPN | H5' | 35.7329245988 | 28.3597310807 | 40.6002861887 |
| 13 | GTPN | H5'' | 34.8688395358 | 27.1722638254 | 41.5948779185 |
| 13 | GTPN | N | 33.8430000000 | 28.4390000000 | 44.4350000000 |
| 13 | GTPN | H1' | 33.9541144304 | 27.4613379164 | 44.4574101761 |
| 13 | GTPN | C6' | 34.5830000000 | 29.2310000000 | 42.2230000000 |
| 13 | GTPN | H6' | 33.7461001039 | 29.7281128920 | 41.7553108159 |
| 13 | GTPN | C6'' | 36.1390000000 | 28.3080000000 | 43.9120000000 |
| 13 | GTPN | H6'' | 35.8471767332 | 27.2644853571 | 44.1572489589 |
| 13 | GTPN | H6''' | 36.0962986738 | 28.9247599362 | 44.8350462929 |
| 13 | GTPN | O7' | 37.5692899481 | 28.3151650936 | 43.3687511382 |
| 13 | GTPN | C8' | 38.4512875516 | 27.7860925502 | 44.3405471410 |
| 13 | GTPN | H8' | 38.1381162312 | 26.7477529775 | 44.5809660883 |
| 13 | GTPN | H8'' | 38.3872381718 | 28.4080275565 | 45.2587634223 |
| 13 | GTPN | C9' | 39.8966038134 | 27.7740872175 | 43.8387336545 |
| 13 | GTPN | H9' | 40.2097751338 | 28.8124267902 | 43.5983147072 |
| 13 | GTPN | H9'' | 39.9606531932 | 27.1521522112 | 42.9205173732 |
| 13 | GTPN | O10' | 40.7786014169 | 27.2450146741 | 44.8105296574 |
| 13 | GTPN | C11' | 42.1013859114 | 27.2516412149 | 44.3081132263 |
| 13 | GTPN | H11' | 42.3856090726 | 28.2968403544 | 44.0615314593 |
| 13 | GTPN | H11'' | 42.1364871320 | 26.6365657753 | 43.3837341253 |
| 13 | GTPN | C12' | 43.0915884775 | 26.6865250818 | 45.3284281945 |
| 13 | GTPN | H12' | 42.8207725716 | 25.6382538115 | 45.5776074035 |
| 13 | GTPN | H12'' | 43.0698945122 | 27.2985283906 | 46.2554047375 |
| 13 | GTPN | O13' | 44.3929092856 | 26.7177613339 | 44.7736188119 |
| 13 | GTPN | H13' | 44.9986475963 | 26.6676931760 | 45.5167031130 |
| 13 | GTPN | C3' | 32.1980000000 | 29.2940000000 | 41.4660000000 |
| 13 | GTPN | O3' | 31.1940000000 | 29.9320000000 | 41.1620000000 |
| 13 | GTPN | C4' | 32.1960000000 | 27.7790000000 | 41.3330000000 |
| 13 | GTPN | H4' | 32.3471801621 | 27.3396074180 | 42.3417860573 |
| 13 | GTPN | H4'' | 33.0420086259 | 27.4849074687 | 40.6762279088 |
| 13 | GTPN | N1 | 30.9330000000 | 27.2390000000 | 40.7590000000 |

| | | | | | |
|---|---|---|---|---|---|
| 13 | GTPN | C6 | 29.8980000000 | 26.9070000000 | 41.5960000000 |
| 13 | GTPN | H6 | 30.0707959320 | 27.0713607809 | 42.6590705725 |
| 13 | GTPN | C2 | 30.8150000000 | 27.0980000000 | 39.3710000000 |
| 13 | GTPN | O2 | 31.6780000000 | 27.4260000000 | 38.5690000000 |
| 13 | GTPN | N3 | 29.6740000000 | 26.5540000000 | 38.8980000000 |
| 13 | GTPN | H3 | 29.6218822174 | 26.4015446851 | 37.9018142255 |
| 13 | GTPN | C4 | 28.6040000000 | 26.2000000000 | 39.6470000000 |
| 13 | GTPN | O4 | 27.6380000000 | 25.7360000000 | 39.0550000000 |
| 13 | GTPN | C5 | 28.7460000000 | 26.4070000000 | 41.0870000000 |
| 13 | GTPN | C5M | 27.5853933335 | 26.0518624010 | 41.9684019806 |
| 13 | GTPN | H51 | 27.8344936517 | 26.2614635588 | 43.0306342463 |
| 13 | GTPN | H52 | 27.3496223325 | 24.9710393202 | 41.8659929786 |
| 13 | GTPN | H53 | 26.6973223709 | 26.6568507634 | 41.6863148532 |
| 14 | GGPN | C | 31.7940000000 | 35.3100000000 | 36.7810000000 |
| 14 | GGPN | O1' | 31.5220293533 | 36.5027645314 | 36.9084548542 |
| 14 | GGPN | C2' | 32.2280000000 | 34.5700000000 | 38.0240000000 |
| 14 | GGPN | H2' | 31.5171654098 | 34.7688079975 | 38.8122782407 |
| 14 | GGPN | H2'' | 33.2112746599 | 34.9147997210 | 38.3076848866 |
| 14 | GGPN | N2' | 32.3070000000 | 33.1210000000 | 37.8700000000 |
| 14 | GGPN | C5' | 34.3719988064 | 31.7790344643 | 36.5417139852 |
| 14 | GGPN | H5' | 34.8313904180 | 32.3005104608 | 35.8440778060 |
| 14 | GGPN | H5'' | 34.4286322814 | 30.8124838405 | 36.7212875005 |
| 14 | GGPN | N | 34.0110000000 | 31.7730000000 | 39.8940000000 |
| 14 | GGPN | H1' | 34.5946764976 | 30.9682963499 | 39.9304353169 |
| 14 | GGPN | C6' | 33.6530000000 | 32.6040000000 | 37.6110000000 |
| 14 | GGPN | H6' | 32.5912016741 | 32.5797336920 | 37.4154206100 |
| 14 | GGPN | C6'' | 35.8960000000 | 32.0780000000 | 38.4970000000 |
| 14 | GGPN | H6'' | 35.9387990533 | 31.0079694970 | 38.7926918067 |
| 14 | GGPN | H6''' | 36.1034528733 | 32.7103835747 | 39.3865420174 |
| 14 | GGPN | O7' | 36.9591680023 | 32.3473008521 | 37.4302053384 |
| 14 | GGPN | C8' | 38.2409317647 | 32.0431521584 | 37.9467324232 |
| 14 | GGPN | H8' | 38.2616628500 | 30.9737576830 | 38.2467195771 |
| 14 | GGPN | H8'' | 38.4263166700 | 32.6761717607 | 38.8405697878 |
| 14 | GGPN | C9' | 39.3378577120 | 32.2983192133 | 36.9110957880 |
| 14 | GGPN | H9' | 39.3171266268 | 33.3677136887 | 36.6111086340 |
| 14 | GGPN | H9'' | 39.1524728068 | 31.6652996110 | 36.0172584233 |
| 14 | GGPN | O10' | 40.6196214745 | 31.9941705196 | 37.4276228727 |
| 14 | GGPN | C11' | 41.6028781563 | 32.2432298044 | 36.4410121236 |
| 14 | GGPN | H11' | 41.5524079996 | 33.3133475880 | 36.1471970339 |
| 14 | GGPN | H11'' | 41.3877541796 | 31.6109335103 | 35.5533468232 |
| 14 | GGPN | C12' | 43.0080134979 | 31.9272080768 | 36.9573694993 |
| 14 | GGPN | H12' | 43.0721341483 | 30.8568477157 | 37.2480951929 |
| 14 | GGPN | H12'' | 43.2367879683 | 32.5592617934 | 37.8419454036 |
| 14 | GGPN | O13' | 43.9463052877 | 32.1940116711 | 35.9323576168 |
| 14 | GGPN | H13' | 44.8074028948 | 31.9824027000 | 36.3002333489 |
| 14 | GGPN | C3' | 31.1850000000 | 32.3810000000 | 37.9350000000 |
| 14 | GGPN | O3' | 30.0870000000 | 32.9050000000 | 38.1200000000 |
| 14 | GGPN | C4' | 31.2820000000 | 30.8700000000 | 37.7480000000 |
| 14 | GGPN | H4' | 31.8342256212 | 30.4388472505 | 38.6100384130 |
| 14 | GGPN | H4'' | 31.8450889699 | 30.6619019310 | 36.8133438807 |
| 14 | GGPN | N9 | 29.9530000000 | 30.2420000000 | 37.6620000000 |
| 14 | GGPN | C4 | 29.3300000000 | 29.8270000000 | 36.5170000000 |
| 14 | GGPN | N2 | 29.2280000000 | 29.6030000000 | 33.1190000000 |
| 14 | GGPN | H21 | 30.1229572949 | 29.9281543508 | 32.8135068112 |
| 14 | GGPN | H22 | 28.5576714581 | 29.3852767920 | 32.4095944385 |
| 14 | GGPN | N3 | 29.7950000000 | 29.9580000000 | 35.2500000000 |
| 14 | GGPN | C2 | 28.9520000000 | 29.4710000000 | 34.3660000000 |
| 14 | GGPN | N1 | 27.7910000000 | 28.8960000000 | 34.6330000000 |
| 14 | GGPN | H1 | 27.2159441304 | 28.5635293936 | 33.8752800548 |
| 14 | GGPN | C6 | 27.3170000000 | 28.7230000000 | 35.8950000000 |
| 14 | GGPN | O6 | 26.2400000000 | 28.1510000000 | 35.9840000000 |
| 14 | GGPN | C5 | 28.1500000000 | 29.2660000000 | 36.9180000000 |
| 14 | GGPN | N7 | 27.9840000000 | 29.3950000000 | 38.3010000000 |
| 14 | GGPN | C8 | 29.0680000000 | 30.0110000000 | 38.6770000000 |
| 14 | GGPN | H8 | 29.2742516699 | 30.3240093657 | 39.7004827862 |
| 15 | GCPN | C | 27.8000000000 | 36.8080000000 | 32.4760000000 |
| 15 | GCPN | O1' | 27.2884844198 | 37.9073418319 | 32.2693617950 |
| 15 | GCPN | C2' | 28.6730000000 | 36.6850000000 | 33.6990000000 |
| 15 | GCPN | H2' | 28.1682351212 | 37.1478095078 | 34.5339961643 |
| 15 | GCPN | H2'' | 29.6131463434 | 37.1799829194 | 33.5058275892 |
| 15 | GCPN | N2' | 28.9820000000 | 35.3030000000 | 34.0900000000 |
| 15 | GCPN | C5' | 30.3163253091 | 33.5397312307 | 32.7244460286 |
| 15 | GCPN | H5' | 30.3283514386 | 33.6633555755 | 31.7334684389 |

| | | | | | |
|---|---|---|---|---|---|
| 15 | GCPN | H5'' | 30.5531190116 | 32.6955655698 | 33.2027526968 |
| 15 | GCPN | N | 31.4770000000 | 34.6370000000 | 35.6890000000 |
| 15 | GCPN | H1' | 31.8539358244 | 33.7354614491 | 35.7004526879 |
| 15 | GCPN | C6' | 30.2540000000 | 34.8120000000 | 33.5720000000 |
| 15 | GCPN | H6' | 29.2215525288 | 35.0255654426 | 33.8058757304 |
| 15 | GCPN | C6'' | 32.7020000000 | 34.8990000000 | 33.7120000000 |
| 15 | GCPN | H6'' | 33.0838749023 | 33.9560779032 | 34.1584304641 |
| 15 | GCPN | H6''' | 33.0089701309 | 35.7548333639 | 34.3503958590 |
| 15 | GCPN | O7' | 33.3026619574 | 35.0730342039 | 32.3156414111 |
| 15 | GCPN | C8' | 34.7139079725 | 35.1221390153 | 32.4061864415 |
| 15 | GCPN | H8' | 35.0765123451 | 34.1771957124 | 32.8640366765 |
| 15 | GCPN | H8'' | 35.0016075737 | 35.9759511731 | 33.0560020714 |
| 15 | GCPN | C9' | 35.3584288272 | 35.2959014942 | 31.0294876841 |
| 15 | GCPN | H9' | 34.9958244546 | 36.2408447971 | 30.5716374490 |
| 15 | GCPN | H9'' | 35.0707292260 | 34.4420893364 | 30.3796720541 |
| 15 | GCPN | O10' | 36.7696748423 | 35.3450063056 | 31.1200327144 |
| 15 | GCPN | C11' | 37.3251890055 | 35.5059595072 | 29.8286291829 |
| 15 | GCPN | H11' | 36.9367616335 | 36.4481448084 | 29.3865458770 |
| 15 | GCPN | H11'' | 37.0116664049 | 34.6493893477 | 29.1945804821 |
| 15 | GCPN | C12' | 38.8532553868 | 35.5642382110 | 29.8787961747 |
| 15 | GCPN | H12' | 39.2534380280 | 34.6233417123 | 30.3133900331 |
| 15 | GCPN | H12'' | 39.1785332566 | 36.4220971730 | 30.5053554280 |
| 15 | GCPN | O13' | 39.3515322325 | 35.7252769072 | 28.5642575775 |
| 15 | GCPN | H13' | 38.7165440355 | 35.2961068592 | 27.9861545940 |
| 15 | GCPN | C3' | 28.0970000000 | 34.5910000000 | 34.7940000000 |
| 15 | GCPN | O3' | 27.0650000000 | 35.0940000000 | 35.2130000000 |
| 15 | GCPN | C4' | 28.3400000000 | 33.1220000000 | 35.0320000000 |
| 15 | GCPN | H4' | 28.9734201098 | 33.0066373634 | 35.9372427623 |
| 15 | GCPN | H4'' | 28.8772991164 | 32.7020073799 | 34.1551249287 |
| 15 | GCPN | N1 | 27.0640000000 | 32.3780000000 | 35.2280000000 |
| 15 | GCPN | C6 | 26.6010000000 | 32.1610000000 | 36.4810000000 |
| 15 | GCPN | H6 | 27.1927658349 | 32.5629130844 | 37.3025826272 |
| 15 | GCPN | C5 | 25.4570000000 | 31.4740000000 | 36.6430000000 |
| 15 | GCPN | H5 | 25.0418036403 | 31.2641696790 | 37.6287399163 |
| 15 | GCPN | C2 | 26.2940000000 | 31.9800000000 | 34.1340000000 |
| 15 | GCPN | O2 | 26.6200000000 | 32.2650000000 | 32.9900000000 |
| 15 | GCPN | N3 | 25.1680000000 | 31.2720000000 | 34.2790000000 |
| 15 | GCPN | C4 | 24.7990000000 | 31.0260000000 | 35.4960000000 |
| 15 | GCPN | N4 | 23.7390000000 | 30.3230000000 | 35.6130000000 |
| 15 | GCPN | H41 | 23.2440579288 | 29.9866044824 | 34.8118318517 |
| 15 | GCPN | H42 | 23.3667252567 | 30.0842507811 | 36.5098892496 |
| 16 | GCPN | C | 22.6900000000 | 37.1630000000 | 29.8140000000 |
| 16 | GCPN | C2' | 24.0720000000 | 37.2460000000 | 30.3130000000 |
| 16 | GCPN | H2' | 24.1796049958 | 38.1512519576 | 30.8919352979 |
| 16 | GCPN | H2'' | 24.7468321586 | 37.2556277745 | 29.4699490617 |
| 16 | GCPN | N2' | 24.4650000000 | 36.1390000000 | 31.1670000000 |
| 16 | GCPN | C5' | 25.2975907012 | 33.7441913879 | 30.1180641595 |
| 16 | GCPN | H5' | 25.0475142881 | 33.5818441363 | 29.1761512336 |
| 16 | GCPN | H5'' | 25.6444592709 | 33.0902315244 | 30.7723679841 |
| 16 | GCPN | N | 27.3410000000 | 35.7160000000 | 31.9130000000 |
| 16 | GCPN | H1' | 27.8766812331 | 34.8959228210 | 32.0543587972 |
| 16 | GCPN | C6' | 25.3870000000 | 35.2130000000 | 30.5370000000 |
| 16 | GCPN | H6' | 24.5076042288 | 35.4668083483 | 31.1101494014 |
| 16 | GCPN | C6'' | 27.6990000000 | 34.7880000000 | 29.8110000000 |
| 16 | GCPN | H6'' | 28.1879639421 | 34.0829148003 | 30.5166910127 |
| 16 | GCPN | H6''' | 28.2672035394 | 35.7425640688 | 29.7974775278 |
| 16 | GCPN | O7' | 27.7058572697 | 34.1793682215 | 28.4072833847 |
| 16 | GCPN | C8' | 29.0388860116 | 33.9385020137 | 27.9983300337 |
| 16 | GCPN | H8' | 29.5145913524 | 33.2411698840 | 28.7204511758 |
| 16 | GCPN | H8'' | 29.5938309497 | 34.9008191525 | 28.0012376909 |
| 16 | GCPN | C9' | 29.0937518262 | 33.3283263897 | 26.5963402155 |
| 16 | GCPN | H9' | 28.6180464854 | 34.0256585195 | 25.8742190734 |
| 16 | GCPN | H9'' | 28.5388068881 | 32.3660092510 | 26.5934325583 |
| 16 | GCPN | O10' | 30.4267805681 | 33.0874601820 | 26.1873868645 |
| 16 | GCPN | C11' | 30.4331224221 | 32.5245752365 | 24.8891783608 |
| 16 | GCPN | H11' | 29.9398315279 | 33.2324603893 | 24.1894717662 |
| 16 | GCPN | H11'' | 29.8605919306 | 31.5728111208 | 24.9086852511 |
| 16 | GCPN | C12' | 31.8577961532 | 32.2468848584 | 24.4053469709 |
| 16 | GCPN | H12' | 32.3589705698 | 31.5340856776 | 25.0945825775 |
| 16 | GCPN | H12'' | 32.4382101672 | 33.1937349461 | 24.3753690925 |
| 16 | GCPN | O13' | 31.8024703000 | 31.6869041184 | 23.1070459193 |
| 16 | GCPN | H13' | 32.6923360501 | 31.3846843208 | 22.9110812778 |
| 16 | GCPN | C3' | 23.9770000000 | 36.0400000000 | 32.4010000000 |

| | | | | | |
|---|---|---|---|---|---|
| 16 | GCPN | O3' | 23.1600000000 | 36.8340000000 | 32.8400000000 |
| 16 | GCPN | C4' | 24.4390000000 | 34.9400000000 | 33.3040000000 |
| 16 | GCPN | H4' | 24.9822825415 | 35.3934185897 | 34.1601160128 |
| 16 | GCPN | H4'' | 25.1345454115 | 34.2873482787 | 32.7348416074 |
| 16 | GCPN | N1 | 23.3250000000 | 34.1140000000 | 33.8260000000 |
| 16 | GCPN | C6 | 23.1510000000 | 34.0350000000 | 35.1640000000 |
| 16 | GCPN | H6 | 23.8279349296 | 34.6218143408 | 35.7838845060 |
| 16 | GCPN | C5 | 22.1760000000 | 33.2560000000 | 35.6510000000 |
| 16 | GCPN | H5 | 21.9881279322 | 33.1576026198 | 36.7201612823 |
| 16 | GCPN | C2 | 22.4650000000 | 33.4130000000 | 32.9810000000 |
| 16 | GCPN | O2 | 22.5540000000 | 33.4860000000 | 31.7650000000 |
| 16 | GCPN | N3 | 21.4980000000 | 32.6270000000 | 33.4490000000 |
| 16 | GCPN | C4 | 21.3930000000 | 32.5610000000 | 34.7330000000 |
| 16 | GCPN | N4 | 20.4630000000 | 31.7980000000 | 35.1500000000 |
| 16 | GCPN | H41 | 19.8837222352 | 31.2856969579 | 34.5159788140 |
| 16 | GCPN | H42 | 20.2789480569 | 31.6840443678 | 36.1262883776 |
| 16 | GCPN | OT1 | 21.9943329052 | 36.1714275372 | 30.1610321948 |
| 16 | GCPN | OT2 | 22.2746843944 | 38.0878491892 | 29.0658242686 |

Model 2

| | | | | | |
|---|---|---|---|---|---|
| 1 | GGPN | N | 14.2150869671 | 28.5958399629 | 25.9489056947 |
| 1 | GGPN | HT1 | 14.7516626156 | 28.8895428742 | 26.7859165630 |
| 1 | GGPN | HT2 | 13.2529928876 | 28.3123982204 | 26.2162881376 |
| 1 | GGPN | HT3 | 14.1346837010 | 29.4312016093 | 25.3254756521 |
| 1 | GGPN | C6' | 15.0531246520 | 27.4745602884 | 25.3564854171 |
| 1 | GGPN | H6' | 14.9146018328 | 26.5949432596 | 25.9799292912 |
| 1 | GGPN | C | 19.2427034160 | 26.2466430962 | 25.3263173657 |
| 1 | GGPN | O1' | 19.7169142847 | 25.3314489473 | 24.6763762768 |
| 1 | GGPN | C2' | 17.9786869350 | 26.0601730263 | 26.1191412930 |
| 1 | GGPN | H2' | 18.1904686426 | 25.4652931191 | 26.9997874976 |
| 1 | GGPN | H2'' | 17.2786067624 | 25.5622056344 | 25.4747910431 |
| 1 | GGPN | N2' | 17.3716261236 | 27.3288293539 | 26.4978026304 |
| 1 | GGPN | C5' | 16.5310369015 | 27.9660144500 | 25.4851248058 |
| 1 | GGPN | H5' | 16.5549101818 | 29.0373558980 | 25.6451236628 |
| 1 | GGPN | H5'' | 17.0114539193 | 27.7238358461 | 24.5444339196 |
| 1 | GGPN | C6'' | 14.6254193931 | 27.1855042629 | 23.8682866732 |
| 1 | GGPN | H6'' | 13.5833595868 | 26.8480595681 | 23.7200538993 |
| 1 | GGPN | H6''' | 14.7255931584 | 28.1978943169 | 23.5214445532 |
| 1 | GGPN | O7' | 15.5741901620 | 26.3272244488 | 22.9765765711 |
| 1 | GGPN | C8' | 15.7603772186 | 26.8776462355 | 21.6433068636 |
| 1 | GGPN | H8' | 16.7902131057 | 26.6088288636 | 21.3075220218 |
| 1 | GGPN | H8'' | 15.7388786002 | 27.9766331983 | 21.7144301482 |
| 1 | GGPN | C9' | 14.7793220866 | 26.5401156283 | 20.4520122969 |
| 1 | GGPN | H9' | 13.7382720641 | 26.5664684252 | 20.8358908565 |
| 1 | GGPN | H9'' | 15.0104861474 | 25.4968786591 | 20.1446496905 |
| 1 | GGPN | O10' | 14.8538693938 | 27.3984571396 | 19.2836864257 |
| 1 | GGPN | C11' | 14.0393535428 | 26.9106035865 | 18.1868623819 |
| 1 | GGPN | H11' | 12.9657812844 | 26.9386665127 | 18.4797699604 |
| 1 | GGPN | H11'' | 14.3087032826 | 25.8472281161 | 18.0013554299 |
| 1 | GGPN | C12' | 14.1169234939 | 27.6044508330 | 16.7851822312 |
| 1 | GGPN | H12' | 15.1703406979 | 27.6575956263 | 16.4366779268 |
| 1 | GGPN | H12'' | 13.7081609571 | 28.6372108656 | 16.8290438383 |
| 1 | GGPN | O13' | 13.3546930160 | 26.8413764519 | 15.8445145626 |
| 1 | GGPN | H13' | 13.4691217555 | 27.2174407727 | 14.9627086510 |
| 1 | GGPN | C3' | 17.7333116721 | 27.8209038118 | 27.7126184122 |
| 1 | GGPN | O3' | 18.5522620113 | 27.2273757628 | 28.4150811247 |
| 1 | GGPN | C4' | 17.1127659916 | 29.1115680756 | 28.2070710008 |
| 1 | GGPN | H4' | 16.0271858378 | 29.0904049308 | 28.0312591879 |
| 1 | GGPN | H4'' | 17.5774040704 | 29.9585601189 | 27.6537432818 |
| 1 | GGPN | N9 | 17.3624119501 | 29.2111376291 | 29.6403999545 |
| 1 | GGPN | C4 | 18.3522521948 | 29.9535887891 | 30.2137093752 |
| 1 | GGPN | N2 | 21.0002087514 | 32.1762295074 | 29.9427227320 |
| 1 | GGPN | H21 | 21.0480189087 | 32.3890643426 | 28.9751228193 |
| 1 | GGPN | H22 | 21.5993932588 | 32.6864712954 | 30.5700514614 |
| 1 | GGPN | N3 | 19.2423970796 | 30.7190421382 | 29.5515485667 |
| 1 | GGPN | C2 | 20.0686189168 | 31.3493403691 | 30.3915205180 |
| 1 | GGPN | N1 | 20.0077438932 | 31.1920112760 | 31.7515992424 |
| 1 | GGPN | H1 | 20.6610287060 | 31.7050954955 | 32.3261547389 |
| 1 | GGPN | C6 | 19.1085909103 | 30.3904346739 | 32.4527658527 |
| 1 | GGPN | O6 | 19.1083253775 | 30.3051748513 | 33.6809458528 |
| 1 | GGPN | C5 | 18.2181746287 | 29.7424001456 | 31.5692637242 |
| 1 | GGPN | N7 | 17.1748155181 | 28.8680597156 | 31.8407388329 |
| 1 | GGPN | C8 | 16.7109325450 | 28.5591585026 | 30.6679430707 |
| 1 | GGPN | H8 | 15.9043995237 | 27.8464071408 | 30.4988791498 |
| 2 | GGPN | C | 24.9326447557 | 26.4161772802 | 25.1419514168 |
| 2 | GGPN | O1' | 25.5553801692 | 25.3993181243 | 24.9667892739 |
| 2 | GGPN | C2' | 23.4147879364 | 26.4503134004 | 25.2093668879 |
| 2 | GGPN | H2' | 23.1574534961 | 25.5310847491 | 25.7524145519 |
| 2 | GGPN | H2'' | 23.0996845174 | 26.3913328025 | 24.1895918780 |
| 2 | GGPN | N2' | 22.7147520251 | 27.5843625793 | 25.8427717398 |
| 2 | GGPN | C5' | 21.9133827846 | 28.5540753861 | 24.9788149489 |
| 2 | GGPN | H5' | 22.5941423064 | 28.9744301468 | 24.2905497073 |
| 2 | GGPN | H5'' | 21.5003980483 | 29.3314869513 | 25.5893849530 |
| 2 | GGPN | N | 19.7960569392 | 27.4805376934 | 25.3185406628 |
| 2 | GGPN | H1' | 19.5816724350 | 28.1493070304 | 26.0262161086 |
| 2 | GGPN | C6' | 20.6852828799 | 27.9264648127 | 24.2980600648 |
| 2 | GGPN | H6' | 21.1208131309 | 27.0623485970 | 23.8058646119 |
| 2 | GGPN | C6'' | 19.9296975935 | 28.7506746877 | 23.1204495934 |
| 2 | GGPN | H6'' | 19.2255726891 | 27.9741233754 | 22.7502466230 |
| 2 | GGPN | H6''' | 20.6175255896 | 28.9746279433 | 22.2885626340 |
| 2 | GGPN | O7' | 19.0249478038 | 30.0197587146 | 23.4696866627 |

| | | | | | |
|---|---|---|---|---|---|
| 2 | GGPN | C8' | 18.0882011003 | 30.3730323723 | 22.3883062480 |
| 2 | GGPN | H8' | 18.1887755348 | 29.6232352821 | 21.5695540950 |
| 2 | GGPN | H8'' | 18.3451917182 | 31.3580296991 | 21.9342917438 |
| 2 | GGPN | C9' | 16.5739419025 | 30.4442456954 | 22.8170873012 |
| 2 | GGPN | H9' | 16.5012662623 | 31.0617941371 | 23.7390936529 |
| 2 | GGPN | H9'' | 16.3686667145 | 29.4003877034 | 23.1264491323 |
| 2 | GGPN | O10' | 15.5372253432 | 30.8973270342 | 21.9020263291 |
| 2 | GGPN | C11' | 14.2272398645 | 30.4870889909 | 22.3526092589 |
| 2 | GGPN | H11' | 14.0132506360 | 30.8312618233 | 23.3907637283 |
| 2 | GGPN | H11'' | 14.3753929755 | 29.4214615289 | 22.3018747074 |
| 2 | GGPN | C12' | 12.9017671449 | 30.3195031851 | 21.5440311965 |
| 2 | GGPN | H12' | 13.1582851731 | 29.7796829753 | 20.6027808294 |
| 2 | GGPN | H12'' | 12.4199021149 | 31.2883997035 | 21.3094486717 |
| 2 | GGPN | O13' | 12.0152773067 | 29.4796458090 | 22.3194206676 |
| 2 | GGPN | H13' | 11.2861659030 | 29.2043141471 | 21.7436992268 |
| 2 | GGPN | C3' | 22.7019588791 | 27.6423640109 | 27.1719493318 |
| 2 | GGPN | O3' | 23.3123952516 | 26.8106073264 | 27.8585825361 |
| 2 | GGPN | C4' | 21.9228502920 | 28.7576998481 | 27.8949629847 |
| 2 | GGPN | H4' | 20.8606910188 | 28.7529826600 | 27.5850733558 |
| 2 | GGPN | H4'' | 22.3760728441 | 29.7373518857 | 27.6244970639 |
| 2 | GGPN | N9 | 21.9420834350 | 28.5575525134 | 29.3258583397 |
| 2 | GGPN | C4 | 22.7568143000 | 29.2098193484 | 30.2004128924 |
| 2 | GGPN | N2 | 25.3317898624 | 31.4394463895 | 30.7996638601 |
| 2 | GGPN | H21 | 25.6117327229 | 31.6882740034 | 29.8842993459 |
| 2 | GGPN | H22 | 25.8409690488 | 31.7587167912 | 31.6058231201 |
| 2 | GGPN | N3 | 23.6967274113 | 30.1199272436 | 29.8606932870 |
| 2 | GGPN | C2 | 24.3045611438 | 30.6122991097 | 30.9344067470 |
| 2 | GGPN | N1 | 23.9817032709 | 30.2419562774 | 32.2071360699 |
| 2 | GGPN | H1 | 24.4309244658 | 30.7318880473 | 32.9712346074 |
| 2 | GGPN | C6 | 23.0017011726 | 29.3268826287 | 32.5844573104 |
| 2 | GGPN | O6 | 22.7739849030 | 29.0528535352 | 33.7575001492 |
| 2 | GGPN | C5 | 22.4021930445 | 28.7474377224 | 31.4419250776 |
| 2 | GGPN | N7 | 21.4323500748 | 27.7581525119 | 31.3526882673 |
| 2 | GGPN | C8 | 21.1942397056 | 27.6705964682 | 30.0705524364 |
| 2 | GGPN | H8 | 20.5074300869 | 26.9683561499 | 29.5941169269 |
| 3 | GCPN | C | 29.9488518693 | 25.4181000774 | 26.6480883769 |
| 3 | GCPN | O1' | 30.6063766505 | 24.4664874020 | 26.2453847488 |
| 3 | GCPN | C2' | 28.5360728773 | 25.6147427022 | 26.2719993935 |
| 3 | GCPN | H2' | 27.9915655532 | 24.7054789792 | 26.5074693826 |
| 3 | GCPN | H2'' | 28.5967286593 | 25.7826723045 | 25.2125054667 |
| 3 | GCPN | N2' | 27.9179059277 | 26.8006184985 | 26.8901459260 |
| 3 | GCPN | C5' | 27.8393307647 | 28.0111740700 | 26.0491567005 |
| 3 | GCPN | H5' | 28.8426021761 | 28.1571616105 | 25.6818977737 |
| 3 | GCPN | H5'' | 27.5338842655 | 28.8529318023 | 26.6461819118 |
| 3 | GCPN | N | 25.5542854334 | 27.6135893490 | 25.2357433214 |
| 3 | GCPN | H1' | 25.0151079504 | 28.4419086565 | 25.3571305875 |
| 3 | GCPN | C6' | 26.8813447052 | 27.8950254208 | 24.8145694867 |
| 3 | GCPN | H6' | 27.2532336155 | 27.0915244819 | 24.1846322354 |
| 3 | GCPN | C6'' | 26.7678924163 | 29.1720356157 | 23.9128277412 |
| 3 | GCPN | H6'' | 27.3999849136 | 29.1499436026 | 22.9984513134 |
| 3 | GCPN | H6''' | 27.1320911690 | 30.0181814641 | 24.5380322972 |
| 3 | GCPN | O7' | 25.2207904440 | 29.4000585355 | 23.5666321107 |
| 3 | GCPN | C8' | 24.8971185654 | 29.4760154918 | 22.1646047989 |
| 3 | GCPN | H8' | 25.7879601255 | 29.1938398960 | 21.5652606653 |
| 3 | GCPN | H8'' | 24.6478435579 | 30.5328293288 | 21.9351727559 |
| 3 | GCPN | C9' | 23.6909386688 | 28.6590933051 | 21.5653052799 |
| 3 | GCPN | H9' | 23.3941622294 | 27.8400761634 | 22.2461500311 |
| 3 | GCPN | H9'' | 24.0994848662 | 28.1649808652 | 20.6632461995 |
| 3 | GCPN | O10' | 22.5260401704 | 29.3781221688 | 21.1568517925 |
| 3 | GCPN | C11' | 22.0784976997 | 28.9937372484 | 19.8452025854 |
| 3 | GCPN | H11' | 22.8073544093 | 29.3800265832 | 19.1145432193 |
| 3 | GCPN | H11'' | 22.3409301483 | 27.9419685300 | 19.5805424194 |
| 3 | GCPN | C12' | 20.5951202132 | 29.5060602730 | 19.6449926761 |
| 3 | GCPN | H12' | 20.5217984376 | 29.9349144025 | 18.6265315491 |
| 3 | GCPN | H12'' | 20.2189936609 | 30.3280643467 | 20.3268745523 |
| 3 | GCPN | O13' | 19.7049595215 | 28.3953677691 | 19.7734989467 |
| 3 | GCPN | H13' | 18.8911879605 | 28.5619131449 | 19.2820224626 |
| 3 | GCPN | C3' | 27.3709906729 | 26.7011833185 | 28.1405994695 |
| 3 | GCPN | O3' | 27.5973122639 | 25.7167208476 | 28.8567383156 |
| 3 | GCPN | C4' | 26.4608213271 | 27.8090303610 | 28.7216765973 |
| 3 | GCPN | H4' | 25.5667120004 | 27.8467301717 | 28.0644533105 |
| 3 | GCPN | H4'' | 26.9711347007 | 28.7892000412 | 28.6465188202 |
| 3 | GCPN | N1 | 26.0451608811 | 27.5239670652 | 30.1080644324 |

| | | | | | |
|---|---|---|---|---|---|
| 3 | GCPN | C6 | 25.0793480126 | 26.5928888738 | 30.3623913541 |
| 3 | GCPN | H6 | 24.6213708244 | 26.1452939184 | 29.4800274865 |
| 3 | GCPN | C5 | 24.7244026924 | 26.2688950496 | 31.6112127826 |
| 3 | GCPN | H5 | 23.9557695155 | 25.5278444262 | 31.8140959560 |
| 3 | GCPN | C2 | 26.7657817071 | 28.0854151106 | 31.1717958880 |
| 3 | GCPN | O2 | 27.7433419910 | 28.8107437776 | 30.9333909664 |
| 3 | GCPN | N3 | 26.3830450919 | 27.8120840256 | 32.4424855276 |
| 3 | GCPN | C4 | 25.3942231245 | 26.9283180942 | 32.6830717742 |
| 3 | GCPN | N4 | 25.0296766231 | 26.6699984333 | 33.9418782857 |
| 3 | GCPN | H41 | 25.4134054431 | 27.2275463278 | 34.6842917369 |
| 3 | GCPN | H42 | 24.1813929242 | 26.1844212276 | 34.1259649268 |
| 4 | GAPN | C | 34.0420213923 | 23.8070311379 | 30.0138674928 |
| 4 | GAPN | O1' | 34.9478073937 | 23.1065033993 | 29.5774124652 |
| 4 | GAPN | C2' | 32.8800257967 | 24.1723892549 | 29.1833974254 |
| 4 | GAPN | H2' | 32.2566955069 | 23.3002548482 | 29.0712649947 |
| 4 | GAPN | H2'' | 33.3538046946 | 24.4601910437 | 28.2610240623 |
| 4 | GAPN | N2' | 32.0782301322 | 25.3083990008 | 29.6823447437 |
| 4 | GAPN | C5' | 32.2229724448 | 26.6107317899 | 29.0233737307 |
| 4 | GAPN | H5' | 33.2657749393 | 26.8632501668 | 29.0461351330 |
| 4 | GAPN | H5'' | 31.6050813053 | 27.3675932109 | 29.4884935417 |
| 4 | GAPN | N | 30.4622680866 | 26.3804509234 | 27.4099395572 |
| 4 | GAPN | H1' | 29.8302854845 | 27.0149165865 | 27.8427057579 |
| 4 | GAPN | C6' | 31.8662680200 | 26.5849529726 | 27.5559951733 |
| 4 | GAPN | H6' | 32.4001539138 | 25.7452469336 | 27.1370353105 |
| 4 | GAPN | C6'' | 32.4190845419 | 27.7997812918 | 26.7851616642 |
| 4 | GAPN | H6'' | 33.1450647956 | 28.3437317984 | 27.4210622750 |
| 4 | GAPN | H6''' | 31.5986109924 | 28.4880216834 | 26.4922800554 |
| 4 | GAPN | O7' | 33.1613858090 | 27.2876187140 | 25.5539778786 |
| 4 | GAPN | C8' | 32.7545949066 | 27.9082613938 | 24.3476205563 |
| 4 | GAPN | H8' | 33.2146915114 | 28.9173765777 | 24.2514008148 |
| 4 | GAPN | H8'' | 31.6474013947 | 27.9949654118 | 24.2732066498 |
| 4 | GAPN | C9' | 33.2751073126 | 26.9806207877 | 23.2576338841 |
| 4 | GAPN | H9' | 32.6791963674 | 26.0396940710 | 23.2670230855 |
| 4 | GAPN | H9'' | 34.3349594017 | 26.7257390543 | 23.4898400918 |
| 4 | GAPN | O10' | 33.2057590959 | 27.5954503376 | 21.9872080091 |
| 4 | GAPN | C11' | 33.7922318838 | 26.7582534296 | 21.0023288870 |
| 4 | GAPN | H11' | 33.2323584644 | 25.7986617199 | 20.9358208864 |
| 4 | GAPN | H11'' | 34.8488722379 | 26.5290880214 | 21.2704908834 |
| 4 | GAPN | C12' | 33.7525899929 | 27.4801784697 | 19.6567324501 |
| 4 | GAPN | H12' | 34.3205101834 | 28.4349257192 | 19.7402577687 |
| 4 | GAPN | H12'' | 32.6940710916 | 27.7128112696 | 19.4008538836 |
| 4 | GAPN | O13' | 34.3317213809 | 26.6413045568 | 18.6668159849 |
| 4 | GAPN | H13' | 34.2977308702 | 27.1158784417 | 17.8310435766 |
| 4 | GAPN | C3' | 31.1389198601 | 25.0339113948 | 30.6336237319 |
| 4 | GAPN | O3' | 31.1495703634 | 23.9609910067 | 31.2450899073 |
| 4 | GAPN | C4' | 30.0585176845 | 26.0592999283 | 30.9918951905 |
| 4 | GAPN | H4' | 29.4652251373 | 26.2742342920 | 30.0910569029 |
| 4 | GAPN | H4'' | 30.5422323587 | 27.0081349730 | 31.3027501000 |
| 4 | GAPN | N9 | 29.2237214761 | 25.5751691367 | 32.0922739623 |
| 4 | GAPN | C5 | 28.4701675117 | 25.2149746724 | 34.1174665487 |
| 4 | GAPN | N7 | 27.5908713450 | 24.5689894671 | 33.2631033188 |
| 4 | GAPN | C8 | 28.0764544705 | 24.8054441985 | 32.0703025720 |
| 4 | GAPN | H8 | 27.6237198465 | 24.4515203831 | 31.1407898356 |
| 4 | GAPN | N1 | 29.5110702542 | 26.0984194524 | 36.0299625923 |
| 4 | GAPN | C2 | 30.4640808552 | 26.5680078038 | 35.2254805819 |
| 4 | GAPN | H2 | 31.2710204403 | 27.1214226336 | 35.7186104706 |
| 4 | GAPN | N3 | 30.5401044050 | 26.4969227694 | 33.8962817743 |
| 4 | GAPN | C4 | 29.4921405865 | 25.8115594040 | 33.4084963777 |
| 4 | GAPN | C6 | 28.4745574031 | 25.3959679950 | 35.5101780367 |
| 4 | GAPN | N6 | 27.4974488780 | 24.9210571567 | 36.3052690523 |
| 4 | GAPN | H61 | 26.7517648897 | 24.3777465045 | 35.9324446742 |
| 4 | GAPN | H62 | 27.4487216809 | 25.1804372820 | 37.2790409478 |
| 5 | GTPN | C | 37.3673488438 | 22.3526825925 | 34.1199136207 |
| 5 | GTPN | O1' | 38.0539320628 | 21.3653478887 | 33.9922002215 |
| 5 | GTPN | C2' | 36.3382226698 | 22.6968834854 | 33.1478082380 |
| 5 | GTPN | H2' | 35.8620926487 | 21.7822612990 | 32.8264332985 |
| 5 | GTPN | H2'' | 36.9468129532 | 23.1282035822 | 32.3793805096 |
| 5 | GTPN | N2' | 35.3620871882 | 23.6791690951 | 33.6265753750 |
| 5 | GTPN | C5' | 35.4860807214 | 25.0308271151 | 33.0895256771 |
| 5 | GTPN | H5' | 36.4995111777 | 25.3380285566 | 33.2763998077 |
| 5 | GTPN | H5'' | 34.7642512281 | 25.7132645482 | 33.5155030951 |
| 5 | GTPN | N | 34.0939777311 | 24.3496685179 | 31.2267192623 |
| 5 | GTPN | H1' | 33.2583469209 | 24.5990301750 | 31.7103418624 |

| | | | | | |
|---|---|---|---|---|---|
| 5 | GTPN | C6' | 35.3117237039 | 24.9879288014 | 31.5830706294 |
| 5 | GTPN | H6' | 36.1230515769 | 24.3720535673 | 31.2356976200 |
| 5 | GTPN | C6'' | 35.5263816454 | 26.2950988315 | 30.8073099868 |
| 5 | GTPN | H6'' | 35.9878696512 | 27.0689285795 | 31.4397141575 |
| 5 | GTPN | H6''' | 34.5474607985 | 26.6697107942 | 30.4778309638 |
| 5 | GTPN | O7' | 36.4027525941 | 25.9376020314 | 29.5900289249 |
| 5 | GTPN | C8' | 36.5058957682 | 26.9275041229 | 28.5809191572 |
| 5 | GTPN | H8' | 36.5518707275 | 27.9509558892 | 29.0041250335 |
| 5 | GTPN | H8'' | 35.6613681397 | 26.8728441974 | 27.8544651374 |
| 5 | GTPN | C9' | 37.7866280295 | 26.5720728392 | 27.8290566605 |
| 5 | GTPN | H9' | 37.6105024657 | 25.5952901412 | 27.3207879051 |
| 5 | GTPN | H9'' | 38.6460389036 | 26.4486824847 | 28.5270811750 |
| 5 | GTPN | O10' | 38.0577733827 | 27.5935904253 | 26.8881462873 |
| 5 | GTPN | C11' | 39.1828120502 | 27.2841550038 | 26.0779775193 |
| 5 | GTPN | H11' | 39.0701005184 | 26.2731783847 | 25.6242031049 |
| 5 | GTPN | H11'' | 40.1164451019 | 27.2975366699 | 26.6835151523 |
| 5 | GTPN | C12' | 39.2662083250 | 28.3356067983 | 24.9693716431 |
| 5 | GTPN | H12' | 39.4637802211 | 29.3337514252 | 25.4224300728 |
| 5 | GTPN | H12'' | 38.2897568498 | 28.3770117776 | 24.4336524167 |
| 5 | GTPN | O13' | 40.3072431653 | 27.9773997584 | 24.0708391492 |
| 5 | GTPN | H13' | 40.2842700373 | 28.5994641263 | 23.3373705049 |
| 5 | GTPN | C3' | 34.2332851226 | 23.1869800449 | 34.2069031576 |
| 5 | GTPN | O3' | 34.1320481904 | 21.9915072131 | 34.4823658752 |
| 5 | GTPN | C4' | 33.0763974676 | 24.1501841795 | 34.5110709948 |
| 5 | GTPN | H4' | 32.6948176077 | 24.5432740908 | 33.5452119751 |
| 5 | GTPN | H4'' | 33.4960478462 | 25.0111909648 | 35.0741960529 |
| 5 | GTPN | N1 | 31.9541930487 | 23.5326103981 | 35.2229929712 |
| 5 | GTPN | C6 | 30.9769913599 | 22.8239936854 | 34.5600278774 |
| 5 | GTPN | H6 | 31.1356398825 | 22.7493074878 | 33.4721286522 |
| 5 | GTPN | C2 | 31.9860039526 | 23.5979877666 | 36.6194232321 |
| 5 | GTPN | O2 | 32.8754305421 | 24.1026171731 | 37.2319460192 |
| 5 | GTPN | N3 | 30.8814586723 | 23.0173006404 | 37.1880266818 |
| 5 | GTPN | H3 | 30.8601365738 | 23.1089481029 | 38.2078754790 |
| 5 | GTPN | C4 | 29.8157780420 | 22.3628625904 | 36.6001274861 |
| 5 | GTPN | O4 | 28.8913004278 | 21.9597578481 | 37.2580676839 |
| 5 | GTPN | C5 | 29.9418243822 | 22.2639642555 | 35.1514579031 |
| 5 | GTPN | C5M | 28.8646397449 | 21.5348327237 | 34.4075997799 |
| 5 | GTPN | H51 | 29.0602984266 | 21.5537378245 | 33.3118304224 |
| 5 | GTPN | H52 | 27.8724174547 | 22.0071193116 | 34.5803095369 |
| 5 | GTPN | H53 | 28.8020869030 | 20.4727397904 | 34.7297615033 |
| 6 | GGPN | C | 39.1939823146 | 19.3717392621 | 38.2150487826 |
| 6 | GGPN | O1' | 40.2195681839 | 18.7006987235 | 38.3876654406 |
| 6 | GGPN | C2' | 38.7955745324 | 19.9684082795 | 36.9012572554 |
| 6 | GGPN | H2' | 38.3472741895 | 19.1818956090 | 36.3058067632 |
| 6 | GGPN | H2'' | 39.7009774961 | 20.3383013897 | 36.4677449762 |
| 6 | GGPN | N2' | 37.8715881639 | 21.1351120426 | 37.0702573600 |
| 6 | GGPN | C5' | 38.3647538159 | 22.5338269614 | 37.1502645184 |
| 6 | GGPN | H5' | 39.2414397530 | 22.5591170978 | 37.7397494761 |
| 6 | GGPN | H5'' | 37.6300135546 | 23.1983419945 | 37.5638105737 |
| 6 | GGPN | N | 37.5365752588 | 23.2413229908 | 35.1037297574 |
| 6 | GGPN | H1' | 36.8545151555 | 23.9213847796 | 35.3383629010 |
| 6 | GGPN | C6' | 38.7311991713 | 23.2239070401 | 35.8683186412 |
| 6 | GGPN | H6' | 39.4931554033 | 22.6376865272 | 35.3703622774 |
| 6 | GGPN | C6'' | 39.3260778803 | 24.6482805544 | 36.1371680035 |
| 6 | GGPN | H6'' | 40.4200771138 | 24.6748628069 | 35.9520058652 |
| 6 | GGPN | H6''' | 39.1737436847 | 24.9022039664 | 37.2110054536 |
| 6 | GGPN | O7' | 38.6004802062 | 25.7094985552 | 35.2663667058 |
| 6 | GGPN | C8' | 39.4778698745 | 26.4195457450 | 34.3869192319 |
| 6 | GGPN | H8' | 40.3468262641 | 25.7998228798 | 34.0709994312 |
| 6 | GGPN | H8'' | 39.8527501712 | 27.3349693006 | 34.8986742246 |
| 6 | GGPN | C9' | 38.6816190859 | 26.8377853355 | 33.1399466595 |
| 6 | GGPN | H9' | 37.6732607202 | 27.1196050062 | 33.5184256264 |
| 6 | GGPN | H9'' | 38.5661859365 | 25.9668575555 | 32.4563350471 |
| 6 | GGPN | O10' | 39.2047704523 | 27.9420369988 | 32.4131762753 |
| 6 | GGPN | C11' | 38.1899629215 | 28.4486242137 | 31.5569303370 |
| 6 | GGPN | H11' | 37.2959491309 | 28.6768412594 | 32.1844050737 |
| 6 | GGPN | H11'' | 37.9254956692 | 27.6773620024 | 30.8026172684 |
| 6 | GGPN | C12' | 38.5324654965 | 29.7342152256 | 30.8058505456 |
| 6 | GGPN | H12' | 39.2955397447 | 29.5426263174 | 30.0185841326 |
| 6 | GGPN | H12'' | 38.9343638255 | 30.4768238152 | 31.5310879131 |
| 6 | GGPN | O13' | 37.3208588045 | 30.2147295911 | 30.2275156690 |
| 6 | GGPN | H13' | 37.1836592288 | 29.7224148282 | 29.4114252121 |
| 6 | GGPN | C3' | 36.5586859017 | 20.8523198965 | 37.2913117113 |

| | | | | | |
|---|---|---|---|---|---|
| 6 | GGPN | O3' | 36.1676232371 | 19.6780664468 | 37.3412338517 |
| 6 | GGPN | C4' | 35.5624518576 | 21.9698563059 | 37.6133250798 |
| 6 | GGPN | H4' | 35.4332206798 | 22.6472466071 | 36.7478846733 |
| 6 | GGPN | H4'' | 35.9261070100 | 22.5418258420 | 38.4897070895 |
| 6 | GGPN | N9 | 34.3117857168 | 21.3429599183 | 37.9883086797 |
| 6 | GGPN | C4 | 33.8069393204 | 21.2065548363 | 39.2528116765 |
| 6 | GGPN | N2 | 34.1050264598 | 21.5000157406 | 42.7045357082 |
| 6 | GGPN | H21 | 34.9933146080 | 21.9211438726 | 42.8292685470 |
| 6 | GGPN | H22 | 33.5898527696 | 21.1721800679 | 43.5047648692 |
| 6 | GGPN | N3 | 34.3861299472 | 21.6294134389 | 40.4037066174 |
| 6 | GGPN | C2 | 33.6636116093 | 21.2845801218 | 41.4721303660 |
| 6 | GGPN | N1 | 32.4623403928 | 20.6309746327 | 41.3814515725 |
| 6 | GGPN | H1 | 31.9485970691 | 20.4473350220 | 42.2293356170 |
| 6 | GGPN | C6 | 31.8460521893 | 20.2070843489 | 40.2131210398 |
| 6 | GGPN | O6 | 30.7662466431 | 19.6102877557 | 40.2357006385 |
| 6 | GGPN | C5 | 32.6318183660 | 20.5033637703 | 39.0823894993 |
| 6 | GGPN | N7 | 32.4411555044 | 20.1558747633 | 37.7540863759 |
| 6 | GGPN | C8 | 33.4726678636 | 20.6696947394 | 37.1441296068 |
| 6 | GGPN | H8 | 33.7365405822 | 20.5633343619 | 36.0916923063 |
| 7 | GCPN | C | 39.2034294161 | 16.0460786158 | 43.0553085235 |
| 7 | GCPN | O1' | 40.0718668845 | 15.2463483079 | 43.3581011285 |
| 7 | GCPN | C2' | 39.2496961180 | 16.6634742098 | 41.6801951877 |
| 7 | GCPN | H2' | 39.1274619227 | 15.8700190169 | 40.9534794424 |
| 7 | GCPN | H2'' | 40.2598244131 | 17.0286076238 | 41.6407481579 |
| 7 | GCPN | N2' | 38.3880401741 | 17.8044606496 | 41.3378890262 |
| 7 | GCPN | C5' | 39.0254983722 | 19.1204439738 | 41.4951284285 |
| 7 | GCPN | H5' | 40.0885404346 | 18.9647714862 | 41.4246944287 |
| 7 | GCPN | H5'' | 38.7066524605 | 19.4870829840 | 42.4563651145 |
| 7 | GCPN | N | 38.3810481952 | 19.7016318764 | 39.2000252741 |
| 7 | GCPN | H1' | 37.4251956773 | 19.8246017909 | 38.9185774922 |
| 7 | GCPN | C6' | 38.7745750264 | 20.2332403245 | 40.4670523141 |
| 7 | GCPN | H6' | 37.8907412445 | 20.7599850648 | 40.8028924018 |
| 7 | GCPN | C6'' | 39.9275285109 | 21.3309921227 | 40.4661027412 |
| 7 | GCPN | H6'' | 40.7297800597 | 21.0750945315 | 41.1789638962 |
| 7 | GCPN | H6''' | 39.5206872480 | 22.3027188730 | 40.8136988494 |
| 7 | GCPN | O7' | 40.5053083102 | 21.5222746018 | 39.0448839135 |
| 7 | GCPN | C8' | 41.8439523385 | 21.9583088370 | 38.9051028644 |
| 7 | GCPN | H8' | 42.5147743924 | 21.3372420284 | 39.5296451808 |
| 7 | GCPN | H8'' | 41.9313621716 | 23.0270701480 | 39.2009239397 |
| 7 | GCPN | C9' | 42.2071253331 | 21.7754621377 | 37.4130244782 |
| 7 | GCPN | H9' | 41.2685177099 | 21.6264264416 | 36.8417558870 |
| 7 | GCPN | H9'' | 42.8158858813 | 20.8525187437 | 37.2855933845 |
| 7 | GCPN | O10' | 42.8668809857 | 22.8995118658 | 36.8535532666 |
| 7 | GCPN | C11' | 42.7870222016 | 22.9037518685 | 35.4313943028 |
| 7 | GCPN | H11' | 41.7246294050 | 22.9348088313 | 35.0983924645 |
| 7 | GCPN | H11'' | 43.2541469931 | 21.9863595964 | 35.0081336759 |
| 7 | GCPN | C12' | 43.4987697202 | 24.1587238407 | 34.9266679398 |
| 7 | GCPN | H12' | 44.5507175289 | 24.1683816740 | 35.2938609391 |
| 7 | GCPN | H12'' | 42.9847010274 | 25.0582134051 | 35.3351351012 |
| 7 | GCPN | O13' | 43.4576955797 | 24.1892258560 | 33.5078865403 |
| 7 | GCPN | H13' | 43.8081825717 | 23.3504976562 | 33.1915194719 |
| 7 | GCPN | C3' | 37.0950255803 | 17.5540721963 | 41.0169806280 |
| 7 | GCPN | O3' | 36.7090800456 | 16.3910511098 | 40.8136776647 |
| 7 | GCPN | C4' | 36.0361295195 | 18.6741451355 | 40.9562708160 |
| 7 | GCPN | H4' | 36.1557694662 | 19.3067608439 | 40.0637311098 |
| 7 | GCPN | H4'' | 36.1752615428 | 19.3159669494 | 41.8502090977 |
| 7 | GCPN | N1 | 34.6947717791 | 18.0977347831 | 40.8498865608 |
| 7 | GCPN | C6 | 34.2029121634 | 17.7006629778 | 39.6335647593 |
| 7 | GCPN | H6 | 34.8227140838 | 17.9433184746 | 38.7648695121 |
| 7 | GCPN | C5 | 33.0078685482 | 17.1047503797 | 39.5370649679 |
| 7 | GCPN | H5 | 32.5933652190 | 16.7880123231 | 38.5833819073 |
| 7 | GCPN | C2 | 34.0113117438 | 17.7949604846 | 42.0269289812 |
| 7 | GCPN | O2 | 34.5355491852 | 18.0400684658 | 43.1280965595 |
| 7 | GCPN | N3 | 32.7832193473 | 17.2472567297 | 41.9339580338 |
| 7 | GCPN | C4 | 32.2725197194 | 16.9027734305 | 40.7389095292 |
| 7 | GCPN | N4 | 31.0544855107 | 16.3708569975 | 40.6817154880 |
| 7 | GCPN | H41 | 30.5122060669 | 16.4352828972 | 41.5304370695 |
| 7 | GCPN | H42 | 30.5347616096 | 16.4697481439 | 39.8405960604 |
| 8 | GCPN | C | 37.2604717204 | 12.0984195584 | 47.1755698750 |
| 8 | GCPN | C2' | 37.8953517957 | 12.9051406222 | 46.0281747827 |
| 8 | GCPN | H2' | 38.2317665458 | 12.1883966562 | 45.2910424856 |
| 8 | GCPN | H2'' | 38.7258687285 | 13.3941649609 | 46.5054674320 |
| 8 | GCPN | N2' | 37.1198330575 | 13.9821648615 | 45.3731688416 |

| | | | | | |
|---|---|---|---|---|---|
| 8 | GCPN | C5' | 37.2664810494 | 15.3279672271 | 45.9532263853 |
| 8 | GCPN | H5' | 37.4089419915 | 15.1796713022 | 47.0121763693 |
| 8 | GCPN | H5'' | 36.3692742776 | 15.8929953926 | 45.8208010684 |
| 8 | GCPN | N | 38.2534893266 | 16.4031790809 | 43.9504601973 |
| 8 | GCPN | H1' | 37.4297584048 | 16.8924109733 | 43.6935976178 |
| 8 | GCPN | C6' | 38.4409732467 | 16.1807408588 | 45.3686489335 |
| 8 | GCPN | H6' | 39.3206901607 | 15.5693048032 | 45.5317810467 |
| 8 | GCPN | C6'' | 38.6989105486 | 17.5405003704 | 46.1290519587 |
| 8 | GCPN | H6'' | 39.3851628186 | 17.4183164531 | 46.9938108264 |
| 8 | GCPN | H6''' | 37.6954522766 | 17.8115354397 | 46.5214327587 |
| 8 | GCPN | O7' | 39.1711316678 | 18.7200204879 | 45.1862490749 |
| 8 | GCPN | C8' | 40.4619016202 | 19.2903933628 | 45.4881907781 |
| 8 | GCPN | H8' | 40.9348038475 | 18.8194325602 | 46.3761789191 |
| 8 | GCPN | H8'' | 40.3355792283 | 20.3774313163 | 45.7183006361 |
| 8 | GCPN | C9' | 41.4534382370 | 19.1418925731 | 44.2978420297 |
| 8 | GCPN | H9' | 40.8734881282 | 19.2220106438 | 43.3641612303 |
| 8 | GCPN | H9'' | 41.8627287768 | 18.1111518717 | 44.3329593312 |
| 8 | GCPN | O10' | 42.5296974912 | 20.0859753135 | 44.2938574654 |
| 8 | GCPN | C11' | 43.0134725238 | 20.4510351421 | 42.9890864261 |
| 8 | GCPN | H11' | 42.4118665040 | 20.0124449469 | 42.1598253646 |
| 8 | GCPN | H11'' | 44.0735866397 | 20.1324820913 | 42.8416650070 |
| 8 | GCPN | C12' | 42.9201602874 | 21.9787862467 | 42.9380834597 |
| 8 | GCPN | H12' | 43.6384264144 | 22.4325708268 | 43.6612553438 |
| 8 | GCPN | H12'' | 41.8891129412 | 22.2720943897 | 43.2403750656 |
| 8 | GCPN | O13' | 43.1589655530 | 22.4569313380 | 41.6260511847 |
| 8 | GCPN | H13' | 44.0990769750 | 22.3658354977 | 41.4455515366 |
| 8 | GCPN | C3' | 36.3751466468 | 13.7206579969 | 44.2540671700 |
| 8 | GCPN | O3' | 36.3567058790 | 12.6218547824 | 43.6693536981 |
| 8 | GCPN | C4' | 35.4939690880 | 14.8271049356 | 43.6193437235 |
| 8 | GCPN | H4' | 36.1105022906 | 15.3175395577 | 42.8451427375 |
| 8 | GCPN | H4'' | 35.2043138086 | 15.5993975115 | 44.3404908561 |
| 8 | GCPN | N1 | 34.2528200015 | 14.3426240044 | 43.0205588551 |
| 8 | GCPN | C6 | 34.1838830345 | 14.0568535968 | 41.6913687378 |
| 8 | GCPN | H6 | 35.1031792660 | 14.2576345379 | 41.1371376695 |
| 8 | GCPN | C5 | 33.0503599699 | 13.5983701105 | 41.1419650334 |
| 8 | GCPN | H5 | 32.9668348304 | 13.3670757461 | 40.0849211603 |
| 8 | GCPN | C2 | 33.1438235672 | 14.1543706870 | 43.8479203196 |
| 8 | GCPN | O2 | 33.2202597729 | 14.4107568487 | 45.0607230829 |
| 8 | GCPN | N3 | 31.9948718636 | 13.7245025901 | 43.3004879438 |
| 8 | GCPN | C4 | 31.9235146731 | 13.4260309260 | 41.9976964671 |
| 8 | GCPN | N4 | 30.7658337021 | 12.9782265138 | 41.5154046620 |
| 8 | GCPN | H41 | 29.9727794899 | 13.0633654420 | 42.1357497349 |
| 8 | GCPN | H42 | 30.5842358135 | 12.9136096201 | 40.5430612620 |
| 8 | GCPN | OT1 | 36.0926145964 | 12.3605157602 | 47.5640466948 |
| 8 | GCPN | OT2 | 37.9884593395 | 11.2109388583 | 47.7033072994 |
| 9 | GGPN | N | 23.7610378531 | 11.9531349444 | 50.2848187644 |
| 9 | GGPN | HT1 | 23.9001418855 | 12.8429542248 | 49.7594877082 |
| 9 | GGPN | HT2 | 22.7396334257 | 11.7604056898 | 50.3792371595 |
| 9 | GGPN | HT3 | 24.2042152514 | 11.1621593521 | 49.7777296509 |
| 9 | GGPN | C6' | 24.3323427837 | 12.1104108932 | 51.6572612306 |
| 9 | GGPN | H6' | 23.7724792073 | 12.9235797719 | 52.0970730088 |
| 9 | GGPN | C | 26.4605020786 | 15.9431870581 | 51.8305948309 |
| 9 | GGPN | O1' | 26.2162002735 | 17.0298228495 | 52.3241739083 |
| 9 | GGPN | C2' | 25.3579707488 | 15.0417752811 | 51.3842331265 |
| 9 | GGPN | H2' | 24.7312573546 | 15.5820123498 | 50.6877330315 |
| 9 | GGPN | H2'' | 24.8201241379 | 14.8097257395 | 52.2895336641 |
| 9 | GGPN | N2' | 25.8475252945 | 13.7965276247 | 50.7875535368 |
| 9 | GGPN | C5' | 25.7826808441 | 12.6043014382 | 51.6136627850 |
| 9 | GGPN | H5' | 26.4607707533 | 11.8348524378 | 51.2691050808 |
| 9 | GGPN | H5'' | 26.0426874350 | 12.9287066922 | 52.6086291903 |
| 9 | GGPN | C6'' | 24.0077715440 | 10.8912466043 | 52.5105928234 |
| 9 | GGPN | H6'' | 24.4550991691 | 11.0149900885 | 53.5174225584 |
| 9 | GGPN | H6''' | 24.3396851372 | 9.9250374390 | 52.0676957262 |
| 9 | GGPN | O7' | 22.4863188113 | 10.9401558892 | 52.5727813457 |
| 9 | GGPN | C8' | 21.9367220573 | 10.4618938070 | 53.7846721710 |
| 9 | GGPN | H8' | 22.5574839707 | 10.6947676297 | 54.6815208335 |
| 9 | GGPN | H8'' | 21.7841856962 | 9.3607874776 | 53.7200876477 |
| 9 | GGPN | C9' | 20.5983525249 | 11.1802425665 | 53.8739909316 |
| 9 | GGPN | H9' | 20.1865015807 | 11.3272872090 | 52.8487633803 |
| 9 | GGPN | H9'' | 20.7165865718 | 12.1777749249 | 54.3512036635 |
| 9 | GGPN | O10' | 19.6926800153 | 10.3941633813 | 54.6017397486 |
| 9 | GGPN | C11' | 18.3964391182 | 10.9510895091 | 54.5182745634 |
| 9 | GGPN | H11' | 18.0748888541 | 11.0447941738 | 53.4551482836 |

| | | | | | |
|---|---|---|---|---|---|
| 9 | GGPN | H11'' | 18.3819248063 | 11.9605318790 | 54.9863934744 |
| 9 | GGPN | C12' | 17.4705238572 | 9.9993949504 | 55.2540070675 |
| 9 | GGPN | H12' | 17.8193192202 | 9.8996908728 | 56.3077718839 |
| 9 | GGPN | H12'' | 17.5116657711 | 8.9993997738 | 54.7623303860 |
| 9 | GGPN | O13' | 16.1595595008 | 10.5376914343 | 55.1990324439 |
| 9 | GGPN | H13' | 15.5858485124 | 9.9348476857 | 55.6795696188 |
| 9 | GGPN | C3' | 26.1403979458 | 13.8019104914 | 49.4640427989 |
| 9 | GGPN | O3' | 25.9285535303 | 14.8033190722 | 48.7888404661 |
| 9 | GGPN | C4' | 26.6985496324 | 12.5293524894 | 48.8578002979 |
| 9 | GGPN | H4' | 26.0421062694 | 11.6664409594 | 49.0453129264 |
| 9 | GGPN | H4'' | 27.6809758582 | 12.3304320069 | 49.3446508925 |
| 9 | GGPN | N9 | 26.8551720336 | 12.6821487411 | 47.4180774718 |
| 9 | GGPN | C4 | 28.0398540230 | 12.9393852066 | 46.8095055571 |
| 9 | GGPN | N2 | 31.4585291334 | 13.4276737234 | 46.9928524856 |
| 9 | GGPN | H21 | 31.6599767587 | 13.3366355500 | 47.9600415128 |
| 9 | GGPN | H22 | 32.2085391080 | 13.6520178896 | 46.3602329951 |
| 9 | GGPN | N3 | 29.2206681199 | 13.0894960657 | 47.4363675799 |
| 9 | GGPN | C2 | 30.2133748552 | 13.2848531536 | 46.5728858555 |
| 9 | GGPN | N1 | 30.0177926420 | 13.3364267052 | 45.2160196610 |
| 9 | GGPN | H1 | 30.8202448264 | 13.4817519760 | 44.6225077970 |
| 9 | GGPN | C6 | 28.8048357721 | 13.1932914205 | 44.5507408319 |
| 9 | GGPN | O6 | 28.7148564195 | 13.2378705201 | 43.3185199005 |
| 9 | GGPN | C5 | 27.7520198923 | 12.9760727653 | 45.4604852428 |
| 9 | GGPN | N7 | 26.4006656655 | 12.7673214190 | 45.2334323854 |
| 9 | GGPN | C8 | 25.9015955472 | 12.6143000806 | 46.4205370726 |
| 9 | GGPN | H8 | 24.8410018199 | 12.4636594820 | 46.6124800749 |
| 10 | GGPN | C | 29.8560701685 | 20.3052769165 | 51.5332049126 |
| 10 | GGPN | O1' | 29.5820150206 | 21.3024144528 | 52.1890852095 |
| 10 | GGPN | C2' | 28.9515808261 | 19.1081767209 | 51.5349375132 |
| 10 | GGPN | H2' | 28.0001330326 | 19.4143934818 | 51.1358062939 |
| 10 | GGPN | H2'' | 28.8857236322 | 18.8576166948 | 52.5754113127 |
| 10 | GGPN | N2' | 29.3834104345 | 17.8789296356 | 50.8490717888 |
| 10 | GGPN | C5' | 29.8460564887 | 16.7414082306 | 51.6480325094 |
| 10 | GGPN | H5' | 30.6460169530 | 17.1349697368 | 52.2458141978 |
| 10 | GGPN | H5'' | 30.2596478728 | 15.9466998235 | 51.0741694248 |
| 10 | GGPN | N | 27.7130795668 | 15.4880395766 | 51.7145534907 |
| 10 | GGPN | H1' | 27.8989697866 | 14.6731736295 | 51.1702417508 |
| 10 | GGPN | C6' | 28.7839025045 | 16.0538011778 | 52.5114299196 |
| 10 | GGPN | H6' | 28.4009933574 | 16.8712268521 | 53.1113169635 |
| 10 | GGPN | C6'' | 29.3979288142 | 15.0331003577 | 53.5225502989 |
| 10 | GGPN | H6'' | 29.0313178898 | 15.4379665252 | 54.4786664052 |
| 10 | GGPN | H6''' | 30.5148075478 | 15.0310169219 | 53.5477558950 |
| 10 | GGPN | O7' | 28.8373319472 | 13.5782880112 | 53.3854614749 |
| 10 | GGPN | C8' | 28.6297225103 | 12.9748035429 | 54.6761055842 |
| 10 | GGPN | H8' | 28.2971054152 | 13.7510765837 | 55.3939912914 |
| 10 | GGPN | H8'' | 29.5653040105 | 12.5150026629 | 55.0626730906 |
| 10 | GGPN | C9' | 27.4819451844 | 11.9421612556 | 54.6507982559 |
| 10 | GGPN | H9' | 27.6006895767 | 11.3329305002 | 53.7279454956 |
| 10 | GGPN | H9'' | 26.5579622188 | 12.5548223086 | 54.5621250377 |
| 10 | GGPN | O10' | 27.2844156538 | 11.0525818768 | 55.7521587436 |
| 10 | GGPN | C11' | 26.0517295232 | 10.3775584630 | 55.5071710076 |
| 10 | GGPN | H11' | 26.1262583073 | 9.9746608575 | 54.4700789950 |
| 10 | GGPN | H11'' | 25.2314346520 | 11.1309511634 | 55.5428308416 |
| 10 | GGPN | C12' | 25.5469544044 | 9.1996818198 | 56.3600580650 |
| 10 | GGPN | H12' | 25.1692227849 | 9.5402465908 | 57.3512278440 |
| 10 | GGPN | H12'' | 26.3459390287 | 8.4399884031 | 56.5118615803 |
| 10 | GGPN | O13' | 24.4739467616 | 8.6750435617 | 55.5657664741 |
| 10 | GGPN | H13' | 24.0881015819 | 7.9150719130 | 56.0140423471 |
| 10 | GGPN | C3' | 29.1873523133 | 17.7848162741 | 49.5246376780 |
| 10 | GGPN | O3' | 28.8402156942 | 18.7877871759 | 48.8526959375 |
| 10 | GGPN | C4' | 29.4091984144 | 16.4337785793 | 48.8971090121 |
| 10 | GGPN | H4' | 28.7512393565 | 15.6741719735 | 49.3562793275 |
| 10 | GGPN | H4'' | 30.4733983459 | 16.1315773627 | 49.0212802315 |
| 10 | GGPN | N9 | 29.1105579149 | 16.5034783378 | 47.4924542515 |
| 10 | GGPN | C4 | 30.0365304234 | 16.7148722450 | 46.5285749962 |
| 10 | GGPN | N2 | 33.2991333620 | 17.3863050067 | 45.5878990269 |
| 10 | GGPN | H21 | 33.8007404637 | 17.3017898723 | 46.4393598878 |
| 10 | GGPN | H22 | 33.7808261839 | 17.5349774128 | 44.7201208270 |
| 10 | GGPN | N3 | 31.3564438261 | 16.9064662745 | 46.7356417562 |
| 10 | GGPN | C2 | 32.0115282470 | 17.0748329313 | 45.5945135551 |
| 10 | GGPN | N1 | 31.3976890779 | 17.0109140766 | 44.3727298452 |
| 10 | GGPN | H1 | 31.9725988999 | 17.0624511877 | 43.5462732410 |
| 10 | GGPN | C6 | 30.0446229874 | 16.7876913689 | 44.1395239658 |

| | | | | | |
|---|---|---|---|---|---|
| 10 | GGPN | O6 | 29.5778318113 | 16.7490418556 | 43.0022289596 |
| 10 | GGPN | C5 | 29.3316167693 | 16.6857018960 | 45.3518793107 |
| 10 | GGPN | N7 | 27.9714757754 | 16.5278484185 | 45.5843307340 |
| 10 | GGPN | C8 | 27.8857474252 | 16.4341745818 | 46.8828288507 |
| 10 | GGPN | H8 | 26.9717910763 | 16.3310056877 | 47.4708088297 |
| 11 | GCPN | C | 32.0649261398 | 24.8424382599 | 49.1907900701 |
| 11 | GCPN | O1' | 31.8254718130 | 25.9681458517 | 49.6015167759 |
| 11 | GCPN | C2' | 31.3673247759 | 23.6579918948 | 49.7300256298 |
| 11 | GCPN | H2' | 30.3019908299 | 23.8187810260 | 49.6482046791 |
| 11 | GCPN | H2'' | 31.6939181170 | 23.6457097016 | 50.7561143298 |
| 11 | GCPN | N2' | 31.7882179669 | 22.3861648521 | 49.1104186639 |
| 11 | GCPN | C5' | 32.7430221915 | 21.5831385509 | 49.8784551523 |
| 11 | GCPN | H5' | 33.5391778507 | 22.2562821260 | 50.1607517845 |
| 11 | GCPN | H5'' | 33.1210961962 | 20.7706168906 | 49.2786530255 |
| 11 | GCPN | N | 30.9849156102 | 20.2230445320 | 50.8281419106 |
| 11 | GCPN | H1' | 31.0935817780 | 19.4844242861 | 50.1790465603 |
| 11 | GCPN | C6' | 32.1551513127 | 20.9800867206 | 51.1674971687 |
| 11 | GCPN | H6' | 31.8794392275 | 21.8206860011 | 51.7962343982 |
| 11 | GCPN | C6'' | 33.1608167966 | 20.1125717811 | 51.9953744674 |
| 11 | GCPN | H6'' | 33.6305852326 | 20.7056280264 | 52.8084576968 |
| 11 | GCPN | H6''' | 33.9706742379 | 19.8007428159 | 51.2996197854 |
| 11 | GCPN | O7' | 32.4641551491 | 18.8213218963 | 52.5624538526 |
| 11 | GCPN | C8' | 32.5768829356 | 18.6654834157 | 53.9837450290 |
| 11 | GCPN | H8' | 33.2113666309 | 19.4585863296 | 54.4254027773 |
| 11 | GCPN | H8'' | 33.1068164018 | 17.7053865684 | 54.1852842203 |
| 11 | GCPN | C9' | 31.2477470744 | 18.6297966738 | 54.7893303719 |
| 11 | GCPN | H9' | 30.3796854166 | 18.5518821811 | 54.1008940879 |
| 11 | GCPN | H9'' | 31.1561050571 | 19.5700637380 | 55.3752488993 |
| 11 | GCPN | O10' | 31.2591408295 | 17.5242652451 | 55.6855590871 |
| 11 | GCPN | C11' | 29.9933354222 | 17.2553869627 | 56.2845957000 |
| 11 | GCPN | H11' | 29.1925706184 | 17.2814073779 | 55.5162673809 |
| 11 | GCPN | H11'' | 29.7255597041 | 17.9969320714 | 57.0721331775 |
| 11 | GCPN | C12' | 30.0898713015 | 15.8495859905 | 56.8899010946 |
| 11 | GCPN | H12' | 30.7390339698 | 15.8784118354 | 57.7938769750 |
| 11 | GCPN | H12'' | 30.5606353524 | 15.1663088541 | 56.1441207491 |
| 11 | GCPN | O13' | 28.7850552206 | 15.3838719493 | 57.2076745882 |
| 11 | GCPN | H13' | 28.8814381182 | 14.5737570981 | 57.7197664345 |
| 11 | GCPN | C3' | 31.2108041503 | 21.9691854336 | 47.9469589791 |
| 11 | GCPN | O3' | 30.5054136389 | 22.7426411609 | 47.2676055434 |
| 11 | GCPN | C4' | 31.4181092936 | 20.5355972326 | 47.4211176468 |
| 11 | GCPN | H4' | 30.9611697145 | 19.8503614833 | 48.1626346690 |
| 11 | GCPN | H4'' | 32.5011763128 | 20.3071749228 | 47.3708942625 |
| 11 | GCPN | N1 | 30.7705879263 | 20.3211094698 | 46.1201704899 |
| 11 | GCPN | C6 | 29.4209201509 | 20.1216682132 | 46.0469334032 |
| 11 | GCPN | H6 | 28.9075101652 | 20.0463390878 | 47.0076848398 |
| 11 | GCPN | C5 | 28.7985215218 | 20.0018006239 | 44.8682914565 |
| 11 | GCPN | H5 | 27.7235767968 | 19.8532536611 | 44.7999773589 |
| 11 | GCPN | C2 | 31.5184531037 | 20.5015664128 | 44.9515218131 |
| 11 | GCPN | O2 | 32.7129047594 | 20.8370715962 | 45.0320746540 |
| 11 | GCPN | N3 | 30.9190547075 | 20.3150387458 | 43.7557872909 |
| 11 | GCPN | C4 | 29.5939649367 | 20.0757363453 | 43.6868326809 |
| 11 | GCPN | N4 | 29.0233611353 | 19.8924638375 | 42.4956053081 |
| 11 | GCPN | H41 | 29.6173635766 | 19.7773032600 | 41.6936788776 |
| 11 | GCPN | H42 | 28.1099412620 | 19.5032395651 | 42.4425512214 |
| 12 | GAPN | C | 32.9358474404 | 28.8502426527 | 45.4172169390 |
| 12 | GAPN | O1' | 33.0320082021 | 30.0167561641 | 45.7787418387 |
| 12 | GAPN | C2' | 32.5535995364 | 27.7846587470 | 46.3574013801 |
| 12 | GAPN | H2' | 31.5011945643 | 27.8854042104 | 46.5668788306 |
| 12 | GAPN | H2'' | 33.1668101520 | 28.0100386574 | 47.2128339163 |
| 12 | GAPN | N2' | 32.8541043543 | 26.4164010811 | 45.8910394797 |
| 12 | GAPN | C5' | 34.0399071557 | 25.7396882227 | 46.4232770960 |
| 12 | GAPN | H5' | 34.8739866578 | 26.3928812323 | 46.2477955588 |
| 12 | GAPN | H5'' | 34.1823423534 | 24.7634046817 | 45.9796468701 |
| 12 | GAPN | N | 32.9978232841 | 24.5928168028 | 48.2684518836 |
| 12 | GAPN | H1' | 33.0196142442 | 23.6910465133 | 47.8506288790 |
| 12 | GAPN | C6' | 34.0038567875 | 25.5468537035 | 47.9222380998 |
| 12 | GAPN | H6' | 33.7447102526 | 26.5107925133 | 48.3326968547 |
| 12 | GAPN | C6'' | 35.3984131676 | 25.2509741102 | 48.5086784765 |
| 12 | GAPN | H6'' | 36.1712223169 | 25.4203150646 | 47.7330752321 |
| 12 | GAPN | H6''' | 35.4588211218 | 24.2011561892 | 48.8641756486 |
| 12 | GAPN | O7' | 35.6572875923 | 26.2255063181 | 49.6540397050 |
| 12 | GAPN | C8' | 36.0693649569 | 25.5903639446 | 50.8510690902 |
| 12 | GAPN | H8' | 37.1428210145 | 25.3014649631 | 50.7927797703 |

| | | | | | |
|---|---|---|---|---|---|
| 12 | GAPN | H8'' | 35.4541952118 | 24.6912633027 | 51.0789467493 |
| 12 | GAPN | C9' | 35.8608083839 | 26.6453023252 | 51.9294394694 |
| 12 | GAPN | H9' | 34.7677314956 | 26.7923247022 | 52.0847231989 |
| 12 | GAPN | H9'' | 36.2960668931 | 27.6062014970 | 51.5700859999 |
| 12 | GAPN | O10' | 36.4842190528 | 26.2694133341 | 53.1407594449 |
| 12 | GAPN | C11' | 36.3646750850 | 27.3099341570 | 54.0988234895 |
| 12 | GAPN | H11' | 35.2910499986 | 27.5001665697 | 54.3221787582 |
| 12 | GAPN | H11'' | 36.8118022740 | 28.2504161573 | 53.7033998484 |
| 12 | GAPN | C12' | 37.0966536729 | 26.8940956627 | 55.3735821046 |
| 12 | GAPN | H12' | 38.1687061333 | 26.7103050824 | 55.1329701461 |
| 12 | GAPN | H12'' | 36.6476998378 | 25.9504628086 | 55.7584663108 |
| 12 | GAPN | O13' | 36.9734007231 | 27.9332821221 | 56.3349737206 |
| 12 | GAPN | H13' | 37.4408232085 | 27.6506807306 | 57.1265095519 |
| 12 | GAPN | C3' | 31.9266988778 | 25.8161449793 | 45.0906724527 |
| 12 | GAPN | O3' | 31.0254984848 | 26.4717773246 | 44.5600002503 |
| 12 | GAPN | C4' | 31.9974104344 | 24.3135323606 | 44.8089893646 |
| 12 | GAPN | H4' | 31.9211856605 | 23.7734978128 | 45.7641983716 |
| 12 | GAPN | H4'' | 32.9867265872 | 24.0687205807 | 44.3699273089 |
| 12 | GAPN | N9 | 30.9543725384 | 23.9058402550 | 43.8677442967 |
| 12 | GAPN | C5 | 29.9366667436 | 23.4101179839 | 41.9926707465 |
| 12 | GAPN | N7 | 29.0061223267 | 23.2008400638 | 42.9986309468 |
| 12 | GAPN | C8 | 29.6509972403 | 23.5082709289 | 44.0956275436 |
| 12 | GAPN | H8 | 29.2225128496 | 23.4465816278 | 45.0990756684 |
| 12 | GAPN | N1 | 31.0123405180 | 23.5153007166 | 39.9061630933 |
| 12 | GAPN | C2 | 32.0699573698 | 24.0205473917 | 40.5398191937 |
| 12 | GAPN | H2 | 32.9331969866 | 24.2588943029 | 39.9084714262 |
| 12 | GAPN | N3 | 32.2389886420 | 24.2183821335 | 41.8474614281 |
| 12 | GAPN | C4 | 31.1256328697 | 23.8733589498 | 42.5155367689 |
| 12 | GAPN | C6 | 29.8965598022 | 23.1936694740 | 40.6054156959 |
| 12 | GAPN | N6 | 28.8219152681 | 22.6735513000 | 39.9829646350 |
| 12 | GAPN | H61 | 27.9889574228 | 22.4659055304 | 40.4863895894 |
| 12 | GAPN | H62 | 28.8632456096 | 22.4090756011 | 39.0107385382 |
| 13 | GTPN | C | 33.2502164025 | 32.1834167439 | 40.9629262917 |
| 13 | GTPN | O1' | 32.9094395343 | 33.3324933287 | 41.1408345753 |
| 13 | GTPN | C2' | 33.0913121001 | 31.1781806089 | 42.0319711953 |
| 13 | GTPN | H2' | 32.1581112862 | 31.3820653884 | 42.5373378871 |
| 13 | GTPN | H2'' | 33.9311750535 | 31.4107417678 | 42.6565831309 |
| 13 | GTPN | N2' | 33.1663542822 | 29.7676382596 | 41.6437344190 |
| 13 | GTPN | C5' | 34.3476846147 | 29.0345058423 | 42.0917558077 |
| 13 | GTPN | H5' | 35.1910656766 | 29.6054882264 | 41.7561664323 |
| 13 | GTPN | H5'' | 34.3696353760 | 28.0180124737 | 41.7262756289 |
| 13 | GTPN | N | 33.2467470278 | 28.4619343199 | 44.1828373544 |
| 13 | GTPN | H1' | 32.8816757731 | 27.6120189451 | 43.8109958646 |
| 13 | GTPN | C6' | 34.4222422902 | 29.0166486614 | 43.6095854767 |
| 13 | GTPN | H6' | 34.4715179423 | 30.0563603227 | 43.8882894816 |
| 13 | GTPN | C6'' | 35.6981232023 | 28.4229697787 | 44.2314950406 |
| 13 | GTPN | H6'' | 36.4550435123 | 28.1981062787 | 43.4628404270 |
| 13 | GTPN | H6''' | 35.4469458186 | 27.4806229057 | 44.7402182620 |
| 13 | GTPN | O7' | 36.2114850892 | 29.4471082109 | 45.2554867114 |
| 13 | GTPN | C8' | 37.1273171089 | 28.9482291557 | 46.2160492453 |
| 13 | GTPN | H8' | 37.9373343806 | 28.3519014349 | 45.7473972064 |
| 13 | GTPN | H8'' | 36.6232023780 | 28.3348729829 | 46.9988700826 |
| 13 | GTPN | C9' | 37.6871193715 | 30.2068897726 | 46.8705294739 |
| 13 | GTPN | H9' | 36.8505709486 | 30.7105515994 | 47.4083804501 |
| 13 | GTPN | H9'' | 38.0761173811 | 30.9077986540 | 46.0969580838 |
| 13 | GTPN | O10' | 38.7192810207 | 29.8527808001 | 47.7685386709 |
| 13 | GTPN | C11' | 39.2009908755 | 30.9855671393 | 48.4755396936 |
| 13 | GTPN | H11' | 38.3601637802 | 31.5178403816 | 48.9759573896 |
| 13 | GTPN | H11'' | 39.7044378291 | 31.6949659763 | 47.7810414463 |
| 13 | GTPN | C12' | 40.1918660522 | 30.4889221809 | 49.5272509486 |
| 13 | GTPN | H12' | 41.0522496310 | 29.9986853056 | 49.0172287822 |
| 13 | GTPN | H12'' | 39.6853225490 | 29.7359425590 | 50.1742960269 |
| 13 | GTPN | O13' | 40.6321577199 | 31.5944111854 | 50.3032344110 |
| 13 | GTPN | H13' | 41.1947864175 | 31.2494354442 | 51.0028986513 |
| 13 | GTPN | C3' | 32.0017705949 | 29.1715217365 | 41.2675172286 |
| 13 | GTPN | O3' | 30.9760876133 | 29.8303196593 | 41.0967614551 |
| 13 | GTPN | C4' | 31.9863195704 | 27.6480746689 | 41.0602513935 |
| 13 | GTPN | H4' | 32.1875381054 | 27.1641860229 | 42.0381134278 |
| 13 | GTPN | H4'' | 32.8411863955 | 27.4007898378 | 40.3900984522 |
| 13 | GTPN | N1 | 30.7353083480 | 27.1210233291 | 40.5330396647 |
| 13 | GTPN | C6 | 29.6524345875 | 26.8527259583 | 41.3683359585 |
| 13 | GTPN | H6 | 29.8375963823 | 27.1037773554 | 42.4259815251 |
| 13 | GTPN | C2 | 30.5752332975 | 26.9876396280 | 39.1827156451 |

| | | | | | |
|---|---|---|---|---|---|
| 13 | GTPN | O2 | 31.5045938133 | 27.3475810069 | 38.3703432399 |
| 13 | GTPN | N3 | 29.4236920007 | 26.4689161561 | 38.7659171562 |
| 13 | GTPN | H3 | 29.3352621321 | 26.3254029661 | 37.7605842075 |
| 13 | GTPN | C4 | 28.3153718702 | 26.0943456856 | 39.5358063853 |
| 13 | GTPN | O4 | 27.3545108435 | 25.5877296292 | 39.0338881465 |
| 13 | GTPN | C5 | 28.5125998812 | 26.3647875574 | 40.9540569722 |
| 13 | GTPN | C5M | 27.3697003266 | 26.0489446713 | 41.8783179778 |
| 13 | GTPN | H51 | 27.6489178968 | 26.2675137410 | 42.9336900825 |
| 13 | GTPN | H52 | 27.0945466587 | 24.9731347385 | 41.8179555850 |
| 13 | GTPN | H53 | 26.4703186968 | 26.6510012601 | 41.6257668260 |
| 14 | GGPN | C | 31.2397899467 | 35.4420741145 | 37.4332305065 |
| 14 | GGPN | O1' | 31.1563499141 | 36.6707654334 | 37.5263111480 |
| 14 | GGPN | C2' | 31.8078540468 | 34.5900755415 | 38.5368289784 |
| 14 | GGPN | H2' | 31.1292234034 | 34.5971828473 | 39.3853116325 |
| 14 | GGPN | H2'' | 32.7569884111 | 35.0273816485 | 38.7899362560 |
| 14 | GGPN | N2' | 32.0934179174 | 33.2008584503 | 38.1041880806 |
| 14 | GGPN | C5' | 33.4734021939 | 32.8281611588 | 37.7485795576 |
| 14 | GGPN | H5' | 33.8360526001 | 33.5674308354 | 37.0559651759 |
| 14 | GGPN | H5'' | 33.4957869987 | 31.8563702163 | 37.2975010455 |
| 14 | GGPN | N | 33.8510697395 | 31.7753529906 | 39.8324583046 |
| 14 | GGPN | H1' | 33.9685918841 | 30.8210243937 | 39.5970441001 |
| 14 | GGPN | C6' | 34.4412832297 | 32.7066733428 | 38.9266482033 |
| 14 | GGPN | H6' | 34.5168007134 | 33.6760621566 | 39.4025544151 |
| 14 | GGPN | C6'' | 35.8992439042 | 32.2784920007 | 38.5188156660 |
| 14 | GGPN | H6'' | 36.6264049293 | 33.1117736386 | 38.6256248983 |
| 14 | GGPN | H6''' | 35.8949385086 | 31.9788307201 | 37.4474914079 |
| 14 | GGPN | O7' | 36.3602977641 | 31.0617621443 | 39.3817726678 |
| 14 | GGPN | C8' | 37.6017608705 | 31.2711213431 | 40.0680322740 |
| 14 | GGPN | H8' | 37.7331456926 | 32.3326915267 | 40.3733952553 |
| 14 | GGPN | H8'' | 38.4505401083 | 30.9726736473 | 39.4113583033 |
| 14 | GGPN | C9' | 37.6361530023 | 30.3993085623 | 41.3370389678 |
| 14 | GGPN | H9' | 37.2295307263 | 29.4071446971 | 41.0362930082 |
| 14 | GGPN | H9'' | 36.9583634972 | 30.8390623563 | 42.1028566802 |
| 14 | GGPN | O10' | 38.9195377292 | 30.1986985797 | 41.9170322310 |
| 14 | GGPN | C11' | 38.8284384023 | 29.1516645349 | 42.8751769416 |
| 14 | GGPN | H11' | 38.3737894494 | 28.2683269221 | 42.3689949182 |
| 14 | GGPN | H11'' | 38.1706695523 | 29.4737796397 | 43.7091009757 |
| 14 | GGPN | C12' | 40.1470072197 | 28.6685640002 | 43.4837675461 |
| 14 | GGPN | H12' | 40.5592369637 | 29.4236029522 | 44.1900737734 |
| 14 | GGPN | H12'' | 40.8791026263 | 28.4985053508 | 42.6629809867 |
| 14 | GGPN | O13' | 39.8758761371 | 27.4394425134 | 44.1560440517 |
| 14 | GGPN | H13' | 39.7519702535 | 27.6421567845 | 45.0890124293 |
| 14 | GGPN | C3' | 31.0601161442 | 32.3134990619 | 38.0643001057 |
| 14 | GGPN | O3' | 29.8930378398 | 32.7063892339 | 38.1890064389 |
| 14 | GGPN | C4' | 31.3176688616 | 30.8246685481 | 37.7988133556 |
| 14 | GGPN | H4' | 31.8890221207 | 30.3765746486 | 38.6346148475 |
| 14 | GGPN | H4'' | 31.8808493014 | 30.6989771239 | 36.8544742435 |
| 14 | GGPN | N9 | 30.0291829463 | 30.1706396957 | 37.6581137236 |
| 14 | GGPN | C4 | 29.4412610778 | 29.7699417285 | 36.4908029680 |
| 14 | GGPN | N2 | 29.3817736025 | 29.6158257281 | 33.0284205907 |
| 14 | GGPN | H21 | 30.2420976618 | 30.0379886937 | 32.7752624032 |
| 14 | GGPN | H22 | 28.7222078564 | 29.3455534020 | 32.3185601434 |
| 14 | GGPN | N3 | 29.9519748342 | 29.9115855380 | 35.2467084178 |
| 14 | GGPN | C2 | 29.1034910639 | 29.4816629887 | 34.3152467044 |
| 14 | GGPN | N1 | 27.8840942875 | 28.9254219145 | 34.6085136701 |
| 14 | GGPN | H1 | 27.3140975396 | 28.5863072916 | 33.8496309875 |
| 14 | GGPN | C6 | 27.3514134140 | 28.7523045298 | 35.8774418302 |
| 14 | GGPN | O6 | 26.2346489188 | 28.2483036531 | 36.0396309874 |
| 14 | GGPN | C5 | 28.2163870671 | 29.2561827300 | 36.8712787721 |
| 14 | GGPN | N7 | 28.0254465102 | 29.3802480381 | 38.2386438371 |
| 14 | GGPN | C8 | 29.1275873403 | 29.9363894411 | 38.6624253315 |
| 14 | GGPN | H8 | 29.3495794427 | 30.2513419478 | 39.6821388379 |
| 15 | GCPN | C | 27.5168443336 | 36.8519240085 | 32.4618654318 |
| 15 | GCPN | O1' | 27.2873805213 | 37.9513711341 | 31.9846922678 |
| 15 | GCPN | C2' | 28.3283318080 | 36.7830508531 | 33.7488791024 |
| 15 | GCPN | H2' | 27.7419452551 | 37.2693118169 | 34.5176531142 |
| 15 | GCPN | H2'' | 29.1837471075 | 37.3942040060 | 33.5141539402 |
| 15 | GCPN | N2' | 28.8841899136 | 35.5095329150 | 34.2597015267 |
| 15 | GCPN | C5' | 30.2457627561 | 35.1558744413 | 33.8675698063 |
| 15 | GCPN | H5' | 30.6429797589 | 35.9885415366 | 33.3181838493 |
| 15 | GCPN | H5'' | 30.1351017788 | 34.3120038356 | 33.2022443792 |
| 15 | GCPN | N | 30.8455507529 | 34.7548244048 | 36.3532356541 |
| 15 | GCPN | H1' | 30.3865806407 | 33.9146481586 | 36.6492383974 |

| | | | | | |
|---|---|---|---|---|---|
| 15 | GCPN | C6' | 31.3186989402 | 34.8204744287 | 34.9705806129 |
| 15 | GCPN | H6' | 31.5033283246 | 33.7646316963 | 34.8004409853 |
| 15 | GCPN | C6'' | 32.7407694317 | 35.4482832508 | 34.6650837625 |
| 15 | GCPN | H6'' | 32.6693653302 | 36.4042003020 | 34.1008303084 |
| 15 | GCPN | H6''' | 33.3769302344 | 34.7510131956 | 34.0845482519 |
| 15 | GCPN | O7' | 33.4803466363 | 35.7268276444 | 35.9492879533 |
| 15 | GCPN | C8' | 34.8556653349 | 35.9866719884 | 35.7908499566 |
| 15 | GCPN | H8' | 35.0641228617 | 36.7512237928 | 35.0096998655 |
| 15 | GCPN | H8'' | 35.3942868551 | 35.0454532278 | 35.5431670536 |
| 15 | GCPN | C9' | 35.2107822193 | 36.4708278387 | 37.1872216585 |
| 15 | GCPN | H9' | 34.5998319720 | 35.8952674962 | 37.9208214742 |
| 15 | GCPN | H9'' | 34.9386153942 | 37.5436000594 | 37.3040587603 |
| 15 | GCPN | O10' | 36.5688593696 | 36.2546370503 | 37.4869616642 |
| 15 | GCPN | C11' | 36.7428856518 | 36.1838102183 | 38.8893874589 |
| 15 | GCPN | H11' | 36.2648602627 | 35.2620802595 | 39.2907673055 |
| 15 | GCPN | H11'' | 36.2647415295 | 37.0619283086 | 39.3800320012 |
| 15 | GCPN | C12' | 38.2388255639 | 36.1495628419 | 39.1514723307 |
| 15 | GCPN | H12' | 38.7244924348 | 37.0100199524 | 38.6348203255 |
| 15 | GCPN | H12'' | 38.6649642995 | 35.2073840527 | 38.7376663609 |
| 15 | GCPN | O13' | 38.4496439933 | 36.2149840343 | 40.5517695604 |
| 15 | GCPN | H13' | 37.9535308039 | 36.9746117786 | 40.8736431900 |
| 15 | GCPN | C3' | 28.0575515670 | 34.6260259002 | 34.8670741550 |
| 15 | GCPN | O3' | 26.9320083067 | 34.9707848225 | 35.2662998715 |
| 15 | GCPN | C4' | 28.4521281916 | 33.1409289344 | 35.0205135069 |
| 15 | GCPN | H4' | 29.1557335929 | 33.0109937227 | 35.8575492703 |
| 15 | GCPN | H4'' | 28.9597982195 | 32.8014856879 | 34.0972086215 |
| 15 | GCPN | N1 | 27.2529915103 | 32.3445078620 | 35.2914696237 |
| 15 | GCPN | C6 | 26.8036350334 | 32.1667519916 | 36.5712254586 |
| 15 | GCPN | H6 | 27.4640659843 | 32.5317968047 | 37.3621365561 |
| 15 | GCPN | C5 | 25.6540735716 | 31.5218402642 | 36.8150698238 |
| 15 | GCPN | H5 | 25.2828792775 | 31.3531371956 | 37.8214494151 |
| 15 | GCPN | C2 | 26.4794088636 | 31.9373119142 | 34.2007647787 |
| 15 | GCPN | O2 | 26.8467618453 | 32.2086101308 | 33.0453701132 |
| 15 | GCPN | N3 | 25.3409807318 | 31.2559930622 | 34.4412506730 |
| 15 | GCPN | C4 | 24.9117507855 | 31.0509026363 | 35.6969758945 |
| 15 | GCPN | N4 | 23.7680011740 | 30.3997325234 | 35.9009821979 |
| 15 | GCPN | H41 | 23.3628187845 | 29.9468613738 | 35.0929521777 |
| 15 | GCPN | H42 | 23.6232515872 | 29.9314470792 | 36.7641159412 |
| 16 | GCPN | C | 22.3837371530 | 37.1592221753 | 29.4919924446 |
| 16 | GCPN | C2' | 23.6886677130 | 37.3411859572 | 30.2852028868 |
| 16 | GCPN | H2' | 23.5579326038 | 38.2044120158 | 30.9228694051 |
| 16 | GCPN | H2'' | 24.4251298438 | 37.5241110049 | 29.5213628378 |
| 16 | GCPN | N2' | 24.1996862947 | 36.2038719990 | 31.0736269852 |
| 16 | GCPN | C5' | 25.1188191873 | 35.3082208891 | 30.3656804865 |
| 16 | GCPN | H5' | 24.7942941292 | 35.2752910204 | 29.3392453242 |
| 16 | GCPN | H5'' | 25.0422212381 | 34.3154135990 | 30.7481838891 |
| 16 | GCPN | N | 27.0771278311 | 35.7304007945 | 31.8358637437 |
| 16 | GCPN | H1' | 27.1271402307 | 34.8371715544 | 32.2639924416 |
| 16 | GCPN | C6' | 26.6073312314 | 35.7388673855 | 30.4536207039 |
| 16 | GCPN | H6' | 26.6331128565 | 36.7527878944 | 30.0721842698 |
| 16 | GCPN | C6'' | 27.5644273205 | 34.9125869999 | 29.5303225828 |
| 16 | GCPN | H6'' | 27.5805606332 | 35.2707191925 | 28.4782674972 |
| 16 | GCPN | H6''' | 27.2274070592 | 33.8551633530 | 29.5677593717 |
| 16 | GCPN | O7' | 28.9672284598 | 34.9878069342 | 30.1573421512 |
| 16 | GCPN | C8' | 30.0976367569 | 34.7398842962 | 29.3231546516 |
| 16 | GCPN | H8' | 30.2104259394 | 35.4965612759 | 28.5159663027 |
| 16 | GCPN | H8'' | 30.1088659212 | 33.7053922601 | 28.9186154145 |
| 16 | GCPN | C9' | 31.1685565991 | 34.9339124045 | 30.3973847945 |
| 16 | GCPN | H9' | 30.9264196490 | 34.1705787167 | 31.1709800096 |
| 16 | GCPN | H9'' | 31.0017226472 | 35.9534154622 | 30.8222425368 |
| 16 | GCPN | O10' | 32.5457497711 | 34.8174090836 | 30.1215870818 |
| 16 | GCPN | C11' | 33.2081275869 | 34.9996377577 | 31.3678071155 |
| 16 | GCPN | H11' | 32.8630129234 | 34.2144711909 | 32.0788271945 |
| 16 | GCPN | H11'' | 32.9534177431 | 36.0047366361 | 31.7857479839 |
| 16 | GCPN | C12' | 34.7187687433 | 34.8863398107 | 31.2759341145 |
| 16 | GCPN | H12' | 35.0676720455 | 35.5864228482 | 30.4836773608 |
| 16 | GCPN | H12'' | 34.9720275459 | 33.8317716170 | 31.0187820955 |
| 16 | GCPN | O13' | 35.2551075914 | 35.2447531986 | 32.5463853574 |
| 16 | GCPN | H13' | 36.2127054780 | 35.2751767523 | 32.4609585153 |
| 16 | GCPN | C3' | 23.8867981254 | 36.0553062562 | 32.3967202114 |
| 16 | GCPN | O3' | 23.2065115925 | 36.8678336325 | 33.0512015027 |
| 16 | GCPN | C4' | 24.3922877032 | 34.8307742094 | 33.2075313201 |
| 16 | GCPN | H4' | 25.0791444163 | 35.2564588190 | 33.9564294262 |

| | | | | | |
|---|---|---|---|---|---|
| 16 | GCPN | H4'' | 25.0056554688 | 34.1321930452 | 32.6344614073 |
| 16 | GCPN | N1 | 23.3522628924 | 34.0435560164 | 33.8672978431 |
| 16 | GCPN | C6 | 23.1973354339 | 34.0812821214 | 35.2213930623 |
| 16 | GCPN | H6 | 23.8994671253 | 34.7348656121 | 35.7434469263 |
| 16 | GCPN | C5 | 22.2549144520 | 33.3474054940 | 35.8300949524 |
| 16 | GCPN | H5 | 22.1128414068 | 33.3557791879 | 36.9070266258 |
| 16 | GCPN | C2 | 22.5920464873 | 33.1652026675 | 33.0929824452 |
| 16 | GCPN | O2 | 22.8337757300 | 33.0419878792 | 31.8804558194 |
| 16 | GCPN | N3 | 21.6260427178 | 32.4430010192 | 33.6882120938 |
| 16 | GCPN | C4 | 21.4257757379 | 32.5247875935 | 35.0106824324 |
| 16 | GCPN | N4 | 20.4388325049 | 31.8082082669 | 35.5454036111 |
| 16 | GCPN | H41 | 19.9427021462 | 31.2001525996 | 34.9078426122 |
| 16 | GCPN | H42 | 20.3475838097 | 31.6426533023 | 36.5182665018 |
| 16 | GCPN | OT1 | 21.7734308962 | 36.0584121099 | 29.5212672334 |
| 16 | GCPN | OT2 | 22.0039097676 | 38.1497547642 | 28.8062726797 |

Model 4

| | | | | | |
|---|---|---|---|---|---|
| 1 | GGPN | N | 15.0227747566 | 32.0983513484 | 24.4773722519 |
| 1 | GGPN | HT1 | 15.4035095006 | 32.1836605478 | 25.3785282043 |
| 1 | GGPN | HT2 | 13.9986487181 | 32.0961400390 | 24.3929415413 |
| 1 | GGPN | HT3 | 15.3706581245 | 32.9713343280 | 23.8815640278 |
| 1 | GGPN | C6' | 15.5680376657 | 30.8174844000 | 23.9958609368 |
| 1 | GGPN | H6' | 14.9299689211 | 30.0851338253 | 24.4827831593 |
| 1 | GGPN | C | 19.4962486888 | 28.8240724956 | 23.8320070944 |
| 1 | GGPN | O1' | 20.0310189957 | 27.9695359578 | 23.1297844413 |
| 1 | GGPN | C2' | 18.1197651202 | 28.5776515163 | 24.3676542902 |
| 1 | GGPN | H2' | 18.1640982222 | 27.6318790485 | 24.9118944301 |
| 1 | GGPN | H2'' | 17.4874233243 | 28.5015257583 | 23.5034992169 |
| 1 | GGPN | N2' | 17.5441894968 | 29.5813620597 | 25.1969118945 |
| 1 | GGPN | C5' | 17.0060627093 | 30.7800810473 | 24.5387862184 |
| 1 | GGPN | H5' | 17.2068300327 | 31.6172527695 | 25.1872003742 |
| 1 | GGPN | H5'' | 17.6864862683 | 30.8991898550 | 23.7075994088 |
| 1 | GGPN | C6'' | 15.6449317927 | 30.5726398999 | 22.4761843251 |
| 1 | GGPN | H6'' | 16.6768420523 | 30.9393004285 | 22.1950613041 |
| 1 | GGPN | H6''' | 15.7127675459 | 29.4579027176 | 22.4054623303 |
| 1 | GGPN | O7' | 14.8224992884 | 31.2744004009 | 21.3616255509 |
| 1 | GGPN | C8' | 15.7573644324 | 31.6672955480 | 20.2720780292 |
| 1 | GGPN | H8' | 16.6193240984 | 30.9093416240 | 20.2924676977 |
| 1 | GGPN | H8'' | 16.1964966567 | 32.6266411907 | 20.5206341611 |
| 1 | GGPN | C9' | 15.3190136731 | 31.8569102095 | 18.8277873961 |
| 1 | GGPN | H9' | 14.2048063071 | 31.9308009936 | 18.8675469968 |
| 1 | GGPN | H9'' | 15.5925064763 | 30.9670519101 | 18.2109378776 |
| 1 | GGPN | O10' | 15.7875720358 | 33.0242884131 | 18.1190439719 |
| 1 | GGPN | C11' | 15.0044686489 | 33.2003230258 | 16.9272868271 |
| 1 | GGPN | H11' | 13.9540703153 | 33.4087723147 | 17.2281962620 |
| 1 | GGPN | H11'' | 15.0013546380 | 32.2416272602 | 16.3573760026 |
| 1 | GGPN | C12' | 15.4001308231 | 34.3019825850 | 15.9206600842 |
| 1 | GGPN | H12' | 16.4682695804 | 34.1913762347 | 15.6350321695 |
| 1 | GGPN | H12'' | 15.2395196278 | 35.3107732531 | 16.3599975044 |
| 1 | GGPN | O13' | 14.5668688155 | 34.1319046937 | 14.7719733091 |
| 1 | GGPN | H13' | 14.8407730091 | 34.7530309063 | 14.0886163005 |
| 1 | GGPN | C3' | 17.6563100752 | 29.4420373236 | 26.5709678365 |
| 1 | GGPN | O3' | 18.3154808843 | 28.5164254910 | 27.0584160018 |
| 1 | GGPN | C4' | 16.9279221501 | 30.4614335155 | 27.3764103750 |
| 1 | GGPN | H4' | 15.8482992166 | 30.3869436765 | 27.1317271647 |
| 1 | GGPN | H4'' | 17.3171116556 | 31.4718746882 | 27.1366785470 |
| 1 | GGPN | N9 | 17.1532350730 | 30.2978808752 | 28.7997219615 |
| 1 | GGPN | C4 | 18.2185908175 | 30.8272783851 | 29.4682416905 |
| 1 | GGPN | N2 | 21.1444657465 | 32.7116346224 | 29.4942944134 |
| 1 | GGPN | H21 | 21.2178521061 | 33.0815702410 | 28.5733720997 |
| 1 | GGPN | H22 | 21.7272731410 | 33.1335130831 | 30.1943487701 |
| 1 | GGPN | N3 | 19.2079263818 | 31.5622902056 | 28.9138444916 |
| 1 | GGPN | C2 | 20.0974192529 | 31.9655026276 | 29.8257621762 |
| 1 | GGPN | N1 | 19.9958337434 | 31.6294134390 | 31.1565546611 |
| 1 | GGPN | H1 | 20.6888996597 | 31.9820320324 | 31.7939634062 |
| 1 | GGPN | C6 | 18.9810014741 | 30.8679458504 | 31.7386377744 |
| 1 | GGPN | O6 | 18.9446223809 | 30.6177682201 | 32.9471387454 |
| 1 | GGPN | C5 | 18.0286189633 | 30.4625210656 | 30.7814675955 |
| 1 | GGPN | N7 | 16.8640319189 | 29.7155219606 | 30.9293785700 |
| 1 | GGPN | C8 | 16.3845961519 | 29.6299900943 | 29.7243272037 |
| 1 | GGPN | H8 | 15.4778625707 | 29.0927488093 | 29.4441475089 |
| 2 | GGPN | C | 24.4380965260 | 27.6856158575 | 23.9201512108 |
| 2 | GGPN | O1' | 25.2375278102 | 28.3721676009 | 23.2417569705 |
| 2 | GGPN | C2' | 22.9742747769 | 27.8711607412 | 23.8893232406 |
| 2 | GGPN | H2' | 22.5149057192 | 26.9133381970 | 24.1262345998 |
| 2 | GGPN | H2'' | 22.7064411687 | 28.2187884486 | 22.9132300499 |
| 2 | GGPN | N2' | 22.5509737712 | 28.8734126646 | 24.8602021172 |
| 2 | GGPN | C5' | 22.5157133112 | 30.1893271240 | 24.2373434287 |
| 2 | GGPN | H5' | 23.4157150752 | 30.2294880501 | 23.6148476061 |
| 2 | GGPN | H5'' | 22.5402506639 | 30.9437548275 | 25.0025747037 |
| 2 | GGPN | N | 20.1017072539 | 30.0032347439 | 24.0792373133 |
| 2 | GGPN | H1' | 19.8290199513 | 30.6073559249 | 24.8120758592 |
| 2 | GGPN | C6' | 21.2702834351 | 30.4288682438 | 23.3287014533 |
| 2 | GGPN | H6' | 21.3639703191 | 29.8264273472 | 22.4272688673 |
| 2 | GGPN | C6'' | 21.1506906289 | 31.8996579338 | 22.8060120306 |
| 2 | GGPN | H6'' | 21.3311946272 | 31.9466833292 | 21.7048783774 |
| 2 | GGPN | H6''' | 21.9642018693 | 32.4469584994 | 23.3408632292 |
| 2 | GGPN | O7' | 19.7445695286 | 32.5225155091 | 23.1492838370 |

| | | | | | |
|---|---|---|---|---|---|
| 2 | GGPN | C8' | 19.2398787677 | 33.4897781096 | 22.2061766552 |
| 2 | GGPN | H8' | 19.3094657896 | 33.0624933567 | 21.1716029960 |
| 2 | GGPN | H8'' | 19.8356387859 | 34.4242806340 | 22.2335555638 |
| 2 | GGPN | C9' | 17.7016168895 | 33.6970376053 | 22.4915289252 |
| 2 | GGPN | H9' | 17.5148599031 | 33.5693460243 | 23.5919809948 |
| 2 | GGPN | H9'' | 17.3687620683 | 32.7588258363 | 22.0347154722 |
| 2 | GGPN | O10' | 16.8402789343 | 34.6581223446 | 21.9738167602 |
| 2 | GGPN | C11' | 15.4470353490 | 34.3955525493 | 22.4253922313 |
| 2 | GGPN | H11' | 15.2785694116 | 34.9083653322 | 23.3998781387 |
| 2 | GGPN | H11'' | 15.3696289738 | 33.3035494475 | 22.4960199651 |
| 2 | GGPN | C12' | 14.3377640526 | 34.7420896469 | 21.4848063235 |
| 2 | GGPN | H12' | 14.6170278748 | 34.4393381734 | 20.4440445708 |
| 2 | GGPN | H12'' | 14.1548968860 | 35.8490822841 | 21.5093643169 |
| 2 | GGPN | O13' | 13.1604578122 | 34.0474134119 | 21.8871418990 |
| 2 | GGPN | H13' | 12.5141320905 | 34.1781360570 | 21.1824863632 |
| 2 | GGPN | C3' | 22.2246224164 | 28.5711430759 | 26.1621380731 |
| 2 | GGPN | O3' | 22.3186317135 | 27.4166780153 | 26.6093667276 |
| 2 | GGPN | C4' | 21.7572147738 | 29.7022237262 | 27.0975654788 |
| 2 | GGPN | H4' | 20.7536259610 | 30.0635864531 | 26.7958968321 |
| 2 | GGPN | H4'' | 22.4556177446 | 30.5576877523 | 27.0075819275 |
| 2 | GGPN | N9 | 21.7196584751 | 29.2679628105 | 28.4951350206 |
| 2 | GGPN | C4 | 22.6386266955 | 29.5741570982 | 29.4702301252 |
| 2 | GGPN | N2 | 25.4372898709 | 31.3063875263 | 30.5034450490 |
| 2 | GGPN | H21 | 25.8085082273 | 31.5907382199 | 29.6309107961 |
| 2 | GGPN | H22 | 25.8865266559 | 31.5582738821 | 31.3659972695 |
| 2 | GGPN | N3 | 23.7487351879 | 30.3180301126 | 29.3093577087 |
| 2 | GGPN | C2 | 24.3184024684 | 30.6109191404 | 30.4735451371 |
| 2 | GGPN | N1 | 23.8644935403 | 30.1418947469 | 31.6809785668 |
| 2 | GGPN | H1 | 24.2992225314 | 30.4902239271 | 32.5198304301 |
| 2 | GGPN | C6 | 22.7521731536 | 29.3367940522 | 31.8695886595 |
| 2 | GGPN | O6 | 22.4356013915 | 28.9588553956 | 33.0036592990 |
| 2 | GGPN | C5 | 22.1471852666 | 29.0007456347 | 30.6307523694 |
| 2 | GGPN | N7 | 21.0002166157 | 28.2728107057 | 30.3693244938 |
| 2 | GGPN | C8 | 20.7806886127 | 28.4593061500 | 29.0930369049 |
| 2 | GGPN | H8 | 19.9595872089 | 28.0249158627 | 28.5225647072 |
| 3 | GCPN | C | 29.8367797030 | 25.1919602250 | 27.2141097753 |
| 3 | GCPN | O1' | 30.7344909627 | 24.3434781469 | 27.0503534817 |
| 3 | GCPN | C2' | 28.4558636607 | 24.9056652414 | 26.7741370839 |
| 3 | GCPN | H2' | 28.2582823780 | 23.9021100102 | 27.1126224580 |
| 3 | GCPN | H2'' | 28.5295825599 | 25.0002455147 | 25.7056566898 |
| 3 | GCPN | N2' | 27.3401701132 | 25.7537449919 | 27.2540607886 |
| 3 | GCPN | C5' | 26.4207446447 | 26.4789764201 | 26.3880234699 |
| 3 | GCPN | H5' | 26.6626660532 | 27.5331027017 | 26.4584739752 |
| 3 | GCPN | H5'' | 25.4558992884 | 26.2174126558 | 26.7992359250 |
| 3 | GCPN | N | 24.8184310726 | 26.7583989180 | 24.7400861575 |
| 3 | GCPN | H1' | 24.0606310848 | 26.4165549958 | 25.3251120125 |
| 3 | GCPN | C6' | 26.1185026882 | 26.2017724738 | 24.9312183705 |
| 3 | GCPN | H6' | 26.8455397894 | 26.8040794079 | 24.3870777058 |
| 3 | GCPN | C6'' | 26.1949590162 | 24.8038502269 | 24.2931263253 |
| 3 | GCPN | H6'' | 25.2208477744 | 24.2644892229 | 24.3810643455 |
| 3 | GCPN | H6''' | 26.9922112281 | 24.1515609528 | 24.6916083990 |
| 3 | GCPN | O7' | 26.5151491658 | 25.1471058090 | 22.8337951042 |
| 3 | GCPN | C8' | 25.8705382048 | 24.3446442006 | 21.8704157004 |
| 3 | GCPN | H8' | 24.7713761412 | 24.3042079999 | 22.0383601813 |
| 3 | GCPN | H8'' | 26.2975332413 | 23.3203846933 | 21.8999126307 |
| 3 | GCPN | C9' | 26.1629274684 | 25.0113252021 | 20.5231871591 |
| 3 | GCPN | H9' | 27.2535172190 | 25.2277829025 | 20.4515605882 |
| 3 | GCPN | H9'' | 25.6234657138 | 25.9836403414 | 20.4549212161 |
| 3 | GCPN | O10' | 25.7737139097 | 24.1557897934 | 19.4632984699 |
| 3 | GCPN | C11' | 26.1016975263 | 24.7239078610 | 18.2039421536 |
| 3 | GCPN | H11' | 27.1970241415 | 24.9138605861 | 18.1452060286 |
| 3 | GCPN | H11'' | 25.5688736667 | 25.6920599746 | 18.0738507806 |
| 3 | GCPN | C12' | 25.6948066671 | 23.7624792485 | 17.0842284899 |
| 3 | GCPN | H12' | 24.5988036126 | 23.5735136333 | 17.1426653104 |
| 3 | GCPN | H12'' | 26.2317454176 | 22.7957237158 | 17.2175081817 |
| 3 | GCPN | O13' | 26.0323216916 | 24.3499968165 | 15.8339804302 |
| 3 | GCPN | H13' | 25.7711491754 | 23.7335887076 | 15.1434929038 |
| 3 | GCPN | C3' | 27.1081956958 | 25.9631395839 | 28.5752427052 |
| 3 | GCPN | O3' | 27.6386139936 | 25.2447768298 | 29.4451047693 |
| 3 | GCPN | C4' | 26.1900265458 | 27.1370888822 | 29.0347529785 |
| 3 | GCPN | H4' | 25.2625045932 | 27.2113454010 | 28.4432867481 |
| 3 | GCPN | H4'' | 26.7427029559 | 28.0827621975 | 28.8352500118 |
| 3 | GCPN | N1 | 25.7138476682 | 27.0161619700 | 30.4222258316 |

| | | | | | |
|---|---|---|---|---|---|
| 3 | GCPN | C6 | 24.6899410145 | 26.1663618569 | 30.7363295199 |
| 3 | GCPN | H6 | 24.2422578331 | 25.6333442819 | 29.8980312889 |
| 3 | GCPN | C5 | 24.2736426544 | 26.0204253718 | 32.0057662623 |
| 3 | GCPN | H5 | 23.4739165023 | 25.3361412586 | 32.2741937198 |
| 3 | GCPN | C2 | 26.3598773199 | 27.7389768952 | 31.4165671229 |
| 3 | GCPN | O2 | 27.3189116538 | 28.4691773208 | 31.1143195865 |
| 3 | GCPN | N3 | 25.9355920769 | 27.6238910997 | 32.6954904785 |
| 3 | GCPN | C4 | 24.9308762078 | 26.7842296070 | 33.0128159505 |
| 3 | GCPN | N4 | 24.5615168035 | 26.6550753536 | 34.2944211066 |
| 3 | GCPN | H41 | 25.0395908724 | 27.1866654406 | 35.0012379112 |
| 3 | GCPN | H42 | 23.7948986365 | 26.0844267025 | 34.5678933941 |
| 4 | GAPN | C | 33.8957754221 | 24.1934844525 | 30.2279616555 |
| 4 | GAPN | O1' | 34.8519930761 | 23.5685980801 | 29.7432780094 |
| 4 | GAPN | C2' | 32.6846522208 | 24.4523934872 | 29.4549874653 |
| 4 | GAPN | H2' | 32.1478137024 | 23.5196929367 | 29.3806391630 |
| 4 | GAPN | H2'' | 33.0778169986 | 24.7956957400 | 28.5182662617 |
| 4 | GAPN | N2' | 31.8182922688 | 25.5056539758 | 30.0264638292 |
| 4 | GAPN | C5' | 31.8010436949 | 26.8479441962 | 29.4127035142 |
| 4 | GAPN | H5' | 32.7928280165 | 27.2764969056 | 29.4974334750 |
| 4 | GAPN | H5'' | 31.0604705616 | 27.4790658632 | 29.8737070025 |
| 4 | GAPN | N | 30.0938747718 | 26.3740672248 | 27.7392541403 |
| 4 | GAPN | H1' | 29.3204103503 | 26.9781780790 | 27.9534526972 |
| 4 | GAPN | C6' | 31.4258380539 | 26.8528180715 | 27.9361636097 |
| 4 | GAPN | H6' | 32.1083916378 | 26.1598860106 | 27.4499240814 |
| 4 | GAPN | C6'' | 31.6844728817 | 28.2006288623 | 27.2368084973 |
| 4 | GAPN | H6'' | 32.3423908656 | 28.8780672539 | 27.8263201007 |
| 4 | GAPN | H6''' | 30.7140323002 | 28.7142055183 | 27.0489367966 |
| 4 | GAPN | O7' | 32.3810479336 | 27.8508888473 | 25.9273792727 |
| 4 | GAPN | C8' | 31.9122268158 | 28.5595267249 | 24.7974456005 |
| 4 | GAPN | H8' | 32.2632935819 | 29.6142860876 | 24.8240764346 |
| 4 | GAPN | H8'' | 30.8022057943 | 28.5357451530 | 24.7072993576 |
| 4 | GAPN | C9' | 32.5466291758 | 27.8045265819 | 23.6350865646 |
| 4 | GAPN | H9' | 32.0995478605 | 26.7863522816 | 23.5641050253 |
| 4 | GAPN | H9'' | 33.6363566295 | 27.6835785139 | 23.8364096533 |
| 4 | GAPN | O10' | 32.3543878453 | 28.5167197953 | 22.4295387027 |
| 4 | GAPN | C11' | 32.9946178025 | 27.8609001061 | 21.3467196737 |
| 4 | GAPN | H11' | 32.5678201498 | 26.8421629262 | 21.2101893411 |
| 4 | GAPN | H11'' | 34.0853412668 | 27.7631912241 | 21.5477023531 |
| 4 | GAPN | C12' | 32.7794133062 | 28.6859360404 | 20.0780703317 |
| 4 | GAPN | H12' | 33.2006571687 | 29.7060830337 | 20.2293240097 |
| 4 | GAPN | H12'' | 31.6865648773 | 28.7767138906 | 19.8820776205 |
| 4 | GAPN | O13' | 33.4293222056 | 28.0349594768 | 18.9947292438 |
| 4 | GAPN | H13' | 33.2626497246 | 28.5562092313 | 18.2039356060 |
| 4 | GAPN | C3' | 30.9180282771 | 25.1012242975 | 30.9729530444 |
| 4 | GAPN | O3' | 30.9441624714 | 23.9704278605 | 31.4688070005 |
| 4 | GAPN | C4' | 29.8864660898 | 26.1020576875 | 31.4713125512 |
| 4 | GAPN | H4' | 29.2611845377 | 26.4472851362 | 30.6307657527 |
| 4 | GAPN | H4'' | 30.4548168232 | 26.9793163468 | 31.8534483817 |
| 4 | GAPN | N9 | 29.0749240725 | 25.5571696694 | 32.5567220424 |
| 4 | GAPN | C5 | 28.3413738533 | 25.1440190382 | 34.5710475691 |
| 4 | GAPN | N7 | 27.4470467890 | 24.5287852886 | 33.7108046759 |
| 4 | GAPN | C8 | 27.9302424232 | 24.7879827375 | 32.5224572079 |
| 4 | GAPN | H8 | 27.4881181894 | 24.4343284179 | 31.5897263546 |
| 4 | GAPN | N1 | 29.4108269670 | 25.9744542625 | 36.4852296078 |
| 4 | GAPN | C2 | 30.3599305787 | 26.4577905862 | 35.6850213751 |
| 4 | GAPN | H2 | 31.1797043287 | 26.9915836276 | 36.1781265235 |
| 4 | GAPN | N3 | 30.4244732956 | 26.4066569543 | 34.3573631971 |
| 4 | GAPN | C4 | 29.3620582971 | 25.7494518351 | 33.8692001206 |
| 4 | GAPN | C6 | 28.3614406867 | 25.2920875883 | 35.9650451997 |
| 4 | GAPN | N6 | 27.3829999910 | 24.8003733592 | 36.7476415083 |
| 4 | GAPN | H61 | 26.6475244242 | 24.2541764310 | 36.3601129579 |
| 4 | GAPN | H62 | 27.3275371571 | 25.0435828560 | 37.7260744898 |
| 5 | GTPN | C | 37.2691640221 | 22.3817736796 | 34.1261518981 |
| 5 | GTPN | O1' | 37.9671595716 | 21.3521919243 | 33.9717317955 |
| 5 | GTPN | C2' | 36.2658603981 | 22.7875106449 | 33.1514604355 |
| 5 | GTPN | H2' | 35.7777464476 | 21.9037697114 | 32.7743649335 |
| 5 | GTPN | H2'' | 36.8319281911 | 23.2962073430 | 32.3935662316 |
| 5 | GTPN | N2' | 35.2756177991 | 23.7260299224 | 33.7056509482 |
| 5 | GTPN | C5' | 35.3554028752 | 25.1396487216 | 33.3237835618 |
| 5 | GTPN | H5' | 36.3506324111 | 25.4566426712 | 33.5793511119 |
| 5 | GTPN | H5'' | 34.6042091998 | 25.7550732063 | 33.7858742648 |
| 5 | GTPN | N | 33.9420486499 | 24.7229824533 | 31.4264984647 |
| 5 | GTPN | H1' | 33.0908020694 | 25.0251839229 | 31.8745870132 |

| | | | | | |
|---|---|---|---|---|---|
| 5 | GTPN | C6' | 35.1608428460 | 25.3084453399 | 31.8396064085 |
| 5 | GTPN | H6' | 35.9853060732 | 24.7554282135 | 31.4043310977 |
| 5 | GTPN | C6'' | 35.2765507598 | 26.7163968313 | 31.2352200885 |
| 5 | GTPN | H6'' | 35.7988555993 | 27.4079645262 | 31.9215837742 |
| 5 | GTPN | H6''' | 34.2567119950 | 27.1185619720 | 31.0523997965 |
| 5 | GTPN | O7' | 36.0531673159 | 26.4779607348 | 29.9183339086 |
| 5 | GTPN | C8' | 35.9413977800 | 27.4358724880 | 28.8763976937 |
| 5 | GTPN | H8' | 36.0112927771 | 28.4734176239 | 29.2640391993 |
| 5 | GTPN | H8'' | 34.9880389344 | 27.3202084566 | 28.3124142429 |
| 5 | GTPN | C9' | 37.1067654108 | 27.0864899702 | 27.9303226001 |
| 5 | GTPN | H9' | 36.8700689818 | 26.1396756873 | 27.3965194903 |
| 5 | GTPN | H9'' | 38.0416580756 | 26.8767969672 | 28.4819366796 |
| 5 | GTPN | O10' | 37.3861165209 | 28.1287776875 | 27.0211863150 |
| 5 | GTPN | C11' | 38.5813054184 | 27.8603110425 | 26.2941919199 |
| 5 | GTPN | H11' | 38.5298410024 | 26.8521511050 | 25.8223220987 |
| 5 | GTPN | H11'' | 39.4665807351 | 27.8815123966 | 26.9706282597 |
| 5 | GTPN | C12' | 38.7295195812 | 28.9344601956 | 25.2170555707 |
| 5 | GTPN | H12' | 38.9339027817 | 29.9132759355 | 25.7074872614 |
| 5 | GTPN | H12'' | 37.7688812564 | 29.0075382248 | 24.6574627662 |
| 5 | GTPN | O13' | 39.7843821773 | 28.5911975776 | 24.3293032431 |
| 5 | GTPN | H13' | 39.7869026494 | 29.2490554494 | 23.6264455292 |
| 5 | GTPN | C3' | 34.1774552049 | 23.1543166973 | 34.2610545270 |
| 5 | GTPN | O3' | 34.1036530428 | 21.9391780857 | 34.4605175560 |
| 5 | GTPN | C4' | 33.0394843368 | 24.0994323339 | 34.6201362575 |
| 5 | GTPN | H4' | 32.6149452184 | 24.4786889558 | 33.6676702542 |
| 5 | GTPN | H4'' | 33.4714597340 | 24.9574918817 | 35.1786728990 |
| 5 | GTPN | N1 | 31.9685897263 | 23.4938387419 | 35.3897485093 |
| 5 | GTPN | C6 | 30.9605802009 | 22.7778857453 | 34.7609866334 |
| 5 | GTPN | H6 | 31.0630307417 | 22.7217990335 | 33.6691944548 |
| 5 | GTPN | C2 | 32.0208593203 | 23.5769572714 | 36.7585912409 |
| 5 | GTPN | O2 | 32.9684428837 | 24.1275920312 | 37.3602597918 |
| 5 | GTPN | N3 | 30.9741410072 | 23.0199156341 | 37.3941708760 |
| 5 | GTPN | H3 | 30.9696828022 | 23.1395396937 | 38.4011836991 |
| 5 | GTPN | C4 | 29.8698270088 | 22.3396040790 | 36.8526625123 |
| 5 | GTPN | O4 | 28.9941792706 | 21.9416015222 | 37.5492796828 |
| 5 | GTPN | C5 | 29.9577267759 | 22.2340862677 | 35.3980811990 |
| 5 | GTPN | C5M | 28.8697178909 | 21.4968737888 | 34.6707181752 |
| 5 | GTPN | H51 | 29.0581630411 | 21.5266047134 | 33.5726404624 |
| 5 | GTPN | H52 | 27.8766329898 | 21.9608604245 | 34.8555912506 |
| 5 | GTPN | H53 | 28.8289958994 | 20.4320681571 | 34.9851803288 |
| 6 | GGPN | C | 39.2845569293 | 19.3383992864 | 38.3131558449 |
| 6 | GGPN | O1' | 40.2979650427 | 18.6261602687 | 38.4759072274 |
| 6 | GGPN | C2' | 38.9155118326 | 19.9734813857 | 37.0037214016 |
| 6 | GGPN | H2' | 38.4560230469 | 19.2172717394 | 36.3795817536 |
| 6 | GGPN | H2'' | 39.8234551066 | 20.3429210303 | 36.5806433229 |
| 6 | GGPN | N2' | 37.9778274964 | 21.1459221197 | 37.1752277067 |
| 6 | GGPN | C5' | 38.4176106782 | 22.5311988752 | 37.2036447179 |
| 6 | GGPN | H5' | 39.3569319692 | 22.6226469673 | 37.6970189001 |
| 6 | GGPN | H5'' | 37.6870799244 | 23.1620340663 | 37.6777544272 |
| 6 | GGPN | N | 37.4487725402 | 23.1787474930 | 35.1484845396 |
| 6 | GGPN | H1' | 36.7703977200 | 23.9044285950 | 35.3502701451 |
| 6 | GGPN | C6' | 38.6599932425 | 23.2205294931 | 35.8798399295 |
| 6 | GGPN | H6' | 39.4252976255 | 22.6378833137 | 35.3757385745 |
| 6 | GGPN | C6'' | 39.2065759497 | 24.6561257564 | 36.0223705657 |
| 6 | GGPN | H6'' | 40.2912760203 | 24.6606392676 | 35.7882398941 |
| 6 | GGPN | H6''' | 39.0863683670 | 25.0672141369 | 37.0563359089 |
| 6 | GGPN | O7' | 38.4588114587 | 25.5757916464 | 35.0279444290 |
| 6 | GGPN | C8' | 39.3516224829 | 26.1720248928 | 34.0960657764 |
| 6 | GGPN | H8' | 40.2112250123 | 25.5083761789 | 33.8491735709 |
| 6 | GGPN | H8'' | 39.7406562322 | 27.1233678175 | 34.5236339186 |
| 6 | GGPN | C9' | 38.6153804071 | 26.4862574234 | 32.8046300969 |
| 6 | GGPN | H9' | 37.6303829698 | 26.9248855693 | 33.0581612004 |
| 6 | GGPN | H9'' | 38.4616918648 | 25.5684633707 | 32.1905525549 |
| 6 | GGPN | O10' | 39.3287441030 | 27.4554385241 | 32.0817233714 |
| 6 | GGPN | C11' | 38.5048186392 | 27.9247086218 | 31.0453094910 |
| 6 | GGPN | H11' | 37.5290074572 | 28.2686589402 | 31.4480486369 |
| 6 | GGPN | H11'' | 38.3184867150 | 27.0745322674 | 30.3747598870 |
| 6 | GGPN | C12' | 39.1272279282 | 29.1507813161 | 30.4086494825 |
| 6 | GGPN | H12' | 40.0734418482 | 28.8958051396 | 29.8841558376 |
| 6 | GGPN | H12'' | 39.3582790456 | 29.8662718332 | 31.2342653647 |
| 6 | GGPN | O13' | 38.1742547001 | 29.7293454363 | 29.5286754956 |
| 6 | GGPN | H13' | 38.0289254462 | 29.1063947118 | 28.8005540476 |
| 6 | GGPN | C3' | 36.6486386094 | 20.8659540361 | 37.3728457849 |

| | | | | | |
|---|---|---|---|---|---|
| 6 | GGPN | O3' | 36.2665942986 | 19.6912251215 | 37.4597265712 |
| 6 | GGPN | C4' | 35.6124236316 | 21.9833212293 | 37.5937108252 |
| 6 | GGPN | H4' | 35.4535160449 | 22.5645549011 | 36.6687011850 |
| 6 | GGPN | H4'' | 35.9166028611 | 22.6737873486 | 38.4034973217 |
| 6 | GGPN | N9 | 34.3820614532 | 21.3470491954 | 38.0126667661 |
| 6 | GGPN | C4 | 33.9238306090 | 21.2271553169 | 39.2946242782 |
| 6 | GGPN | N2 | 34.3472157384 | 21.5972877154 | 42.7139382555 |
| 6 | GGPN | H21 | 35.2250297360 | 22.0551942735 | 42.7732175434 |
| 6 | GGPN | H22 | 33.8992684056 | 21.2583710715 | 43.5489926108 |
| 6 | GGPN | N3 | 34.5339940801 | 21.6773148623 | 40.4143856911 |
| 6 | GGPN | C2 | 33.8530082236 | 21.3530953868 | 41.5110146238 |
| 6 | GGPN | N1 | 32.6430621229 | 20.7023883948 | 41.4782863572 |
| 6 | GGPN | H1 | 32.1763931616 | 20.5121290241 | 42.3503944521 |
| 6 | GGPN | C6 | 31.9896977169 | 20.2598925268 | 40.3382657204 |
| 6 | GGPN | O6 | 30.9029681488 | 19.6715512725 | 40.4148199785 |
| 6 | GGPN | C5 | 32.7451335052 | 20.5163033206 | 39.1762824082 |
| 6 | GGPN | N7 | 32.5195416977 | 20.1392569542 | 37.8613131965 |
| 6 | GGPN | C8 | 33.5263161901 | 20.6503584359 | 37.2062321495 |
| 6 | GGPN | H8 | 33.7419902295 | 20.5414987403 | 36.1425514879 |
| 7 | GCPN | C | 38.8330361707 | 15.9648063292 | 42.9214668945 |
| 7 | GCPN | O1' | 39.6794941854 | 14.8826629483 | 43.1480895403 |
| 7 | GCPN | C2' | 39.1197999984 | 16.7683921404 | 41.7565213527 |
| 7 | GCPN | H2' | 39.1766984249 | 16.1514543660 | 40.8802855238 |
| 7 | GCPN | H2'' | 40.0662076119 | 17.2426557021 | 41.9510118968 |
| 7 | GCPN | N2' | 38.1812061794 | 17.9466592633 | 41.4479311090 |
| 7 | GCPN | C5' | 38.5058991033 | 19.3128390548 | 41.7522217527 |
| 7 | GCPN | H5' | 39.4153392237 | 19.3350223340 | 42.3454144767 |
| 7 | GCPN | H5'' | 37.7162231706 | 19.7452985296 | 42.3515406464 |
| 7 | GCPN | N | 38.4290849231 | 19.6352870368 | 39.2641940314 |
| 7 | GCPN | H1' | 37.5027878528 | 19.6441302005 | 38.8367346908 |
| 7 | GCPN | C6' | 38.6336450315 | 20.3247074739 | 40.5436047936 |
| 7 | GCPN | H6' | 37.7259075544 | 20.9225568280 | 40.5900108603 |
| 7 | GCPN | C6'' | 39.7349338533 | 21.4454267573 | 40.6594594778 |
| 7 | GCPN | H6'' | 40.5348991906 | 21.2494130987 | 41.4054133049 |
| 7 | GCPN | H6''' | 39.2350776809 | 22.4063032467 | 40.9589750063 |
| 7 | GCPN | O7' | 40.3278136881 | 21.6402818554 | 39.2596398374 |
| 7 | GCPN | C8' | 41.6584784306 | 22.1103750913 | 39.1298654077 |
| 7 | GCPN | H8' | 42.3239056931 | 21.5216162712 | 39.7838450191 |
| 7 | GCPN | H8'' | 41.6990745542 | 23.1922784328 | 39.3783930699 |
| 7 | GCPN | C9' | 42.1085685367 | 21.9333713741 | 37.6600059018 |
| 7 | GCPN | H9' | 41.2409376582 | 21.6372704630 | 37.0467616571 |
| 7 | GCPN | H9'' | 42.8691845238 | 21.1269736954 | 37.5740728998 |
| 7 | GCPN | O10' | 42.6114912027 | 23.1388195033 | 37.1232456758 |
| 7 | GCPN | C11' | 42.6206334822 | 23.1229944991 | 35.7032527478 |
| 7 | GCPN | H11' | 41.5941246772 | 22.9606771184 | 35.3035429533 |
| 7 | GCPN | H11'' | 43.2813679767 | 22.3197142475 | 35.3062206122 |
| 7 | GCPN | C12' | 43.0939093068 | 24.5070426420 | 35.2888594329 |
| 7 | GCPN | H12' | 44.1533252678 | 24.6613012436 | 35.5995886469 |
| 7 | GCPN | H12'' | 42.4634464999 | 25.2690858105 | 35.8049441796 |
| 7 | GCPN | O13' | 42.9392149545 | 24.6234616656 | 33.8857037138 |
| 7 | GCPN | H13' | 43.3979293687 | 23.8714638648 | 33.4973202886 |
| 7 | GCPN | C3' | 36.9534493452 | 17.5611364948 | 41.0015831954 |
| 7 | GCPN | O3' | 36.6644648661 | 16.4098616630 | 40.7288981554 |
| 7 | GCPN | C4' | 35.8745855631 | 18.6570409411 | 40.8834981973 |
| 7 | GCPN | H4' | 36.0083900388 | 19.2094840121 | 39.9343253715 |
| 7 | GCPN | H4'' | 35.9079256887 | 19.3886626897 | 41.6998697510 |
| 7 | GCPN | N1 | 34.5645774922 | 18.0365074005 | 40.9453032931 |
| 7 | GCPN | C6 | 33.9837537937 | 17.6150830255 | 39.7996701220 |
| 7 | GCPN | H6 | 34.5468219018 | 17.8451289951 | 38.8886711396 |
| 7 | GCPN | C5 | 32.7841234501 | 17.0270899564 | 39.8233223669 |
| 7 | GCPN | H5 | 32.2872003851 | 16.7339545070 | 38.9058364122 |
| 7 | GCPN | C2 | 34.0086114308 | 17.7491538983 | 42.2005726857 |
| 7 | GCPN | O2 | 34.6385943984 | 18.0216459084 | 43.2377515328 |
| 7 | GCPN | N3 | 32.7793188560 | 17.1935695020 | 42.2424117789 |
| 7 | GCPN | C4 | 32.1603239206 | 16.8508647283 | 41.1000287923 |
| 7 | GCPN | N4 | 30.9329759900 | 16.3471906082 | 41.1619305609 |
| 7 | GCPN | H41 | 30.4861789194 | 16.3537098460 | 42.0642629079 |
| 7 | GCPN | H42 | 30.3537948320 | 16.3619850141 | 40.3568164222 |
| 8 | GCPN | C | 36.9140302944 | 11.7140541483 | 46.8448276363 |
| 8 | GCPN | C2' | 37.6397963852 | 12.5920940302 | 45.8141209710 |
| 8 | GCPN | H2' | 38.0835400128 | 11.9281023294 | 45.0842240823 |
| 8 | GCPN | H2'' | 38.3909484337 | 13.0964251837 | 46.3984684278 |
| 8 | GCPN | N2' | 36.8801269564 | 13.6558535450 | 45.1366878377 |

| | | | | | |
|---|---|---|---|---|---|
| 8 | GCPN | C5' | 36.9151769020 | 14.9703435054 | 45.7646111681 |
| 8 | GCPN | H5' | 37.0814994873 | 14.8033016383 | 46.8306424796 |
| 8 | GCPN | H5'' | 35.9750870528 | 15.4717366457 | 45.5928849831 |
| 8 | GCPN | N | 37.9421179153 | 16.1383127838 | 43.8298567486 |
| 8 | GCPN | H1' | 37.1622643779 | 16.7868437046 | 43.5847545749 |
| 8 | GCPN | C6' | 38.0625517558 | 15.8852538304 | 45.2232356700 |
| 8 | GCPN | H6' | 38.9881046749 | 15.3307552451 | 45.4125198214 |
| 8 | GCPN | C6'' | 38.2486306806 | 17.2332848112 | 45.9517821644 |
| 8 | GCPN | H6'' | 38.9651845698 | 17.1229829533 | 46.7891446344 |
| 8 | GCPN | H6''' | 37.2496131987 | 17.5483915130 | 46.3402746889 |
| 8 | GCPN | O7' | 38.7581858491 | 18.3044584336 | 44.9191361098 |
| 8 | GCPN | C8' | 39.8992863544 | 19.0434906049 | 45.3623053932 |
| 8 | GCPN | H8' | 40.1789149304 | 18.7779431593 | 46.3987606922 |
| 8 | GCPN | H8'' | 39.5819770806 | 20.1146459150 | 45.3509663426 |
| 8 | GCPN | C9' | 41.1463286015 | 18.9906263742 | 44.4463153712 |
| 8 | GCPN | H9' | 42.0413741401 | 18.5003573787 | 44.8838917331 |
| 8 | GCPN | H9'' | 40.8479619630 | 18.4547984573 | 43.5195635004 |
| 8 | GCPN | O10' | 41.4944734295 | 20.3326479177 | 44.1869876621 |
| 8 | GCPN | C11' | 42.4373034807 | 20.5150526283 | 43.1491829904 |
| 8 | GCPN | H11' | 43.4569366937 | 20.1366625614 | 43.3948660873 |
| 8 | GCPN | H11'' | 42.0595563566 | 20.0396859627 | 42.2160791950 |
| 8 | GCPN | C12' | 42.5403120844 | 22.0366106844 | 43.1018483333 |
| 8 | GCPN | H12' | 43.2183165033 | 22.3580486506 | 43.9252361725 |
| 8 | GCPN | H12'' | 41.5170979661 | 22.4426317120 | 43.2991335573 |
| 8 | GCPN | O13' | 43.0333717886 | 22.5026532832 | 41.8635394235 |
| 8 | GCPN | H13' | 43.3735390605 | 23.3928855161 | 42.0117008190 |
| 8 | GCPN | C3' | 36.2618357272 | 13.4001258153 | 43.9218074716 |
| 8 | GCPN | O3' | 36.2941036503 | 12.3144949352 | 43.3454595636 |
| 8 | GCPN | C4' | 35.4749266930 | 14.5124500677 | 43.1689790717 |
| 8 | GCPN | H4' | 36.1293805162 | 14.7878405551 | 42.3204933622 |
| 8 | GCPN | H4'' | 35.3503336365 | 15.4131479438 | 43.7887214619 |
| 8 | GCPN | N1 | 34.1734268628 | 14.0795239006 | 42.6396368898 |
| 8 | GCPN | C6 | 33.9776680685 | 13.8612215612 | 41.2991757631 |
| 8 | GCPN | H6 | 34.8545901297 | 14.0415548923 | 40.6747481305 |
| 8 | GCPN | C5 | 32.7786288191 | 13.4790094686 | 40.8316279771 |
| 8 | GCPN | H5 | 32.5910521300 | 13.3285606324 | 39.7673758119 |
| 8 | GCPN | C2 | 33.1103742739 | 13.9722774148 | 43.5201014818 |
| 8 | GCPN | O2 | 33.2691850905 | 14.3114708115 | 44.7161979745 |
| 8 | GCPN | N3 | 31.9153409892 | 13.5402261950 | 43.0826202206 |
| 8 | GCPN | C4 | 31.7213853036 | 13.2941003085 | 41.7737205778 |
| 8 | GCPN | N4 | 30.4972030480 | 12.9186945111 | 41.3952631168 |
| 8 | GCPN | H41 | 29.7846969685 | 12.9432093568 | 42.1047628791 |
| 8 | GCPN | H42 | 30.2139504576 | 12.8695054135 | 40.4421058491 |
| 8 | GCPN | OT1 | 35.6905947457 | 11.8974231435 | 47.0747988017 |
| 8 | GCPN | OT2 | 37.6169975238 | 10.8525175729 | 47.4441429187 |
| 9 | GGPN | N | 23.4580837796 | 13.8314544382 | 47.9372905093 |
| 9 | GGPN | HT1 | 23.5327163847 | 12.7924589747 | 47.9273287948 |
| 9 | GGPN | HT2 | 23.9231170195 | 14.2070305040 | 47.0831355449 |
| 9 | GGPN | HT3 | 22.4517998131 | 14.0955025360 | 47.9094427171 |
| 9 | GGPN | C6' | 24.1114680473 | 14.3434628028 | 49.1881880677 |
| 9 | GGPN | H6' | 23.5697304172 | 13.9117685208 | 50.0286946264 |
| 9 | GGPN | C | 27.1037424650 | 15.7499617178 | 51.5249610893 |
| 9 | GGPN | O1' | 26.7135972641 | 16.7510671988 | 50.9282450780 |
| 9 | GGPN | C2' | 26.3663128102 | 14.4529829011 | 51.5204044532 |
| 9 | GGPN | H2' | 25.3258311742 | 14.6359455337 | 51.7331456915 |
| 9 | GGPN | H2'' | 26.8028948139 | 13.8742291683 | 52.3202067706 |
| 9 | GGPN | N2' | 26.4767255266 | 13.6139598468 | 50.3276591772 |
| 9 | GGPN | C5' | 25.5678863011 | 13.7951859520 | 49.2093634672 |
| 9 | GGPN | H5' | 26.0289618508 | 14.7810548505 | 49.2308696763 |
| 9 | GGPN | H5'' | 25.1915255132 | 12.9160677007 | 49.7483296665 |
| 9 | GGPN | C6'' | 23.9043296059 | 15.8687010118 | 49.2911513205 |
| 9 | GGPN | H6'' | 23.3439981905 | 16.2706442377 | 48.4191174392 |
| 9 | GGPN | H6''' | 24.8352941387 | 16.4696463350 | 49.3634818622 |
| 9 | GGPN | O7' | 23.0639426852 | 16.1242148491 | 50.5261398498 |
| 9 | GGPN | C8' | 22.7044943146 | 17.4854018525 | 50.5298419353 |
| 9 | GGPN | H8' | 22.0999706147 | 17.7046302835 | 49.6192320842 |
| 9 | GGPN | H8'' | 23.6221262568 | 18.1155288628 | 50.5121653424 |
| 9 | GGPN | C9' | 21.8698080835 | 17.8367281337 | 51.7432560324 |
| 9 | GGPN | H9' | 22.4861400571 | 17.8128615771 | 52.6687778585 |
| 9 | GGPN | H9'' | 21.0248177194 | 17.1204128670 | 51.8485653207 |
| 9 | GGPN | O10' | 21.3689156531 | 19.1369249436 | 51.5162661905 |
| 9 | GGPN | C11' | 20.5353064133 | 19.5196073359 | 52.5887906063 |
| 9 | GGPN | H11' | 21.1220124432 | 19.4984782945 | 53.5334022023 |

| | | | | | |
|---|---|---|---|---|---|
| 9 | GGPN | H11'' | 19.6916795272 | 18.7994118582 | 52.6762176447 |
| 9 | GGPN | C12' | 19.9827384992 | 20.9237110216 | 52.3633081867 |
| 9 | GGPN | H12' | 19.3873220827 | 20.9467200173 | 51.4221030105 |
| 9 | GGPN | H12'' | 20.8254297344 | 21.6471362031 | 52.2773439527 |
| 9 | GGPN | O13' | 19.1653115127 | 21.2336124023 | 53.4833775913 |
| 9 | GGPN | H13' | 18.8139313230 | 22.1186866456 | 53.3539272352 |
| 9 | GGPN | C3' | 27.5830517510 | 12.7959799244 | 50.2431176102 |
| 9 | GGPN | O3' | 28.5477232007 | 12.9482287572 | 50.9849004292 |
| 9 | GGPN | C4' | 27.5791145270 | 11.6107239188 | 49.2594545277 |
| 9 | GGPN | H4' | 26.7478306387 | 10.9463602727 | 49.5770096758 |
| 9 | GGPN | H4'' | 28.5331578564 | 11.0582521640 | 49.4078142010 |
| 9 | GGPN | N9 | 27.4618026374 | 11.9457197994 | 47.8396956087 |
| 9 | GGPN | C4 | 28.4502778573 | 12.5032102792 | 47.0858343775 |
| 9 | GGPN | N2 | 31.7588644627 | 13.4308741642 | 46.8146631438 |
| 9 | GGPN | H21 | 32.0630202188 | 13.3531199557 | 47.7586307580 |
| 9 | GGPN | H22 | 32.4488967584 | 13.5795995108 | 46.0982691247 |
| 9 | GGPN | N3 | 29.6719570732 | 12.8140393620 | 47.5290096808 |
| 9 | GGPN | C2 | 30.5010969452 | 13.1305198618 | 46.5459520010 |
| 9 | GGPN | N1 | 30.1088325894 | 13.1867579603 | 45.2267492523 |
| 9 | GGPN | H1 | 30.8061660597 | 13.3811171331 | 44.5258093855 |
| 9 | GGPN | C6 | 28.8254252214 | 12.9264327042 | 44.7457781674 |
| 9 | GGPN | O6 | 28.5560801720 | 12.9981551868 | 43.5391735709 |
| 9 | GGPN | C5 | 27.9377565006 | 12.5888038873 | 45.8051186470 |
| 9 | GGPN | N7 | 26.6155741765 | 12.1397652674 | 45.7785753975 |
| 9 | GGPN | C8 | 26.3978265304 | 11.7341915360 | 46.9954559093 |
| 9 | GGPN | H8 | 25.4805495543 | 11.2564001160 | 47.3347508112 |
| 10 | GGPN | C | 30.4184874721 | 21.0072825805 | 51.4975302862 |
| 10 | GGPN | O1' | 30.4010658514 | 22.1774545044 | 51.9545783222 |
| 10 | GGPN | C2' | 29.1579777708 | 20.2161405710 | 51.4866324427 |
| 10 | GGPN | H2' | 28.5431063050 | 20.5874328953 | 50.6799264425 |
| 10 | GGPN | H2'' | 28.7203527037 | 20.4451441911 | 52.4427810951 |
| 10 | GGPN | N2' | 29.2469016995 | 18.7311279129 | 51.4187103202 |
| 10 | GGPN | C5' | 29.0316355902 | 18.1372419035 | 52.7435192515 |
| 10 | GGPN | H5' | 28.1459732374 | 18.6010222767 | 53.1582998669 |
| 10 | GGPN | H5'' | 29.9163126989 | 18.4420351972 | 53.2893204131 |
| 10 | GGPN | N | 28.2065929852 | 15.6718267023 | 52.2606413737 |
| 10 | GGPN | H1' | 28.4179075785 | 14.7324116703 | 52.5574686233 |
| 10 | GGPN | C6' | 28.8957054796 | 16.6162852875 | 53.0720595563 |
| 10 | GGPN | H6' | 29.9118123712 | 16.2500280562 | 53.0990681426 |
| 10 | GGPN | C6'' | 28.3382451981 | 16.3349782208 | 54.4811467910 |
| 10 | GGPN | H6'' | 27.2536444060 | 16.6112857025 | 54.4955518320 |
| 10 | GGPN | H6''' | 28.8577502227 | 16.8455426035 | 55.3141592532 |
| 10 | GGPN | O7' | 28.4838877242 | 14.7991022854 | 54.5671531768 |
| 10 | GGPN | C8' | 27.7880574719 | 14.1809344782 | 55.6221149975 |
| 10 | GGPN | H8' | 26.7824004308 | 14.6242592471 | 55.7983467392 |
| 10 | GGPN | H8'' | 28.3893151700 | 14.2603371518 | 56.5540513848 |
| 10 | GGPN | C9' | 27.6065899089 | 12.7266184463 | 55.2034399001 |
| 10 | GGPN | H9' | 28.5413816665 | 12.2921031449 | 54.7800049295 |
| 10 | GGPN | H9'' | 26.8026750862 | 12.6357087826 | 54.4381410072 |
| 10 | GGPN | O10' | 27.2457336601 | 12.0531645825 | 56.3835475320 |
| 10 | GGPN | C11' | 26.9954596158 | 10.6854520649 | 56.1479559140 |
| 10 | GGPN | H11' | 27.9136088405 | 10.1781284727 | 55.7770955543 |
| 10 | GGPN | H11'' | 26.1832496959 | 10.5646132503 | 55.3962487714 |
| 10 | GGPN | C12' | 26.5628921510 | 10.0980689601 | 57.4833400052 |
| 10 | GGPN | H12' | 25.7154801086 | 10.6980310751 | 57.8915705925 |
| 10 | GGPN | H12'' | 27.4118373984 | 10.1459658500 | 58.2045601170 |
| 10 | GGPN | O13' | 26.1634649243 | 8.7575074572 | 57.2443025398 |
| 10 | GGPN | H13' | 25.8641923949 | 8.3912871110 | 58.0806174277 |
| 10 | GGPN | C3' | 29.5314326212 | 18.2111315515 | 50.1794577222 |
| 10 | GGPN | O3' | 29.4881977503 | 18.9822365155 | 49.2208372214 |
| 10 | GGPN | C4' | 29.9907449493 | 16.7852859720 | 49.7496780674 |
| 10 | GGPN | H4' | 29.5488011682 | 15.9965777797 | 50.3564130910 |
| 10 | GGPN | H4'' | 31.0868178285 | 16.7452200907 | 49.9041615365 |
| 10 | GGPN | N9 | 29.6448969691 | 16.5438266374 | 48.3421629372 |
| 10 | GGPN | C4 | 30.4643604537 | 16.6784871877 | 47.2389491259 |
| 10 | GGPN | N2 | 33.5470106664 | 17.4132087443 | 45.8066259190 |
| 10 | GGPN | H21 | 34.1257877378 | 17.5616810368 | 46.5975122545 |
| 10 | GGPN | H22 | 33.9153418251 | 17.5125035968 | 44.8778183559 |
| 10 | GGPN | N3 | 31.7660434006 | 17.0429606735 | 47.2447493162 |
| 10 | GGPN | C2 | 32.2826783086 | 17.0753020065 | 46.0133902432 |
| 10 | GGPN | N1 | 31.5399957649 | 16.8539567833 | 44.8814276558 |
| 10 | GGPN | H1 | 32.0104503387 | 16.8800357829 | 43.9910018248 |
| 10 | GGPN | C6 | 30.2019879559 | 16.4998651654 | 44.8437871730 |

| | | | | | |
|---|---|---|---|---|---|
| 10 | GGPN | O6 | 29.6274670218 | 16.3218739226 | 43.7626574764 |
| 10 | GGPN | C5 | 29.6509675454 | 16.4044213528 | 46.1487987477 |
| 10 | GGPN | N7 | 28.3595098012 | 16.0852987675 | 46.5581343098 |
| 10 | GGPN | C8 | 28.4079662023 | 16.1867214460 | 47.8593280941 |
| 10 | GGPN | H8 | 27.5693821348 | 16.0260996648 | 48.5342798618 |
| 11 | GCPN | C | 32.6836141572 | 24.9525454078 | 49.2236698641 |
| 11 | GCPN | O1' | 32.3981800916 | 26.1182957176 | 49.5559583521 |
| 11 | GCPN | C2' | 32.0878654640 | 23.7852034845 | 49.8670817977 |
| 11 | GCPN | H2' | 31.0166718438 | 23.9026909281 | 49.8536007581 |
| 11 | GCPN | H2'' | 32.5231158115 | 23.8161617840 | 50.8522905021 |
| 11 | GCPN | N2' | 32.4970708197 | 22.4926082221 | 49.2727686843 |
| 11 | GCPN | C5' | 33.4412553447 | 21.7187276329 | 50.0575824926 |
| 11 | GCPN | H5' | 34.2066979949 | 22.4207604907 | 50.3870044964 |
| 11 | GCPN | H5'' | 33.8492516017 | 20.9222583767 | 49.4618176605 |
| 11 | GCPN | N | 31.5609753811 | 20.5031985425 | 51.0395142080 |
| 11 | GCPN | H1' | 31.5269100894 | 19.6483333545 | 50.4988645752 |
| 11 | GCPN | C6' | 32.8540885015 | 21.0665274733 | 51.3385452297 |
| 11 | GCPN | H6' | 32.7285480979 | 21.8519608542 | 52.0791420848 |
| 11 | GCPN | C6'' | 33.8374841771 | 20.0638195975 | 52.0100433435 |
| 11 | GCPN | H6'' | 34.3740932090 | 20.5227757827 | 52.8743419222 |
| 11 | GCPN | H6''' | 34.5821754859 | 19.6922067383 | 51.2690430270 |
| 11 | GCPN | O7' | 33.0195258609 | 18.8679455423 | 52.5027116792 |
| 11 | GCPN | C8' | 33.5986829540 | 18.1715909874 | 53.5967403773 |
| 11 | GCPN | H8' | 33.9772790509 | 18.8647474015 | 54.3806323245 |
| 11 | GCPN | H8'' | 34.4261591017 | 17.5144089582 | 53.2551640888 |
| 11 | GCPN | C9' | 32.4075572601 | 17.3737673643 | 54.1314076268 |
| 11 | GCPN | H9' | 31.8887812474 | 16.9202589498 | 53.2591029708 |
| 11 | GCPN | H9'' | 31.7170463181 | 18.1041918749 | 54.6093418352 |
| 11 | GCPN | O10' | 32.7341699577 | 16.3501836984 | 55.0563372270 |
| 11 | GCPN | C11' | 31.5412178220 | 15.8110462879 | 55.6235541587 |
| 11 | GCPN | H11' | 30.9889914984 | 15.1949070828 | 54.8797941408 |
| 11 | GCPN | H11'' | 30.8750181628 | 16.6519733184 | 55.9210738973 |
| 11 | GCPN | C12' | 31.8217741192 | 14.9655529853 | 56.8653553852 |
| 11 | GCPN | H12' | 32.6292876317 | 15.3992778894 | 57.4980785617 |
| 11 | GCPN | H12'' | 32.1251146839 | 13.9295330475 | 56.5966445191 |
| 11 | GCPN | O13' | 30.5929047455 | 14.9603064728 | 57.5809511840 |
| 11 | GCPN | H13' | 30.1302797676 | 14.1656791965 | 57.3129774050 |
| 11 | GCPN | C3' | 31.8694425539 | 22.0253437292 | 48.1294089955 |
| 11 | GCPN | O3' | 31.1256515078 | 22.7472363481 | 47.4716827922 |
| 11 | GCPN | C4' | 32.0781802301 | 20.5737282005 | 47.6185151537 |
| 11 | GCPN | H4' | 31.6986909553 | 19.8855435420 | 48.4001928913 |
| 11 | GCPN | H4'' | 33.1578534993 | 20.3536193453 | 47.5074930819 |
| 11 | GCPN | N1 | 31.3432712146 | 20.3298739712 | 46.3612946958 |
| 11 | GCPN | C6 | 29.9926523439 | 20.1181737008 | 46.3734167658 |
| 11 | GCPN | H6 | 29.5346128235 | 20.0954387209 | 47.3614618526 |
| 11 | GCPN | C5 | 29.2990182236 | 19.9383333881 | 45.2407698864 |
| 11 | GCPN | H5 | 28.2278867809 | 19.7534886386 | 45.2471641801 |
| 11 | GCPN | C2 | 32.0058326693 | 20.5051954552 | 45.1402396612 |
| 11 | GCPN | O2 | 33.1921270013 | 20.8711483111 | 45.1346135773 |
| 11 | GCPN | N3 | 31.3335812127 | 20.2780404041 | 43.9872994104 |
| 11 | GCPN | C4 | 30.0205220484 | 19.9821841978 | 44.0105232052 |
| 11 | GCPN | N4 | 29.3955561019 | 19.7042740879 | 42.8627540361 |
| 11 | GCPN | H41 | 29.9256441770 | 19.6688556091 | 42.0093712867 |
| 11 | GCPN | H42 | 28.4516769439 | 19.3941111604 | 42.8436091349 |
| 12 | GAPN | C | 33.0398363729 | 28.8947494165 | 45.4998348399 |
| 12 | GAPN | O1' | 33.1137980291 | 30.0686483773 | 45.8859180511 |
| 12 | GAPN | C2' | 32.8097764211 | 27.8081230123 | 46.4413291996 |
| 12 | GAPN | H2' | 31.7681148113 | 27.8595469644 | 46.7157249912 |
| 12 | GAPN | H2'' | 33.4809034770 | 28.0619268467 | 47.2385526355 |
| 12 | GAPN | N2' | 33.1617033671 | 26.4651800315 | 45.9233177976 |
| 12 | GAPN | C5' | 34.4020284982 | 25.7835991537 | 46.3559248599 |
| 12 | GAPN | H5' | 35.2695687423 | 26.3282974307 | 46.0220838662 |
| 12 | GAPN | H5'' | 34.4371956346 | 24.7733205445 | 45.9891656093 |
| 12 | GAPN | N | 33.5791761078 | 24.6827626459 | 48.3079711713 |
| 12 | GAPN | H1' | 33.6529534388 | 23.7246757749 | 48.0059403857 |
| 12 | GAPN | C6' | 34.5243529994 | 25.6334084271 | 47.8521689404 |
| 12 | GAPN | H6' | 34.2705975057 | 26.5998148971 | 48.2778929417 |
| 12 | GAPN | C6'' | 35.9493183240 | 25.3367714439 | 48.3556254956 |
| 12 | GAPN | H6'' | 36.7258323287 | 25.5136946355 | 47.5789406928 |
| 12 | GAPN | H6''' | 36.0119823711 | 24.2755324383 | 48.6887501719 |
| 12 | GAPN | O7' | 36.1859400979 | 26.2895671331 | 49.5231107766 |
| 12 | GAPN | C8' | 36.7725214191 | 25.6940898650 | 50.6644260030 |
| 12 | GAPN | H8' | 37.8523406886 | 25.4947538845 | 50.4868773743 |

| | | | | | |
|---|---|---|---|---|---|
| 12 | GAPN | H8'' | 36.2629547250 | 24.7502856802 | 50.9649733652 |
| 12 | GAPN | C9' | 36.5909556566 | 26.7488213984 | 51.7512842272 |
| 12 | GAPN | H9' | 35.5091707014 | 26.8445935217 | 52.0012511932 |
| 12 | GAPN | H9'' | 36.9361409660 | 27.7325651396 | 51.3566380083 |
| 12 | GAPN | O10' | 37.3388086317 | 26.3993289733 | 52.8999684254 |
| 12 | GAPN | C11' | 37.2448992971 | 27.4092303829 | 53.8933009599 |
| 12 | GAPN | H11' | 36.1854305674 | 27.5365320653 | 54.2095059643 |
| 12 | GAPN | H11'' | 37.6108183579 | 28.3805076805 | 53.4899701806 |
| 12 | GAPN | C12' | 38.0974852169 | 27.0042826316 | 55.0969506502 |
| 12 | GAPN | H12' | 39.1555007792 | 26.8801656334 | 54.7712482140 |
| 12 | GAPN | H12'' | 37.7300840004 | 26.0313037265 | 55.4953301446 |
| 12 | GAPN | O13' | 37.9981619956 | 28.0177368979 | 56.0884810505 |
| 12 | GAPN | H13' | 38.5367563005 | 27.7450655929 | 56.8372274569 |
| 12 | GAPN | C3' | 32.1639418401 | 25.8292909001 | 45.2394976218 |
| 12 | GAPN | O3' | 31.1556438698 | 26.4277121612 | 44.8521522136 |
| 12 | GAPN | C4' | 32.3038703423 | 24.3482358049 | 44.9155170389 |
| 12 | GAPN | H4' | 32.3300188956 | 23.7845103263 | 45.8576042318 |
| 12 | GAPN | H4'' | 33.2746427801 | 24.1924344303 | 44.4006251206 |
| 12 | GAPN | N9 | 31.2291676832 | 23.8942278153 | 44.0384372463 |
| 12 | GAPN | C5 | 30.1345814211 | 23.3659966176 | 42.2243741612 |
| 12 | GAPN | N7 | 29.2756237008 | 23.0958103524 | 43.2782690497 |
| 12 | GAPN | C8 | 29.9665867887 | 23.4306849869 | 44.3402936423 |
| 12 | GAPN | H8 | 29.6046292227 | 23.3492532071 | 45.3680265065 |
| 12 | GAPN | N1 | 31.0834806652 | 23.5564878581 | 40.0833645757 |
| 12 | GAPN | C2 | 32.1534818830 | 24.0993046143 | 40.6639016649 |
| 12 | GAPN | H2 | 32.9677520318 | 24.3889674787 | 39.9906492247 |
| 12 | GAPN | N3 | 32.3830524558 | 24.2907905808 | 41.9612615422 |
| 12 | GAPN | C4 | 31.3272504652 | 23.8836400922 | 42.6831943887 |
| 12 | GAPN | C6 | 30.0243995764 | 23.1720314889 | 40.8382513559 |
| 12 | GAPN | N6 | 28.9373222632 | 22.6152591337 | 40.2739031945 |
| 12 | GAPN | H61 | 28.1371099737 | 22.3827665930 | 40.8168326241 |
| 12 | GAPN | H62 | 28.9416398989 | 22.3529404542 | 39.2985801570 |
| 13 | GTPN | C | 33.3258663645 | 32.0684567967 | 40.5864555179 |
| 13 | GTPN | O1' | 33.1253587305 | 33.2872961784 | 40.6142378006 |
| 13 | GTPN | C2' | 33.0719200095 | 31.2728259135 | 41.7963203083 |
| 13 | GTPN | H2' | 32.1308041407 | 31.5990877487 | 42.2202311522 |
| 13 | GTPN | H2'' | 33.9005160679 | 31.5281347882 | 42.4266350926 |
| 13 | GTPN | N2' | 33.0817289296 | 29.8268850054 | 41.6468244454 |
| 13 | GTPN | C5' | 34.2867358908 | 29.1501781620 | 42.1419387959 |
| 13 | GTPN | H5' | 35.1131787503 | 29.7293671277 | 41.7714429967 |
| 13 | GTPN | H5'' | 34.3140784991 | 28.1208275084 | 41.8220480419 |
| 13 | GTPN | N | 33.2815006818 | 28.5531345007 | 44.2567074773 |
| 13 | GTPN | H1' | 32.9902850980 | 27.6542731058 | 43.8977863846 |
| 13 | GTPN | C6' | 34.3872637205 | 29.1960894125 | 43.6622208753 |
| 13 | GTPN | H6' | 34.3276840617 | 30.2513196473 | 43.9093884192 |
| 13 | GTPN | C6'' | 35.7050097528 | 28.7017927347 | 44.3226776366 |
| 13 | GTPN | H6'' | 36.2963861196 | 28.0309573100 | 43.6742746053 |
| 13 | GTPN | H6''' | 35.4613310189 | 28.0949200967 | 45.2102738421 |
| 13 | GTPN | O7' | 36.5006678763 | 29.8991752593 | 44.8476878147 |
| 13 | GTPN | C8' | 37.4327022249 | 29.5336788317 | 45.8583749558 |
| 13 | GTPN | H8' | 38.3035107587 | 28.9961336734 | 45.4261127087 |
| 13 | GTPN | H8'' | 36.9796389818 | 28.8889880131 | 46.6483492413 |
| 13 | GTPN | C9' | 37.9405900445 | 30.8213467900 | 46.4792920831 |
| 13 | GTPN | H9' | 37.1044756222 | 31.3355705114 | 47.0062112317 |
| 13 | GTPN | H9'' | 38.3260024453 | 31.4916161281 | 45.6762977121 |
| 13 | GTPN | O10' | 38.9741820705 | 30.4791412283 | 47.3822164840 |
| 13 | GTPN | C11' | 39.5140350560 | 31.6353779004 | 47.9938321633 |
| 13 | GTPN | H11' | 38.7046712015 | 32.2083297729 | 48.4999569171 |
| 13 | GTPN | H11'' | 39.9821568596 | 32.2864941238 | 47.2228141119 |
| 13 | GTPN | C12' | 40.5609105362 | 31.2193941305 | 49.0263293143 |
| 13 | GTPN | H12' | 41.4025815097 | 30.6953260644 | 48.5186606208 |
| 13 | GTPN | H12'' | 40.0977822298 | 30.5221498057 | 49.7622002434 |
| 13 | GTPN | O13' | 41.0184352736 | 32.3991445984 | 49.6734965139 |
| 13 | GTPN | H13' | 41.6382067347 | 32.1365831512 | 50.3596241424 |
| 13 | GTPN | C3' | 31.8816753211 | 29.2372173846 | 41.4081432859 |
| 13 | GTPN | O3' | 30.8662919955 | 29.8998368562 | 41.1787658692 |
| 13 | GTPN | C4' | 31.8475067689 | 27.7133105402 | 41.4286863857 |
| 13 | GTPN | H4' | 31.9504569616 | 27.3850494048 | 42.4799962208 |
| 13 | GTPN | H4'' | 32.7171195148 | 27.3434845154 | 40.8511404644 |
| 13 | GTPN | N1 | 30.6015013530 | 27.1328903964 | 40.9226043054 |
| 13 | GTPN | C6 | 29.5692432838 | 26.8242529900 | 41.7678921904 |
| 13 | GTPN | H6 | 29.7628466138 | 27.0157045972 | 42.8281389607 |
| 13 | GTPN | C2 | 30.5209349082 | 27.0119782502 | 39.5334127126 |

| | | | | | |
|---|---|---|---|---|---|
| 13 | GTPN | O2 | 31.3530786811 | 27.3435843840 | 38.7863549023 |
| 13 | GTPN | N3 | 29.3083700550 | 26.4431214951 | 39.1687452563 |
| 13 | GTPN | H3 | 29.2444057090 | 26.2913811242 | 38.1662463479 |
| 13 | GTPN | C4 | 28.2401102198 | 26.0330113783 | 39.9439015539 |
| 13 | GTPN | O4 | 27.2757779103 | 25.5079887723 | 39.4403259573 |
| 13 | GTPN | C5 | 28.4336386262 | 26.2807114191 | 41.3605615086 |
| 13 | GTPN | C5M | 27.3513063715 | 25.9078512416 | 42.3217644865 |
| 13 | GTPN | H51 | 27.6704128367 | 26.1277194096 | 43.3663092771 |
| 13 | GTPN | H52 | 27.1204236234 | 24.8211770404 | 42.2618159434 |
| 13 | GTPN | H53 | 26.4204043271 | 26.4803803508 | 42.1172184366 |
| 14 | GGPN | C | 31.4976719011 | 35.2802976720 | 37.0756516881 |
| 14 | GGPN | O1' | 31.3635725498 | 36.5086696337 | 37.3047819476 |
| 14 | GGPN | C2' | 32.1172660971 | 34.3345379540 | 38.0762590565 |
| 14 | GGPN | H2' | 31.5258834284 | 34.3247868593 | 38.9824542147 |
| 14 | GGPN | H2'' | 33.1223432517 | 34.6691511486 | 38.2711703120 |
| 14 | GGPN | N2' | 32.2657630580 | 32.9373364877 | 37.5857565911 |
| 14 | GGPN | C5' | 33.5962514276 | 32.4469193407 | 37.2716572753 |
| 14 | GGPN | H5' | 33.9984363089 | 33.1735398393 | 36.5613311630 |
| 14 | GGPN | H5'' | 33.5246358275 | 31.4602241688 | 36.8510435877 |
| 14 | GGPN | N | 33.8340882748 | 31.4848494331 | 39.4999152367 |
| 14 | GGPN | H1' | 33.8426218623 | 30.4883896377 | 39.3950714721 |
| 14 | GGPN | C6' | 34.5357957067 | 32.2676429212 | 38.5039449174 |
| 14 | GGPN | H6' | 34.7707232618 | 33.2473948606 | 38.9048525009 |
| 14 | GGPN | C6'' | 35.9237027504 | 31.6445788954 | 38.1918544881 |
| 14 | GGPN | H6'' | 36.7232668299 | 32.4119260454 | 38.3158124694 |
| 14 | GGPN | H6''' | 35.9622593421 | 31.2343723364 | 37.1572468394 |
| 14 | GGPN | O7' | 36.2030419976 | 30.5013647337 | 39.1865807222 |
| 14 | GGPN | C8' | 37.5400760722 | 30.5392196015 | 39.6722108889 |
| 14 | GGPN | H8' | 37.8599812066 | 31.5816228257 | 39.8957232466 |
| 14 | GGPN | H8'' | 38.2240925862 | 30.0865501221 | 38.9248236714 |
| 14 | GGPN | C9' | 37.5525729582 | 29.7315472740 | 40.9623945090 |
| 14 | GGPN | H9' | 36.9169054145 | 28.8356712163 | 40.7689080595 |
| 14 | GGPN | H9'' | 37.0696312880 | 30.3407244177 | 41.7605457534 |
| 14 | GGPN | O10' | 38.8117063348 | 29.2845671089 | 41.4347184188 |
| 14 | GGPN | C11' | 38.5262020935 | 28.4845663606 | 42.5655634792 |
| 14 | GGPN | H11' | 37.8301506483 | 27.6749079820 | 42.2465748510 |
| 14 | GGPN | H11'' | 38.0144566814 | 29.1367388191 | 43.2936456773 |
| 14 | GGPN | C12' | 39.6731059581 | 27.8137776658 | 43.3083773459 |
| 14 | GGPN | H12' | 40.3001411325 | 28.5803680948 | 43.8193624025 |
| 14 | GGPN | H12'' | 40.2879940119 | 27.2358922646 | 42.5853404729 |
| 14 | GGPN | O13' | 39.0499347676 | 26.9326292591 | 44.2451614113 |
| 14 | GGPN | H13' | 39.3602367058 | 27.1839747720 | 45.1243873247 |
| 14 | GGPN | C3' | 31.1986524025 | 32.0817617041 | 37.6862170378 |
| 14 | GGPN | O3' | 30.0717719067 | 32.5466096850 | 37.8889535314 |
| 14 | GGPN | C4' | 31.3463764050 | 30.5566813522 | 37.5295097235 |
| 14 | GGPN | H4' | 31.8855769736 | 30.1183647864 | 38.3894487033 |
| 14 | GGPN | H4'' | 31.8939832508 | 30.2851200962 | 36.6044647945 |
| 14 | GGPN | N9 | 30.0008788139 | 30.0071121460 | 37.4940094975 |
| 14 | GGPN | C4 | 29.3206077128 | 29.6387588346 | 36.3771325294 |
| 14 | GGPN | N2 | 29.1173939188 | 29.3096499520 | 32.9508113771 |
| 14 | GGPN | H21 | 29.9552624871 | 29.7488687045 | 32.6538465816 |
| 14 | GGPN | H22 | 28.4009864950 | 29.0755960946 | 32.2843785513 |
| 14 | GGPN | N3 | 29.7741740029 | 29.7113207542 | 35.1110946913 |
| 14 | GGPN | C2 | 28.8826173820 | 29.2504311756 | 34.2473441754 |
| 14 | GGPN | N1 | 27.6663337004 | 28.7269019916 | 34.6216181213 |
| 14 | GGPN | H1 | 27.0481011252 | 28.3750537317 | 33.9089643201 |
| 14 | GGPN | C6 | 27.1826982269 | 28.6431995239 | 35.9179308067 |
| 14 | GGPN | O6 | 26.0633770758 | 28.1699667281 | 36.1588618903 |
| 14 | GGPN | C5 | 28.0909460771 | 29.2005590726 | 36.8375261127 |
| 14 | GGPN | N7 | 27.9782192255 | 29.3822531236 | 38.2031610495 |
| 14 | GGPN | C8 | 29.1365120219 | 29.8734380073 | 38.5520705819 |
| 14 | GGPN | H8 | 29.4256644418 | 30.1881091951 | 39.5532870234 |
| 15 | GCPN | C | 27.6007680294 | 36.4364466019 | 32.0519771586 |
| 15 | GCPN | O1' | 27.3021647105 | 37.5057921421 | 31.5087401848 |
| 15 | GCPN | C2' | 28.4998574912 | 36.4663570161 | 33.2503551793 |
| 15 | GCPN | H2' | 28.0749607791 | 37.0654869073 | 34.0408003693 |
| 15 | GCPN | H2'' | 29.3719436674 | 36.9532850750 | 32.8409003772 |
| 15 | GCPN | N2' | 28.9477767949 | 35.1963443116 | 33.8087777739 |
| 15 | GCPN | C5' | 30.2727547444 | 34.8016272639 | 33.4316528365 |
| 15 | GCPN | H5' | 30.6209239836 | 35.4802571348 | 32.6544007009 |
| 15 | GCPN | H5'' | 30.1894144310 | 33.8291172877 | 32.9858586539 |
| 15 | GCPN | N | 31.1683003362 | 34.6227188552 | 35.9658570508 |
| 15 | GCPN | H1' | 31.2749634723 | 33.6693776492 | 36.2189136815 |

| | | | | | |
|---|---|---|---|---|---|
| 15 | GCPN | C6' | 31.3770100526 | 34.8158036869 | 34.5374299696 |
| 15 | GCPN | H6' | 31.5124805942 | 33.7387585958 | 34.4794863735 |
| 15 | GCPN | C6'' | 32.7679030950 | 35.1553648415 | 34.0143050207 |
| 15 | GCPN | H6'' | 32.7825678028 | 35.7598496269 | 33.0838036478 |
| 15 | GCPN | H6''' | 33.1964542973 | 34.1481778125 | 33.8284057095 |
| 15 | GCPN | O7' | 33.5890289218 | 35.8402562592 | 35.0613471525 |
| 15 | GCPN | C8' | 34.9564481434 | 35.4912684118 | 34.9789553964 |
| 15 | GCPN | H8' | 35.4908482234 | 36.0708303745 | 34.1963639529 |
| 15 | GCPN | H8'' | 35.1094452644 | 34.3978344822 | 34.8271422159 |
| 15 | GCPN | C9' | 35.3599144574 | 35.9053742423 | 36.3693583858 |
| 15 | GCPN | H9' | 34.7422332783 | 35.3120604706 | 37.0785944824 |
| 15 | GCPN | H9'' | 35.1324447927 | 36.9886643046 | 36.5023478844 |
| 15 | GCPN | O10' | 36.7061655824 | 35.6773672804 | 36.6586825114 |
| 15 | GCPN | C11' | 36.8718970264 | 35.7725820121 | 38.0568293264 |
| 15 | GCPN | H11' | 36.4262655066 | 34.8812073110 | 38.5500736635 |
| 15 | GCPN | H11'' | 36.3668122434 | 36.6831195393 | 38.4599751428 |
| 15 | GCPN | C12' | 38.3662831746 | 35.8313697120 | 38.2623114033 |
| 15 | GCPN | H12' | 38.7845027368 | 36.6566846269 | 37.6387003526 |
| 15 | GCPN | H12'' | 38.8096652362 | 34.8659155592 | 37.9258725858 |
| 15 | GCPN | O13' | 38.6172874381 | 36.0619873845 | 39.6359202685 |
| 15 | GCPN | H13' | 38.1919470632 | 36.8989526105 | 39.8501394749 |
| 15 | GCPN | C3' | 28.1075646743 | 34.4051805134 | 34.5519398528 |
| 15 | GCPN | O3' | 27.0404616166 | 34.8350392800 | 34.9634105727 |
| 15 | GCPN | C4' | 28.4130645931 | 32.9108025327 | 34.8569838552 |
| 15 | GCPN | H4' | 29.0195804888 | 32.8728368345 | 35.7816980165 |
| 15 | GCPN | H4'' | 29.0254001268 | 32.4595707857 | 34.0527155154 |
| 15 | GCPN | N1 | 27.1482674804 | 32.1719884560 | 35.0386095763 |
| 15 | GCPN | C6 | 26.5912467040 | 32.0147457043 | 36.2801543400 |
| 15 | GCPN | H6 | 27.1996612293 | 32.3786538583 | 37.1135492351 |
| 15 | GCPN | C5 | 25.4153039347 | 31.3884545636 | 36.4420460067 |
| 15 | GCPN | H5 | 24.9893138013 | 31.2097646970 | 37.4269706031 |
| 15 | GCPN | C2 | 26.4362553496 | 31.7660358341 | 33.9024617472 |
| 15 | GCPN | O2 | 26.9096640108 | 32.0031213069 | 32.7732469884 |
| 15 | GCPN | N3 | 25.2571755214 | 31.1205286375 | 34.0469686928 |
| 15 | GCPN | C4 | 24.7423633743 | 30.9142800267 | 35.2734990251 |
| 15 | GCPN | N4 | 23.5909488502 | 30.2421443800 | 35.3928855036 |
| 15 | GCPN | H41 | 23.1962552934 | 29.8281552783 | 34.5648508536 |
| 15 | GCPN | H42 | 23.3463610715 | 29.8204655965 | 36.2599875730 |
| 16 | GCPN | C | 22.4335404528 | 37.0814303844 | 29.4101163165 |
| 16 | GCPN | C2' | 23.8531189711 | 37.1719021322 | 30.0023900116 |
| 16 | GCPN | H2' | 23.9192865901 | 38.0978688394 | 30.5574257276 |
| 16 | GCPN | H2'' | 24.4938609467 | 37.1880646207 | 29.1369449308 |
| 16 | GCPN | N2' | 24.3381456583 | 36.0535029040 | 30.8321916918 |
| 16 | GCPN | C5' | 25.1104623147 | 35.0234195555 | 30.1339463799 |
| 16 | GCPN | H5' | 24.7798833897 | 35.0558074941 | 29.0893033777 |
| 16 | GCPN | H5'' | 24.8912620844 | 34.0644326750 | 30.5842396149 |
| 16 | GCPN | N | 27.1829240786 | 35.2756000746 | 31.5218341955 |
| 16 | GCPN | H1' | 27.2548344277 | 34.4069485439 | 32.0190204640 |
| 16 | GCPN | C6' | 26.6765347263 | 35.2079744693 | 30.1614999426 |
| 16 | GCPN | H6' | 26.9228802859 | 36.1315003324 | 29.6430012306 |
| 16 | GCPN | C6'' | 27.4185166323 | 34.1309227969 | 29.3269704410 |
| 16 | GCPN | H6'' | 27.7512955815 | 34.5636267422 | 28.3548027401 |
| 16 | GCPN | H6''' | 26.7071900856 | 33.2978865182 | 29.1399307164 |
| 16 | GCPN | O7' | 28.6472266877 | 33.5961888007 | 30.1087246663 |
| 16 | GCPN | C8' | 29.8153028994 | 33.4105867718 | 29.3143067336 |
| 16 | GCPN | H8' | 29.8852143671 | 34.1699557313 | 28.5037602912 |
| 16 | GCPN | H8'' | 29.8009954542 | 32.3937089570 | 28.8671389030 |
| 16 | GCPN | C9' | 30.9773518152 | 33.5536375439 | 30.3103094604 |
| 16 | GCPN | H9' | 30.6802501875 | 32.9203582394 | 31.1713528047 |
| 16 | GCPN | H9'' | 31.0166090084 | 34.6141514444 | 30.6531259516 |
| 16 | GCPN | O10' | 32.2944239208 | 33.1591943415 | 30.0213016609 |
| 16 | GCPN | C11' | 33.0177683458 | 33.3795097253 | 31.2234480061 |
| 16 | GCPN | H11' | 32.5450276661 | 32.8215054683 | 32.0709075519 |
| 16 | GCPN | H11'' | 32.9721961511 | 34.4633235530 | 31.4720532868 |
| 16 | GCPN | C12' | 34.4759064133 | 32.9896816657 | 31.1273884970 |
| 16 | GCPN | H12' | 34.9648721884 | 33.6474383258 | 30.3741494688 |
| 16 | GCPN | H12'' | 34.5503974815 | 31.9257839046 | 30.8115780158 |
| 16 | GCPN | O13' | 35.0415019085 | 33.1802876231 | 32.4174766773 |
| 16 | GCPN | H13' | 35.9968022166 | 33.1721673505 | 32.2958945101 |
| 16 | GCPN | C3' | 24.0830496846 | 36.0364050050 | 32.1858074966 |
| 16 | GCPN | O3' | 23.5618397452 | 36.9865098101 | 32.8029095578 |
| 16 | GCPN | C4' | 24.4556114960 | 34.8356352690 | 33.1080461999 |
| 16 | GCPN | H4' | 25.0057470724 | 35.3164542859 | 33.9447518900 |

| | | | | | |
|---|---|---|---|---|---|
| 16 | GCPN | H4'' | 25.1549490087 | 34.1517938028 | 32.6000840315 |
| 16 | GCPN | N1 | 23.3413257060 | 34.0496116323 | 33.6561343009 |
| 16 | GCPN | C6 | 23.1270662273 | 33.9753529500 | 35.0220152153 |
| 16 | GCPN | H6 | 23.8163960642 | 34.5705136442 | 35.6215400602 |
| 16 | GCPN | C5 | 22.1456564921 | 33.2204183311 | 35.5252397129 |
| 16 | GCPN | H5 | 21.9674087281 | 33.1303908979 | 36.6030919080 |
| 16 | GCPN | C2 | 22.6124609761 | 33.2141974175 | 32.8309930118 |
| 16 | GCPN | O2 | 22.9533072401 | 33.0966104073 | 31.6106106937 |
| 16 | GCPN | N3 | 21.5883050508 | 32.5048572732 | 33.2963918014 |
| 16 | GCPN | C4 | 21.3268914959 | 32.4876051922 | 34.6249723725 |
| 16 | GCPN | N4 | 20.3059670202 | 31.7405270646 | 35.0286878695 |
| 16 | GCPN | H41 | 19.8027513956 | 31.2441463215 | 34.3112741294 |
| 16 | GCPN | H42 | 20.0832908174 | 31.5572311189 | 35.9847158651 |
| 16 | GCPN | OT1 | 21.7005005805 | 36.0918884751 | 29.6769941396 |
| 16 | GCPN | OT2 | 22.0827831753 | 38.0161260684 | 28.6358924449 |

Model 8

| | | | | | |
|---|---|---|---|---|---|
| 1 | GGPN | N | 14.5594662909 | 29.5767484405 | 25.5979613235 |
| 1 | GGPN | HT1 | 15.2510269029 | 29.9573133346 | 26.2997764338 |
| 1 | GGPN | HT2 | 13.7135031221 | 29.2950345120 | 26.1488024080 |
| 1 | GGPN | HT3 | 14.3251382977 | 30.2745392777 | 24.9471504800 |
| 1 | GGPN | C6' | 15.1917251715 | 28.2712486628 | 25.0698567233 |
| 1 | GGPN | H6' | 14.5687638834 | 27.4604707678 | 25.3652751164 |
| 1 | GGPN | C | 19.4510190128 | 26.8861650983 | 25.1204102639 |
| 1 | GGPN | O1' | 20.1089913937 | 26.0884747516 | 24.4137415091 |
| 1 | GGPN | C2' | 18.2647421882 | 26.3404727759 | 25.8862457810 |
| 1 | GGPN | H2' | 18.6183601845 | 25.6481053646 | 26.6215683521 |
| 1 | GGPN | H2'' | 17.6853166795 | 25.8021751833 | 25.1455913739 |
| 1 | GGPN | N2' | 17.3786800652 | 27.3676566591 | 26.5492688445 |
| 1 | GGPN | C5' | 16.6417998666 | 28.2123218534 | 25.6740871731 |
| 1 | GGPN | H5' | 17.2981382368 | 28.8987639497 | 25.1900938205 |
| 1 | GGPN | H5'' | 16.5270806827 | 27.2724224111 | 25.1765208181 |
| 1 | GGPN | C6'' | 15.5534096005 | 28.1874410994 | 23.5438404086 |
| 1 | GGPN | H6'' | 15.6873891654 | 29.2285214643 | 23.2315711914 |
| 1 | GGPN | H6''' | 16.5078189599 | 27.6821544795 | 23.3708629707 |
| 1 | GGPN | O7' | 14.6171917405 | 27.4861835269 | 22.5543095801 |
| 1 | GGPN | C8' | 15.3302110838 | 27.4609952777 | 21.2641052473 |
| 1 | GGPN | H8' | 16.2153097000 | 26.8044970262 | 21.3394306619 |
| 1 | GGPN | H8'' | 15.7004260506 | 28.4485568513 | 21.0614619758 |
| 1 | GGPN | C9' | 14.4484681115 | 27.2166577334 | 20.0285714204 |
| 1 | GGPN | H9' | 13.4097783315 | 27.2415905043 | 20.3980641584 |
| 1 | GGPN | H9'' | 14.6773973276 | 26.2022447231 | 19.6193609530 |
| 1 | GGPN | O10' | 14.6270491818 | 28.2299443827 | 19.0027211056 |
| 1 | GGPN | C11' | 13.6955574130 | 28.0557791480 | 17.9024035966 |
| 1 | GGPN | H11' | 12.6703186796 | 28.2323049575 | 18.2853583953 |
| 1 | GGPN | H11'' | 13.7731148164 | 26.9985639843 | 17.5791691106 |
| 1 | GGPN | C12' | 13.8408899523 | 28.9186156985 | 16.6151385499 |
| 1 | GGPN | H12' | 14.8850733641 | 28.9028319679 | 16.2340129267 |
| 1 | GGPN | H12'' | 13.5562488709 | 29.9792438934 | 16.8006590725 |
| 1 | GGPN | O13' | 12.9751960971 | 28.3787971784 | 15.6080869123 |
| 1 | GGPN | H13' | 13.1298176338 | 28.8429462176 | 14.7728905538 |
| 1 | GGPN | C3' | 17.8888614786 | 27.8928216490 | 27.6726922696 |
| 1 | GGPN | O3' | 18.9005957180 | 27.4302402032 | 28.1884632644 |
| 1 | GGPN | C4' | 17.2205049697 | 29.1533150486 | 28.1982284466 |
| 1 | GGPN | H4' | 16.1210539224 | 29.1513135665 | 28.0376566687 |
| 1 | GGPN | H4'' | 17.6784922028 | 30.0012109673 | 27.6294355511 |
| 1 | GGPN | N9 | 17.4580559393 | 29.2657175163 | 29.5736299278 |
| 1 | GGPN | C4 | 18.4244671083 | 30.0247491697 | 30.1909163300 |
| 1 | GGPN | N2 | 21.0523020740 | 32.3076539605 | 29.9476933305 |
| 1 | GGPN | H21 | 21.0912645617 | 32.5251921682 | 28.9480198442 |
| 1 | GGPN | H22 | 21.6379386456 | 32.7973470136 | 30.5490683065 |
| 1 | GGPN | N3 | 19.3179418810 | 30.8189650467 | 29.5198756501 |
| 1 | GGPN | C2 | 20.1184684834 | 31.4451061468 | 30.3345412754 |
| 1 | GGPN | N1 | 20.0802295388 | 31.2890604031 | 31.7608003681 |
| 1 | GGPN | H1 | 20.7017085529 | 31.7864118368 | 32.2931127519 |
| 1 | GGPN | C6 | 19.1716527540 | 30.4717315155 | 32.3521460721 |
| 1 | GGPN | O6 | 19.1671703604 | 30.3689331369 | 33.6762377435 |
| 1 | GGPN | C5 | 18.2947241069 | 29.8329603400 | 31.5466377387 |
| 1 | GGPN | N7 | 17.2321851227 | 28.9534679498 | 31.7864513404 |
| 1 | GGPN | C8 | 16.7957113961 | 28.6417403739 | 30.6481588333 |
| 1 | GGPN | H8 | 15.9690620801 | 27.9344778712 | 30.4525156940 |
| 2 | GGPN | C | 24.8825729278 | 26.6895918906 | 24.8538847283 |
| 2 | GGPN | O1' | 25.5737415069 | 26.0444514177 | 24.0880389659 |
| 2 | GGPN | C2' | 23.3759703571 | 26.5766561209 | 24.9411416582 |
| 2 | GGPN | H2' | 23.1460513293 | 25.6426741392 | 25.4358094057 |
| 2 | GGPN | H2'' | 23.0375916595 | 26.5831333883 | 23.9247545052 |
| 2 | GGPN | N2' | 22.6938728030 | 27.7096055207 | 25.6382136112 |
| 2 | GGPN | C5' | 22.1256009726 | 28.8899991325 | 24.9032383431 |
| 2 | GGPN | H5' | 22.8629563597 | 29.1320550357 | 24.1559691076 |
| 2 | GGPN | H5'' | 22.0154218129 | 29.7259480208 | 25.5416978707 |
| 2 | GGPN | N | 19.7873967215 | 28.1798090084 | 25.1553102101 |
| 2 | GGPN | H1' | 19.4710363037 | 28.7705753712 | 25.8883471181 |
| 2 | GGPN | C6' | 20.7212254174 | 28.7502137749 | 24.2077317542 |
| 2 | GGPN | H6' | 20.8721370687 | 28.0612258416 | 23.4009154158 |
| 2 | GGPN | C6'' | 20.2423275361 | 30.1357637770 | 23.5056176052 |
| 2 | GGPN | H6'' | 20.7354476387 | 30.1824535206 | 22.5229173549 |
| 2 | GGPN | H6''' | 20.6742590165 | 30.9394752134 | 24.1402060878 |
| 2 | GGPN | O7' | 18.6297841751 | 30.3665268840 | 23.3682196035 |

| | | | | | |
|---|---|---|---|---|---|
| 2 | GGPN | C8' | 18.1988153296 | 31.1228161724 | 22.1759426657 |
| 2 | GGPN | H8' | 18.7082537086 | 30.6195478767 | 21.3126063607 |
| 2 | GGPN | H8'' | 18.5406370947 | 32.1705683607 | 22.1716275114 |
| 2 | GGPN | C9' | 16.6490679129 | 31.0729600971 | 21.6498792716 |
| 2 | GGPN | H9' | 16.7135155497 | 31.2275936959 | 20.5613256741 |
| 2 | GGPN | H9'' | 16.5329652247 | 29.9976028727 | 21.7438865515 |
| 2 | GGPN | O10' | 15.4650758700 | 31.8188733634 | 22.0665134541 |
| 2 | GGPN | C11' | 14.1840193627 | 31.0260853195 | 22.1452858816 |
| 2 | GGPN | H11' | 13.9342318896 | 31.1036447934 | 23.2171858357 |
| 2 | GGPN | H11'' | 14.4753653426 | 30.0098973839 | 21.8495887147 |
| 2 | GGPN | C12' | 12.7564019460 | 31.0378929363 | 21.4693363721 |
| 2 | GGPN | H12' | 12.7718018590 | 30.6918655986 | 20.4162451634 |
| 2 | GGPN | H12'' | 12.1436017967 | 31.9646830168 | 21.4723547857 |
| 2 | GGPN | O13' | 12.0367506717 | 30.0542310438 | 22.2480230774 |
| 2 | GGPN | H13' | 11.2691001726 | 29.7296068054 | 21.7348194491 |
| 2 | GGPN | C3' | 22.6906982293 | 27.6896048211 | 26.9939685065 |
| 2 | GGPN | O3' | 23.2767293597 | 26.8123528376 | 27.6420036010 |
| 2 | GGPN | C4' | 21.9912568719 | 28.8305501621 | 27.7102973728 |
| 2 | GGPN | H4' | 20.9488215917 | 28.9394292521 | 27.3690962864 |
| 2 | GGPN | H4'' | 22.5413840299 | 29.7725423270 | 27.5034778605 |
| 2 | GGPN | N9 | 21.9868363873 | 28.6044642514 | 29.1453621596 |
| 2 | GGPN | C4 | 22.7755560431 | 29.2642466631 | 30.0187798479 |
| 2 | GGPN | N2 | 25.3258381464 | 31.4811834046 | 30.7391999893 |
| 2 | GGPN | H21 | 25.6402786757 | 31.7541765142 | 29.8372983096 |
| 2 | GGPN | H22 | 25.8120137284 | 31.7751557744 | 31.5664987565 |
| 2 | GGPN | N3 | 23.7060314743 | 30.1852301707 | 29.7314528232 |
| 2 | GGPN | C2 | 24.2950342366 | 30.6613921825 | 30.8181017731 |
| 2 | GGPN | N1 | 23.9339487320 | 30.2640431008 | 32.0872392258 |
| 2 | GGPN | H1 | 24.3734732997 | 30.7245569982 | 32.8668168684 |
| 2 | GGPN | C6 | 22.9583770413 | 29.3408172280 | 32.4036914901 |
| 2 | GGPN | O6 | 22.6923035286 | 29.0383347652 | 33.5810318371 |
| 2 | GGPN | C5 | 22.3928694199 | 28.7634101679 | 31.2592770162 |
| 2 | GGPN | N7 | 21.4648447253 | 27.7544517321 | 31.1242216472 |
| 2 | GGPN | C8 | 21.2587307299 | 27.6811364842 | 29.8340903070 |
| 2 | GGPN | H8 | 20.6389305293 | 26.9516757387 | 29.3267682963 |
| 3 | GCPN | C | 29.9185352326 | 25.2861471770 | 26.6274956909 |
| 3 | GCPN | O1' | 30.5955107702 | 24.3688724886 | 26.1875873527 |
| 3 | GCPN | C2' | 28.4922374573 | 25.4518122186 | 26.2488396617 |
| 3 | GCPN | H2' | 27.9734148504 | 24.5560287437 | 26.5624746179 |
| 3 | GCPN | H2'' | 28.5248866621 | 25.5303146827 | 25.1740196846 |
| 3 | GCPN | N2' | 27.8261335555 | 26.6619069577 | 26.7775452738 |
| 3 | GCPN | C5' | 27.8509671121 | 27.8377021252 | 25.8893654791 |
| 3 | GCPN | H5' | 28.3485542725 | 27.5442810712 | 24.9780324050 |
| 3 | GCPN | H5'' | 28.4055708326 | 28.5995981478 | 26.4170409403 |
| 3 | GCPN | N | 25.3781160220 | 27.6050152488 | 25.6897486712 |
| 3 | GCPN | H1' | 24.7896482985 | 27.8116140855 | 26.4556669717 |
| 3 | GCPN | C6' | 26.4997285509 | 28.4620170664 | 25.4506049132 |
| 3 | GCPN | H6' | 26.3249343966 | 29.2477428095 | 26.1747964098 |
| 3 | GCPN | C6'' | 26.4845175702 | 29.2070487375 | 24.0499138142 |
| 3 | GCPN | H6'' | 27.1959982748 | 28.7447527800 | 23.3318972731 |
| 3 | GCPN | H6''' | 26.7774382820 | 30.2713709049 | 24.1843710634 |
| 3 | GCPN | O7' | 25.0418293539 | 29.1550677308 | 23.4641731760 |
| 3 | GCPN | C8' | 24.9080510066 | 29.3390601346 | 22.0531037742 |
| 3 | GCPN | H8' | 25.8671102403 | 29.2284120725 | 21.5034303479 |
| 3 | GCPN | H8'' | 24.5153402227 | 30.3653636735 | 21.8743305009 |
| 3 | GCPN | C9' | 23.8734853748 | 28.3403754326 | 21.4833725752 |
| 3 | GCPN | H9' | 23.3090189027 | 27.9026141913 | 22.3297680941 |
| 3 | GCPN | H9'' | 24.3862143686 | 27.5136508663 | 20.9488844074 |
| 3 | GCPN | O10' | 22.9522475911 | 28.9874276893 | 20.6172887070 |
| 3 | GCPN | C11' | 21.7941220865 | 28.1982799825 | 20.3745652551 |
| 3 | GCPN | H11' | 21.3139924986 | 27.9264044089 | 21.3391303403 |
| 3 | GCPN | H11'' | 22.0475602120 | 27.2582338539 | 19.8378422020 |
| 3 | GCPN | C12' | 20.8184577753 | 29.0532337445 | 19.5639502247 |
| 3 | GCPN | H12' | 21.2076680513 | 29.2148944506 | 18.5328467885 |
| 3 | GCPN | H12'' | 20.7292192902 | 30.0483103362 | 20.0547773899 |
| 3 | GCPN | O13' | 19.5465608107 | 28.4202756033 | 19.5376645505 |
| 3 | GCPN | H13' | 19.6603259388 | 27.6002429637 | 19.0471907659 |
| 3 | GCPN | C3' | 27.3322697756 | 26.6324789119 | 28.0603280802 |
| 3 | GCPN | O3' | 27.5750697239 | 25.6792135416 | 28.7976758146 |
| 3 | GCPN | C4' | 26.4693323849 | 27.7684217411 | 28.6805343572 |
| 3 | GCPN | H4' | 25.5361155987 | 27.8515667727 | 28.1027348045 |
| 3 | GCPN | H4'' | 27.0097395697 | 28.7298973290 | 28.5779651409 |
| 3 | GCPN | N1 | 26.0348909146 | 27.4801085915 | 30.0584765290 |

| | | | | | |
|---|---|---|---|---|---|
| 3 | GCPN | C6 | 25.0277173418 | 26.5833317888 | 30.3042380325 |
| 3 | GCPN | H6 | 24.5500940935 | 26.1566333689 | 29.4171576821 |
| 3 | GCPN | C5 | 24.6521202431 | 26.2771726634 | 31.5511021412 |
| 3 | GCPN | H5 | 23.8513913073 | 25.5695635460 | 31.7558948779 |
| 3 | GCPN | C2 | 26.7508266498 | 28.0331164899 | 31.1259748617 |
| 3 | GCPN | O2 | 27.7394360623 | 28.7460090227 | 30.8907699730 |
| 3 | GCPN | N3 | 26.3500648690 | 27.7748481595 | 32.3915813570 |
| 3 | GCPN | C4 | 25.3308956683 | 26.9244250542 | 32.6279872802 |
| 3 | GCPN | N4 | 24.9429797094 | 26.6928683414 | 33.8843636947 |
| 3 | GCPN | H41 | 25.3465193552 | 27.2319460346 | 34.6310897167 |
| 3 | GCPN | H42 | 24.0909153091 | 26.2161756107 | 34.0709598185 |
| 4 | GAPN | C | 34.0868228166 | 23.8026937490 | 30.2239756768 |
| 4 | GAPN | O1' | 35.0398531085 | 23.1342372862 | 29.8479302706 |
| 4 | GAPN | C2' | 32.9219080424 | 24.0689584712 | 29.3538010144 |
| 4 | GAPN | H2' | 32.3371331132 | 23.1667846580 | 29.2924460707 |
| 4 | GAPN | H2'' | 33.3910964357 | 24.3264235459 | 28.4183143820 |
| 4 | GAPN | N2' | 32.0718139293 | 25.1998100868 | 29.7811164541 |
| 4 | GAPN | C5' | 32.2118674086 | 26.4747029632 | 29.0779721774 |
| 4 | GAPN | H5' | 33.2579786750 | 26.7234055821 | 29.1092084182 |
| 4 | GAPN | H5'' | 31.6005350115 | 27.2440725052 | 29.5252471458 |
| 4 | GAPN | N | 30.4201844870 | 26.2154236721 | 27.4471385199 |
| 4 | GAPN | H1' | 29.7864991965 | 26.8322242510 | 27.8948427240 |
| 4 | GAPN | C6' | 31.8341448125 | 26.4010375183 | 27.6057886544 |
| 4 | GAPN | H6' | 32.3470507502 | 25.5323616322 | 27.2207297501 |
| 4 | GAPN | C6'' | 32.3995157063 | 27.5879524643 | 26.7971521250 |
| 4 | GAPN | H6'' | 33.0752545513 | 28.1861114470 | 27.4381180964 |
| 4 | GAPN | H6''' | 31.5758603333 | 28.2362465840 | 26.4325051141 |
| 4 | GAPN | O7' | 33.2059672813 | 27.0365807474 | 25.6232942825 |
| 4 | GAPN | C8' | 32.8356291422 | 27.5934495909 | 24.3738879588 |
| 4 | GAPN | H8' | 33.2757899557 | 28.6084307708 | 24.2525186325 |
| 4 | GAPN | H8'' | 31.7306796773 | 27.6525019291 | 24.2535162009 |
| 4 | GAPN | C9' | 33.4125516011 | 26.6390349804 | 23.3350541031 |
| 4 | GAPN | H9' | 32.8340763298 | 25.6874515642 | 23.3575997047 |
| 4 | GAPN | H9'' | 34.4679039656 | 26.4131659724 | 23.6118268771 |
| 4 | GAPN | O10' | 33.3752389015 | 27.2089631427 | 22.0413062868 |
| 4 | GAPN | C11' | 34.0017014490 | 26.3475237081 | 21.1026793775 |
| 4 | GAPN | H11' | 33.4604005543 | 25.3760813077 | 21.0575690486 |
| 4 | GAPN | H11'' | 35.0543145902 | 26.1483285635 | 21.4074054499 |
| 4 | GAPN | C12' | 33.9872547481 | 27.0145757543 | 19.7275941212 |
| 4 | GAPN | H12' | 34.5320347826 | 27.9843025177 | 19.7884511446 |
| 4 | GAPN | H12'' | 32.9323415813 | 27.2132590297 | 19.4311473425 |
| 4 | GAPN | O13' | 34.6141192775 | 26.1501645946 | 18.7895923220 |
| 4 | GAPN | H13' | 34.5928732804 | 26.5887459041 | 17.9340220045 |
| 4 | GAPN | C3' | 31.1070180473 | 24.9475613987 | 30.7145416769 |
| 4 | GAPN | O3' | 31.1023144947 | 23.8926805614 | 31.3495895472 |
| 4 | GAPN | C4' | 30.0186107882 | 25.9843499138 | 31.0271565300 |
| 4 | GAPN | H4' | 29.4298458415 | 26.1686292505 | 30.1153918871 |
| 4 | GAPN | H4'' | 30.4990137781 | 26.9411623202 | 31.3179556524 |
| 4 | GAPN | N9 | 29.1748272124 | 25.5308557003 | 32.1339423561 |
| 4 | GAPN | C5 | 28.4131698434 | 25.1921917795 | 34.1609784423 |
| 4 | GAPN | N7 | 27.5243981643 | 24.5578538586 | 33.3070904182 |
| 4 | GAPN | C8 | 28.0159653947 | 24.7799718778 | 32.1147170836 |
| 4 | GAPN | H8 | 27.5597939842 | 24.4302711960 | 31.1855234329 |
| 4 | GAPN | N1 | 29.4672293244 | 26.0585044229 | 36.0745143605 |
| 4 | GAPN | C2 | 30.4234847693 | 26.5197004036 | 35.2680996280 |
| 4 | GAPN | H2 | 31.2361595547 | 27.0633196765 | 35.7632100724 |
| 4 | GAPN | N3 | 30.5002641250 | 26.4462661815 | 33.9387542062 |
| 4 | GAPN | C4 | 29.4443707242 | 25.7718899153 | 33.4502507975 |
| 4 | GAPN | C6 | 28.4223169729 | 25.3680236008 | 35.5537325747 |
| 4 | GAPN | N6 | 27.4423559663 | 24.8954954476 | 36.3476630611 |
| 4 | GAPN | H61 | 26.6930766617 | 24.3580046999 | 35.9734031281 |
| 4 | GAPN | H62 | 27.3982015150 | 25.1466462682 | 37.3237087288 |
| 5 | GTPN | C | 37.3969146199 | 22.4003139571 | 34.2287448353 |
| 5 | GTPN | O1' | 38.0942470431 | 21.4105454470 | 34.0588872416 |
| 5 | GTPN | C2' | 36.3524922271 | 22.7822488529 | 33.2703921806 |
| 5 | GTPN | H2' | 35.8805168201 | 21.8808596226 | 32.9071583167 |
| 5 | GTPN | H2'' | 36.9202311031 | 23.2648480141 | 32.4980851382 |
| 5 | GTPN | N2' | 35.3532649262 | 23.7274556484 | 33.7811044883 |
| 5 | GTPN | C5' | 35.4368090537 | 25.0998815624 | 33.2937301535 |
| 5 | GTPN | H5' | 36.4418165096 | 25.4220100850 | 33.4891650178 |
| 5 | GTPN | H5'' | 34.7084256640 | 25.7495322091 | 33.7503160794 |
| 5 | GTPN | N | 34.0694587253 | 24.4029534836 | 31.4128787364 |
| 5 | GTPN | H1' | 33.2038451185 | 24.6292559170 | 31.8406336700 |

| | | | | | |
|---|---|---|---|---|---|
| 5 | GTPN | C6' | 35.2354165441 | 25.1322672890 | 31.7867608870 |
| 5 | GTPN | H6' | 36.1070334071 | 24.6357834609 | 31.3850427351 |
| 5 | GTPN | C6'' | 35.2561962269 | 26.5076547688 | 31.0996679820 |
| 5 | GTPN | H6'' | 35.8948585346 | 27.2153446651 | 31.6483700424 |
| 5 | GTPN | H6''' | 34.2197342823 | 26.9015612449 | 31.0506057006 |
| 5 | GTPN | O7' | 35.7962875362 | 26.2308770056 | 29.6834253878 |
| 5 | GTPN | C8' | 35.9557371196 | 27.3540207878 | 28.8328710844 |
| 5 | GTPN | H8' | 35.9382351775 | 28.3140872128 | 29.3777909963 |
| 5 | GTPN | H8'' | 35.1526329117 | 27.3575666049 | 28.0609848857 |
| 5 | GTPN | C9' | 37.2605889983 | 27.0937827924 | 28.0807077758 |
| 5 | GTPN | H9' | 37.1267804585 | 26.1290432136 | 27.5370722383 |
| 5 | GTPN | H9'' | 38.1313352688 | 26.9765643082 | 28.7563706670 |
| 5 | GTPN | O10' | 37.4807003892 | 28.1581435642 | 27.1726460866 |
| 5 | GTPN | C11' | 38.5628408035 | 27.8804867346 | 26.2932326959 |
| 5 | GTPN | H11' | 38.4421506906 | 26.8737895952 | 25.8313854335 |
| 5 | GTPN | H11'' | 39.5258722173 | 27.8988100325 | 26.8497358498 |
| 5 | GTPN | C12' | 38.5825645629 | 28.9393386528 | 25.1856726959 |
| 5 | GTPN | H12' | 38.7651333027 | 29.9410194088 | 25.6355533312 |
| 5 | GTPN | H12'' | 37.5917556787 | 28.9524696626 | 24.6767328255 |
| 5 | GTPN | O13' | 39.6083222610 | 28.6159216340 | 24.2559171907 |
| 5 | GTPN | H13' | 39.5630276211 | 29.2560875479 | 23.5388120844 |
| 5 | GTPN | C3' | 34.2277215673 | 23.1870257608 | 34.3286950530 |
| 5 | GTPN | O3' | 34.1435257281 | 21.9855437253 | 34.5612636393 |
| 5 | GTPN | C4' | 33.0597465226 | 24.1327824896 | 34.6361147709 |
| 5 | GTPN | H4' | 32.6717275945 | 24.5226627917 | 33.6738939783 |
| 5 | GTPN | H4'' | 33.4652787415 | 24.9962433363 | 35.2070625021 |
| 5 | GTPN | N1 | 31.9479058622 | 23.5112949378 | 35.3565624510 |
| 5 | GTPN | C6 | 30.9743425597 | 22.8045650146 | 34.6802198924 |
| 5 | GTPN | H6 | 31.1240819201 | 22.7310851123 | 33.5991953486 |
| 5 | GTPN | C2 | 31.9549883456 | 23.5592745140 | 36.7252345526 |
| 5 | GTPN | O2 | 32.8937917520 | 24.0939458003 | 37.3718382818 |
| 5 | GTPN | N3 | 30.8846428843 | 22.9974877641 | 37.3188994221 |
| 5 | GTPN | H3 | 30.8573351610 | 23.0925456674 | 38.3304583383 |
| 5 | GTPN | C4 | 29.8135875053 | 22.3455667867 | 36.7225034365 |
| 5 | GTPN | O4 | 28.8842460769 | 21.9417586892 | 37.3886171392 |
| 5 | GTPN | C5 | 29.9273125136 | 22.2407921792 | 35.2816921614 |
| 5 | GTPN | C5M | 28.8672143932 | 21.5129344654 | 34.5239980781 |
| 5 | GTPN | H51 | 29.0739123626 | 21.5369315978 | 33.4301581551 |
| 5 | GTPN | H52 | 27.8699342192 | 21.9788341230 | 34.6893278690 |
| 5 | GTPN | H53 | 28.8106555098 | 20.4480506393 | 34.8411939698 |
| 6 | GGPN | C | 39.3315450157 | 19.3476191393 | 38.3413598455 |
| 6 | GGPN | O1' | 40.2545589602 | 18.6749465055 | 38.5302071977 |
| 6 | GGPN | C2' | 38.9058846069 | 19.9698644393 | 37.0276340362 |
| 6 | GGPN | H2' | 38.4740404038 | 19.1810884875 | 36.4180181188 |
| 6 | GGPN | H2'' | 39.8313673775 | 20.3387031796 | 36.5988918249 |
| 6 | GGPN | N2' | 37.9840627828 | 21.1108409509 | 37.1921661418 |
| 6 | GGPN | C5' | 38.4944115984 | 22.5039233472 | 37.3179546647 |
| 6 | GGPN | H5' | 39.3795725149 | 22.4574544480 | 37.9145835163 |
| 6 | GGPN | H5'' | 37.7653971280 | 23.1326478472 | 37.7774520043 |
| 6 | GGPN | N | 37.5814570680 | 23.2295640838 | 35.2615485297 |
| 6 | GGPN | H1' | 36.9071244825 | 23.9085015538 | 35.5085763540 |
| 6 | GGPN | C6' | 38.7995140622 | 23.2281701200 | 36.0013578998 |
| 6 | GGPN | H6' | 39.5579011115 | 22.6671176412 | 35.4785864632 |
| 6 | GGPN | C6'' | 39.3463798080 | 24.6937014129 | 36.1749935362 |
| 6 | GGPN | H6'' | 40.4258526141 | 24.7428418463 | 35.9224898841 |
| 6 | GGPN | H6''' | 39.2297299165 | 25.0079144086 | 37.2340823754 |
| 6 | GGPN | O7' | 38.5459933090 | 25.6805789533 | 35.2632028705 |
| 6 | GGPN | C8' | 39.3743178542 | 26.4766923542 | 34.4059025649 |
| 6 | GGPN | H8' | 40.2861620523 | 25.9237614708 | 34.0923039408 |
| 6 | GGPN | H8'' | 39.6852251642 | 27.4042599336 | 34.9380761473 |
| 6 | GGPN | C9' | 38.5862881834 | 26.8772953499 | 33.1412319138 |
| 6 | GGPN | H9' | 37.5988869556 | 27.2469300402 | 33.4901319575 |
| 6 | GGPN | H9'' | 38.4296142759 | 25.9781379252 | 32.5050901838 |
| 6 | GGPN | O10' | 39.1817698020 | 27.9007280987 | 32.3606468006 |
| 6 | GGPN | C11' | 38.2254651072 | 28.4424513180 | 31.4594531149 |
| 6 | GGPN | H11' | 37.3202285496 | 28.7708662697 | 32.0221081957 |
| 6 | GGPN | H11'' | 37.9111484446 | 27.6657235964 | 30.7334258775 |
| 6 | GGPN | C12' | 38.7497369861 | 29.6746627078 | 30.7306951707 |
| 6 | GGPN | H12' | 39.6662209683 | 29.4410483708 | 30.1439216989 |
| 6 | GGPN | H12'' | 39.0067298934 | 30.4475199678 | 31.4933691849 |
| 6 | GGPN | O13' | 37.7073609047 | 30.1389935864 | 29.8870123361 |
| 6 | GGPN | H13' | 37.6403809942 | 29.5168696447 | 29.1452707391 |
| 6 | GGPN | C3' | 36.6630347711 | 20.8506358185 | 37.3792531392 |

| | | | | | |
|---|---|---|---|---|---|
| 6 | GGPN | O3' | 36.2689948653 | 19.6661260353 | 37.4175220965 |
| 6 | GGPN | C4' | 35.6508891021 | 21.9608979021 | 37.6742381590 |
| 6 | GGPN | H4' | 35.5132928053 | 22.6173451872 | 36.7954259797 |
| 6 | GGPN | H4'' | 35.9949635118 | 22.5674754484 | 38.5353504790 |
| 6 | GGPN | N9 | 34.4024005793 | 21.3284983010 | 38.0475932565 |
| 6 | GGPN | C4 | 33.8924179678 | 21.1956769455 | 39.3104231486 |
| 6 | GGPN | N2 | 34.1639163840 | 21.5378670815 | 42.7547191175 |
| 6 | GGPN | H21 | 35.0358091045 | 21.9945093669 | 42.8721623228 |
| 6 | GGPN | H22 | 33.6489488358 | 21.2224851452 | 43.5595099878 |
| 6 | GGPN | N3 | 34.4641822871 | 21.6296449395 | 40.4597740508 |
| 6 | GGPN | C2 | 33.7332900387 | 21.2964108654 | 41.5255091605 |
| 6 | GGPN | N1 | 32.5289271050 | 20.6476579002 | 41.4351062688 |
| 6 | GGPN | H1 | 32.0086878352 | 20.4769377225 | 42.2817032303 |
| 6 | GGPN | C6 | 31.9150977967 | 20.2197805533 | 40.2665066573 |
| 6 | GGPN | O6 | 30.8292170763 | 19.6363930567 | 40.2892695709 |
| 6 | GGPN | C5 | 32.7168204971 | 20.4943277691 | 39.1389530599 |
| 6 | GGPN | N7 | 32.5348142218 | 20.1347866187 | 37.8129608704 |
| 6 | GGPN | C8 | 33.5686782779 | 20.6450332484 | 37.2044051655 |
| 6 | GGPN | H8 | 33.8336394119 | 20.5273154965 | 36.1530346482 |
| 7 | GCPN | C | 39.2469189623 | 15.8689919897 | 42.8650853760 |
| 7 | GCPN | O1' | 40.1034035082 | 15.0310220802 | 43.0971211041 |
| 7 | GCPN | C2' | 39.2695300976 | 16.6050183988 | 41.5461706804 |
| 7 | GCPN | H2' | 39.1399933596 | 15.8708091708 | 40.7520153768 |
| 7 | GCPN | H2'' | 40.2679116569 | 16.9913015794 | 41.5078477392 |
| 7 | GCPN | N2' | 38.3774164943 | 17.7323217726 | 41.3236851991 |
| 7 | GCPN | C5' | 38.9795344575 | 19.0831861479 | 41.6260463509 |
| 7 | GCPN | H5' | 40.0386616534 | 18.9381760868 | 41.6600505369 |
| 7 | GCPN | H5'' | 38.5646629894 | 19.3678284455 | 42.5730926519 |
| 7 | GCPN | N | 38.4340086004 | 19.7837592785 | 39.3133062601 |
| 7 | GCPN | H1' | 37.5570020495 | 19.9189180113 | 38.9809771608 |
| 7 | GCPN | C6' | 38.8012158583 | 20.2324165545 | 40.6267235353 |
| 7 | GCPN | H6' | 37.8898696981 | 20.7578455388 | 40.9453664881 |
| 7 | GCPN | C6'' | 39.9150946662 | 21.3724760231 | 40.6855601116 |
| 7 | GCPN | H6'' | 40.5745880911 | 21.2968165269 | 41.5744442804 |
| 7 | GCPN | H6''' | 39.3915986517 | 22.3434705056 | 40.7214642583 |
| 7 | GCPN | O7' | 40.7490685456 | 21.3052179492 | 39.3858419737 |
| 7 | GCPN | C8' | 41.8544346047 | 22.1667773760 | 39.1912863829 |
| 7 | GCPN | H8' | 42.6827205641 | 21.8987027104 | 39.8660824076 |
| 7 | GCPN | H8'' | 41.5771860863 | 23.2315416576 | 39.3215078056 |
| 7 | GCPN | C9' | 42.2348207057 | 21.9103722125 | 37.7141012175 |
| 7 | GCPN | H9' | 41.2914467216 | 21.7073400590 | 37.1710301843 |
| 7 | GCPN | H9'' | 42.8668119768 | 20.9998751985 | 37.6191665119 |
| 7 | GCPN | O10' | 42.8476136019 | 23.0285117715 | 37.0948157914 |
| 7 | GCPN | C11' | 42.7957177751 | 22.9499447141 | 35.6703475009 |
| 7 | GCPN | H11' | 41.7429647827 | 22.9664352876 | 35.3110666480 |
| 7 | GCPN | H11'' | 43.2704178500 | 22.0141172713 | 35.3000477070 |
| 7 | GCPN | C12' | 43.5235291454 | 24.1806443118 | 35.1270136587 |
| 7 | GCPN | H12' | 44.5808450256 | 24.1725865596 | 35.4783157882 |
| 7 | GCPN | H12'' | 43.0344913801 | 25.0954842536 | 35.5329990998 |
| 7 | GCPN | O13' | 43.4609169432 | 24.2013539245 | 33.7085756006 |
| 7 | GCPN | H13' | 43.8835164379 | 23.3986436982 | 33.3865604649 |
| 7 | GCPN | C3' | 37.1119856717 | 17.4863332764 | 40.9622506409 |
| 7 | GCPN | O3' | 36.7251634760 | 16.3316875924 | 40.6896914063 |
| 7 | GCPN | C4' | 36.0848217824 | 18.6221985581 | 40.9275865879 |
| 7 | GCPN | H4' | 36.2283206307 | 19.2444862173 | 40.0289298665 |
| 7 | GCPN | H4'' | 36.2421933655 | 19.2569749318 | 41.8240682205 |
| 7 | GCPN | N1 | 34.7396905198 | 18.0666889221 | 40.8351875826 |
| 7 | GCPN | C6 | 34.2480518249 | 17.6683735562 | 39.6236465411 |
| 7 | GCPN | H6 | 34.8824885992 | 17.8889765582 | 38.7605667897 |
| 7 | GCPN | C5 | 33.0424932798 | 17.0910583304 | 39.5299501172 |
| 7 | GCPN | H5 | 32.6220063835 | 16.7954513904 | 38.5746268962 |
| 7 | GCPN | C2 | 34.0667655118 | 17.7647482621 | 42.0152223932 |
| 7 | GCPN | O2 | 34.6069605055 | 17.9973880417 | 43.1130347758 |
| 7 | GCPN | N3 | 32.8329654676 | 17.2294041870 | 41.9317577438 |
| 7 | GCPN | C4 | 32.3121609906 | 16.8978097919 | 40.7382406392 |
| 7 | GCPN | N4 | 31.0846385916 | 16.3864261317 | 40.6870455110 |
| 7 | GCPN | H41 | 30.5473858783 | 16.4571627208 | 41.5382414268 |
| 7 | GCPN | H42 | 30.5688659719 | 16.4731704804 | 39.8442559044 |
| 8 | GCPN | C | 37.0711943071 | 11.8230716389 | 46.8969698354 |
| 8 | GCPN | C2' | 37.7803888078 | 12.6486228566 | 45.8082559042 |
| 8 | GCPN | H2' | 38.1029295457 | 11.9488716639 | 45.0481276719 |
| 8 | GCPN | H2'' | 38.6182525326 | 13.0767990472 | 46.3273026780 |
| 8 | GCPN | N2' | 37.0687992801 | 13.7776843797 | 45.1773397903 |

| | | | | | |
|---|---|---|---|---|---|
| 8 | GCPN | C5' | 37.2907639405 | 15.1025876163 | 45.7816472385 |
| 8 | GCPN | H5' | 37.4427567037 | 14.9255583367 | 46.8331700297 |
| 8 | GCPN | H5'' | 36.4231701114 | 15.7147850841 | 45.6595785506 |
| 8 | GCPN | N | 38.3132649957 | 16.1831240609 | 43.7970115898 |
| 8 | GCPN | H1' | 37.5245276821 | 16.7288961600 | 43.5749996040 |
| 8 | GCPN | C6' | 38.5130196565 | 15.8878454178 | 45.2001599405 |
| 8 | GCPN | H6' | 39.3575355258 | 15.2237722212 | 45.3273254680 |
| 8 | GCPN | C6'' | 38.8637372728 | 17.2018932752 | 45.9890156747 |
| 8 | GCPN | H6'' | 39.6094434709 | 17.0207165672 | 46.7933501455 |
| 8 | GCPN | H6''' | 37.9141907462 | 17.5054031799 | 46.4781884946 |
| 8 | GCPN | O7' | 39.3238700982 | 18.3767523176 | 45.0383222976 |
| 8 | GCPN | C8' | 40.6754754245 | 18.8123938473 | 45.2362491058 |
| 8 | GCPN | H8' | 41.1451507952 | 18.2869910717 | 46.0943527342 |
| 8 | GCPN | H8'' | 40.6767094902 | 19.8968226091 | 45.5049766952 |
| 8 | GCPN | C9' | 41.6512962753 | 18.6413228561 | 44.0377847047 |
| 8 | GCPN | H9' | 41.2305802121 | 18.0108612391 | 43.2338437742 |
| 8 | GCPN | H9'' | 42.5512053682 | 18.1481171927 | 44.4461161135 |
| 8 | GCPN | O10' | 41.9812355525 | 19.9329486139 | 43.5322963062 |
| 8 | GCPN | C11' | 42.9222706937 | 19.9429541987 | 42.4531881341 |
| 8 | GCPN | H11' | 42.4211033241 | 19.6926575462 | 41.4923075828 |
| 8 | GCPN | H11'' | 43.7733811412 | 19.2518681576 | 42.6159296845 |
| 8 | GCPN | C12' | 43.4352130114 | 21.3899637010 | 42.4657555371 |
| 8 | GCPN | H12' | 43.8437492971 | 21.5755221597 | 43.4846921171 |
| 8 | GCPN | H12'' | 42.5841119132 | 22.0886408880 | 42.3001469059 |
| 8 | GCPN | O13' | 44.4501166271 | 21.6090069104 | 41.4941946789 |
| 8 | GCPN | H13' | 44.3908558682 | 22.5256699088 | 41.2162156296 |
| 8 | GCPN | C3' | 36.3221072215 | 13.5720819336 | 44.0500151230 |
| 8 | GCPN | O3' | 36.2674560727 | 12.4930501766 | 43.4410394282 |
| 8 | GCPN | C4' | 35.4790123230 | 14.7218708615 | 43.4400836132 |
| 8 | GCPN | H4' | 36.0980834577 | 15.1791621678 | 42.6503649884 |
| 8 | GCPN | H4'' | 35.2595899076 | 15.5083890445 | 44.1710503797 |
| 8 | GCPN | N1 | 34.2071683999 | 14.2780582024 | 42.8660093207 |
| 8 | GCPN | C6 | 34.0911988534 | 14.0006856119 | 41.5359750026 |
| 8 | GCPN | H6 | 34.9967645501 | 14.1795810482 | 40.9531526902 |
| 8 | GCPN | C5 | 32.9275388328 | 13.5777178121 | 41.0152437933 |
| 8 | GCPN | H5 | 32.8006623933 | 13.3705230444 | 39.9576840368 |
| 8 | GCPN | C2 | 33.1185540079 | 14.1131449365 | 43.7216264811 |
| 8 | GCPN | O2 | 33.2342590454 | 14.3682272902 | 44.9306363894 |
| 8 | GCPN | N3 | 31.9451687772 | 13.7058927201 | 43.2064388185 |
| 8 | GCPN | C4 | 31.8242079128 | 13.4226625513 | 41.9053758351 |
| 8 | GCPN | N4 | 30.6366092758 | 13.0093244078 | 41.4590330232 |
| 8 | GCPN | H41 | 29.8690734887 | 13.0873439079 | 42.1130523542 |
| 8 | GCPN | H42 | 30.4194445582 | 12.9116102861 | 40.4977402569 |
| 8 | GCPN | OT1 | 35.9008906578 | 12.1146745342 | 47.2514246545 |
| 8 | GCPN | OT2 | 37.7443709093 | 10.8864564466 | 47.4129304621 |
| 9 | GGPN | N | 24.0600469509 | 11.7769385353 | 50.4724379206 |
| 9 | GGPN | HT1 | 24.1126122844 | 12.6461790624 | 49.9018690295 |
| 9 | GGPN | HT2 | 23.0590988474 | 11.5190645360 | 50.6243916548 |
| 9 | GGPN | HT3 | 24.5316655140 | 10.9884453169 | 49.9892221775 |
| 9 | GGPN | C6' | 24.6642177208 | 12.0323658372 | 51.8177663068 |
| 9 | GGPN | H6' | 24.0950914288 | 12.8606953811 | 52.2155399415 |
| 9 | GGPN | C | 26.8659829542 | 15.8674046201 | 52.0038985828 |
| 9 | GGPN | O1' | 26.6662311026 | 16.9643226949 | 52.4989153881 |
| 9 | GGPN | C2' | 25.7327850270 | 14.9887270719 | 51.5928218424 |
| 9 | GGPN | H2' | 25.0807654623 | 15.5559304368 | 50.9432823584 |
| 9 | GGPN | H2'' | 25.2495142057 | 14.7331235813 | 52.5218212929 |
| 9 | GGPN | N2' | 26.1647867932 | 13.7482214838 | 50.9432959655 |
| 9 | GGPN | C5' | 26.1082194697 | 12.5374190096 | 51.7405096830 |
| 9 | GGPN | H5' | 26.7814398370 | 11.7743525853 | 51.3723132818 |
| 9 | GGPN | H5'' | 26.3820460844 | 12.8413068367 | 52.7363980484 |
| 9 | GGPN | C6'' | 24.3774401143 | 10.8645679977 | 52.7480038894 |
| 9 | GGPN | H6'' | 24.7623973090 | 11.1016457855 | 53.7583444134 |
| 9 | GGPN | H6''' | 24.7799134655 | 9.8858365976 | 52.4078250913 |
| 9 | GGPN | O7' | 22.8625634465 | 10.8399898989 | 52.7388137732 |
| 9 | GGPN | C8' | 22.2793715621 | 10.1801543159 | 53.8341293189 |
| 9 | GGPN | H8' | 22.8145029659 | 10.3616775541 | 54.7936370028 |
| 9 | GGPN | H8'' | 22.2204429139 | 9.0873455616 | 53.6348232647 |
| 9 | GGPN | C9' | 20.8968644135 | 10.8037096489 | 53.8643329956 |
| 9 | GGPN | H9' | 20.5176661148 | 10.9335862291 | 52.8239562775 |
| 9 | GGPN | H9'' | 20.9270869751 | 11.8034206138 | 54.3519251356 |
| 9 | GGPN | O10' | 20.0225311396 | 9.9521681564 | 54.5500688478 |
| 9 | GGPN | C11' | 18.7072922738 | 10.4564327507 | 54.4609475108 |
| 9 | GGPN | H11' | 18.3961154822 | 10.5553888698 | 53.3953554183 |

| | | | | | |
|---|---|---|---|---|---|
| 9 | GGPN | H11'' | 18.6470178128 | 11.4564786945 | 54.9457861287 |
| 9 | GGPN | C12' | 17.8158505987 | 9.4569398147 | 55.1717278047 |
| 9 | GGPN | H12' | 18.1523324612 | 9.3610152612 | 56.2300176068 |
| 9 | GGPN | H12'' | 17.9077263842 | 8.4656183568 | 54.6694544248 |
| 9 | GGPN | O13' | 16.4859332897 | 9.9431481138 | 55.0989585365 |
| 9 | GGPN | H13' | 15.9282426555 | 9.3119715782 | 55.5610278205 |
| 9 | GGPN | C3' | 26.3877925865 | 13.7727613245 | 49.6052512935 |
| 9 | GGPN | O3' | 26.1667773871 | 14.7826588597 | 48.9534786480 |
| 9 | GGPN | C4' | 26.8936683702 | 12.4930188425 | 48.9652035273 |
| 9 | GGPN | H4' | 26.2418505222 | 11.6338333278 | 49.1733064053 |
| 9 | GGPN | H4'' | 27.8951462795 | 12.2771979969 | 49.4031568097 |
| 9 | GGPN | N9 | 26.9824115201 | 12.6547790230 | 47.5229475648 |
| 9 | GGPN | C4 | 28.1365824974 | 12.9251074150 | 46.8672104651 |
| 9 | GGPN | N2 | 31.5568773523 | 13.4176577921 | 46.9204587817 |
| 9 | GGPN | H21 | 31.7998499458 | 13.3027872985 | 47.8754036133 |
| 9 | GGPN | H22 | 32.2877902399 | 13.6139972465 | 46.2566981467 |
| 9 | GGPN | N3 | 29.3402190059 | 13.0793577218 | 47.4479637814 |
| 9 | GGPN | C2 | 30.2965697157 | 13.2786449730 | 46.5465614494 |
| 9 | GGPN | N1 | 30.0460826024 | 13.3366657511 | 45.1994668420 |
| 9 | GGPN | H1 | 30.8242916795 | 13.4813254679 | 44.5733601089 |
| 9 | GGPN | C6 | 28.8066777801 | 13.1914688326 | 44.5804510981 |
| 9 | GGPN | O6 | 28.6650277048 | 13.2400157403 | 43.3557608420 |
| 9 | GGPN | C5 | 27.7927127794 | 12.9673801240 | 45.5337922052 |
| 9 | GGPN | N7 | 26.4332275459 | 12.7506448109 | 45.3616370370 |
| 9 | GGPN | C8 | 25.9870120042 | 12.5864646397 | 46.5684098725 |
| 9 | GGPN | H8 | 24.9375825691 | 12.4254948307 | 46.8082124121 |
| 10 | GGPN | C | 30.2258108202 | 20.2095517673 | 51.5184840564 |
| 10 | GGPN | O1' | 30.2897400710 | 21.1984472850 | 52.2333926897 |
| 10 | GGPN | C2' | 29.1454776963 | 19.1609851321 | 51.6172688759 |
| 10 | GGPN | H2' | 28.2444609738 | 19.5724703298 | 51.1792590830 |
| 10 | GGPN | H2'' | 29.0217634657 | 18.9632764964 | 52.6633620902 |
| 10 | GGPN | N2' | 29.4864488046 | 17.8786339230 | 50.9418694167 |
| 10 | GGPN | C5' | 30.1068861093 | 16.7444081003 | 51.6815881713 |
| 10 | GGPN | H5' | 30.8790856063 | 17.1761951470 | 52.2890481910 |
| 10 | GGPN | H5'' | 30.5341040819 | 16.0209071478 | 51.0359340954 |
| 10 | GGPN | N | 28.1015670171 | 15.3751370169 | 51.8500285504 |
| 10 | GGPN | H1' | 28.2696388664 | 14.5668208337 | 51.3011360147 |
| 10 | GGPN | C6' | 29.2101681705 | 15.9142856304 | 52.5937643044 |
| 10 | GGPN | H6' | 28.8532785896 | 16.5540669548 | 53.3768576376 |
| 10 | GGPN | C6'' | 30.0571660787 | 14.7866028217 | 53.2676631778 |
| 10 | GGPN | H6'' | 30.3863941159 | 15.1339606336 | 54.2657186936 |
| 10 | GGPN | H6''' | 30.9718198391 | 14.5785505262 | 52.6687901827 |
| 10 | GGPN | O7' | 29.2277050923 | 13.4695574150 | 53.3606435211 |
| 10 | GGPN | C8' | 29.0311767835 | 13.0180893490 | 54.7030889685 |
| 10 | GGPN | H8' | 28.7080449988 | 13.8699405190 | 55.3378121701 |
| 10 | GGPN | H8'' | 29.9770554889 | 12.5992622129 | 55.1041127008 |
| 10 | GGPN | C9' | 27.9093616326 | 11.9518329711 | 54.7115407170 |
| 10 | GGPN | H9' | 28.0071282507 | 11.3907430335 | 53.7538384242 |
| 10 | GGPN | H9'' | 26.9643739112 | 12.5366243643 | 54.6837477572 |
| 10 | GGPN | O10' | 27.7748171756 | 10.9855334187 | 55.7572431076 |
| 10 | GGPN | C11' | 26.5294025453 | 10.3213856631 | 55.5369097715 |
| 10 | GGPN | H11' | 26.5346231323 | 9.9972379226 | 54.4695780572 |
| 10 | GGPN | H11'' | 25.7113601690 | 11.0653597206 | 55.6817576624 |
| 10 | GGPN | C12' | 26.0731270244 | 9.0791698148 | 56.3260797799 |
| 10 | GGPN | H12' | 25.7524099932 | 9.3489174843 | 57.3582699550 |
| 10 | GGPN | H12'' | 26.8810773592 | 8.3160912504 | 56.3828853743 |
| 10 | GGPN | O13' | 24.9592711745 | 8.5954478928 | 55.5628892893 |
| 10 | GGPN | H13' | 24.5898524269 | 7.8212415369 | 55.9995537401 |
| 10 | GGPN | C3' | 29.2715986106 | 17.7652636850 | 49.6224136347 |
| 10 | GGPN | O3' | 28.9037579582 | 18.7449937684 | 48.9395569994 |
| 10 | GGPN | C4' | 29.5388890381 | 16.4250935932 | 48.9550045617 |
| 10 | GGPN | H4' | 28.9394336404 | 15.6222862648 | 49.4167697327 |
| 10 | GGPN | H4'' | 30.6181470743 | 16.1738312751 | 49.0498905843 |
| 10 | GGPN | N9 | 29.2262749536 | 16.5031542624 | 47.5547202552 |
| 10 | GGPN | C4 | 30.1341223189 | 16.7228373068 | 46.5720660896 |
| 10 | GGPN | N2 | 33.3852454392 | 17.3411402085 | 45.5524584603 |
| 10 | GGPN | H21 | 33.9055702989 | 17.2594435673 | 46.3932967270 |
| 10 | GGPN | H22 | 33.8512830479 | 17.4807733884 | 44.6751792868 |
| 10 | GGPN | N3 | 31.4582689155 | 16.9184453252 | 46.7487796482 |
| 10 | GGPN | C2 | 32.0919632498 | 17.0578229158 | 45.5912422295 |
| 10 | GGPN | N1 | 31.4519093007 | 16.9906476494 | 44.3809493031 |
| 10 | GGPN | H1 | 32.0111048014 | 17.0224729287 | 43.5435518572 |
| 10 | GGPN | C6 | 30.0932049912 | 16.7762217120 | 44.1788745117 |

| | | | | | |
|---|---|---|---|---|---|
| 10 | GGPN | O6 | 29.6068074504 | 16.7320707396 | 43.0479184095 |
| 10 | GGPN | C5 | 29.4055947213 | 16.6896107698 | 45.4077106773 |
| 10 | GGPN | N7 | 28.0515988212 | 16.5248933587 | 45.6698982905 |
| 10 | GGPN | C8 | 27.9930850561 | 16.4273094068 | 46.9697501183 |
| 10 | GGPN | H8 | 27.0921245398 | 16.3195830532 | 47.5761934306 |
| 11 | GCPN | C | 32.1079135258 | 24.8867959142 | 49.3176434950 |
| 11 | GCPN | O1' | 31.9050373138 | 26.0151763542 | 49.7420077054 |
| 11 | GCPN | C2' | 31.3861233984 | 23.7078254739 | 49.8525023353 |
| 11 | GCPN | H2' | 30.3321583402 | 23.8791850403 | 49.6820652459 |
| 11 | GCPN | H2'' | 31.6268206279 | 23.7262260088 | 50.9027531662 |
| 11 | GCPN | N2' | 31.8063316773 | 22.3980522526 | 49.3057312584 |
| 11 | GCPN | C5' | 32.8368033581 | 21.6869607901 | 50.0832129689 |
| 11 | GCPN | H5' | 33.0467736732 | 22.2807103725 | 50.9590909581 |
| 11 | GCPN | H5'' | 33.7005409657 | 21.6147062749 | 49.4382155061 |
| 11 | GCPN | N | 31.1310390662 | 19.9264458509 | 50.5827036615 |
| 11 | GCPN | H1' | 30.8254817641 | 19.2672158044 | 49.9103644339 |
| 11 | GCPN | C6' | 32.5224624285 | 20.2575194413 | 50.6057252063 |
| 11 | GCPN | H6' | 32.9141744862 | 19.6024267380 | 49.8376388193 |
| 11 | GCPN | C6'' | 33.2705115525 | 19.8141374821 | 51.9282208628 |
| 11 | GCPN | H6'' | 33.4371452933 | 20.6780031328 | 52.6071431722 |
| 11 | GCPN | H6''' | 34.2616507276 | 19.3813940304 | 51.6677357783 |
| 11 | GCPN | O7' | 32.4190678693 | 18.7319372884 | 52.6624973237 |
| 11 | GCPN | C8' | 32.6020855085 | 18.6281995510 | 54.0772827891 |
| 11 | GCPN | H8' | 33.1621021752 | 19.4918253668 | 54.4936962906 |
| 11 | GCPN | H8'' | 33.1878972274 | 17.7046882673 | 54.2842585452 |
| 11 | GCPN | C9' | 31.2592216199 | 18.5052327377 | 54.8444543526 |
| 11 | GCPN | H9' | 30.4165358026 | 18.4845190060 | 54.1286534874 |
| 11 | GCPN | H9'' | 31.1303048730 | 19.3863842771 | 55.5055859618 |
| 11 | GCPN | O10' | 31.2211111907 | 17.3178900056 | 55.6231620196 |
| 11 | GCPN | C11' | 29.9534988459 | 17.1038500348 | 56.2384747666 |
| 11 | GCPN | H11' | 29.1742662307 | 16.9582801236 | 55.4612895288 |
| 11 | GCPN | H11'' | 29.6496200129 | 17.9640939796 | 56.8743893022 |
| 11 | GCPN | C12' | 30.0994979006 | 15.8399506055 | 57.0941849358 |
| 11 | GCPN | H12' | 30.7004783622 | 16.0874662777 | 57.9982951845 |
| 11 | GCPN | H12'' | 30.6557927992 | 15.0760885488 | 56.5069845790 |
| 11 | GCPN | O13' | 28.8283856264 | 15.3172820326 | 57.4566616463 |
| 11 | GCPN | H13' | 28.9865436828 | 14.5241242884 | 57.9793883516 |
| 11 | GCPN | C3' | 31.2793291459 | 21.9848158146 | 48.1049521971 |
| 11 | GCPN | O3' | 30.5799609514 | 22.7458244520 | 47.4398947760 |
| 11 | GCPN | C4' | 31.5259865960 | 20.5763702605 | 47.4928877325 |
| 11 | GCPN | H4' | 31.0704656430 | 19.8255820215 | 48.1569706696 |
| 11 | GCPN | H4'' | 32.6152924633 | 20.3768183639 | 47.4630619397 |
| 11 | GCPN | N1 | 30.8589309076 | 20.3798788655 | 46.1933126837 |
| 11 | GCPN | C6 | 29.5070159943 | 20.1607861321 | 46.1277219327 |
| 11 | GCPN | H6 | 28.9939822219 | 20.0892774203 | 47.0905142915 |
| 11 | GCPN | C5 | 28.8785681274 | 20.0243105884 | 44.9524847413 |
| 11 | GCPN | H5 | 27.8063733660 | 19.8513817917 | 44.8882436468 |
| 11 | GCPN | C2 | 31.5954498416 | 20.5517968870 | 45.0191286705 |
| 11 | GCPN | O2 | 32.7926504765 | 20.8801183976 | 45.0908502941 |
| 11 | GCPN | N3 | 30.9875938124 | 20.3622134709 | 43.8263868998 |
| 11 | GCPN | C4 | 29.6658232208 | 20.1013760266 | 43.7651203073 |
| 11 | GCPN | N4 | 29.0918980703 | 19.9024695951 | 42.5754203790 |
| 11 | GCPN | H41 | 29.6787570657 | 19.7965155972 | 41.7663624428 |
| 11 | GCPN | H42 | 28.1702587065 | 19.5338758295 | 42.5181022166 |
| 12 | GAPN | C | 32.9837131340 | 28.8748772248 | 45.4351489201 |
| 12 | GAPN | O1' | 33.1174959867 | 30.0429905452 | 45.7707436060 |
| 12 | GAPN | C2' | 32.5937391764 | 27.8322966439 | 46.3985262219 |
| 12 | GAPN | H2' | 31.5440136346 | 27.9533977321 | 46.6066253933 |
| 12 | GAPN | H2'' | 33.2162128680 | 28.0640125421 | 47.2464544307 |
| 12 | GAPN | N2' | 32.8742969314 | 26.4512883257 | 45.9583340488 |
| 12 | GAPN | C5' | 34.0472489052 | 25.7609959794 | 46.5018207406 |
| 12 | GAPN | H5' | 34.9007151831 | 26.3839714812 | 46.3162390757 |
| 12 | GAPN | H5'' | 34.1640743015 | 24.7752351733 | 46.0763226146 |
| 12 | GAPN | N | 33.0108658280 | 24.6315432602 | 48.3698175480 |
| 12 | GAPN | H1' | 33.0074792133 | 23.7321600160 | 47.9531972700 |
| 12 | GAPN | C6' | 34.0116488420 | 25.5868819648 | 48.0035349283 |
| 12 | GAPN | H6' | 33.7448003708 | 26.5536470441 | 48.4025441560 |
| 12 | GAPN | C6'' | 35.4116348004 | 25.2946163112 | 48.5800140338 |
| 12 | GAPN | H6'' | 36.1774948390 | 25.4295338935 | 47.7913569194 |
| 12 | GAPN | H6''' | 35.4634934279 | 24.2553910778 | 48.9653268286 |
| 12 | GAPN | O7' | 35.6917320355 | 26.2982176523 | 49.6939598353 |
| 12 | GAPN | C8' | 36.1104292473 | 25.6904311774 | 50.9020251711 |
| 12 | GAPN | H8' | 37.1811713223 | 25.3941751869 | 50.8384232511 |

| | | | | | |
|---|---|---|---|---|---|
| 12 | GAPN | H8'' | 35.4937459454 | 24.7992319796 | 51.1550807028 |
| 12 | GAPN | C9' | 35.9185114510 | 26.7651554374 | 51.9643549532 |
| 12 | GAPN | H9' | 34.8279314739 | 26.9186074147 | 52.1310591529 |
| 12 | GAPN | H9'' | 36.3516171477 | 27.7189039917 | 51.5845147498 |
| 12 | GAPN | O10' | 36.5570789929 | 26.4037866176 | 53.1729747851 |
| 12 | GAPN | C11' | 36.4493120349 | 27.4536589069 | 54.1221194572 |
| 12 | GAPN | H11' | 35.3782063771 | 27.6491721855 | 54.3527282045 |
| 12 | GAPN | H11'' | 36.8959542890 | 28.3882133380 | 53.7127975744 |
| 12 | GAPN | C12' | 37.1901067712 | 27.0506654134 | 55.3966788954 |
| 12 | GAPN | H12' | 38.2591825342 | 26.8576851654 | 55.1502918618 |
| 12 | GAPN | H12'' | 36.7396141020 | 26.1148337244 | 55.7985160370 |
| 12 | GAPN | O13' | 37.0805955432 | 28.1037082535 | 56.3448542675 |
| 12 | GAPN | H13' | 37.5502863673 | 27.8283050513 | 57.1375434530 |
| 12 | GAPN | C3' | 31.9280721446 | 25.8471840766 | 45.1796345339 |
| 12 | GAPN | O3' | 31.0253133764 | 26.4978234543 | 44.6525308774 |
| 12 | GAPN | C4' | 31.9887818400 | 24.3405182739 | 44.9205239731 |
| 12 | GAPN | H4' | 31.9032311049 | 23.8140245005 | 45.8821192497 |
| 12 | GAPN | H4'' | 32.9772362054 | 24.0861232963 | 44.4851375623 |
| 12 | GAPN | N9 | 30.9477625431 | 23.9240397156 | 43.9821268829 |
| 12 | GAPN | C5 | 29.9306956381 | 23.4120646163 | 42.1122445862 |
| 12 | GAPN | N7 | 29.0019734543 | 23.2057673664 | 43.1210807545 |
| 12 | GAPN | C8 | 29.6472811240 | 23.5221008020 | 44.2150936820 |
| 12 | GAPN | H8 | 29.2227195661 | 23.4631914369 | 45.2201380759 |
| 12 | GAPN | N1 | 31.0021154382 | 23.5114705063 | 40.0236480217 |
| 12 | GAPN | C2 | 32.0585023768 | 24.0255426494 | 40.6529472397 |
| 12 | GAPN | H2 | 32.9169771032 | 24.2694361887 | 40.0168489844 |
| 12 | GAPN | N3 | 32.2298321425 | 24.2301990851 | 41.9584324752 |
| 12 | GAPN | C4 | 31.1187848105 | 23.8831044462 | 42.6309138607 |
| 12 | GAPN | C6 | 29.8892728681 | 23.1872496718 | 40.7271961757 |
| 12 | GAPN | N6 | 28.8151413250 | 22.6598189010 | 40.1092196466 |
| 12 | GAPN | H61 | 27.9818202855 | 22.4563130740 | 40.6138541526 |
| 12 | GAPN | H62 | 28.8551760023 | 22.3903866870 | 39.1383918623 |
| 13 | GTPN | C | 33.2840963449 | 32.0759529775 | 40.9585273107 |
| 13 | GTPN | O1' | 32.9875782943 | 33.2556253295 | 41.0912641973 |
| 13 | GTPN | C2' | 33.0903695567 | 31.1266856768 | 42.0525623286 |
| 13 | GTPN | H2' | 32.1572482681 | 31.3593504400 | 42.5461293617 |
| 13 | GTPN | H2'' | 33.9350836760 | 31.3561740085 | 42.6718628746 |
| 13 | GTPN | N2' | 33.1412070909 | 29.7118219832 | 41.6767645462 |
| 13 | GTPN | C5' | 34.3237274472 | 28.9650681805 | 42.0923152786 |
| 13 | GTPN | H5' | 35.1676931331 | 29.5041452619 | 41.7109007718 |
| 13 | GTPN | H5'' | 34.3069188420 | 27.9402567904 | 41.7553912635 |
| 13 | GTPN | N | 33.2572703646 | 28.4533842736 | 44.2016010041 |
| 13 | GTPN | H1' | 32.8686266545 | 27.6084600922 | 43.8552062646 |
| 13 | GTPN | C6' | 34.4403863288 | 28.9662298004 | 43.6051606559 |
| 13 | GTPN | H6' | 34.5354251753 | 30.0051477920 | 43.8750350593 |
| 13 | GTPN | C6'' | 35.6942929796 | 28.3197181092 | 44.2138757155 |
| 13 | GTPN | H6'' | 36.4485994575 | 28.0749448106 | 43.4535923964 |
| 13 | GTPN | H6''' | 35.3987508863 | 27.3796585445 | 44.7033549338 |
| 13 | GTPN | O7' | 36.2298796813 | 29.3158350114 | 45.2541910566 |
| 13 | GTPN | C8' | 37.1716039121 | 28.7967364976 | 46.1737689184 |
| 13 | GTPN | H8' | 37.9333260468 | 28.1690754665 | 45.6712956930 |
| 13 | GTPN | H8'' | 36.6792026003 | 28.2081439445 | 46.9799848728 |
| 13 | GTPN | C9' | 37.8012817236 | 30.0386172411 | 46.7973468942 |
| 13 | GTPN | H9' | 37.0027263335 | 30.5868873398 | 47.3495015601 |
| 13 | GTPN | H9'' | 38.2034475433 | 30.7159658666 | 46.0098944633 |
| 13 | GTPN | O10' | 38.8343237444 | 29.6385282102 | 47.6757596353 |
| 13 | GTPN | C11' | 39.3803579084 | 30.7470763726 | 48.3744451456 |
| 13 | GTPN | H11' | 38.5732760425 | 31.3168367715 | 48.8889058250 |
| 13 | GTPN | H11'' | 39.9017793250 | 31.4318808142 | 47.6690660925 |
| 13 | GTPN | C12' | 40.3684238682 | 30.2152395265 | 49.4130873432 |
| 13 | GTPN | H12' | 41.2018236612 | 29.6894873389 | 48.8940416274 |
| 13 | GTPN | H12'' | 39.8443139816 | 29.4865368670 | 50.0736822348 |
| 13 | GTPN | O13' | 40.8622786362 | 31.3088065365 | 50.1740490067 |
| 13 | GTPN | H13' | 41.4261479071 | 30.9495681457 | 50.8653323972 |
| 13 | GTPN | C3' | 31.9603293370 | 29.1419112138 | 41.3023166571 |
| 13 | GTPN | O3' | 30.9525647168 | 29.8182544018 | 41.1151031942 |
| 13 | GTPN | C4' | 31.9339968193 | 27.6207536882 | 41.1262827348 |
| 13 | GTPN | H4' | 32.1100541525 | 27.1606081399 | 42.1188146893 |
| 13 | GTPN | H4'' | 32.7901699872 | 27.3452116387 | 40.4714857071 |
| 13 | GTPN | N1 | 30.6810552059 | 27.0905255653 | 40.5875298310 |
| 13 | GTPN | C6 | 29.6076249866 | 26.8393702890 | 41.4240360463 |
| 13 | GTPN | H6 | 29.7752208354 | 27.0878209349 | 42.4739922545 |
| 13 | GTPN | C2 | 30.5484181462 | 26.9469549588 | 39.2395458140 |

| | | | | | |
|---|---|---|---|---|---|
| 13 | GTPN | O2 | 31.4830749835 | 27.2972308495 | 38.4282525302 |
| 13 | GTPN | N3 | 29.3944412151 | 26.4243917378 | 38.8138055995 |
| 13 | GTPN | H3 | 29.3146165568 | 26.2792399246 | 37.8124613215 |
| 13 | GTPN | C4 | 28.2843619852 | 26.0620437973 | 39.5806084552 |
| 13 | GTPN | O4 | 27.3195146541 | 25.5498844349 | 39.0722161435 |
| 13 | GTPN | C5 | 28.4491037687 | 26.3365262544 | 40.9970012977 |
| 13 | GTPN | C5M | 27.3183096327 | 26.0350676527 | 41.9216257323 |
| 13 | GTPN | H51 | 27.5936258376 | 26.2686710943 | 42.9748762931 |
| 13 | GTPN | H52 | 27.0464443115 | 24.9567376090 | 41.8749228964 |
| 13 | GTPN | H53 | 26.4176367448 | 26.6325481103 | 41.6585374022 |
| 14 | GGPN | C | 31.2838850008 | 35.2938142982 | 37.4069662002 |
| 14 | GGPN | O1' | 31.3135045446 | 36.5153553266 | 37.4169999301 |
| 14 | GGPN | C2' | 31.8514226749 | 34.4408935412 | 38.5069885866 |
| 14 | GGPN | H2' | 31.1647399154 | 34.4652946050 | 39.3492273815 |
| 14 | GGPN | H2'' | 32.7945028979 | 34.8871541512 | 38.7621629886 |
| 14 | GGPN | N2' | 32.1202948245 | 33.0389929762 | 38.0962380931 |
| 14 | GGPN | C5' | 33.4943511061 | 32.6053413432 | 37.7430347884 |
| 14 | GGPN | H5' | 33.8846400567 | 33.3028447324 | 37.0408295280 |
| 14 | GGPN | H5'' | 33.4866743158 | 31.6189403565 | 37.3318592163 |
| 14 | GGPN | N | 33.8626310624 | 31.6052548932 | 39.8458053023 |
| 14 | GGPN | H1' | 33.9248807998 | 30.6377028485 | 39.6534570746 |
| 14 | GGPN | C6' | 34.4662107496 | 32.4967930724 | 38.9088981246 |
| 14 | GGPN | H6' | 34.5866531338 | 33.4772558442 | 39.3433511045 |
| 14 | GGPN | C6'' | 35.8919226020 | 32.0192685320 | 38.4698243313 |
| 14 | GGPN | H6'' | 36.6251349456 | 32.8479416523 | 38.5498362506 |
| 14 | GGPN | H6''' | 35.8605225877 | 31.6878862397 | 37.4108070699 |
| 14 | GGPN | O7' | 36.3436433293 | 30.8270555981 | 39.3529910288 |
| 14 | GGPN | C8' | 37.6107064668 | 31.0358006612 | 39.9806440383 |
| 14 | GGPN | H8' | 37.7857689688 | 32.1065099651 | 40.2224615077 |
| 14 | GGPN | H8'' | 38.4245561652 | 30.6733600915 | 39.3136660991 |
| 14 | GGPN | C9' | 37.6298531619 | 30.2240759235 | 41.2824830925 |
| 14 | GGPN | H9' | 37.1833007848 | 29.2381594495 | 41.0218281134 |
| 14 | GGPN | H9'' | 36.9779703157 | 30.7170947212 | 42.0384375415 |
| 14 | GGPN | O10' | 38.9113746197 | 29.9936741330 | 41.8463732519 |
| 14 | GGPN | C11' | 38.7940671603 | 28.9714924531 | 42.8236955514 |
| 14 | GGPN | H11' | 38.2916771288 | 28.0963382008 | 42.3470829626 |
| 14 | GGPN | H11'' | 38.1674462493 | 29.3377104966 | 43.6635635580 |
| 14 | GGPN | C12' | 40.1108847675 | 28.4465753837 | 43.3895797091 |
| 14 | GGPN | H12' | 40.5781289849 | 29.1952206926 | 44.0677408735 |
| 14 | GGPN | H12'' | 40.8028870687 | 28.2386532626 | 42.5432062516 |
| 14 | GGPN | O13' | 39.8163819251 | 27.2372198593 | 44.0864954051 |
| 14 | GGPN | H13' | 39.6790907818 | 27.4673266282 | 45.0094630646 |
| 14 | GGPN | C3' | 31.0940600911 | 32.1659122191 | 38.0752813986 |
| 14 | GGPN | O3' | 29.9198197393 | 32.5799403885 | 38.2318218559 |
| 14 | GGPN | C4' | 31.3064040299 | 30.6748689356 | 37.7980332377 |
| 14 | GGPN | H4' | 31.8707541225 | 30.2040755843 | 38.6268669446 |
| 14 | GGPN | H4'' | 31.8646907003 | 30.5392959723 | 36.8517639738 |
| 14 | GGPN | N9 | 30.0015561484 | 30.0606969715 | 37.6478874289 |
| 14 | GGPN | C4 | 29.4011588334 | 29.6971678396 | 36.4748716938 |
| 14 | GGPN | N2 | 29.3340082155 | 29.5779950125 | 33.0102121140 |
| 14 | GGPN | H21 | 30.1927279598 | 30.0070430951 | 32.7600679064 |
| 14 | GGPN | H22 | 28.6764149572 | 29.3115398961 | 32.2980355239 |
| 14 | GGPN | N3 | 29.9127147522 | 29.8444278310 | 35.2312035622 |
| 14 | GGPN | C2 | 29.0611766810 | 29.4276992047 | 34.2965731081 |
| 14 | GGPN | N1 | 27.8389635321 | 28.8744856906 | 34.5860282715 |
| 14 | GGPN | H1 | 27.2711628896 | 28.5357780979 | 33.8258583082 |
| 14 | GGPN | C6 | 27.3030613228 | 28.7008236249 | 35.8545464886 |
| 14 | GGPN | O6 | 26.1843999968 | 28.2037578156 | 36.0114887957 |
| 14 | GGPN | C5 | 28.1681122100 | 29.2022805404 | 36.8505726312 |
| 14 | GGPN | N7 | 27.9777982975 | 29.3187746138 | 38.2187211704 |
| 14 | GGPN | C8 | 29.0930580630 | 29.8427548693 | 38.6494265949 |
| 14 | GGPN | H8 | 29.3222288581 | 30.1414302801 | 39.6726505477 |
| 15 | GCPN | C | 27.5858639667 | 36.8515168782 | 32.7214107740 |
| 15 | GCPN | O1' | 27.3492445527 | 37.9878735869 | 32.3577351374 |
| 15 | GCPN | C2' | 28.4175610002 | 36.6689264407 | 33.9714481596 |
| 15 | GCPN | H2' | 27.8756029901 | 37.1256810832 | 34.7882739854 |
| 15 | GCPN | H2'' | 29.2875853777 | 37.2613679024 | 33.7434075188 |
| 15 | GCPN | N2' | 28.9120346794 | 35.3451211905 | 34.3740897224 |
| 15 | GCPN | C5' | 30.2693851015 | 35.0004965012 | 33.9526248938 |
| 15 | GCPN | H5' | 30.6899296121 | 35.8828037091 | 33.5064678860 |
| 15 | GCPN | H5'' | 30.1717206281 | 34.2136294818 | 33.2197884355 |
| 15 | GCPN | N | 30.7789000419 | 34.5801497981 | 36.3906013776 |
| 15 | GCPN | H1' | 30.2947804832 | 33.7788809235 | 36.7124901983 |

| | | | | | |
|---|---|---|---|---|---|
| 15 | GCPN | C6' | 31.2992617798 | 34.5816467912 | 35.0319919054 |
| 15 | GCPN | H6' | 31.4798066117 | 33.5217595137 | 34.8680473214 |
| 15 | GCPN | C6'' | 32.6959333610 | 35.2527847134 | 34.7497021274 |
| 15 | GCPN | H6'' | 32.5327588580 | 36.3181613488 | 34.4847599691 |
| 15 | GCPN | H6''' | 33.2417547303 | 34.7545326691 | 33.9289468593 |
| 15 | GCPN | O7' | 33.5775014013 | 35.1987109571 | 35.9780339757 |
| 15 | GCPN | C8' | 34.8591446472 | 35.7735905655 | 35.8394476088 |
| 15 | GCPN | H8' | 34.8808269230 | 36.6330744960 | 35.1353859992 |
| 15 | GCPN | H8'' | 35.5681045045 | 34.9932020666 | 35.4843222451 |
| 15 | GCPN | C9' | 35.1907285176 | 36.1836300840 | 37.2736701012 |
| 15 | GCPN | H9' | 34.6470354965 | 35.4984168080 | 37.9585855446 |
| 15 | GCPN | H9'' | 34.8332914581 | 37.2143280325 | 37.4873450382 |
| 15 | GCPN | O10' | 36.5658525155 | 36.0507479184 | 37.5504999102 |
| 15 | GCPN | C11' | 36.7886618950 | 35.9321582160 | 38.9452723907 |
| 15 | GCPN | H11' | 36.3061529449 | 35.0138054531 | 39.3505041926 |
| 15 | GCPN | H11'' | 36.3669683720 | 36.8114152930 | 39.4827027996 |
| 15 | GCPN | C12' | 38.2959462572 | 35.8376543159 | 39.1216076011 |
| 15 | GCPN | H12' | 38.7839781485 | 36.6765845121 | 38.5728113356 |
| 15 | GCPN | H12'' | 38.6606962884 | 34.8789530973 | 38.6872248161 |
| 15 | GCPN | O13' | 38.5934045173 | 35.9002651298 | 40.5053496985 |
| 15 | GCPN | H13' | 38.1089339641 | 36.6530011057 | 40.8573531853 |
| 15 | GCPN | C3' | 28.0610391752 | 34.4748162502 | 34.9599997250 |
| 15 | GCPN | O3' | 26.9489056544 | 34.8535303814 | 35.3863317368 |
| 15 | GCPN | C4' | 28.3945339752 | 32.9764079574 | 35.0613313196 |
| 15 | GCPN | H4' | 29.0840917972 | 32.7813876485 | 35.8995646427 |
| 15 | GCPN | H4'' | 28.9051393844 | 32.6652026204 | 34.1281514420 |
| 15 | GCPN | N1 | 27.1619452082 | 32.2147896263 | 35.2939500503 |
| 15 | GCPN | C6 | 26.6794196570 | 32.0399231597 | 36.5639297026 |
| 15 | GCPN | H6 | 27.3261895669 | 32.3892264230 | 37.3712285484 |
| 15 | GCPN | C5 | 25.5115197946 | 31.4135531167 | 36.7761217636 |
| 15 | GCPN | H5 | 25.1202080403 | 31.2388024852 | 37.7739582575 |
| 15 | GCPN | C2 | 26.3938595900 | 31.8551034780 | 34.1882080089 |
| 15 | GCPN | O2 | 26.7824495150 | 32.1513449406 | 33.0412532603 |
| 15 | GCPN | N3 | 25.2410425735 | 31.1856901543 | 34.3918278188 |
| 15 | GCPN | C4 | 24.7908779722 | 30.9599956122 | 35.6385029402 |
| 15 | GCPN | N4 | 23.6467438624 | 30.2968206580 | 35.8102712191 |
| 15 | GCPN | H41 | 23.2548966323 | 29.8625376066 | 34.9872722756 |
| 15 | GCPN | H42 | 23.4855713330 | 29.8198688291 | 36.6658817539 |
| 16 | GCPN | C | 22.5325018552 | 37.5195903824 | 29.6309850523 |
| 16 | GCPN | C2' | 23.8181708149 | 37.5823890932 | 30.4702166419 |
| 16 | GCPN | H2' | 23.6901138369 | 38.3817953154 | 31.1888542440 |
| 16 | GCPN | H2'' | 24.5788968241 | 37.8270913858 | 29.7499393381 |
| 16 | GCPN | N2' | 24.2705866371 | 36.3564250327 | 31.1567258665 |
| 16 | GCPN | C5' | 25.2219582864 | 35.5256789257 | 30.4065782694 |
| 16 | GCPN | H5' | 24.9576811097 | 35.6253904691 | 29.3699742033 |
| 16 | GCPN | H5'' | 25.1225426589 | 34.4968839389 | 30.6816782648 |
| 16 | GCPN | N | 27.1373131892 | 35.7889941240 | 31.9960777849 |
| 16 | GCPN | H1' | 27.1567233544 | 34.8614130839 | 32.3320821975 |
| 16 | GCPN | C6' | 26.6924620940 | 35.9762267858 | 30.6192811873 |
| 16 | GCPN | H6' | 26.6726300350 | 37.0288209933 | 30.3755616938 |
| 16 | GCPN | C6'' | 27.7401132228 | 35.3706374267 | 29.6428274223 |
| 16 | GCPN | H6'' | 27.5276085117 | 35.6192070433 | 28.5847745936 |
| 16 | GCPN | H6''' | 27.7572904961 | 34.2736077000 | 29.8045864874 |
| 16 | GCPN | O7' | 29.0920748446 | 35.9711845375 | 30.0639322097 |
| 16 | GCPN | C8' | 30.2670069112 | 35.4560106107 | 29.4548401148 |
| 16 | GCPN | H8' | 30.4453158338 | 35.8891857220 | 28.4476482052 |
| 16 | GCPN | H8'' | 30.2832162867 | 34.3444387109 | 29.4195509650 |
| 16 | GCPN | C9' | 31.2854134528 | 36.0142008867 | 30.4464994539 |
| 16 | GCPN | H9' | 31.0206129477 | 35.5572669630 | 31.4249826282 |
| 16 | GCPN | H9'' | 31.1268513211 | 37.1113337713 | 30.5487884711 |
| 16 | GCPN | O10' | 32.6534302830 | 35.7776479264 | 30.1838241741 |
| 16 | GCPN | C11' | 33.2909108247 | 35.5450434835 | 31.4229155027 |
| 16 | GCPN | H11' | 32.9260151756 | 34.5703176278 | 31.8177892117 |
| 16 | GCPN | H11'' | 33.0044025616 | 36.3529935334 | 32.1429733686 |
| 16 | GCPN | C12' | 34.8054059455 | 35.4837052097 | 31.3266315748 |
| 16 | GCPN | H12' | 35.1476615319 | 36.2764982238 | 30.6217803536 |
| 16 | GCPN | H12'' | 35.1065161342 | 34.4803033871 | 30.9465875661 |
| 16 | GCPN | O13' | 35.3192587714 | 35.7098672454 | 32.6342276092 |
| 16 | GCPN | H13' | 36.2575260775 | 35.8986015693 | 32.5524828180 |
| 16 | GCPN | C3' | 23.9423715233 | 36.1180622802 | 32.4611183529 |
| 16 | GCPN | O3' | 23.2422238677 | 36.8722404765 | 33.1566380636 |
| 16 | GCPN | C4' | 24.4612627392 | 34.8502626990 | 33.1892648931 |
| 16 | GCPN | H4' | 25.1910809139 | 35.2156267910 | 33.9286397896 |

| | | | | | |
|---|---|---|---|---|---|
| 16 | GCPN | H4'' | 25.0187535527 | 34.1692478776 | 32.5431552183 |
| 16 | GCPN | N1 | 23.4219224273 | 34.0639778628 | 33.8565216758 |
| 16 | GCPN | C6 | 23.2745568825 | 34.0970720543 | 35.2109020422 |
| 16 | GCPN | H6 | 23.9873386480 | 34.7306848668 | 35.7388004659 |
| 16 | GCPN | C5 | 22.3191884040 | 33.3664149503 | 35.8150136918 |
| 16 | GCPN | H5 | 22.1823321893 | 33.3597101336 | 36.8924070146 |
| 16 | GCPN | C2 | 22.6394322516 | 33.2276950939 | 33.0716600037 |
| 16 | GCPN | O2 | 22.8582563141 | 33.1260831357 | 31.8537655471 |
| 16 | GCPN | N3 | 21.6604565994 | 32.5086223743 | 33.6604837652 |
| 16 | GCPN | C4 | 21.4772515143 | 32.5721503318 | 34.9895419641 |
| 16 | GCPN | N4 | 20.4872905542 | 31.8458466696 | 35.5118138803 |
| 16 | GCPN | H41 | 19.9964061888 | 31.2485233139 | 34.8679780976 |
| 16 | GCPN | H42 | 20.4096222520 | 31.6530980965 | 36.4839502354 |
| 16 | GCPN | OT1 | 21.8999931054 | 36.4375777057 | 29.5303877019 |
| 16 | GCPN | OT2 | 22.1932564289 | 38.5851514119 | 29.0429908552 |

Model 9

| | | | | | |
|---|---|---|---|---|---|
| 1 | GGPN | N | 15.0583647923 | 29.5725211568 | 25.7468491281 |
| 1 | GGPN | HT1 | 15.7461613098 | 30.0141246143 | 26.3831241359 |
| 1 | GGPN | HT2 | 14.1100337883 | 29.8018376566 | 26.1099409875 |
| 1 | GGPN | HT3 | 15.1180070244 | 30.0373403960 | 24.8172175801 |
| 1 | GGPN | C6' | 15.1140253815 | 28.0490033602 | 25.6276960364 |
| 1 | GGPN | H6' | 14.5246017372 | 27.5969528133 | 26.4251478340 |
| 1 | GGPN | C | 19.7278247127 | 26.5570455348 | 24.9617723803 |
| 1 | GGPN | O1' | 20.1169123064 | 25.7112584573 | 24.1685105829 |
| 1 | GGPN | C2' | 18.6362064113 | 26.2424077198 | 25.9496942627 |
| 1 | GGPN | H2' | 19.0990519123 | 25.8205890317 | 26.8299548466 |
| 1 | GGPN | H2'' | 18.0741830790 | 25.4861928392 | 25.4164672522 |
| 1 | GGPN | N2' | 17.7111398943 | 27.3357213899 | 26.3722975357 |
| 1 | GGPN | C5' | 16.5054028067 | 27.3168110971 | 25.5622319454 |
| 1 | GGPN | H5' | 16.1953751225 | 26.3164552590 | 25.7582527919 |
| 1 | GGPN | H5'' | 16.8752223588 | 27.3960540538 | 24.5636174228 |
| 1 | GGPN | C6'' | 14.4207955449 | 27.6677718293 | 24.2808827930 |
| 1 | GGPN | H6'' | 15.1545016321 | 27.3663672876 | 23.4909723178 |
| 1 | GGPN | H6''' | 13.8973083421 | 28.5600244473 | 23.8973581287 |
| 1 | GGPN | O7' | 13.3482852171 | 26.5780031115 | 24.3627887018 |
| 1 | GGPN | C8' | 12.6750514835 | 26.6198267542 | 23.1068484446 |
| 1 | GGPN | H8' | 13.4180819823 | 26.4666051956 | 22.2914532221 |
| 1 | GGPN | H8'' | 12.2473023644 | 27.6425279190 | 22.9868568001 |
| 1 | GGPN | C9' | 11.4927479592 | 25.6820498532 | 22.8672342664 |
| 1 | GGPN | H9' | 10.8177371932 | 25.6908330626 | 23.7503443377 |
| 1 | GGPN | H9'' | 11.8436728660 | 24.6444415321 | 22.6789950304 |
| 1 | GGPN | O10' | 10.8103007966 | 26.1953409603 | 21.7262097611 |
| 1 | GGPN | C11' | 9.6346894564 | 25.4492872273 | 21.4508631045 |
| 1 | GGPN | H11' | 8.9526120621 | 25.4983144708 | 22.3282581538 |
| 1 | GGPN | H11'' | 9.9102949526 | 24.3862024831 | 21.2760955774 |
| 1 | GGPN | C12' | 8.8698719410 | 25.9572794865 | 20.2186572158 |
| 1 | GGPN | H12' | 9.5290242373 | 25.9343741159 | 19.3220122345 |
| 1 | GGPN | H12'' | 8.5250572403 | 27.0023231435 | 20.3849768581 |
| 1 | GGPN | O13' | 7.7545576772 | 25.0924137585 | 20.0339831327 |
| 1 | GGPN | H13' | 7.2797311764 | 25.3702141518 | 19.2449901705 |
| 1 | GGPN | C3' | 18.1756829666 | 28.1650031916 | 27.3460096823 |
| 1 | GGPN | O3' | 19.2975831928 | 27.9642640816 | 27.7692801554 |
| 1 | GGPN | C4' | 17.4389161337 | 29.3401065721 | 27.9748420393 |
| 1 | GGPN | H4' | 16.3639615931 | 29.2784191224 | 27.8180514294 |
| 1 | GGPN | H4'' | 17.8557144978 | 30.2812410171 | 27.5468175262 |
| 1 | GGPN | N9 | 17.6581717770 | 29.3091327941 | 29.4153563794 |
| 1 | GGPN | C4 | 18.6044899809 | 30.0339503476 | 30.0795840728 |
| 1 | GGPN | N2 | 21.1825064291 | 32.3440055948 | 30.0449575577 |
| 1 | GGPN | H21 | 21.1750233930 | 32.7046496725 | 29.1199350298 |
| 1 | GGPN | H22 | 21.7141944436 | 32.8611624702 | 30.7238000962 |
| 1 | GGPN | N3 | 19.4740340351 | 30.8870145280 | 29.5103573760 |
| 1 | GGPN | C2 | 20.2680164141 | 31.4612436255 | 30.4126507992 |
| 1 | GGPN | N1 | 20.1724722498 | 31.2028920609 | 31.7577690083 |
| 1 | GGPN | H1 | 20.7869754686 | 31.7023231021 | 32.3855542380 |
| 1 | GGPN | C6 | 19.2669728180 | 30.3406183063 | 32.3743847362 |
| 1 | GGPN | O6 | 19.2294281613 | 30.1861416111 | 33.6004629269 |
| 1 | GGPN | C5 | 18.4317589134 | 29.7198778537 | 31.4125703517 |
| 1 | GGPN | N7 | 17.4025618958 | 28.8006752913 | 31.5759932978 |
| 1 | GGPN | C8 | 16.9832633849 | 28.5746529337 | 30.3668808709 |
| 1 | GGPN | H8 | 16.1838378987 | 27.8771714794 | 30.1180941975 |
| 2 | GGPN | C | 25.0907581736 | 26.4115908012 | 24.7311745918 |
| 2 | GGPN | O1' | 25.7429779166 | 25.7258473282 | 23.9664889781 |
| 2 | GGPN | C2' | 23.6054068300 | 26.2860186417 | 24.9092300968 |
| 2 | GGPN | H2' | 23.4175872179 | 25.4299746368 | 25.5369884393 |
| 2 | GGPN | H2'' | 23.1753296474 | 26.1663649493 | 23.9281127868 |
| 2 | GGPN | N2' | 23.0427882294 | 27.4979321032 | 25.5357596608 |
| 2 | GGPN | C5' | 22.5132032213 | 28.5940904761 | 24.7216588768 |
| 2 | GGPN | H5' | 23.2334380296 | 28.7819367277 | 23.9548911286 |
| 2 | GGPN | H5'' | 22.3860237247 | 29.4841833094 | 25.3115875635 |
| 2 | GGPN | N | 20.2344536859 | 27.7997313032 | 24.9920505355 |
| 2 | GGPN | H1' | 19.9391281978 | 28.4109474900 | 25.7187559537 |
| 2 | GGPN | C6' | 21.1737674074 | 28.3108628434 | 24.0271186329 |
| 2 | GGPN | H6' | 21.3555800678 | 27.5404669019 | 23.2971253414 |
| 2 | GGPN | C6'' | 20.6558451892 | 29.5456002649 | 23.2191086893 |
| 2 | GGPN | H6'' | 20.9871676092 | 29.5257791443 | 22.1555836707 |
| 2 | GGPN | H6''' | 21.0691378454 | 30.4689439831 | 23.6762077159 |
| 2 | GGPN | O7' | 19.1158819564 | 29.6191319024 | 23.3156547794 |

| | | | | | |
|---|---|---|---|---|---|
| 2 | GGPN | C8' | 18.4934599365 | 29.9293568650 | 22.0713082297 |
| 2 | GGPN | H8' | 18.8999631675 | 29.2854462074 | 21.2618075489 |
| 2 | GGPN | H8'' | 18.6469535768 | 30.9983920057 | 21.8096624474 |
| 2 | GGPN | C9' | 17.0001315732 | 29.6313480758 | 22.2615216447 |
| 2 | GGPN | H9' | 16.7751914982 | 29.9122290386 | 23.3150899442 |
| 2 | GGPN | H9'' | 16.8814839074 | 28.5273522352 | 22.1679070083 |
| 2 | GGPN | O10' | 16.0550927110 | 30.2947400617 | 21.4165899424 |
| 2 | GGPN | C11' | 14.7551490994 | 29.9291564666 | 21.8613208803 |
| 2 | GGPN | H11' | 14.7189723160 | 30.2307059479 | 22.9293066298 |
| 2 | GGPN | H11'' | 14.6588602031 | 28.8246145404 | 21.7646202948 |
| 2 | GGPN | C12' | 13.4558543360 | 30.4952839912 | 21.2555987410 |
| 2 | GGPN | H12' | 13.2246587888 | 30.0009938692 | 20.2849904346 |
| 2 | GGPN | H12'' | 13.5378384938 | 31.5935155073 | 21.1001340965 |
| 2 | GGPN | O13' | 12.4445903583 | 30.1951695858 | 22.2269605668 |
| 2 | GGPN | H13' | 11.6143850223 | 30.0205391415 | 21.7623519464 |
| 2 | GGPN | C3' | 22.9936929011 | 27.5160717040 | 26.9039890743 |
| 2 | GGPN | O3' | 23.4587883960 | 26.6020762850 | 27.5617611922 |
| 2 | GGPN | C4' | 22.3964768234 | 28.7367462564 | 27.5892097784 |
| 2 | GGPN | H4' | 21.3957274923 | 28.9593238805 | 27.1853777271 |
| 2 | GGPN | H4'' | 23.0532151496 | 29.6097696565 | 27.3787898642 |
| 2 | GGPN | N9 | 22.2769454820 | 28.5181841118 | 29.0205880949 |
| 2 | GGPN | C4 | 22.9890993123 | 29.1778332036 | 29.9706697694 |
| 2 | GGPN | N2 | 25.4524602513 | 31.4185694587 | 30.8764307743 |
| 2 | GGPN | H21 | 25.8530261043 | 31.6403141611 | 29.9989616096 |
| 2 | GGPN | H22 | 25.8630481445 | 31.7256881445 | 31.7394875227 |
| 2 | GGPN | N3 | 23.9431054336 | 30.1042569776 | 29.7450651194 |
| 2 | GGPN | C2 | 24.4146763005 | 30.6021629356 | 30.8820610718 |
| 2 | GGPN | N1 | 23.9625448036 | 30.2148204705 | 32.1143864875 |
| 2 | GGPN | H1 | 24.3006072225 | 30.7113729679 | 32.9244694538 |
| 2 | GGPN | C6 | 22.9722825553 | 29.2760418028 | 32.3644292412 |
| 2 | GGPN | O6 | 22.6146260770 | 28.9709446660 | 33.5028192173 |
| 2 | GGPN | C5 | 22.5154894523 | 28.6934371110 | 31.1705582371 |
| 2 | GGPN | N7 | 21.5844063919 | 27.6903203749 | 30.9769077938 |
| 2 | GGPN | C8 | 21.4735044152 | 27.6142102125 | 29.6814668458 |
| 2 | GGPN | H8 | 20.8416482171 | 26.9026944646 | 29.1488996781 |
| 3 | GCPN | C | 30.2674317102 | 25.2981006039 | 26.5948546550 |
| 3 | GCPN | O1' | 31.0228546520 | 24.3967280428 | 26.2537963453 |
| 3 | GCPN | C2' | 28.8197262180 | 25.3119617710 | 26.2049452763 |
| 3 | GCPN | H2' | 28.3425431398 | 24.4457535901 | 26.6353704820 |
| 3 | GCPN | H2'' | 28.7997957443 | 25.2525794242 | 25.1278385758 |
| 3 | GCPN | N2' | 28.0930143369 | 26.5224164495 | 26.6269825799 |
| 3 | GCPN | C5' | 28.1050913978 | 27.6221860807 | 25.6541775210 |
| 3 | GCPN | H5' | 28.6216974248 | 27.2461864664 | 24.7824545639 |
| 3 | GCPN | H5'' | 28.6620004044 | 28.4246194083 | 26.1177063916 |
| 3 | GCPN | N | 25.6127486458 | 27.3906418622 | 25.4769048110 |
| 3 | GCPN | H1' | 25.0224978824 | 27.7135363291 | 26.2047481135 |
| 3 | GCPN | C6' | 26.7443194523 | 28.1988530832 | 25.1505761222 |
| 3 | GCPN | H6' | 26.5589845749 | 29.0729574314 | 25.7602136296 |
| 3 | GCPN | C6'' | 26.7287911341 | 28.7705390301 | 23.6882665022 |
| 3 | GCPN | H6'' | 27.3843390722 | 28.1407318839 | 23.0529533271 |
| 3 | GCPN | H6''' | 27.0886077378 | 29.8229358512 | 23.6783432679 |
| 3 | GCPN | O7' | 25.2747446824 | 28.7340301252 | 23.1463683642 |
| 3 | GCPN | C8' | 25.1281877962 | 28.7472459956 | 21.7294559298 |
| 3 | GCPN | H8' | 26.0870382697 | 28.5338778165 | 21.2144450839 |
| 3 | GCPN | H8'' | 24.7923150523 | 29.7662370503 | 21.4354973874 |
| 3 | GCPN | C9' | 24.0407131774 | 27.7343883383 | 21.2812054887 |
| 3 | GCPN | H9' | 23.5075208705 | 27.3649748736 | 22.1791428536 |
| 3 | GCPN | H9'' | 24.5160304462 | 26.8495420069 | 20.8040102758 |
| 3 | GCPN | O10' | 23.0805431681 | 28.3212336044 | 20.4072835216 |
| 3 | GCPN | C11' | 21.9265778501 | 27.4981223799 | 20.2331658617 |
| 3 | GCPN | H11' | 21.4505955979 | 27.2943772043 | 21.2165246447 |
| 3 | GCPN | H11'' | 22.2013604957 | 26.5211437111 | 19.7795213045 |
| 3 | GCPN | C12' | 20.9010702540 | 28.2242487079 | 19.3504045747 |
| 3 | GCPN | H12' | 21.2558435962 | 28.2395101125 | 18.2959033898 |
| 3 | GCPN | H12'' | 20.8096205572 | 29.2793296995 | 19.6960162493 |
| 3 | GCPN | O13' | 19.6286795482 | 27.5940380091 | 19.4504613227 |
| 3 | GCPN | H13' | 19.6897428717 | 26.7389258496 | 19.0116569644 |
| 3 | GCPN | C3' | 27.5586485727 | 26.5282909847 | 27.8945207868 |
| 3 | GCPN | O3' | 27.7652483508 | 25.5818456609 | 28.6543514589 |
| 3 | GCPN | C4' | 26.7039358677 | 27.6842548712 | 28.4755675696 |
| 3 | GCPN | H4' | 25.7819840296 | 27.7740593975 | 27.8827855070 |
| 3 | GCPN | H4'' | 27.2596814464 | 28.6357103524 | 28.3579211018 |
| 3 | GCPN | N1 | 26.2388546037 | 27.4287717392 | 29.8525929668 |

| | | | | | |
|---|---|---|---|---|---|
| 3 | GCPN | C6 | 25.2457363147 | 26.5130221752 | 30.1027119926 |
| 3 | GCPN | H6 | 24.8286674475 | 26.0024319197 | 29.2308774321 |
| 3 | GCPN | C5 | 24.8196436327 | 26.2667494539 | 31.3470216736 |
| 3 | GCPN | H5 | 24.0177515979 | 25.5624281900 | 31.5570760431 |
| 3 | GCPN | C2 | 26.8854304915 | 28.0647162305 | 30.9185190167 |
| 3 | GCPN | O2 | 27.8514760651 | 28.8130403260 | 30.6935418627 |
| 3 | GCPN | N3 | 26.4439137859 | 27.8464943467 | 32.1787447129 |
| 3 | GCPN | C4 | 25.4372774482 | 26.9796563002 | 32.4130554329 |
| 3 | GCPN | N4 | 25.0046603577 | 26.7866961289 | 33.6604074758 |
| 3 | GCPN | H41 | 25.3478521385 | 27.3746173571 | 34.4009563171 |
| 3 | GCPN | H42 | 24.1531010861 | 26.3010057637 | 33.8293429658 |
| 4 | GAPN | C | 34.2965903981 | 24.1180790708 | 30.1556473401 |
| 4 | GAPN | O1' | 35.2973509664 | 23.5683835959 | 29.7142849555 |
| 4 | GAPN | C2' | 33.0668802187 | 24.2824091519 | 29.3423677135 |
| 4 | GAPN | H2' | 32.5097948341 | 23.3577834276 | 29.3244452557 |
| 4 | GAPN | H2'' | 33.4630099679 | 24.5268624077 | 28.3766669551 |
| 4 | GAPN | N2' | 32.1988296341 | 25.3909161733 | 29.7764915463 |
| 4 | GAPN | C5' | 32.3467727640 | 26.6779496261 | 29.0938236269 |
| 4 | GAPN | H5' | 33.3826781187 | 26.9500747257 | 29.1896783661 |
| 4 | GAPN | H5'' | 31.7099266646 | 27.4296870735 | 29.5302636665 |
| 4 | GAPN | N | 30.6863306363 | 26.3405842693 | 27.3290153110 |
| 4 | GAPN | H1' | 29.9935664978 | 26.9454020089 | 27.6975692464 |
| 4 | GAPN | C6' | 32.0656093410 | 26.6417818637 | 27.5939733173 |
| 4 | GAPN | H6' | 32.6651674227 | 25.8389495761 | 27.1920220524 |
| 4 | GAPN | C6'' | 32.6047606421 | 27.8987625647 | 26.8728294401 |
| 4 | GAPN | H6'' | 33.2522730407 | 28.5193959090 | 27.5296914802 |
| 4 | GAPN | H6''' | 31.7625279634 | 28.5334887458 | 26.5195046381 |
| 4 | GAPN | O7' | 33.4623319307 | 27.3995155586 | 25.7093763814 |
| 4 | GAPN | C8' | 33.0930313360 | 27.9450718794 | 24.4596301534 |
| 4 | GAPN | H8' | 33.5001364286 | 28.9755611541 | 24.3723040044 |
| 4 | GAPN | H8'' | 31.9885500777 | 27.9653351357 | 24.3328124416 |
| 4 | GAPN | C9' | 33.7130833603 | 27.0334388795 | 23.4020963744 |
| 4 | GAPN | H9' | 33.1735035063 | 26.0594569114 | 23.3914044737 |
| 4 | GAPN | H9'' | 34.7739052465 | 26.8370143594 | 23.6787288687 |
| 4 | GAPN | O10' | 33.6689961784 | 27.6249886717 | 22.1181641758 |
| 4 | GAPN | C11' | 34.3468876798 | 26.8028280659 | 21.1791910060 |
| 4 | GAPN | H11' | 33.8393737670 | 25.8151315620 | 21.1044325782 |
| 4 | GAPN | H11'' | 35.3967958083 | 26.6321471043 | 21.5093077738 |
| 4 | GAPN | C12' | 34.3524196185 | 27.4897241118 | 19.8137456040 |
| 4 | GAPN | H12' | 34.8720110423 | 28.4706693114 | 19.9056525155 |
| 4 | GAPN | H12'' | 33.3007869460 | 27.6662254671 | 19.4934362333 |
| 4 | GAPN | O13' | 35.0246421899 | 26.6580718879 | 18.8771137388 |
| 4 | GAPN | H13' | 35.0189920787 | 27.1140974873 | 18.0302046408 |
| 4 | GAPN | C3' | 31.2485056852 | 25.1283162615 | 30.7209371986 |
| 4 | GAPN | O3' | 31.2840608269 | 24.0935605419 | 31.3902822653 |
| 4 | GAPN | C4' | 30.1315933525 | 26.1405166533 | 30.9926111864 |
| 4 | GAPN | H4' | 29.5499312125 | 26.2984900162 | 30.0672537618 |
| 4 | GAPN | H4'' | 30.5908540026 | 27.1028211389 | 31.2994180464 |
| 4 | GAPN | N9 | 29.2708932208 | 25.6693061414 | 32.0745225462 |
| 4 | GAPN | C5 | 28.4482194675 | 25.3092968441 | 34.0710752159 |
| 4 | GAPN | N7 | 27.6099033133 | 24.6495731207 | 33.1830282186 |
| 4 | GAPN | C8 | 28.1414114926 | 24.8859077363 | 32.0089278424 |
| 4 | GAPN | H8 | 27.7465378794 | 24.5312066621 | 31.0531643161 |
| 4 | GAPN | N1 | 29.4221234427 | 26.1891113718 | 36.0269509082 |
| 4 | GAPN | C2 | 30.3972245311 | 26.6666601655 | 35.2540225481 |
| 4 | GAPN | H2 | 31.1824826048 | 27.2212872538 | 35.7788986669 |
| 4 | GAPN | N3 | 30.5115710342 | 26.6125627454 | 33.9255110527 |
| 4 | GAPN | C4 | 29.4890162612 | 25.9168424615 | 33.3993469998 |
| 4 | GAPN | C6 | 28.4087923290 | 25.4862277471 | 35.4659887215 |
| 4 | GAPN | N6 | 27.4182733968 | 25.0117241437 | 36.2414762923 |
| 4 | GAPN | H61 | 26.6770386206 | 24.4684739294 | 35.8594132433 |
| 4 | GAPN | H62 | 27.3773444078 | 25.2528944980 | 37.2180388980 |
| 5 | GTPN | C | 37.5554511088 | 22.7076637669 | 34.4170128937 |
| 5 | GTPN | O1' | 38.3295430186 | 21.7888274645 | 34.2018645417 |
| 5 | GTPN | C2' | 36.4773828372 | 23.0461292833 | 33.4623451447 |
| 5 | GTPN | H2' | 36.0303303780 | 22.1263863962 | 33.1146140196 |
| 5 | GTPN | H2'' | 37.0466780241 | 23.5240765094 | 32.6918507401 |
| 5 | GTPN | N2' | 35.4286055744 | 23.9765424706 | 33.9119676397 |
| 5 | GTPN | C5' | 35.5060177153 | 25.3359272371 | 33.3986514313 |
| 5 | GTPN | H5' | 36.4818519354 | 25.6860042179 | 33.6885041108 |
| 5 | GTPN | H5'' | 34.7310534540 | 25.9705294182 | 33.7983020273 |
| 5 | GTPN | N | 34.2606158473 | 24.6669478190 | 31.3689896801 |
| 5 | GTPN | H1' | 33.3664119259 | 24.8172203254 | 31.7794748647 |

| | | | | | |
|---|---|---|---|---|---|
| 5 | GTPN | C6' | 35.4124535889 | 25.3577727154 | 31.8683882583 |
| 5 | GTPN | H6' | 36.2857787583 | 24.8254636020 | 31.5214658695 |
| 5 | GTPN | C6'' | 35.5584811692 | 26.7435482503 | 31.2262415097 |
| 5 | GTPN | H6'' | 35.8299751663 | 27.5215344706 | 31.9584280799 |
| 5 | GTPN | H6''' | 34.5919192308 | 27.0485087016 | 30.7849281366 |
| 5 | GTPN | O7' | 36.6410424491 | 26.5859449583 | 30.1472685877 |
| 5 | GTPN | C8' | 36.5949804355 | 27.5503226236 | 29.1182837661 |
| 5 | GTPN | H8' | 36.6187112591 | 28.5713077255 | 29.5489787648 |
| 5 | GTPN | H8'' | 35.6805906372 | 27.4236838200 | 28.4988030606 |
| 5 | GTPN | C9' | 37.8432984155 | 27.3110760663 | 28.2666975697 |
| 5 | GTPN | H9' | 37.7739388572 | 26.3113093742 | 27.7797651136 |
| 5 | GTPN | H9'' | 38.7377883897 | 27.3123277545 | 28.9297996467 |
| 5 | GTPN | O10' | 37.9861491246 | 28.3180467531 | 27.2838378629 |
| 5 | GTPN | C11' | 39.1127972931 | 28.0559823065 | 26.4576636485 |
| 5 | GTPN | H11' | 39.0353305982 | 27.0366345272 | 26.0154363771 |
| 5 | GTPN | H11'' | 40.0506478737 | 28.1084423254 | 27.0548184355 |
| 5 | GTPN | C12' | 39.1474268722 | 29.0947969508 | 25.3361282237 |
| 5 | GTPN | H12' | 39.2629062565 | 30.1080748352 | 25.7830803588 |
| 5 | GTPN | H12'' | 38.1810852374 | 29.0524372158 | 24.7834016328 |
| 5 | GTPN | O13' | 40.2289943108 | 28.8130544731 | 24.4584108042 |
| 5 | GTPN | H13' | 40.1805760159 | 29.4444289216 | 23.7338168535 |
| 5 | GTPN | C3' | 34.2862833711 | 23.4484068132 | 34.4376928228 |
| 5 | GTPN | O3' | 34.2150251253 | 22.2549580292 | 34.7189033403 |
| 5 | GTPN | C4' | 33.0643803236 | 24.3627217110 | 34.6744294177 |
| 5 | GTPN | H4' | 32.7178839119 | 24.7294035806 | 33.6858330221 |
| 5 | GTPN | H4'' | 33.3977974794 | 25.2483607705 | 35.2557194362 |
| 5 | GTPN | N1 | 31.9374775028 | 23.7109788041 | 35.3591134183 |
| 5 | GTPN | C6 | 30.9933887300 | 22.9945109137 | 34.6627287340 |
| 5 | GTPN | H6 | 31.1721809169 | 22.9293052946 | 33.5843766066 |
| 5 | GTPN | C2 | 31.9417153158 | 23.7521171708 | 36.7443611241 |
| 5 | GTPN | O2 | 32.8308110115 | 24.2700529562 | 37.3925217216 |
| 5 | GTPN | N3 | 30.8489153103 | 23.1531840049 | 37.3053395484 |
| 5 | GTPN | H3 | 30.8362597077 | 23.1948670576 | 38.3212262043 |
| 5 | GTPN | C4 | 29.8171013062 | 22.4952741143 | 36.6807495023 |
| 5 | GTPN | O4 | 28.8722090299 | 22.0648956456 | 37.3396962221 |
| 5 | GTPN | C5 | 29.9435424057 | 22.4048104727 | 35.2448945306 |
| 5 | GTPN | C5M | 28.8921302404 | 21.6603226702 | 34.4896321219 |
| 5 | GTPN | H51 | 29.0784752221 | 21.6846043519 | 33.3931869978 |
| 5 | GTPN | H52 | 27.8903451642 | 22.1109896130 | 34.6722599959 |
| 5 | GTPN | H53 | 28.8528900135 | 20.5956768874 | 34.8115638952 |
| 6 | GGPN | C | 39.2233719186 | 19.4015415405 | 38.0658752745 |
| 6 | GGPN | O1' | 40.1523156619 | 18.6223321303 | 38.0048017997 |
| 6 | GGPN | C2' | 38.7881425429 | 20.2556630656 | 36.9150603734 |
| 6 | GGPN | H2' | 38.2710386991 | 19.6311981921 | 36.2028327507 |
| 6 | GGPN | H2'' | 39.6823085202 | 20.6887504769 | 36.4935641112 |
| 6 | GGPN | N2' | 37.9020046765 | 21.3399786953 | 37.3735897113 |
| 6 | GGPN | C5' | 38.4699898999 | 22.6671949231 | 37.6303240138 |
| 6 | GGPN | H5' | 39.3942057317 | 22.4967404507 | 38.1636213193 |
| 6 | GGPN | H5'' | 37.7862742252 | 23.2586601303 | 38.2162164985 |
| 6 | GGPN | N | 37.6660937570 | 23.5030621010 | 35.4851452571 |
| 6 | GGPN | H1' | 36.9724189039 | 24.1966924647 | 35.6386367307 |
| 6 | GGPN | C6' | 38.8088095621 | 23.4729008469 | 36.3620062244 |
| 6 | GGPN | H6' | 39.6193609706 | 22.9485360755 | 35.8703277666 |
| 6 | GGPN | C6'' | 39.3609542969 | 24.9002401992 | 36.6715696369 |
| 6 | GGPN | H6'' | 40.4458091851 | 24.9638307352 | 36.4296179960 |
| 6 | GGPN | H6''' | 39.2544624331 | 25.1169522268 | 37.7546174659 |
| 6 | GGPN | O7' | 38.5313017288 | 25.9486650098 | 35.8746880253 |
| 6 | GGPN | C8' | 39.3190159488 | 26.8856152966 | 35.1383592291 |
| 6 | GGPN | H8' | 40.2419358375 | 26.4114609425 | 34.7394492408 |
| 6 | GGPN | H8'' | 39.5963268520 | 27.7430638406 | 35.7893196118 |
| 6 | GGPN | C9' | 38.4645803290 | 27.4028930813 | 33.9657485431 |
| 6 | GGPN | H9' | 37.4466993345 | 27.5969129737 | 34.3745507085 |
| 6 | GGPN | H9'' | 38.3891371161 | 26.5804763376 | 33.2178777677 |
| 6 | GGPN | O10' | 38.9140069906 | 28.5941673130 | 33.3232695881 |
| 6 | GGPN | C11' | 37.9317066920 | 29.0073467076 | 32.3763328988 |
| 6 | GGPN | H11' | 36.9565526470 | 29.0914657215 | 32.9091947325 |
| 6 | GGPN | H11'' | 37.8495782245 | 28.2232389527 | 31.5949080789 |
| 6 | GGPN | C12' | 38.1494111601 | 30.3557156474 | 31.6662545001 |
| 6 | GGPN | H12' | 38.9349192754 | 30.2537052308 | 30.8848903434 |
| 6 | GGPN | H12'' | 38.4782460502 | 31.1162824532 | 32.4080549048 |
| 6 | GGPN | O13' | 36.9101130158 | 30.7660131273 | 31.0846291163 |
| 6 | GGPN | H13' | 37.0082783995 | 30.7163543738 | 30.1271131106 |
| 6 | GGPN | C3' | 36.5700940042 | 21.0460326335 | 37.4784242476 |

| | | | | | |
|---|---|---|---|---|---|
| 6 | GGPN | O3' | 36.1826320468 | 19.8854232784 | 37.3338228762 |
| 6 | GGPN | C4' | 35.5414013088 | 22.1143011778 | 37.8475477183 |
| 6 | GGPN | H4' | 35.4098653814 | 22.8018678065 | 36.9919537774 |
| 6 | GGPN | H4'' | 35.9021398926 | 22.6874385383 | 38.7252635600 |
| 6 | GGPN | N9 | 34.2823458657 | 21.4552721206 | 38.1881189996 |
| 6 | GGPN | C4 | 33.8024404403 | 21.2535169589 | 39.4552597603 |
| 6 | GGPN | N2 | 34.1744680270 | 21.4400369791 | 42.9014741437 |
| 6 | GGPN | H21 | 35.0667895923 | 21.8566905326 | 43.0136855190 |
| 6 | GGPN | H22 | 33.6656006772 | 21.1201981484 | 43.7077141219 |
| 6 | GGPN | N3 | 34.4139345939 | 21.6147357618 | 40.6060317518 |
| 6 | GGPN | C2 | 33.7108658787 | 21.2460519479 | 41.6763410437 |
| 6 | GGPN | N1 | 32.4991472728 | 20.6126762387 | 41.5961391877 |
| 6 | GGPN | H1 | 32.0147097348 | 20.3966192302 | 42.4537013067 |
| 6 | GGPN | C6 | 31.8414410756 | 20.2512410210 | 40.4285220774 |
| 6 | GGPN | O6 | 30.7506971623 | 19.6758360853 | 40.4658893005 |
| 6 | GGPN | C5 | 32.6046977898 | 20.5861563639 | 39.2854999806 |
| 6 | GGPN | N7 | 32.3660218256 | 20.3198677333 | 37.9465670203 |
| 6 | GGPN | C8 | 33.3920594560 | 20.8458940992 | 37.3287411206 |
| 6 | GGPN | H8 | 33.5711614559 | 20.8000440168 | 36.2533267811 |
| 7 | GCPN | C | 38.6624117796 | 15.4570561736 | 43.0384957690 |
| 7 | GCPN | O1' | 39.4027851126 | 14.5886305231 | 43.4767565559 |
| 7 | GCPN | C2' | 38.9535926292 | 16.0434680263 | 41.6840936171 |
| 7 | GCPN | H2' | 38.7701768920 | 15.2949645027 | 40.9301380857 |
| 7 | GCPN | H2'' | 40.0098544777 | 16.2449273221 | 41.7709565946 |
| 7 | GCPN | N2' | 38.3042549881 | 17.2942380470 | 41.2725974425 |
| 7 | GCPN | C5' | 39.2970405993 | 18.3626498763 | 41.1747474111 |
| 7 | GCPN | H5' | 40.1296118375 | 17.9457432893 | 40.6174572748 |
| 7 | GCPN | H5'' | 39.5495821063 | 18.5846558995 | 42.1982796468 |
| 7 | GCPN | N | 38.5005257263 | 19.6298283869 | 39.1759551625 |
| 7 | GCPN | H1' | 37.5810355719 | 19.9562465919 | 38.9925638321 |
| 7 | GCPN | C6' | 38.9972580127 | 19.7184792787 | 40.5191591037 |
| 7 | GCPN | H6' | 38.1907260889 | 20.1577383968 | 41.0867715600 |
| 7 | GCPN | C6'' | 40.1899056109 | 20.7059560126 | 40.7182497486 |
| 7 | GCPN | H6'' | 40.7996061968 | 20.3980340089 | 41.5846993309 |
| 7 | GCPN | H6''' | 39.7804848025 | 21.7265697295 | 40.8930573428 |
| 7 | GCPN | O7' | 41.1043305065 | 20.7179789190 | 39.5043966082 |
| 7 | GCPN | C8' | 41.9742328971 | 21.8227967026 | 39.4367496337 |
| 7 | GCPN | H8' | 42.8150339161 | 21.7143288172 | 40.1538320426 |
| 7 | GCPN | H8'' | 41.4201585954 | 22.7633491905 | 39.6438011375 |
| 7 | GCPN | C9' | 42.4484648764 | 21.7849867264 | 37.9867328453 |
| 7 | GCPN | H9' | 41.5666815557 | 21.5531458207 | 37.3472086405 |
| 7 | GCPN | H9'' | 43.1819125797 | 20.9595364221 | 37.8410285305 |
| 7 | GCPN | O10' | 42.9951357636 | 23.0193213296 | 37.5668892353 |
| 7 | GCPN | C11' | 42.9737127896 | 23.0980504350 | 36.1507117655 |
| 7 | GCPN | H11' | 41.9305471432 | 23.2624900240 | 35.8020300879 |
| 7 | GCPN | H11'' | 43.3350076540 | 22.1404394513 | 35.7128554370 |
| 7 | GCPN | C12' | 43.8562437859 | 24.2510258728 | 35.6803927009 |
| 7 | GCPN | H12' | 44.8862644740 | 24.1206710909 | 36.0835628089 |
| 7 | GCPN | H12'' | 43.4511577783 | 25.2147696707 | 36.0627418478 |
| 7 | GCPN | O13' | 43.8741232192 | 24.2622748272 | 34.2603819237 |
| 7 | GCPN | H13' | 44.2518648664 | 23.4253610536 | 33.9701359723 |
| 7 | GCPN | C3' | 37.0242682787 | 17.2335322077 | 40.7806448670 |
| 7 | GCPN | O3' | 36.5569624613 | 16.1517845256 | 40.4263245613 |
| 7 | GCPN | C4' | 36.0140439828 | 18.4151539568 | 40.6990629227 |
| 7 | GCPN | H4' | 36.1806268954 | 18.9554220077 | 39.7528891935 |
| 7 | GCPN | H4'' | 36.2016515309 | 19.1048621851 | 41.5470228926 |
| 7 | GCPN | N1 | 34.6199854231 | 17.9300510627 | 40.6918557259 |
| 7 | GCPN | C6 | 33.9652849727 | 17.6505919445 | 39.5174484227 |
| 7 | GCPN | H6 | 34.4985575609 | 17.9118235554 | 38.5984907225 |
| 7 | GCPN | C5 | 32.7199573299 | 17.1524146397 | 39.5173252858 |
| 7 | GCPN | H5 | 32.1702193537 | 16.9553467386 | 38.5995937297 |
| 7 | GCPN | C2 | 34.0269407314 | 17.6030482634 | 41.9172086005 |
| 7 | GCPN | O2 | 34.6707627626 | 17.7539824136 | 42.9693245412 |
| 7 | GCPN | N3 | 32.7604778353 | 17.1345503885 | 41.9279689210 |
| 7 | GCPN | C4 | 32.1052775671 | 16.9018326666 | 40.7784246721 |
| 7 | GCPN | N4 | 30.8575483110 | 16.4330058139 | 40.8214132839 |
| 7 | GCPN | H41 | 30.4161828693 | 16.4052162438 | 41.7288454121 |
| 7 | GCPN | H42 | 30.2465161232 | 16.5890169105 | 40.0542228387 |
| 8 | GCPN | C | 36.0235236861 | 11.3206167313 | 46.2337667680 |
| 8 | GCPN | C2' | 36.9578607168 | 12.1440412091 | 45.3358992570 |
| 8 | GCPN | H2' | 37.4306398903 | 11.4556784734 | 44.6520680906 |
| 8 | GCPN | H2'' | 37.6803186962 | 12.5779100631 | 46.0081843101 |
| 8 | GCPN | N2' | 36.3373014388 | 13.2450876112 | 44.5792983632 |

| | | | | | |
|---|---|---|---|---|---|
| 8 | GCPN | C5' | 36.1758276382 | 14.5067934033 | 45.2920600316 |
| 8 | GCPN | H5' | 36.1274262152 | 14.2168732061 | 46.3358372859 |
| 8 | GCPN | H5'' | 35.2590856562 | 14.9840838587 | 44.9752142550 |
| 8 | GCPN | N | 37.6159952275 | 15.9167734431 | 43.7567892808 |
| 8 | GCPN | H1' | 37.0118830655 | 16.5995161949 | 43.3576521051 |
| 8 | GCPN | C6' | 37.3641652581 | 15.5161202903 | 45.1395468944 |
| 8 | GCPN | H6' | 38.2435347090 | 14.9893455520 | 45.4968057828 |
| 8 | GCPN | C6'' | 37.1993555509 | 16.7261651033 | 46.1217483826 |
| 8 | GCPN | H6'' | 37.9140172942 | 16.6350824983 | 46.9684629993 |
| 8 | GCPN | H6''' | 36.1819551558 | 16.7107805013 | 46.5617172199 |
| 8 | GCPN | O7' | 37.3705639480 | 18.0735295032 | 45.3717889630 |
| 8 | GCPN | C8' | 38.2780163647 | 18.9918203608 | 45.9833014432 |
| 8 | GCPN | H8' | 38.9838472628 | 18.4918695286 | 46.6814353948 |
| 8 | GCPN | H8'' | 37.7193163039 | 19.7736281049 | 46.5443667405 |
| 8 | GCPN | C9' | 39.0603578068 | 19.6424843819 | 44.8342709438 |
| 8 | GCPN | H9' | 38.3585159211 | 19.7721350590 | 43.9766360212 |
| 8 | GCPN | H9'' | 39.8402696478 | 18.9170525764 | 44.5082894667 |
| 8 | GCPN | O10' | 39.6539449249 | 20.9059712816 | 45.1314040162 |
| 8 | GCPN | C11' | 40.3065087772 | 21.3798300314 | 43.9602571082 |
| 8 | GCPN | H11' | 39.5571372185 | 21.3750964412 | 43.1410583426 |
| 8 | GCPN | H11'' | 41.1124464332 | 20.6572317106 | 43.6992174297 |
| 8 | GCPN | C12' | 40.9307914684 | 22.7903986962 | 43.9935378411 |
| 8 | GCPN | H12' | 41.7593105442 | 22.8178454848 | 44.7342901037 |
| 8 | GCPN | H12'' | 40.1739681697 | 23.5519758911 | 44.2839632520 |
| 8 | GCPN | O13' | 41.4296318930 | 23.0784277496 | 42.6864431298 |
| 8 | GCPN | H13' | 41.8671613624 | 23.9386077890 | 42.7220529142 |
| 8 | GCPN | C3' | 35.9463855110 | 13.0378776874 | 43.2823007876 |
| 8 | GCPN | O3' | 36.1587305679 | 11.9870639400 | 42.6690743312 |
| 8 | GCPN | C4' | 35.2239627701 | 14.1201783705 | 42.4310529106 |
| 8 | GCPN | H4' | 35.8652100712 | 14.1734713300 | 41.5244111800 |
| 8 | GCPN | H4'' | 35.2314404868 | 15.1128696480 | 42.9081649989 |
| 8 | GCPN | N1 | 33.8448205153 | 13.7938194285 | 42.0378917208 |
| 8 | GCPN | C6 | 33.5061529838 | 13.5861261926 | 40.7291360261 |
| 8 | GCPN | H6 | 34.3395376553 | 13.6335259350 | 40.0266451824 |
| 8 | GCPN | C5 | 32.2369362606 | 13.3348241675 | 40.3667081925 |
| 8 | GCPN | H5 | 31.9414407818 | 13.1726774776 | 39.3337003250 |
| 8 | GCPN | C2 | 32.8436912741 | 13.8542545268 | 43.0112311277 |
| 8 | GCPN | O2 | 33.1224691726 | 14.2106804981 | 44.1634598388 |
| 8 | GCPN | N3 | 31.5778796774 | 13.5517032423 | 42.6684296101 |
| 8 | GCPN | C4 | 31.2552968639 | 13.2824963095 | 41.4006409177 |
| 8 | GCPN | N4 | 29.9901887706 | 12.9657915488 | 41.1281996603 |
| 8 | GCPN | H41 | 29.3560863119 | 12.9880756161 | 41.9157686473 |
| 8 | GCPN | H42 | 29.6145425068 | 12.9560711912 | 40.2115904622 |
| 8 | GCPN | OT1 | 34.7935612590 | 11.5925565106 | 46.2643710189 |
| 8 | GCPN | OT2 | 36.5556701355 | 10.4115605978 | 46.9291955691 |
| 9 | GGPN | N | 24.7249865241 | 11.7148692893 | 50.8629735416 |
| 9 | GGPN | HT1 | 24.7291172531 | 12.6525280915 | 50.4045493667 |
| 9 | GGPN | HT2 | 23.7401122649 | 11.4312278316 | 51.0642014532 |
| 9 | GGPN | HT3 | 25.1640273122 | 11.0085272676 | 50.2409989647 |
| 9 | GGPN | C6' | 25.4347518747 | 11.8087556403 | 52.1756306132 |
| 9 | GGPN | H6' | 24.8669190182 | 12.5445642520 | 52.7239602041 |
| 9 | GGPN | C | 27.1728686298 | 15.8609506951 | 52.4291896803 |
| 9 | GGPN | O1' | 26.8383685545 | 16.9241074793 | 52.9210705218 |
| 9 | GGPN | C2' | 26.1678819837 | 14.8338176682 | 52.0184602254 |
| 9 | GGPN | H2' | 25.4338303908 | 15.3396417857 | 51.4092842042 |
| 9 | GGPN | H2'' | 25.7479702567 | 14.4813596496 | 52.9471918755 |
| 9 | GGPN | N2' | 26.7363336994 | 13.6788809307 | 51.3000737801 |
| 9 | GGPN | C5' | 26.8346996642 | 12.4247864396 | 52.0288276600 |
| 9 | GGPN | H5' | 27.5283972083 | 11.7307147072 | 51.5710463934 |
| 9 | GGPN | H5'' | 27.1703729471 | 12.7052049383 | 53.0110938718 |
| 9 | GGPN | C6'' | 25.2643761074 | 10.5111621401 | 52.9636322355 |
| 9 | GGPN | H6'' | 25.8119203343 | 10.5396896671 | 53.9235849020 |
| 9 | GGPN | H6''' | 25.5710328176 | 9.5993758776 | 52.4075911479 |
| 9 | GGPN | O7' | 23.7615750875 | 10.5047935648 | 53.2041130929 |
| 9 | GGPN | C8' | 23.3493293104 | 9.8251246652 | 54.3722904758 |
| 9 | GGPN | H8' | 24.0036909967 | 10.0157634122 | 55.2544448907 |
| 9 | GGPN | H8'' | 23.3206810632 | 8.7333606001 | 54.1683496917 |
| 9 | GGPN | C9' | 21.9474466531 | 10.3750348306 | 54.6298932863 |
| 9 | GGPN | H9' | 21.4468124854 | 10.5575211292 | 53.6513983541 |
| 9 | GGPN | H9'' | 22.0011551236 | 11.3447337162 | 55.1733367985 |
| 9 | GGPN | O10' | 21.1778991676 | 9.4471234535 | 55.3584721182 |
| 9 | GGPN | C11' | 19.8342036034 | 9.8894212826 | 55.4723071706 |
| 9 | GGPN | H11' | 19.3934103959 | 10.0623888855 | 54.4640688255 |

| | | | | | |
|---|---|---|---|---|---|
| 9 | GGPN | H11'' | 19.7893678378 | 10.8418727798 | 56.0462126661 |
| 9 | GGPN | C12' | 19.0439706680 | 8.8020612812 | 56.1949875066 |
| 9 | GGPN | H12' | 19.5008651134 | 8.6303092415 | 57.1961349690 |
| 9 | GGPN | H12'' | 19.1056746616 | 7.8592255806 | 55.6045549375 |
| 9 | GGPN | O13' | 17.6923388130 | 9.2167911848 | 56.3279547647 |
| 9 | GGPN | H13' | 17.2242230684 | 8.5157547473 | 56.7920715924 |
| 9 | GGPN | C3' | 26.8791186031 | 13.7635758637 | 49.9518134534 |
| 9 | GGPN | O3' | 26.4837179571 | 14.7484817982 | 49.3456360542 |
| 9 | GGPN | C4' | 27.4976337209 | 12.5780911342 | 49.2307956664 |
| 9 | GGPN | H4' | 27.0257276381 | 11.6189087683 | 49.4943812567 |
| 9 | GGPN | H4'' | 28.5678742190 | 12.5251973009 | 49.5361087802 |
| 9 | GGPN | N9 | 27.3707810416 | 12.7337664047 | 47.7907241564 |
| 9 | GGPN | C4 | 28.4136402025 | 12.9971263655 | 46.9670833711 |
| 9 | GGPN | N2 | 31.8157519461 | 13.4234587286 | 46.5031529148 |
| 9 | GGPN | H21 | 32.1997182068 | 13.3128348987 | 47.4114905564 |
| 9 | GGPN | H22 | 32.4576190672 | 13.4461126198 | 45.7294122069 |
| 9 | GGPN | N3 | 29.6900803551 | 13.1520367969 | 47.3595594298 |
| 9 | GGPN | C2 | 30.5138669711 | 13.2768496535 | 46.3214026445 |
| 9 | GGPN | N1 | 30.0746548411 | 13.2496786737 | 45.0215274756 |
| 9 | GGPN | H1 | 30.7565507782 | 13.3399534613 | 44.2829487728 |
| 9 | GGPN | C6 | 28.7527551182 | 13.1127285786 | 44.5971938819 |
| 9 | GGPN | O6 | 28.4299756320 | 13.0885017494 | 43.4063012418 |
| 9 | GGPN | C5 | 27.8787653890 | 12.9930299358 | 45.6981762628 |
| 9 | GGPN | N7 | 26.5063818969 | 12.7823584069 | 45.7341054272 |
| 9 | GGPN | C8 | 26.2447195542 | 12.6453196171 | 46.9963350261 |
| 9 | GGPN | H8 | 25.2427763865 | 12.4892438561 | 47.3922089625 |
| 10 | GGPN | C | 30.0925319963 | 20.4065021377 | 51.8196696682 |
| 10 | GGPN | O1' | 29.7831452253 | 21.4223237732 | 52.4247966356 |
| 10 | GGPN | C2' | 29.2038622276 | 19.1977633287 | 51.8045093653 |
| 10 | GGPN | H2' | 28.3010317949 | 19.5048667758 | 51.2915703729 |
| 10 | GGPN | H2'' | 28.9740792424 | 18.9612472569 | 52.8282079158 |
| 10 | GGPN | N2' | 29.7840995724 | 17.9949192826 | 51.1690022436 |
| 10 | GGPN | C5' | 30.4503673626 | 16.9391763985 | 51.9671927176 |
| 10 | GGPN | H5' | 31.1508447748 | 17.4532701158 | 52.6075580005 |
| 10 | GGPN | H5'' | 30.9663434162 | 16.2338506247 | 51.3223540881 |
| 10 | GGPN | N | 28.4529302567 | 15.5024084324 | 52.2546118788 |
| 10 | GGPN | H1' | 28.6734933446 | 14.6599850470 | 51.7864537179 |
| 10 | GGPN | C6' | 29.5584379994 | 16.1136314087 | 52.9282145438 |
| 10 | GGPN | H6' | 29.1701455317 | 16.7994142018 | 53.6707671908 |
| 10 | GGPN | C6'' | 30.3994147464 | 15.0241072782 | 53.6849757475 |
| 10 | GGPN | H6'' | 30.8066644070 | 15.3875079457 | 54.6490440033 |
| 10 | GGPN | H6''' | 31.2759812857 | 14.7853879564 | 53.0484484202 |
| 10 | GGPN | O7' | 29.5867602190 | 13.6995511518 | 53.8639550302 |
| 10 | GGPN | C8' | 29.4868410493 | 13.2584605119 | 55.2229123333 |
| 10 | GGPN | H8' | 29.2632098674 | 14.1234366704 | 55.8790436130 |
| 10 | GGPN | H8'' | 30.4392486032 | 12.7862788863 | 55.5534823711 |
| 10 | GGPN | C9' | 28.3375804611 | 12.2230545446 | 55.3057858145 |
| 10 | GGPN | H9' | 28.4872116496 | 11.5497641937 | 54.4308047443 |
| 10 | GGPN | H9'' | 27.3919523393 | 12.7910603208 | 55.1555213215 |
| 10 | GGPN | O10' | 28.1950581806 | 11.3979001856 | 56.4676167108 |
| 10 | GGPN | C11' | 27.1668648766 | 10.4310040614 | 56.2173463367 |
| 10 | GGPN | H11' | 27.4561783334 | 9.8700944424 | 55.2995162544 |
| 10 | GGPN | H11'' | 26.2148284626 | 10.9774494883 | 56.0323069247 |
| 10 | GGPN | C12' | 26.8257594938 | 9.3296274524 | 57.2477354874 |
| 10 | GGPN | H12' | 26.4051198241 | 9.7721958090 | 58.1769814797 |
| 10 | GGPN | H12'' | 27.7359364369 | 8.7411540780 | 57.4979426540 |
| 10 | GGPN | O13' | 25.8529504623 | 8.4856763236 | 56.6236230286 |
| 10 | GGPN | H13' | 25.5906502689 | 7.8036930608 | 57.2555507222 |
| 10 | GGPN | C3' | 29.5524714307 | 17.8577603888 | 49.8409227152 |
| 10 | GGPN | O3' | 29.0980102951 | 18.7941023821 | 49.1631230724 |
| 10 | GGPN | C4' | 29.8649255300 | 16.5198394309 | 49.1954806461 |
| 10 | GGPN | H4' | 29.3230786858 | 15.7073504529 | 49.7129308583 |
| 10 | GGPN | H4'' | 30.9567850371 | 16.3288484060 | 49.2728365235 |
| 10 | GGPN | N9 | 29.4584935423 | 16.5419544021 | 47.8035396555 |
| 10 | GGPN | C4 | 30.3023188450 | 16.7038408734 | 46.7486296492 |
| 10 | GGPN | N2 | 33.4904606166 | 17.1523630880 | 45.4783212990 |
| 10 | GGPN | H21 | 34.0278347162 | 17.3065370944 | 46.2957874253 |
| 10 | GGPN | H22 | 33.9003303110 | 17.2714947557 | 44.5694857398 |
| 10 | GGPN | N3 | 31.6376951571 | 16.8787078627 | 46.8227754796 |
| 10 | GGPN | C2 | 32.1962047208 | 16.9177599002 | 45.6174959792 |
| 10 | GGPN | N1 | 31.4758104498 | 16.8185763694 | 44.4564231149 |
| 10 | GGPN | H1 | 31.9847387384 | 16.7995213321 | 43.5870549430 |
| 10 | GGPN | C6 | 30.0999050118 | 16.6594342827 | 44.3568301685 |

| | | | | | |
|---|---|---|---|---|---|
| 10 | GGPN | O6 | 29.5333094233 | 16.5927142212 | 43.2633056300 |
| 10 | GGPN | C5 | 29.4949878669 | 16.6365120088 | 45.6331643512 |
| 10 | GGPN | N7 | 28.1618864026 | 16.4990082232 | 45.9892988271 |
| 10 | GGPN | C8 | 28.1844586921 | 16.4528504757 | 47.2938782195 |
| 10 | GGPN | H8 | 27.3097069149 | 16.3701993729 | 47.9416278371 |
| 11 | GCPN | C | 32.0555294571 | 25.0489696912 | 49.3938346048 |
| 11 | GCPN | O1' | 31.7919026116 | 26.1725026326 | 49.8033486544 |
| 11 | GCPN | C2' | 31.3773775311 | 23.8271990109 | 49.9154586066 |
| 11 | GCPN | H2' | 30.3210291887 | 23.9134232945 | 49.7163977367 |
| 11 | GCPN | H2'' | 31.5881080129 | 23.8084549478 | 50.9722255760 |
| 11 | GCPN | N2' | 31.8773153771 | 22.5794521286 | 49.3232595588 |
| 11 | GCPN | C5' | 32.8755227209 | 21.8393803392 | 50.0868260911 |
| 11 | GCPN | H5' | 33.6256942191 | 22.5771930138 | 50.3421393252 |
| 11 | GCPN | H5'' | 33.2926744795 | 21.0501269558 | 49.4845716188 |
| 11 | GCPN | N | 31.2549648011 | 20.3076271734 | 51.1560274993 |
| 11 | GCPN | H1' | 31.4084508196 | 19.4890169530 | 50.6162965112 |
| 11 | GCPN | C6' | 32.3673899759 | 21.1927613029 | 51.4003213457 |
| 11 | GCPN | H6' | 32.0414182897 | 21.9931073901 | 52.0512728563 |
| 11 | GCPN | C6'' | 33.5038535116 | 20.4714705366 | 52.1770221021 |
| 11 | GCPN | H6'' | 33.9958316257 | 21.1396435390 | 52.9167694646 |
| 11 | GCPN | H6''' | 34.2861680258 | 20.1805785772 | 51.4436789606 |
| 11 | GCPN | O7' | 32.9557893590 | 19.1668391428 | 52.8380919140 |
| 11 | GCPN | C8' | 32.9511981644 | 19.1958706473 | 54.2673315241 |
| 11 | GCPN | H8' | 33.4429899343 | 20.1230923505 | 54.6343124969 |
| 11 | GCPN | H8'' | 33.5446743740 | 18.3283096145 | 54.6351194864 |
| 11 | GCPN | C9' | 31.5553469946 | 19.0635297969 | 54.9363756024 |
| 11 | GCPN | H9' | 30.7851271391 | 19.5170289794 | 54.2860957707 |
| 11 | GCPN | H9'' | 31.5712420476 | 19.6715514499 | 55.8685873924 |
| 11 | GCPN | O10' | 31.2006964319 | 17.7073168155 | 55.2328663795 |
| 11 | GCPN | C11' | 30.1919255021 | 17.5982898742 | 56.2462293334 |
| 11 | GCPN | H11' | 29.2513438354 | 17.1721110476 | 55.8216404127 |
| 11 | GCPN | H11'' | 29.9505525949 | 18.5868310313 | 56.6946350615 |
| 11 | GCPN | C12' | 30.6588910490 | 16.6546650335 | 57.3617398287 |
| 11 | GCPN | H12' | 31.1997109597 | 17.2532496718 | 58.1283976350 |
| 11 | GCPN | H12'' | 31.3643883113 | 15.9026787870 | 56.9407641004 |
| 11 | GCPN | O13' | 29.5251894715 | 16.0130593335 | 57.9384739579 |
| 11 | GCPN | H13' | 29.5697926381 | 15.0734682631 | 57.7401784875 |
| 11 | GCPN | C3' | 31.2652744231 | 22.1165515377 | 48.1865555734 |
| 11 | GCPN | O3' | 30.4983127667 | 22.8351667470 | 47.5520264796 |
| 11 | GCPN | C4' | 31.5303458198 | 20.6833987803 | 47.6721765222 |
| 11 | GCPN | H4' | 31.1454622130 | 19.9838379465 | 48.4378999866 |
| 11 | GCPN | H4'' | 32.6224548077 | 20.5113886648 | 47.6021737325 |
| 11 | GCPN | N1 | 30.8585296844 | 20.4086814918 | 46.3894454968 |
| 11 | GCPN | C6 | 29.5079278102 | 20.1744194228 | 46.3312090929 |
| 11 | GCPN | H6 | 28.9851473825 | 20.1509181767 | 47.2919314340 |
| 11 | GCPN | C5 | 28.8875739972 | 19.9734953047 | 45.1615395088 |
| 11 | GCPN | H5 | 27.8174745261 | 19.7867402095 | 45.0973624417 |
| 11 | GCPN | C2 | 31.5986368699 | 20.5164511801 | 45.2062878288 |
| 11 | GCPN | O2 | 32.7973148222 | 20.8470749989 | 45.2610096249 |
| 11 | GCPN | N3 | 30.9963345465 | 20.2704039286 | 44.0232521247 |
| 11 | GCPN | C4 | 29.6746962553 | 20.0086299284 | 43.9731276878 |
| 11 | GCPN | N4 | 29.0982517766 | 19.7772313102 | 42.7924395337 |
| 11 | GCPN | H41 | 29.6756778294 | 19.6928380648 | 41.9742808079 |
| 11 | GCPN | H42 | 28.1773168079 | 19.4045519020 | 42.7455054201 |
| 12 | GAPN | C | 32.9174151061 | 28.9653802699 | 45.4576024128 |
| 12 | GAPN | O1' | 33.0461096255 | 30.1313060147 | 45.8080547431 |
| 12 | GAPN | C2' | 32.4270160828 | 27.9153843590 | 46.3792008326 |
| 12 | GAPN | H2' | 31.3593028973 | 28.0086258491 | 46.5100387342 |
| 12 | GAPN | H2'' | 32.9607184710 | 28.1381609314 | 47.2815385139 |
| 12 | GAPN | N2' | 32.7735515725 | 26.5447699442 | 45.9637994935 |
| 12 | GAPN | C5' | 33.9762200989 | 25.9364062088 | 46.5359778884 |
| 12 | GAPN | H5' | 34.7852289497 | 26.6062518286 | 46.3271380953 |
| 12 | GAPN | H5'' | 34.1682710327 | 24.9646461698 | 46.1114471838 |
| 12 | GAPN | N | 32.9861374065 | 24.8249456508 | 48.4602624832 |
| 12 | GAPN | H1' | 33.0163710676 | 23.9198008363 | 48.0560310912 |
| 12 | GAPN | C6' | 33.9559468414 | 25.7953227629 | 48.0509791384 |
| 12 | GAPN | H6' | 33.6511893465 | 26.7538628095 | 48.4420821754 |
| 12 | GAPN | C6'' | 35.3692891140 | 25.5848978474 | 48.6401929176 |
| 12 | GAPN | H6'' | 36.1712811287 | 25.7052539327 | 47.8800797200 |
| 12 | GAPN | H6''' | 35.4521887042 | 24.5618710884 | 49.0680242526 |
| 12 | GAPN | O7' | 35.5670206757 | 26.6599354942 | 49.7118831778 |
| 12 | GAPN | C8' | 35.9541030290 | 26.1355997495 | 50.9663619524 |
| 12 | GAPN | H8' | 37.0311145435 | 25.8627270450 | 50.9361516377 |

| | | | | | |
|---|---|---|---|---|---|
| 12 | GAPN | H8'' | 35.3491309693 | 25.2422586531 | 51.2356148130 |
| 12 | GAPN | C9' | 35.7128877867 | 27.2419990654 | 51.9932319993 |
| 12 | GAPN | H9' | 34.6169347664 | 27.3849980169 | 52.1289954437 |
| 12 | GAPN | H9'' | 36.1387127478 | 28.1923272010 | 51.5988276087 |
| 12 | GAPN | O10' | 36.3185652529 | 26.9406987622 | 53.2365535865 |
| 12 | GAPN | C11' | 36.1627304220 | 28.0321455013 | 54.1329306698 |
| 12 | GAPN | H11' | 35.0814193723 | 28.2128241969 | 54.3250819206 |
| 12 | GAPN | H11'' | 36.5967260910 | 28.9557761935 | 53.6870161651 |
| 12 | GAPN | C12' | 36.8780457166 | 27.7259691213 | 55.4496128147 |
| 12 | GAPN | H12' | 37.9592625960 | 27.5566767471 | 55.2427757883 |
| 12 | GAPN | H12'' | 36.4450734930 | 26.8000553231 | 55.8908651330 |
| 12 | GAPN | O13' | 36.7093084838 | 28.8247244899 | 56.3360999227 |
| 12 | GAPN | H13' | 37.1683395109 | 28.6103854313 | 57.1538574657 |
| 12 | GAPN | C3' | 31.8933893112 | 25.8833651733 | 45.1583773490 |
| 12 | GAPN | O3' | 31.0032485181 | 26.4899902370 | 44.5588252013 |
| 12 | GAPN | C4' | 32.0191910831 | 24.3696644642 | 44.9637169502 |
| 12 | GAPN | H4' | 31.9190819400 | 23.8656043220 | 45.9405873154 |
| 12 | GAPN | H4'' | 33.0201631398 | 24.1456638555 | 44.5407890819 |
| 12 | GAPN | N9 | 31.0001045210 | 23.9048382935 | 44.0277036436 |
| 12 | GAPN | C5 | 29.9740432458 | 23.3737990141 | 42.1697210807 |
| 12 | GAPN | N7 | 29.0577960150 | 23.1605627354 | 43.1907629253 |
| 12 | GAPN | C8 | 29.7104833489 | 23.4946121745 | 44.2770010584 |
| 12 | GAPN | H8 | 29.3117312000 | 23.4507274749 | 45.2939938872 |
| 12 | GAPN | N1 | 31.0106116499 | 23.5147442146 | 40.0603138087 |
| 12 | GAPN | C2 | 32.0789200498 | 24.0120118377 | 40.6835401080 |
| 12 | GAPN | H2 | 32.9268211199 | 24.2630682221 | 40.0367779729 |
| 12 | GAPN | N3 | 32.2661608576 | 24.2051767954 | 41.9899489810 |
| 12 | GAPN | C4 | 31.1645968366 | 23.8540514069 | 42.6746582073 |
| 12 | GAPN | C6 | 29.9124854653 | 23.1785901507 | 40.7785676376 |
| 12 | GAPN | N6 | 28.8369903155 | 22.6707099729 | 40.1509860944 |
| 12 | GAPN | H61 | 28.0234800585 | 22.4022676888 | 40.6573198833 |
| 12 | GAPN | H62 | 28.8778456121 | 22.4594839966 | 39.1678580498 |
| 13 | GTPN | C | 33.4381590903 | 32.0294952719 | 40.7589865351 |
| 13 | GTPN | O1' | 33.2762495930 | 33.2424307312 | 40.7896317720 |
| 13 | GTPN | C2' | 33.0803018946 | 31.1724108885 | 41.9040510132 |
| 13 | GTPN | H2' | 32.1020424238 | 31.4578015591 | 42.2600329346 |
| 13 | GTPN | H2'' | 33.8510313823 | 31.4186794389 | 42.6066799947 |
| 13 | GTPN | N2' | 33.1283421518 | 29.7285627669 | 41.6268024004 |
| 13 | GTPN | C5' | 34.3204672319 | 29.0362676905 | 42.0866038275 |
| 13 | GTPN | H5' | 35.1440644723 | 29.5888693043 | 41.6674247626 |
| 13 | GTPN | H5'' | 34.3369634603 | 28.0035832025 | 41.7743515652 |
| 13 | GTPN | N | 33.2761225874 | 28.5374468609 | 44.2498392570 |
| 13 | GTPN | H1' | 32.8834860262 | 27.6838648485 | 43.9230670897 |
| 13 | GTPN | C6' | 34.4320394143 | 29.0868719048 | 43.6112419235 |
| 13 | GTPN | H6' | 34.4821304385 | 30.1316698703 | 43.8794646306 |
| 13 | GTPN | C6'' | 35.7321250003 | 28.5026133178 | 44.1751794910 |
| 13 | GTPN | H6'' | 36.3684921052 | 28.0183073173 | 43.4131381436 |
| 13 | GTPN | H6''' | 35.4675729276 | 27.7157770343 | 44.8955724057 |
| 13 | GTPN | O7' | 36.4603375815 | 29.6396498453 | 44.9012618066 |
| 13 | GTPN | C8' | 37.2302761055 | 29.1923703479 | 45.9996178820 |
| 13 | GTPN | H8' | 38.0805116305 | 28.5813718751 | 45.6339380377 |
| 13 | GTPN | H8'' | 36.6169325117 | 28.5840539420 | 46.7004859400 |
| 13 | GTPN | C9' | 37.7541854266 | 30.4349123991 | 46.7172537898 |
| 13 | GTPN | H9' | 36.9056338110 | 30.9682855236 | 47.2033425284 |
| 13 | GTPN | H9'' | 38.2141871431 | 31.1182742729 | 45.9684884768 |
| 13 | GTPN | O10' | 38.7220799956 | 30.0773059042 | 47.6851407139 |
| 13 | GTPN | C11' | 39.2047798783 | 31.2320120389 | 48.3566725820 |
| 13 | GTPN | H11' | 38.3557791103 | 31.7920867510 | 48.8100348908 |
| 13 | GTPN | H11'' | 39.7245881988 | 31.9042111386 | 47.6377885621 |
| 13 | GTPN | C12' | 40.1744463596 | 30.7978047004 | 49.4562908934 |
| 13 | GTPN | H12' | 41.0350905482 | 30.2642330737 | 48.9931401138 |
| 13 | GTPN | H12'' | 39.6450088697 | 30.0998810402 | 50.1443929134 |
| 13 | GTPN | O13' | 40.6247278870 | 31.9439680936 | 50.1656298752 |
| 13 | GTPN | H13' | 41.1744909135 | 31.6346878247 | 50.8922778046 |
| 13 | GTPN | C3' | 31.9771445819 | 29.0910813831 | 41.2629956349 |
| 13 | GTPN | O3' | 30.9468613480 | 29.7218045290 | 41.0323586320 |
| 13 | GTPN | C4' | 31.9561426051 | 27.5510878543 | 41.1268983200 |
| 13 | GTPN | H4' | 32.1372651526 | 27.1150018141 | 42.1308933360 |
| 13 | GTPN | H4'' | 32.8041032792 | 27.2470716956 | 40.4780717005 |
| 13 | GTPN | N1 | 30.6893837981 | 27.0205997083 | 40.5959625987 |
| 13 | GTPN | C6 | 29.6197499263 | 26.7762015361 | 41.4234913306 |
| 13 | GTPN | H6 | 29.7939142645 | 27.0205033661 | 42.4766453352 |
| 13 | GTPN | C2 | 30.5874428858 | 26.8860833149 | 39.2192340591 |

| | | | | | |
|---|---|---|---|---|---|
| 13 | GTPN | O2 | 31.4826952873 | 27.1870787665 | 38.4521418502 |
| 13 | GTPN | N3 | 29.3806863910 | 26.3902998931 | 38.8101507869 |
| 13 | GTPN | H3 | 29.2954120909 | 26.2898132401 | 37.8014280381 |
| 13 | GTPN | C4 | 28.2865362851 | 26.0626748466 | 39.5749705306 |
| 13 | GTPN | O4 | 27.2845499485 | 25.5868944730 | 39.0448650745 |
| 13 | GTPN | C5 | 28.4496752399 | 26.2971008349 | 40.9902821494 |
| 13 | GTPN | C5M | 27.3061294014 | 25.9757113664 | 41.8952500323 |
| 13 | GTPN | H51 | 27.5573402139 | 26.1718223495 | 42.9610125398 |
| 13 | GTPN | H52 | 27.0320767742 | 24.9001079090 | 41.8056984096 |
| 13 | GTPN | H53 | 26.4102347123 | 26.5811611911 | 41.6322902029 |
| 14 | GGPN | C | 31.5555746295 | 34.6497994692 | 36.5910285376 |
| 14 | GGPN | O1' | 31.7009847504 | 35.8174169556 | 36.2580883323 |
| 14 | GGPN | C2' | 32.1265085754 | 34.0964555739 | 37.8657888707 |
| 14 | GGPN | H2' | 31.4744279149 | 34.3436458272 | 38.6897583906 |
| 14 | GGPN | H2'' | 33.0988670428 | 34.5348082253 | 37.9883292909 |
| 14 | GGPN | N2' | 32.2866817920 | 32.6303776992 | 37.7686478412 |
| 14 | GGPN | C5' | 33.6018880085 | 32.0793762760 | 37.4276553800 |
| 14 | GGPN | H5' | 34.0361983344 | 32.7125043386 | 36.6773938567 |
| 14 | GGPN | H5'' | 33.5166462920 | 31.0706607851 | 37.0637229076 |
| 14 | GGPN | N | 33.9930160705 | 31.3903247197 | 39.7216921901 |
| 14 | GGPN | H1' | 34.0718348221 | 30.4002996973 | 39.7462232472 |
| 14 | GGPN | C6' | 34.5787467589 | 32.0581200520 | 38.6004946682 |
| 14 | GGPN | H6' | 34.7494894007 | 33.0907051506 | 38.8681789849 |
| 14 | GGPN | C6'' | 35.9614923816 | 31.4859859430 | 38.1944854921 |
| 14 | GGPN | H6'' | 36.6983650137 | 32.2969848540 | 38.2800575222 |
| 14 | GGPN | H6''' | 35.9568732018 | 31.1511848520 | 37.1368197045 |
| 14 | GGPN | O7' | 36.3711574491 | 30.3193791746 | 39.1225067438 |
| 14 | GGPN | C8' | 37.6549707054 | 30.5273300597 | 39.7165612115 |
| 14 | GGPN | H8' | 37.8261631692 | 31.6032811967 | 39.9483123456 |
| 14 | GGPN | H8'' | 38.4517008926 | 30.1754101133 | 39.0266613332 |
| 14 | GGPN | C9' | 37.7067826146 | 29.7349862222 | 41.0303783308 |
| 14 | GGPN | H9' | 37.1534596055 | 28.7859350638 | 40.8492342730 |
| 14 | GGPN | H9'' | 37.1477593099 | 30.3257533825 | 41.7926726182 |
| 14 | GGPN | O10' | 38.9993781624 | 29.4034672514 | 41.5326640105 |
| 14 | GGPN | C11' | 38.8384546469 | 28.5639143697 | 42.6716180128 |
| 14 | GGPN | H11' | 38.2408495458 | 27.6764416168 | 42.3612026273 |
| 14 | GGPN | H11'' | 38.2701619206 | 29.1346296302 | 43.4310889154 |
| 14 | GGPN | C12' | 40.1062996736 | 28.0235705113 | 43.3580933534 |
| 14 | GGPN | H12' | 40.5813513391 | 28.8301384108 | 43.9600449372 |
| 14 | GGPN | H12'' | 40.8255330521 | 27.6778486097 | 42.5846512983 |
| 14 | GGPN | O13' | 39.7489937503 | 26.9187260248 | 44.1910983232 |
| 14 | GGPN | H13' | 39.7884413986 | 27.2145709391 | 45.1073085311 |
| 14 | GGPN | C3' | 31.2062632909 | 31.8684493195 | 38.0706853628 |
| 14 | GGPN | O3' | 30.1474676381 | 32.3840460006 | 38.4514086039 |
| 14 | GGPN | C4' | 31.2455541705 | 30.3673645958 | 37.8235365349 |
| 14 | GGPN | H4' | 31.7457635240 | 29.8585292016 | 38.6652336681 |
| 14 | GGPN | H4'' | 31.7964414783 | 30.1481352088 | 36.8907722323 |
| 14 | GGPN | N9 | 29.8794775104 | 29.9087845925 | 37.6190902332 |
| 14 | GGPN | C4 | 29.2815944121 | 29.6946041414 | 36.4048643821 |
| 14 | GGPN | N2 | 29.3542011765 | 29.6741960425 | 32.9310343563 |
| 14 | GGPN | H21 | 30.2622362100 | 30.0052417824 | 32.7153631645 |
| 14 | GGPN | H22 | 28.7248159788 | 29.4073528787 | 32.1934665665 |
| 14 | GGPN | N3 | 29.8440237858 | 29.8827852431 | 35.1864398291 |
| 14 | GGPN | C2 | 29.0161660514 | 29.5249253811 | 34.2034096259 |
| 14 | GGPN | N1 | 27.7627613849 | 29.0232160418 | 34.4243216281 |
| 14 | GGPN | H1 | 27.2227723512 | 28.7191223669 | 33.6286825849 |
| 14 | GGPN | C6 | 27.1680329617 | 28.8207771705 | 35.6620872516 |
| 14 | GGPN | O6 | 26.0282675137 | 28.3629161713 | 35.7538362043 |
| 14 | GGPN | C5 | 28.0101366420 | 29.2530308659 | 36.7109573900 |
| 14 | GGPN | N7 | 27.7860567906 | 29.2804871466 | 38.0781975093 |
| 14 | GGPN | C8 | 28.9232288670 | 29.6867898750 | 38.5812803693 |
| 14 | GGPN | H8 | 29.1293899987 | 29.8590040341 | 39.6389033068 |
| 15 | GCPN | C | 27.6496869483 | 36.4692578141 | 32.3515731835 |
| 15 | GCPN | O1' | 27.6336611231 | 37.5226877815 | 31.7236253555 |
| 15 | GCPN | C2' | 28.3710752059 | 36.3941249593 | 33.6653743537 |
| 15 | GCPN | H2' | 27.7836439833 | 36.9249563637 | 34.3936530641 |
| 15 | GCPN | H2'' | 29.2913844675 | 36.9229104228 | 33.4840363509 |
| 15 | GCPN | N2' | 28.7028408535 | 35.0603370112 | 34.1871685736 |
| 15 | GCPN | C5' | 29.9299891761 | 34.5082969703 | 33.6151937074 |
| 15 | GCPN | H5' | 30.5211837817 | 35.3667755236 | 33.3483045878 |
| 15 | GCPN | H5'' | 29.5970987018 | 33.9379992540 | 32.7553011382 |
| 15 | GCPN | N | 30.9173201987 | 33.7339603425 | 35.8553768353 |
| 15 | GCPN | H1' | 30.6237140728 | 32.9399690107 | 36.3783559698 |

| | | | | | |
|---|---|---|---|---|---|
| 15 | GCPN | C6' | 30.9100743274 | 33.6024836002 | 34.4189938681 |
| 15 | GCPN | H6' | 30.5615879780 | 32.5879078513 | 34.2877044077 |
| 15 | GCPN | C6'' | 32.3217622312 | 33.5419765829 | 33.7519779544 |
| 15 | GCPN | H6'' | 32.2758316711 | 33.6149823630 | 32.6471685148 |
| 15 | GCPN | H6''' | 32.7004769640 | 32.5318411109 | 33.9928800039 |
| 15 | GCPN | O7' | 33.2869883615 | 34.6134853131 | 34.3165460515 |
| 15 | GCPN | C8' | 34.6508603184 | 34.2068969780 | 34.3990761620 |
| 15 | GCPN | H8' | 35.1690437804 | 34.4351348110 | 33.4472960060 |
| 15 | GCPN | H8'' | 34.7500623102 | 33.1181660254 | 34.6018346618 |
| 15 | GCPN | C9' | 35.3242341825 | 34.9474559013 | 35.5702235302 |
| 15 | GCPN | H9' | 34.6534644034 | 34.9131488757 | 36.4559928679 |
| 15 | GCPN | H9'' | 35.4586996526 | 36.0154838864 | 35.2943773581 |
| 15 | GCPN | O10' | 36.5686654667 | 34.3544591376 | 35.9245906125 |
| 15 | GCPN | C11' | 36.9973844753 | 34.7884361975 | 37.2127690823 |
| 15 | GCPN | H11' | 36.2219725991 | 34.5104259875 | 37.9621433557 |
| 15 | GCPN | H11'' | 37.0999148353 | 35.8939185618 | 37.2358342111 |
| 15 | GCPN | C12' | 38.3317781479 | 34.1304125543 | 37.6083748381 |
| 15 | GCPN | H12' | 39.1865829111 | 34.6765324533 | 37.1562801103 |
| 15 | GCPN | H12'' | 38.3596524551 | 33.0887542032 | 37.2139655949 |
| 15 | GCPN | O13' | 38.4465782463 | 34.0781482271 | 39.0268280293 |
| 15 | GCPN | H13' | 38.6476032410 | 34.9658831297 | 39.3423818665 |
| 15 | GCPN | C3' | 27.8587443404 | 34.4668636976 | 35.0849809489 |
| 15 | GCPN | O3' | 26.9115476994 | 35.0773813647 | 35.5783911508 |
| 15 | GCPN | C4' | 27.9994337352 | 32.9860713256 | 35.4913928312 |
| 15 | GCPN | H4' | 28.5315599272 | 32.9766504515 | 36.4636684679 |
| 15 | GCPN | H4'' | 28.6040917716 | 32.4425323967 | 34.7457682961 |
| 15 | GCPN | N1 | 26.7194211903 | 32.2891595543 | 35.6362936830 |
| 15 | GCPN | C6 | 26.1608120788 | 32.0322537678 | 36.8629685012 |
| 15 | GCPN | H6 | 26.7290661060 | 32.3718375047 | 37.7333754308 |
| 15 | GCPN | C5 | 25.0040070763 | 31.3630508192 | 36.9662937800 |
| 15 | GCPN | H5 | 24.5519745627 | 31.1081845861 | 37.9219041417 |
| 15 | GCPN | C2 | 26.0703906052 | 31.8956827647 | 34.4631547237 |
| 15 | GCPN | O2 | 26.5889998562 | 32.1426845116 | 33.3586922175 |
| 15 | GCPN | N3 | 24.9043121328 | 31.2341908815 | 34.5626675262 |
| 15 | GCPN | C4 | 24.3733400168 | 30.9430839573 | 35.7616132316 |
| 15 | GCPN | N4 | 23.2365842076 | 30.2542703112 | 35.8190121480 |
| 15 | GCPN | H41 | 22.9201971288 | 29.8593422706 | 34.9436059310 |
| 15 | GCPN | H42 | 22.9936615124 | 29.7405517828 | 36.6320942434 |
| 16 | GCPN | C | 22.3658661015 | 36.7421875982 | 29.5025913888 |
| 16 | GCPN | C2' | 23.7233420826 | 36.9792950020 | 30.1823444185 |
| 16 | GCPN | H2' | 23.6335480292 | 37.8906341283 | 30.7529127472 |
| 16 | GCPN | H2'' | 24.4262949613 | 37.1000683726 | 29.3746161442 |
| 16 | GCPN | N2' | 24.2317515580 | 35.9099816719 | 31.0574043583 |
| 16 | GCPN | C5' | 25.0860128332 | 34.8981396192 | 30.4306703893 |
| 16 | GCPN | H5' | 24.7783719558 | 34.8730880888 | 29.3910120251 |
| 16 | GCPN | H5'' | 24.9010182804 | 33.9571306287 | 30.9222053669 |
| 16 | GCPN | N | 27.0660864351 | 35.3568622776 | 31.8751973364 |
| 16 | GCPN | H1' | 27.0666421297 | 34.5280248430 | 32.4267194231 |
| 16 | GCPN | C6' | 26.6203424953 | 35.2384275640 | 30.5010817100 |
| 16 | GCPN | H6' | 26.7107850361 | 36.2088949585 | 30.0259685917 |
| 16 | GCPN | C6'' | 27.6169521518 | 34.3312889733 | 29.6890609955 |
| 16 | GCPN | H6'' | 27.7015877610 | 34.6593699145 | 28.6296398918 |
| 16 | GCPN | H6''' | 27.2574822832 | 33.2813544320 | 29.6900054038 |
| 16 | GCPN | O7' | 29.0141631116 | 34.3820318965 | 30.4017116456 |
| 16 | GCPN | C8' | 30.1571826597 | 34.3782860605 | 29.5452683496 |
| 16 | GCPN | H8' | 29.8957357171 | 34.6820820652 | 28.5107729324 |
| 16 | GCPN | H8'' | 30.5453237336 | 33.3395174649 | 29.4937546285 |
| 16 | GCPN | C9' | 31.2959101544 | 35.2700428474 | 30.1227607279 |
| 16 | GCPN | H9' | 31.0767117810 | 35.4426880779 | 31.1970037707 |
| 16 | GCPN | H9'' | 31.2990494066 | 36.2654374399 | 29.6230575752 |
| 16 | GCPN | O10' | 32.5836308672 | 34.6590375655 | 30.0386297867 |
| 16 | GCPN | C11' | 33.6124247888 | 35.4696888676 | 30.6194627145 |
| 16 | GCPN | H11' | 33.3420344393 | 35.7114143901 | 31.6735391648 |
| 16 | GCPN | H11'' | 33.7160894661 | 36.4249260950 | 30.0522233101 |
| 16 | GCPN | C12' | 34.9665482848 | 34.7244511942 | 30.6250825753 |
| 16 | GCPN | H12' | 35.2636918166 | 34.4078252162 | 29.6010447655 |
| 16 | GCPN | H12'' | 34.8516175186 | 33.7892536812 | 31.2153845302 |
| 16 | GCPN | O13' | 35.9957866186 | 35.5405097948 | 31.1797101146 |
| 16 | GCPN | H13' | 35.9111971612 | 35.5201926273 | 32.1386928081 |
| 16 | GCPN | C3' | 23.9309093093 | 35.9651487717 | 32.3996741004 |
| 16 | GCPN | O3' | 23.3241377762 | 36.9064270527 | 32.9175181380 |
| 16 | GCPN | C4' | 24.3503773127 | 34.8742952994 | 33.4317193824 |
| 16 | GCPN | H4' | 24.9269648399 | 35.4422197717 | 34.1933031745 |

| | | | | | |
|---|---|---|---|---|---|
| 16 | GCPN | H4'' | 25.0331386499 | 34.1405243402 | 32.9802345125 |
| 16 | GCPN | N1 | 23.2447857155 | 34.1377182316 | 34.0693026627 |
| 16 | GCPN | C6 | 22.9602311664 | 34.2493021828 | 35.4055993416 |
| 16 | GCPN | H6 | 23.5785020560 | 34.9615623812 | 35.9551917351 |
| 16 | GCPN | C5 | 21.9788463253 | 33.5232898617 | 35.9735613509 |
| 16 | GCPN | H5 | 21.7418455674 | 33.5789034953 | 37.0327942342 |
| 16 | GCPN | C2 | 22.5581285282 | 33.2036198606 | 33.2905349325 |
| 16 | GCPN | O2 | 22.9026303288 | 33.0105171088 | 32.1181526844 |
| 16 | GCPN | N3 | 21.5501404728 | 32.5025129460 | 33.8391042038 |
| 16 | GCPN | C4 | 21.2406931371 | 32.6374199770 | 35.1294371533 |
| 16 | GCPN | N4 | 20.2298350768 | 31.9069113404 | 35.6039233449 |
| 16 | GCPN | H41 | 19.8172868829 | 31.2552784065 | 34.9507593048 |
| 16 | GCPN | H42 | 19.9925214073 | 31.8483137381 | 36.5640648991 |
| 16 | GCPN | OT1 | 21.7462332036 | 35.6636597189 | 29.7037753658 |
| 16 | GCPN | OT2 | 21.9450517809 | 37.6578922863 | 28.7421596937 |

Model 10

| | | | | | |
|---|---|---|---|---|---|
| 1 | GGPN | N | 14.9588156687 | 27.9162144712 | 25.2883581299 |
| 1 | GGPN | HT1 | 14.4610004851 | 27.7372873385 | 26.1807422937 |
| 1 | GGPN | HT2 | 14.2956946523 | 27.9442537291 | 24.4745219701 |
| 1 | GGPN | HT3 | 15.3637157644 | 28.8783704703 | 25.3178157783 |
| 1 | GGPN | C6' | 16.0469582804 | 26.9300411606 | 24.9732298430 |
| 1 | GGPN | H6' | 15.7528036748 | 25.9763412404 | 25.4003215963 |
| 1 | GGPN | C | 19.8689923025 | 26.0657019206 | 25.4678156330 |
| 1 | GGPN | O1' | 20.4245451457 | 25.2147604164 | 24.8034047591 |
| 1 | GGPN | C2' | 18.7578093996 | 25.7222806511 | 26.4155624173 |
| 1 | GGPN | H2' | 19.1616484332 | 25.3209712841 | 27.3260265724 |
| 1 | GGPN | H2'' | 18.1657864680 | 24.9662244640 | 25.9427936740 |
| 1 | GGPN | N2' | 17.9303087455 | 26.9266621736 | 26.7298574432 |
| 1 | GGPN | C5' | 17.2075453839 | 27.5876080771 | 25.6859962947 |
| 1 | GGPN | H5' | 17.1169455479 | 28.6722762383 | 25.8199490613 |
| 1 | GGPN | H5'' | 17.9091328570 | 27.6074526515 | 24.9165619705 |
| 1 | GGPN | C6'' | 16.6022589351 | 26.6536931616 | 23.5133143603 |
| 1 | GGPN | H6'' | 17.3964287926 | 25.8779941203 | 23.5351324906 |
| 1 | GGPN | H6''' | 17.1776293317 | 27.4972807922 | 23.1453393614 |
| 1 | GGPN | O7' | 15.6195834156 | 26.3500219741 | 22.4123341760 |
| 1 | GGPN | C8' | 16.1563509275 | 26.8122133574 | 21.1718718260 |
| 1 | GGPN | H8' | 17.2384875708 | 26.6040598967 | 21.0933214068 |
| 1 | GGPN | H8'' | 16.0889910342 | 27.9027278003 | 21.0825754705 |
| 1 | GGPN | C9' | 15.3834577247 | 26.1673815324 | 20.0499326130 |
| 1 | GGPN | H9' | 14.3078593785 | 26.1010851200 | 20.3451257340 |
| 1 | GGPN | H9'' | 15.8139506190 | 25.1476537651 | 19.9194142652 |
| 1 | GGPN | O10' | 15.4717224248 | 26.8846884031 | 18.8377837806 |
| 1 | GGPN | C11' | 14.7216140721 | 26.1599815555 | 17.8850853960 |
| 1 | GGPN | H11' | 13.6661464270 | 26.1035352385 | 18.2297290855 |
| 1 | GGPN | H11'' | 15.1301275406 | 25.1254710611 | 17.8484432373 |
| 1 | GGPN | C12' | 14.7709964049 | 26.7441571391 | 16.4775211703 |
| 1 | GGPN | H12' | 15.8361850048 | 26.8840234099 | 16.1860013018 |
| 1 | GGPN | H12'' | 14.2405661102 | 27.7221668331 | 16.4459679504 |
| 1 | GGPN | O13' | 14.1399927866 | 25.8012166252 | 15.6198050300 |
| 1 | GGPN | H13' | 14.2355334454 | 26.1144172039 | 14.7163354150 |
| 1 | GGPN | C3' | 18.3073076450 | 27.6202921963 | 27.8097203261 |
| 1 | GGPN | O3' | 19.3114641183 | 27.2988118636 | 28.4208087151 |
| 1 | GGPN | C4' | 17.4960440490 | 28.8381127267 | 28.2451578850 |
| 1 | GGPN | H4' | 16.4279813316 | 28.7357147142 | 28.0013718804 |
| 1 | GGPN | H4'' | 17.9284504541 | 29.7464234889 | 27.7633108138 |
| 1 | GGPN | N9 | 17.6282428195 | 28.9082156108 | 29.6861840523 |
| 1 | GGPN | C4 | 18.5289554185 | 29.6980253323 | 30.3233542840 |
| 1 | GGPN | N2 | 21.0862340890 | 32.0411143886 | 30.2336452883 |
| 1 | GGPN | H21 | 21.1376484253 | 32.3201785987 | 29.2831755233 |
| 1 | GGPN | H22 | 21.5770231855 | 32.6118456814 | 30.9024477847 |
| 1 | GGPN | N3 | 19.4046972428 | 30.5308746074 | 29.7264971326 |
| 1 | GGPN | C2 | 20.1766228308 | 31.1550673013 | 30.6154324141 |
| 1 | GGPN | N1 | 20.0869511199 | 30.9195155514 | 31.9648683900 |
| 1 | GGPN | H1 | 20.6907748138 | 31.4415605360 | 32.5814552262 |
| 1 | GGPN | C6 | 19.1950375807 | 30.0526017773 | 32.5943597222 |
| 1 | GGPN | O6 | 19.1617117383 | 29.8931020472 | 33.8192589462 |
| 1 | GGPN | C5 | 18.3444543629 | 29.4307805468 | 31.6624466574 |
| 1 | GGPN | N7 | 17.3270362877 | 28.5047909777 | 31.8559510792 |
| 1 | GGPN | C8 | 16.9406548561 | 28.2066652178 | 30.6543743532 |
| 1 | GGPN | H8 | 16.1603139169 | 27.4780986491 | 30.4304704274 |
| 2 | GGPN | C | 25.1886815188 | 26.5658393073 | 24.7897968044 |
| 2 | GGPN | O1' | 25.8134975013 | 25.6546019735 | 24.2588357824 |
| 2 | GGPN | C2' | 23.6711314905 | 26.5303980717 | 24.8099889350 |
| 2 | GGPN | H2' | 23.3846417654 | 25.6041790477 | 25.2893057023 |
| 2 | GGPN | H2'' | 23.3965334268 | 26.5209851451 | 23.7680054620 |
| 2 | GGPN | N2' | 22.9214589125 | 27.6167205987 | 25.4657387189 |
| 2 | GGPN | C5' | 22.0690178253 | 28.5407673205 | 24.6719868747 |
| 2 | GGPN | H5' | 22.5904572845 | 28.6501748100 | 23.7471441509 |
| 2 | GGPN | H5'' | 21.9879684676 | 29.4568571769 | 25.2371440196 |
| 2 | GGPN | N | 20.1212668855 | 27.3860136423 | 25.3647308379 |
| 2 | GGPN | H1' | 19.8596933190 | 27.9533898065 | 26.1158497036 |
| 2 | GGPN | C6' | 20.6409089086 | 28.0600117026 | 24.2147451572 |
| 2 | GGPN | H6' | 20.8024263424 | 27.2825347752 | 23.4852785289 |
| 2 | GGPN | C6'' | 19.7026304750 | 29.1414566858 | 23.3793943807 |
| 2 | GGPN | H6'' | 19.7936662589 | 28.7760791156 | 22.3319038942 |
| 2 | GGPN | H6''' | 20.1901501915 | 30.1355590727 | 23.3814196709 |
| 2 | GGPN | O7' | 18.1307108641 | 29.3971241533 | 23.7001574440 |

| | | | | | |
|---|---|---|---|---|---|
| 2 | GGPN | C8' | 17.3576307017 | 29.9494564676 | 22.5752738962 |
| 2 | GGPN | H8' | 17.4704398965 | 29.2975301620 | 21.6719852537 |
| 2 | GGPN | H8'' | 17.7858225502 | 30.9310738815 | 22.3061221637 |
| 2 | GGPN | C9' | 15.8131136479 | 30.3763023597 | 22.5826595649 |
| 2 | GGPN | H9' | 15.3780816511 | 30.5840615143 | 23.5861440524 |
| 2 | GGPN | H9'' | 15.8241449477 | 31.3594624368 | 22.0099433960 |
| 2 | GGPN | O10' | 14.9482229590 | 29.5159435650 | 21.8098497110 |
| 2 | GGPN | C11' | 13.8188125248 | 28.7939840454 | 22.4187092197 |
| 2 | GGPN | H11' | 13.7170781686 | 27.7581000447 | 22.0027007112 |
| 2 | GGPN | H11'' | 14.1628686130 | 28.1130179090 | 23.1738126985 |
| 2 | GGPN | C12' | 12.4392238124 | 28.8940191390 | 21.6785338801 |
| 2 | GGPN | H12' | 12.5513769566 | 28.4902135704 | 20.6402594996 |
| 2 | GGPN | H12'' | 12.1368362491 | 29.9331490797 | 21.5379310113 |
| 2 | GGPN | O13' | 11.3573862360 | 28.1681206636 | 22.2720517701 |
| 2 | GGPN | H13' | 10.6790684636 | 28.0579114444 | 21.5893599752 |
| 2 | GGPN | C3' | 23.0015008897 | 27.6219086626 | 26.8356006083 |
| 2 | GGPN | O3' | 23.6596514366 | 26.7726584393 | 27.4445806014 |
| 2 | GGPN | C4' | 22.2973056029 | 28.6959242120 | 27.6637842583 |
| 2 | GGPN | H4' | 21.2417971106 | 28.7952833804 | 27.3544855127 |
| 2 | GGPN | H4'' | 22.8069329460 | 29.6611913565 | 27.4501542366 |
| 2 | GGPN | N9 | 22.3287321876 | 28.4143725395 | 29.1019315305 |
| 2 | GGPN | C4 | 23.0283078898 | 29.1283981612 | 30.0323723325 |
| 2 | GGPN | N2 | 25.3237863448 | 31.5645111788 | 30.8643386837 |
| 2 | GGPN | H21 | 25.7005343403 | 31.7833436554 | 29.9755125066 |
| 2 | GGPN | H22 | 25.7806935356 | 31.8673205297 | 31.7070862005 |
| 2 | GGPN | N3 | 23.8575033766 | 30.1586341099 | 29.7807991013 |
| 2 | GGPN | C2 | 24.3491481922 | 30.6750535337 | 30.9037686715 |
| 2 | GGPN | N1 | 23.9742996743 | 30.2505398766 | 32.1524305867 |
| 2 | GGPN | H1 | 24.3377473966 | 30.7445667527 | 32.9512272484 |
| 2 | GGPN | C6 | 23.0944502775 | 29.2201612080 | 32.4361189318 |
| 2 | GGPN | O6 | 22.8116746905 | 28.8992215873 | 33.5943191174 |
| 2 | GGPN | C5 | 22.6764753170 | 28.5764997755 | 31.2525294148 |
| 2 | GGPN | N7 | 21.8928414374 | 27.4558016149 | 31.0856870198 |
| 2 | GGPN | C8 | 21.7179024826 | 27.3843273788 | 29.7947661528 |
| 2 | GGPN | H8 | 21.1727796814 | 26.5880362239 | 29.2926678223 |
| 3 | GCPN | C | 30.3048576013 | 25.5415852409 | 26.6197915674 |
| 3 | GCPN | O1' | 30.9811512412 | 24.6048934176 | 26.2130834501 |
| 3 | GCPN | C2' | 28.8857374508 | 25.7411341628 | 26.2324097238 |
| 3 | GCPN | H2' | 28.3162608712 | 24.8687867610 | 26.5115747676 |
| 3 | GCPN | H2'' | 28.9098982710 | 25.8862625096 | 25.1637939656 |
| 3 | GCPN | N2' | 28.2878980486 | 26.9269349178 | 26.8703828510 |
| 3 | GCPN | C5' | 28.1843719957 | 28.1219936619 | 26.0464180441 |
| 3 | GCPN | H5' | 29.1734096250 | 28.2488811216 | 25.6281485461 |
| 3 | GCPN | H5'' | 27.9069771703 | 28.9682831689 | 26.6511675314 |
| 3 | GCPN | N | 25.8424330711 | 27.6209730915 | 25.3273805861 |
| 3 | GCPN | H1' | 25.3394727271 | 28.2587774812 | 25.8987620743 |
| 3 | GCPN | C6' | 27.1781142522 | 27.9886502572 | 24.8797703946 |
| 3 | GCPN | H6' | 27.5519607600 | 27.1939047998 | 24.2490606514 |
| 3 | GCPN | C6'' | 27.1321132360 | 29.2067670246 | 23.9187321071 |
| 3 | GCPN | H6'' | 28.0396337812 | 29.3180493541 | 23.2892645428 |
| 3 | GCPN | H6''' | 26.9933764287 | 30.1316941103 | 24.5112937507 |
| 3 | GCPN | O7' | 25.8896709364 | 28.9536558628 | 23.0571074747 |
| 3 | GCPN | C8' | 25.6019508381 | 29.8569234177 | 22.0016202027 |
| 3 | GCPN | H8' | 26.2835729639 | 29.7024035798 | 21.1416834820 |
| 3 | GCPN | H8'' | 25.6226782654 | 30.9170250739 | 22.3362892596 |
| 3 | GCPN | C9' | 24.1541886911 | 29.4167280258 | 21.7279974938 |
| 3 | GCPN | H9' | 23.6262822192 | 29.8402176556 | 22.6100770529 |
| 3 | GCPN | H9'' | 24.1078180480 | 28.2985559540 | 21.7577229449 |
| 3 | GCPN | O10' | 23.4485571830 | 29.8389396109 | 20.5732079211 |
| 3 | GCPN | C11' | 22.0996677631 | 29.3780798239 | 20.6859817049 |
| 3 | GCPN | H11' | 21.7113080748 | 29.6892498814 | 21.6769026981 |
| 3 | GCPN | H11'' | 22.0665556397 | 28.2614376297 | 20.6300262605 |
| 3 | GCPN | C12' | 21.1304341036 | 29.9845544199 | 19.6626090657 |
| 3 | GCPN | H12' | 21.5486197033 | 29.8817172250 | 18.6363379327 |
| 3 | GCPN | H12'' | 20.9989304636 | 31.0669014826 | 19.8844853258 |
| 3 | GCPN | O13' | 19.8712636805 | 29.3215813210 | 19.7740162946 |
| 3 | GCPN | H13' | 19.3841593719 | 29.5049801737 | 18.9618067838 |
| 3 | GCPN | C3' | 27.6728850342 | 26.7699838021 | 28.0805005468 |
| 3 | GCPN | O3' | 27.8450014045 | 25.7421388055 | 28.7380908973 |
| 3 | GCPN | C4' | 26.7493523272 | 27.8663570985 | 28.6640025449 |
| 3 | GCPN | H4' | 25.8591806108 | 27.9299698904 | 28.0095632563 |
| 3 | GCPN | H4'' | 27.2596675613 | 28.8500416267 | 28.6189307207 |
| 3 | GCPN | N1 | 26.2920945934 | 27.5494936787 | 30.0272086963 |

| | | | | | |
|---|---|---|---|---|---|
| 3 | GCPN | C6 | 25.3328368103 | 26.5939702182 | 30.2471985263 |
| 3 | GCPN | H6 | 24.8900846581 | 26.1466491441 | 29.3516009725 |
| 3 | GCPN | C5 | 24.9555907514 | 26.2630824138 | 31.4907951957 |
| 3 | GCPN | H5 | 24.1877785886 | 25.5176386574 | 31.6841642495 |
| 3 | GCPN | C2 | 26.9555978214 | 28.1337790991 | 31.1118659487 |
| 3 | GCPN | O2 | 27.9172886299 | 28.8943036932 | 30.9019609568 |
| 3 | GCPN | N3 | 26.5449849465 | 27.8546088128 | 32.3681238039 |
| 3 | GCPN | C4 | 25.5737633717 | 26.9435770483 | 32.5811343526 |
| 3 | GCPN | N4 | 25.1822639430 | 26.6810551832 | 33.8331315936 |
| 3 | GCPN | H41 | 25.5303541181 | 27.2404715808 | 34.5909692272 |
| 3 | GCPN | H42 | 24.3543512087 | 26.1559066274 | 34.0013452789 |
| 4 | GAPN | C | 34.4093984836 | 24.0288704329 | 30.1388138754 |
| 4 | GAPN | O1' | 35.3673949777 | 23.3784782119 | 29.7389830603 |
| 4 | GAPN | C2' | 33.2259989096 | 24.3111024663 | 29.2920812156 |
| 4 | GAPN | H2' | 32.6430595999 | 23.4083579952 | 29.1914747606 |
| 4 | GAPN | H2'' | 33.6754400516 | 24.6091211883 | 28.3637891015 |
| 4 | GAPN | N2' | 32.3714737102 | 25.4201971322 | 29.7691026221 |
| 4 | GAPN | C5' | 32.5049916860 | 26.7350424434 | 29.1250626952 |
| 4 | GAPN | H5' | 33.5390407898 | 27.0227665508 | 29.2003250583 |
| 4 | GAPN | H5'' | 31.8670337737 | 27.4703684138 | 29.5896627817 |
| 4 | GAPN | N | 30.7870732339 | 26.4877618162 | 27.4248468383 |
| 4 | GAPN | H1' | 30.1281074303 | 27.1115025531 | 27.8246318400 |
| 4 | GAPN | C6' | 32.1782049734 | 26.7215904638 | 27.6385976176 |
| 4 | GAPN | H6' | 32.7275766502 | 25.8894406661 | 27.2285701363 |
| 4 | GAPN | C6'' | 32.7076953812 | 27.9480383712 | 26.8689699581 |
| 4 | GAPN | H6'' | 33.3857657747 | 28.5717583424 | 27.4878437391 |
| 4 | GAPN | H6''' | 31.8465897687 | 28.5683698802 | 26.5339902475 |
| 4 | GAPN | O7' | 33.4930934248 | 27.4098442575 | 25.6731729068 |
| 4 | GAPN | C8' | 33.1535632336 | 28.0246285412 | 24.4460222836 |
| 4 | GAPN | H8' | 33.6029364322 | 29.0399962891 | 24.3964788253 |
| 4 | GAPN | H8'' | 32.0511823226 | 28.0913102661 | 24.3109275630 |
| 4 | GAPN | C9' | 33.7468300515 | 27.1251018734 | 23.3644766595 |
| 4 | GAPN | H9' | 33.1822001526 | 26.1654620990 | 23.3367863712 |
| 4 | GAPN | H9'' | 34.8037870362 | 26.8993594558 | 23.6335847543 |
| 4 | GAPN | O10' | 33.7102481373 | 27.7464538570 | 22.0946046574 |
| 4 | GAPN | C11' | 34.3510287504 | 26.9258194731 | 21.1283351119 |
| 4 | GAPN | H11' | 33.8179957092 | 25.9524180743 | 21.0426229120 |
| 4 | GAPN | H11'' | 35.4024497479 | 26.7228388950 | 21.4347119993 |
| 4 | GAPN | C12' | 34.3450436177 | 27.6413957488 | 19.7775973797 |
| 4 | GAPN | H12' | 34.8841172999 | 28.6107478811 | 19.8797731212 |
| 4 | GAPN | H12'' | 33.2911473814 | 27.8443977890 | 19.4808697202 |
| 4 | GAPN | O13' | 34.9841391192 | 26.8174659187 | 18.8114246131 |
| 4 | GAPN | H13' | 34.9660650110 | 27.2884918094 | 17.9729379561 |
| 4 | GAPN | C3' | 31.3979991476 | 25.1184328226 | 30.6773236810 |
| 4 | GAPN | O3' | 31.3977284219 | 24.0488370572 | 31.2908648025 |
| 4 | GAPN | C4' | 30.2951504181 | 26.1365747283 | 30.9749975970 |
| 4 | GAPN | H4' | 29.7194875931 | 26.3308583021 | 30.0550097283 |
| 4 | GAPN | H4'' | 30.7634401515 | 27.0841784430 | 31.3107650194 |
| 4 | GAPN | N9 | 29.4343837138 | 25.6560987249 | 32.0492449956 |
| 4 | GAPN | C5 | 28.6161340390 | 25.2940317947 | 34.0427229974 |
| 4 | GAPN | N7 | 27.7838029244 | 24.6231011733 | 33.1575940025 |
| 4 | GAPN | C8 | 28.3113739975 | 24.8635662830 | 31.9828142558 |
| 4 | GAPN | H8 | 27.9188667545 | 24.5013456301 | 31.0292875023 |
| 4 | GAPN | N1 | 29.5781158554 | 26.1943096100 | 35.9926558644 |
| 4 | GAPN | C2 | 30.5468339031 | 26.6851173574 | 35.2199398981 |
| 4 | GAPN | H2 | 31.3243556839 | 27.2524600418 | 35.7432185594 |
| 4 | GAPN | N3 | 30.6649070640 | 26.6203345156 | 33.8934237716 |
| 4 | GAPN | C4 | 29.6495633174 | 25.9104695827 | 33.3707222864 |
| 4 | GAPN | C6 | 28.5743845180 | 25.4743138460 | 35.4353131167 |
| 4 | GAPN | N6 | 27.5894644380 | 24.9843762561 | 36.2080048606 |
| 4 | GAPN | H61 | 26.8711879209 | 24.4096002701 | 35.8274731181 |
| 4 | GAPN | H62 | 27.5294766683 | 25.2413993573 | 37.1795935468 |
| 5 | GTPN | C | 37.5946167908 | 22.4535909699 | 34.3096666726 |
| 5 | GTPN | O1' | 38.3167770029 | 21.4919985509 | 34.0799922603 |
| 5 | GTPN | C2' | 36.5609580607 | 22.8793324373 | 33.3446631894 |
| 5 | GTPN | H2' | 36.0841167072 | 21.9921284219 | 32.9508158789 |
| 5 | GTPN | H2'' | 37.1646349689 | 23.3615348132 | 32.6033606913 |
| 5 | GTPN | N2' | 35.5453030323 | 23.8394389575 | 33.8107120545 |
| 5 | GTPN | C5' | 35.6440568908 | 25.2168449044 | 33.3314799623 |
| 5 | GTPN | H5' | 36.6092821980 | 25.5602150208 | 33.6402892906 |
| 5 | GTPN | H5'' | 34.8784283023 | 25.8532237439 | 33.7392558404 |
| 5 | GTPN | N | 34.3890409006 | 24.5877979359 | 31.3408549878 |
| 5 | GTPN | H1' | 33.5027527062 | 24.8213931454 | 31.7252832788 |

| | | | | | |
|---|---|---|---|---|---|
| 5 | GTPN | C6' | 35.5556898264 | 25.2639086129 | 31.8056225814 |
| 5 | GTPN | H6' | 36.4354801769 | 24.7395140216 | 31.4583837725 |
| 5 | GTPN | C6'' | 35.6630915754 | 26.6265952743 | 31.1112777018 |
| 5 | GTPN | H6'' | 36.1989178818 | 27.3698548516 | 31.7149481472 |
| 5 | GTPN | H6''' | 34.6405600398 | 27.0170937547 | 30.9391360223 |
| 5 | GTPN | O7' | 36.3861881749 | 26.3185238833 | 29.7777059226 |
| 5 | GTPN | C8' | 36.6238542495 | 27.4199243564 | 28.9295350246 |
| 5 | GTPN | H8' | 36.6442711078 | 28.3606475482 | 29.5078072102 |
| 5 | GTPN | H8'' | 35.8264649367 | 27.4861806097 | 28.1563959189 |
| 5 | GTPN | C9' | 37.9642076544 | 27.1635377117 | 28.2294538676 |
| 5 | GTPN | H9' | 37.9090219779 | 26.1943122366 | 27.6800747448 |
| 5 | GTPN | H9'' | 38.7843934150 | 27.0792832965 | 28.9769457548 |
| 5 | GTPN | O10' | 38.2278690956 | 28.2243450262 | 27.3278168820 |
| 5 | GTPN | C11' | 39.3678532918 | 27.9545097469 | 26.5217610779 |
| 5 | GTPN | H11' | 39.2798426577 | 26.9479545865 | 26.0522154389 |
| 5 | GTPN | H11'' | 40.2913781100 | 27.9752001921 | 27.1417955267 |
| 5 | GTPN | C12' | 39.4572267610 | 29.0157549792 | 25.4209569636 |
| 5 | GTPN | H12' | 39.5992070924 | 30.0171503296 | 25.8868295842 |
| 5 | GTPN | H12'' | 38.5029771859 | 29.0206701817 | 24.8455636606 |
| 5 | GTPN | O13' | 40.5473485624 | 28.7074688959 | 24.5623497910 |
| 5 | GTPN | H13' | 40.5391926229 | 29.3453797845 | 23.8418235195 |
| 5 | GTPN | C3' | 34.3986188661 | 23.3196645282 | 34.3277707377 |
| 5 | GTPN | O3' | 34.2953657713 | 22.1211477953 | 34.5761625632 |
| 5 | GTPN | C4' | 33.2273767504 | 24.2784738607 | 34.5885319281 |
| 5 | GTPN | H4' | 32.8698428607 | 24.6559517424 | 33.6073709975 |
| 5 | GTPN | H4'' | 33.6060152445 | 25.1428143668 | 35.1695630210 |
| 5 | GTPN | N1 | 32.0902229846 | 23.6647090792 | 35.2908433182 |
| 5 | GTPN | C6 | 31.1480264924 | 22.9593496836 | 34.6095272460 |
| 5 | GTPN | H6 | 31.3146201463 | 22.8832123785 | 33.5321286387 |
| 5 | GTPN | C2 | 32.1318368979 | 23.7486669664 | 36.6868528098 |
| 5 | GTPN | O2 | 32.9993514149 | 24.2388646050 | 37.2993195326 |
| 5 | GTPN | N3 | 31.0013637834 | 23.1669310953 | 37.2492102540 |
| 5 | GTPN | H3 | 30.9844312672 | 23.2570518411 | 38.2616704290 |
| 5 | GTPN | C4 | 29.9733472853 | 22.5103987531 | 36.6380195217 |
| 5 | GTPN | O4 | 29.0172075593 | 22.0982243990 | 37.3088735845 |
| 5 | GTPN | C5 | 30.0891497971 | 22.3827956735 | 35.2067816457 |
| 5 | GTPN | C5M | 29.0488457896 | 21.6404919032 | 34.4482992998 |
| 5 | GTPN | H51 | 29.2557879107 | 21.6478569526 | 33.3544706423 |
| 5 | GTPN | H52 | 28.0488908157 | 22.1045622508 | 34.6067048501 |
| 5 | GTPN | H53 | 28.9990319736 | 20.5807367226 | 34.7854538444 |
| 6 | GGPN | C | 39.3116791041 | 19.0394347801 | 37.9228601791 |
| 6 | GGPN | O1' | 40.2157108733 | 18.2223555122 | 37.7901718658 |
| 6 | GGPN | C2' | 38.9025425993 | 19.9860588323 | 36.8243558126 |
| 6 | GGPN | H2' | 38.3660865758 | 19.4296105327 | 36.0695407595 |
| 6 | GGPN | H2'' | 39.8092729631 | 20.4069324136 | 36.4158058690 |
| 6 | GGPN | N2' | 38.0496005860 | 21.0888007546 | 37.3240021758 |
| 6 | GGPN | C5' | 38.6378047212 | 22.4153393272 | 37.5271572059 |
| 6 | GGPN | H5' | 39.5764631237 | 22.2556775844 | 38.0313460943 |
| 6 | GGPN | H5'' | 37.9862006361 | 23.0285509977 | 38.1335822626 |
| 6 | GGPN | N | 37.7496236078 | 23.2051246391 | 35.3999336055 |
| 6 | GGPN | H1' | 37.0839647156 | 23.9196316145 | 35.5925911562 |
| 6 | GGPN | C6' | 38.9334031450 | 23.1610275680 | 36.2119667362 |
| 6 | GGPN | H6' | 39.7026874257 | 22.5940915846 | 35.6923470187 |
| 6 | GGPN | C6'' | 39.5307782194 | 24.5839983295 | 36.4196332199 |
| 6 | GGPN | H6'' | 40.6162146472 | 24.5898608634 | 36.1898804494 |
| 6 | GGPN | H6''' | 39.4197489522 | 24.9073570063 | 37.4737744629 |
| 6 | GGPN | O7' | 38.7554940101 | 25.5833022192 | 35.5183037115 |
| 6 | GGPN | C8' | 39.5979618917 | 26.4307599726 | 34.7421583694 |
| 6 | GGPN | H8' | 40.5202536138 | 25.9058357078 | 34.4126820098 |
| 6 | GGPN | H8'' | 39.8796662623 | 27.3287487951 | 35.3346346754 |
| 6 | GGPN | C9' | 38.8070905504 | 26.8800237200 | 33.5079179648 |
| 6 | GGPN | H9' | 37.8041867697 | 27.1977155541 | 33.8696759378 |
| 6 | GGPN | H9'' | 38.6800217097 | 26.0121729536 | 32.8192237195 |
| 6 | GGPN | O10' | 39.3923634329 | 27.9731114910 | 32.8169372060 |
| 6 | GGPN | C11' | 38.4448846232 | 28.5263442983 | 31.9148463195 |
| 6 | GGPN | H11' | 37.5182786893 | 28.7819170093 | 32.4820676032 |
| 6 | GGPN | H11'' | 38.2095317700 | 27.7684617426 | 31.1392975639 |
| 6 | GGPN | C12' | 38.8925124270 | 29.8145291218 | 31.2205810133 |
| 6 | GGPN | H12' | 39.7470214285 | 29.6138005498 | 30.5381246341 |
| 6 | GGPN | H12'' | 39.2139502713 | 30.5387981559 | 32.0003883646 |
| 6 | GGPN | O13' | 37.7893765309 | 30.3560695418 | 30.4988660282 |
| 6 | GGPN | H13' | 37.7614716693 | 29.9149427380 | 29.6419055347 |
| 6 | GGPN | C3' | 36.7172106563 | 20.8330410381 | 37.4581318809 |

| | | | | | |
|---|---|---|---|---|---|
| 6 | GGPN | O3' | 36.3004469310 | 19.6724843425 | 37.3830882762 |
| 6 | GGPN | C4' | 35.7095390804 | 21.9479413182 | 37.7492674200 |
| 6 | GGPN | H4' | 35.6179971750 | 22.5944573284 | 36.8584726005 |
| 6 | GGPN | H4'' | 36.0528977851 | 22.5540016106 | 38.6104219100 |
| 6 | GGPN | N9 | 34.4152176412 | 21.3511846573 | 38.0602184041 |
| 6 | GGPN | C4 | 33.8750858393 | 21.2218671298 | 39.3102750139 |
| 6 | GGPN | N2 | 34.1386480546 | 21.5090497975 | 42.7500061418 |
| 6 | GGPN | H21 | 35.0233650137 | 21.9422094949 | 42.8626600858 |
| 6 | GGPN | H22 | 33.6228742826 | 21.1963329735 | 43.5546326850 |
| 6 | GGPN | N3 | 34.4499094783 | 21.6084295814 | 40.4685199401 |
| 6 | GGPN | C2 | 33.7058523496 | 21.2842976394 | 41.5214061731 |
| 6 | GGPN | N1 | 32.4817906463 | 20.6744985333 | 41.4198542687 |
| 6 | GGPN | H1 | 31.9691658019 | 20.4858818932 | 42.2669586255 |
| 6 | GGPN | C6 | 31.8551877234 | 20.2966320764 | 40.2404150760 |
| 6 | GGPN | O6 | 30.7490082456 | 19.7489821594 | 40.2585056517 |
| 6 | GGPN | C5 | 32.6652796249 | 20.5816118296 | 39.1149213146 |
| 6 | GGPN | N7 | 32.4754251267 | 20.2718032292 | 37.7776863874 |
| 6 | GGPN | C8 | 33.5433274460 | 20.7416645960 | 37.1861828725 |
| 6 | GGPN | H8 | 33.7734567132 | 20.6459330742 | 36.1238401061 |
| 7 | GCPN | C | 38.5354599342 | 15.2869070726 | 42.9005052368 |
| 7 | GCPN | O1' | 39.2634828216 | 14.3852035568 | 43.2934390946 |
| 7 | GCPN | C2' | 38.8823370393 | 15.9959550694 | 41.6128989745 |
| 7 | GCPN | H2' | 38.7869141396 | 15.2837190797 | 40.8092915158 |
| 7 | GCPN | H2'' | 39.9160518188 | 16.2659493528 | 41.7629287900 |
| 7 | GCPN | N2' | 38.1602478840 | 17.2169977753 | 41.2286678235 |
| 7 | GCPN | C5' | 38.7915752447 | 18.4850615374 | 41.5360328824 |
| 7 | GCPN | H5' | 39.7166111419 | 18.2546037503 | 42.0410835887 |
| 7 | GCPN | H5'' | 38.1050462419 | 18.9964097676 | 42.1854928175 |
| 7 | GCPN | N | 38.6258244591 | 19.2019161845 | 39.0604086292 |
| 7 | GCPN | H1' | 37.7063018495 | 19.5372472931 | 38.8830732298 |
| 7 | GCPN | C6' | 39.1466385069 | 19.4667872433 | 40.3802193464 |
| 7 | GCPN | H6' | 38.4885947639 | 20.2985409738 | 40.5782366144 |
| 7 | GCPN | C6'' | 40.5147673948 | 20.1830555792 | 40.5568685245 |
| 7 | GCPN | H6'' | 41.2674891796 | 19.5513851709 | 41.0692117948 |
| 7 | GCPN | H6''' | 40.3441162645 | 21.1087357212 | 41.1360458984 |
| 7 | GCPN | O7' | 41.0538755011 | 20.5858735528 | 39.2179908294 |
| 7 | GCPN | C8' | 42.0755268432 | 21.5448429037 | 39.2530919471 |
| 7 | GCPN | H8' | 42.8966818974 | 21.2756475565 | 39.9511582568 |
| 7 | GCPN | H8'' | 41.6580303287 | 22.5381174064 | 39.5246807391 |
| 7 | GCPN | C9' | 42.4938395854 | 21.4790129085 | 37.7972795465 |
| 7 | GCPN | H9' | 41.5874470684 | 21.3353840773 | 37.1645909519 |
| 7 | GCPN | H9'' | 43.1452058664 | 20.5932066287 | 37.6238047930 |
| 7 | GCPN | O10' | 43.1227295239 | 22.6636918060 | 37.3805982616 |
| 7 | GCPN | C11' | 43.0466245072 | 22.7500004673 | 35.9720933325 |
| 7 | GCPN | H11' | 41.9967596080 | 22.9430307144 | 35.6612684216 |
| 7 | GCPN | H11'' | 43.3662406067 | 21.7861247948 | 35.5153797780 |
| 7 | GCPN | C12' | 43.9420761089 | 23.8920411802 | 35.5266483580 |
| 7 | GCPN | H12' | 44.9754130614 | 23.7151389859 | 35.9041627788 |
| 7 | GCPN | H12'' | 43.5705404810 | 24.8490573785 | 35.9584716622 |
| 7 | GCPN | O13' | 43.9206084350 | 23.9497946071 | 34.1096388515 |
| 7 | GCPN | H13' | 44.2356697953 | 23.0991501779 | 33.7867414419 |
| 7 | GCPN | C3' | 36.9043280793 | 17.1070119990 | 40.7163953801 |
| 7 | GCPN | O3' | 36.4656359505 | 16.0209925428 | 40.3383392250 |
| 7 | GCPN | C4' | 35.9688147332 | 18.3358327060 | 40.6470730471 |
| 7 | GCPN | H4' | 36.1912174771 | 18.8969018051 | 39.7241064872 |
| 7 | GCPN | H4'' | 36.1458471607 | 19.0128016483 | 41.5056835160 |
| 7 | GCPN | N1 | 34.5694526833 | 17.8993656461 | 40.6218616925 |
| 7 | GCPN | C6 | 33.9315041944 | 17.6256351043 | 39.4406009864 |
| 7 | GCPN | H6 | 34.4860349788 | 17.8708185252 | 38.5285571660 |
| 7 | GCPN | C5 | 32.6762445567 | 17.1570379524 | 39.4294892460 |
| 7 | GCPN | H5 | 32.1359203406 | 16.9624243942 | 38.5066439748 |
| 7 | GCPN | C2 | 33.9605994950 | 17.5896690140 | 41.8429559230 |
| 7 | GCPN | O2 | 34.6047587733 | 17.7154267828 | 42.9009383343 |
| 7 | GCPN | N3 | 32.6831320422 | 17.1611548995 | 41.8422009305 |
| 7 | GCPN | C4 | 32.0379424724 | 16.9358348680 | 40.6847218489 |
| 7 | GCPN | N4 | 30.7792691206 | 16.4985762074 | 40.7143611343 |
| 7 | GCPN | H41 | 30.3197310014 | 16.4911809841 | 41.6126905996 |
| 7 | GCPN | H42 | 30.1888547953 | 16.6311738702 | 39.9271596752 |
| 8 | GCPN | C | 35.6308833941 | 11.1562138571 | 46.1175654468 |
| 8 | GCPN | C2' | 36.6246410349 | 11.9704397872 | 45.2778052562 |
| 8 | GCPN | H2' | 37.1060410912 | 11.2928430782 | 44.5925738521 |
| 8 | GCPN | H2'' | 37.3311933956 | 12.3715553820 | 45.9889004359 |
| 8 | GCPN | N2' | 36.0635262722 | 13.0937094472 | 44.5149680733 |

| | | | | | |
|---|---|---|---|---|---|
| 8 | GCPN | C5' | 36.0055668041 | 14.3633491312 | 45.2169630155 |
| 8 | GCPN | H5' | 35.9448637555 | 14.0939618450 | 46.2580927700 |
| 8 | GCPN | H5'' | 35.1125945221 | 14.8933174045 | 44.9356485089 |
| 8 | GCPN | N | 37.4596914216 | 15.6649332329 | 43.6195288075 |
| 8 | GCPN | H1' | 36.8215133706 | 16.3287723447 | 43.2389237150 |
| 8 | GCPN | C6' | 37.2570059909 | 15.2727360056 | 45.0110324158 |
| 8 | GCPN | H6' | 38.1057679235 | 14.6753310808 | 45.3366867931 |
| 8 | GCPN | C6'' | 37.2488214693 | 16.5159654194 | 45.9574621050 |
| 8 | GCPN | H6'' | 37.8117246184 | 16.3330977139 | 46.8958694332 |
| 8 | GCPN | H6''' | 36.2094487790 | 16.7742107958 | 46.2355806034 |
| 8 | GCPN | O7' | 37.8209192606 | 17.7145482247 | 45.1802841016 |
| 8 | GCPN | C8' | 38.6168769119 | 18.6265323132 | 45.9284863510 |
| 8 | GCPN | H8' | 39.3174686536 | 18.1061487203 | 46.6152595085 |
| 8 | GCPN | H8'' | 37.9863156881 | 19.3506885962 | 46.4877135157 |
| 8 | GCPN | C9' | 39.3699957967 | 19.3184107737 | 44.7899084205 |
| 8 | GCPN | H9' | 38.5829131743 | 19.6145093530 | 44.0618891781 |
| 8 | GCPN | H9'' | 40.0148076821 | 18.5434474421 | 44.3129971085 |
| 8 | GCPN | O10' | 40.1505590871 | 20.4748051273 | 45.0439253989 |
| 8 | GCPN | C11' | 40.6091745140 | 20.9509128116 | 43.7818185776 |
| 8 | GCPN | H11' | 39.7198100453 | 21.1398743236 | 43.1387275731 |
| 8 | GCPN | H11'' | 41.2104329582 | 20.1457125446 | 43.3011125938 |
| 8 | GCPN | C12' | 41.4508798835 | 22.2381779724 | 43.7964938771 |
| 8 | GCPN | H12' | 42.3174799655 | 22.1137797678 | 44.4816341586 |
| 8 | GCPN | H12'' | 40.8253799792 | 23.0821544696 | 44.1619688938 |
| 8 | GCPN | O13' | 41.9060717736 | 22.5003112603 | 42.4684207846 |
| 8 | GCPN | H13' | 42.4825909335 | 23.2736070710 | 42.4922044148 |
| 8 | GCPN | C3' | 35.6718397805 | 12.9168650003 | 43.2168528607 |
| 8 | GCPN | O3' | 35.8526113057 | 11.8677488566 | 42.5896174413 |
| 8 | GCPN | C4' | 34.9887390988 | 14.0506037454 | 42.3976270516 |
| 8 | GCPN | H4' | 35.6314072388 | 14.1391510131 | 41.5011431039 |
| 8 | GCPN | H4'' | 35.0257807750 | 15.0271383360 | 42.9033702535 |
| 8 | GCPN | N1 | 33.6017022536 | 13.7863976322 | 41.9942594316 |
| 8 | GCPN | C6 | 33.2488935312 | 13.5768812069 | 40.6863260223 |
| 8 | GCPN | H6 | 34.0725771250 | 13.5942414129 | 39.9700030592 |
| 8 | GCPN | C5 | 31.9666113427 | 13.3702886169 | 40.3374156309 |
| 8 | GCPN | H5 | 31.6483168737 | 13.2239254906 | 39.3094106180 |
| 8 | GCPN | C2 | 32.6132485947 | 13.8860638403 | 42.9750511983 |
| 8 | GCPN | O2 | 32.9180358892 | 14.2273303140 | 44.1250307497 |
| 8 | GCPN | N3 | 31.3357638346 | 13.6351356253 | 42.6407488611 |
| 8 | GCPN | C4 | 30.9920773474 | 13.3699268280 | 41.3782503937 |
| 8 | GCPN | N4 | 29.7084068842 | 13.1202142074 | 41.1171037360 |
| 8 | GCPN | H41 | 29.0751810469 | 13.1801542948 | 41.9039015578 |
| 8 | GCPN | H42 | 29.3388670415 | 12.9579957335 | 40.2126832496 |
| 8 | GCPN | OT1 | 34.4054895804 | 11.4543334352 | 46.1069817730 |
| 8 | GCPN | OT2 | 36.1167639536 | 10.2213245866 | 46.8109572307 |
| 9 | GGPN | N | 24.2494717912 | 12.4752048540 | 51.1201365188 |
| 9 | GGPN | HT1 | 24.5982610614 | 13.3386953063 | 50.6486311612 |
| 9 | GGPN | HT2 | 23.3049259483 | 12.6424101271 | 51.5245837615 |
| 9 | GGPN | HT3 | 24.1980577913 | 11.7099541667 | 50.4214975765 |
| 9 | GGPN | C6' | 25.1705878501 | 12.1442746322 | 52.2434334150 |
| 9 | GGPN | H6' | 24.8522208602 | 12.7462164979 | 53.0843305583 |
| 9 | GGPN | C | 26.9396124877 | 16.0362786153 | 52.3177459692 |
| 9 | GGPN | O1' | 26.6548420727 | 17.0481711107 | 52.9429008929 |
| 9 | GGPN | C2' | 25.8946238230 | 15.0417082359 | 51.9779830183 |
| 9 | GGPN | H2' | 25.1063480254 | 15.5453620335 | 51.4362985505 |
| 9 | GGPN | H2'' | 25.5651536693 | 14.6859819151 | 52.9440746112 |
| 9 | GGPN | N2' | 26.4235460046 | 13.9117191922 | 51.1958408867 |
| 9 | GGPN | C5' | 26.5581517393 | 12.6375516275 | 51.8720296388 |
| 9 | GGPN | H5' | 27.0796320258 | 11.8749540518 | 51.3196244893 |
| 9 | GGPN | H5'' | 27.1224269613 | 12.8702250134 | 52.7392058888 |
| 9 | GGPN | C6'' | 24.9785640404 | 10.6940738636 | 52.6543877615 |
| 9 | GGPN | H6'' | 25.7869875456 | 10.3232341979 | 53.3028950414 |
| 9 | GGPN | H6''' | 24.9104131242 | 10.0028064767 | 51.7888430253 |
| 9 | GGPN | O7' | 23.6813953752 | 10.7649688669 | 53.4451811676 |
| 9 | GGPN | C8' | 22.7759015336 | 9.7187922831 | 53.1819649463 |
| 9 | GGPN | H8' | 22.8805481242 | 8.9409832235 | 53.9702021079 |
| 9 | GGPN | H8'' | 22.9168764989 | 9.2508601477 | 52.1824824523 |
| 9 | GGPN | C9' | 21.4038613254 | 10.3720025289 | 53.2620649371 |
| 9 | GGPN | H9' | 21.2185158066 | 11.0152989633 | 52.3749875327 |
| 9 | GGPN | H9'' | 21.3456413534 | 11.0053653618 | 54.1778404680 |
| 9 | GGPN | O10' | 20.4346595349 | 9.3557362069 | 53.3275616906 |
| 9 | GGPN | C11' | 19.1485710670 | 9.9019144743 | 53.5560381486 |
| 9 | GGPN | H11' | 18.8308653317 | 10.5133985149 | 52.6827283904 |

| | | | | | |
|---|---|---|---|---|---|
| 9 | GGPN | H11'' | 19.1533913236 | 10.5494730246 | 54.4624211229 |
| 9 | GGPN | C12' | 18.1863402040 | 8.7388458994 | 53.7678832566 |
| 9 | GGPN | H12' | 18.5080641342 | 8.1602217532 | 54.6644008748 |
| 9 | GGPN | H12'' | 18.2191589551 | 8.0694228299 | 52.8782670063 |
| 9 | GGPN | O13' | 16.8802944157 | 9.2632828405 | 53.9515596215 |
| 9 | GGPN | H13' | 16.2905108541 | 8.5173970558 | 54.0975097827 |
| 9 | GGPN | C3' | 26.6446944271 | 14.0842775148 | 49.8722119357 |
| 9 | GGPN | O3' | 26.3629639448 | 15.1415250257 | 49.3318412459 |
| 9 | GGPN | C4' | 27.2101586719 | 12.8811008167 | 49.1684853439 |
| 9 | GGPN | H4' | 26.6329474990 | 11.9645217881 | 49.3712698111 |
| 9 | GGPN | H4'' | 28.2623098522 | 12.7288194081 | 49.5062847901 |
| 9 | GGPN | N9 | 27.1070862690 | 13.0339970912 | 47.7401402650 |
| 9 | GGPN | C4 | 28.1602286309 | 13.2331823663 | 46.9227440714 |
| 9 | GGPN | N2 | 31.5778897148 | 13.5022957817 | 46.4468083726 |
| 9 | GGPN | H21 | 31.9586802042 | 13.4016414952 | 47.3574321619 |
| 9 | GGPN | H22 | 32.2176867515 | 13.4753458886 | 45.6706535131 |
| 9 | GGPN | N3 | 29.4433413033 | 13.3315209559 | 47.3149824581 |
| 9 | GGPN | C2 | 30.2695904693 | 13.4097858176 | 46.2733657610 |
| 9 | GGPN | N1 | 29.8233571118 | 13.3911719131 | 44.9749341626 |
| 9 | GGPN | H1 | 30.5036292398 | 13.4585746653 | 44.2333998372 |
| 9 | GGPN | C6 | 28.4950993972 | 13.3109042257 | 44.5582399126 |
| 9 | GGPN | O6 | 28.1576900658 | 13.2918061958 | 43.3706137432 |
| 9 | GGPN | C5 | 27.6222018086 | 13.2341848359 | 45.6567087246 |
| 9 | GGPN | N7 | 26.2419328153 | 13.0784130788 | 45.6953047949 |
| 9 | GGPN | C8 | 25.9789975413 | 12.9697967569 | 46.9565221441 |
| 9 | GGPN | H8 | 24.9791367453 | 12.8496434811 | 47.3702208338 |
| 10 | GGPN | C | 30.2122877048 | 20.5070473508 | 51.6670498920 |
| 10 | GGPN | O1' | 29.9524134728 | 21.5250683514 | 52.3009178615 |
| 10 | GGPN | C2' | 29.3571993372 | 19.2915883388 | 51.7487531448 |
| 10 | GGPN | H2' | 28.3759595097 | 19.5416156325 | 51.3850983795 |
| 10 | GGPN | H2'' | 29.3482295834 | 19.0650674677 | 52.8017104384 |
| 10 | GGPN | N2' | 29.8443936795 | 18.1067657673 | 51.0255296468 |
| 10 | GGPN | C5' | 30.3728450174 | 17.0255721993 | 51.8304295270 |
| 10 | GGPN | H5' | 31.0420747635 | 17.6200002756 | 52.4371036202 |
| 10 | GGPN | H5'' | 30.9231604079 | 16.3047844802 | 51.2511392556 |
| 10 | GGPN | N | 28.1891147508 | 15.7178852326 | 51.9642847842 |
| 10 | GGPN | H1' | 28.3172491447 | 14.9055106766 | 51.4044828760 |
| 10 | GGPN | C6' | 29.3108522721 | 16.2623405243 | 52.6916231984 |
| 10 | GGPN | H6' | 28.9144408719 | 17.0489311765 | 53.3040767553 |
| 10 | GGPN | C6'' | 29.8586613218 | 15.2360694108 | 53.7247521744 |
| 10 | GGPN | H6'' | 29.4211877324 | 15.6617613784 | 54.6356220119 |
| 10 | GGPN | H6''' | 30.9617322183 | 15.1931281675 | 53.8539575459 |
| 10 | GGPN | O7' | 29.2301588222 | 13.8258134400 | 53.6434773071 |
| 10 | GGPN | C8' | 28.9858857703 | 13.3384007520 | 54.9715408566 |
| 10 | GGPN | H8' | 28.6052972771 | 14.1504580386 | 55.6308449568 |
| 10 | GGPN | H8'' | 29.9198419382 | 12.9293141769 | 55.4107193301 |
| 10 | GGPN | C9' | 27.8764800630 | 12.2909271164 | 54.9606411439 |
| 10 | GGPN | H9' | 28.1513602989 | 11.5494203288 | 54.1760589809 |
| 10 | GGPN | H9'' | 26.9453187923 | 12.8283554093 | 54.6725674982 |
| 10 | GGPN | O10' | 27.5882838379 | 11.5991217967 | 56.1597319347 |
| 10 | GGPN | C11' | 26.5301730380 | 10.7000151694 | 55.8609247415 |
| 10 | GGPN | H11' | 26.8473405037 | 10.0999207288 | 54.9807760436 |
| 10 | GGPN | H11'' | 25.6135994009 | 11.2822528832 | 55.6033175710 |
| 10 | GGPN | C12' | 26.1594655152 | 9.6511079973 | 56.9040610149 |
| 10 | GGPN | H12' | 25.8103536709 | 10.1333561456 | 57.8430181689 |
| 10 | GGPN | H12'' | 27.0461847821 | 9.0097682557 | 57.1097409085 |
| 10 | GGPN | O13' | 25.1202997321 | 8.8845498174 | 56.2966266218 |
| 10 | GGPN | H13' | 24.8483874692 | 8.2065699822 | 56.9234394356 |
| 10 | GGPN | C3' | 29.4976626598 | 17.9812553127 | 49.7265260488 |
| 10 | GGPN | O3' | 28.9993585993 | 18.9168823042 | 49.1084042276 |
| 10 | GGPN | C4' | 29.7350305684 | 16.6309833181 | 49.0914403611 |
| 10 | GGPN | H4' | 29.1336127470 | 15.8705874171 | 49.6157971986 |
| 10 | GGPN | H4'' | 30.8104160874 | 16.3694931092 | 49.1804419642 |
| 10 | GGPN | N9 | 29.3348027472 | 16.6719641676 | 47.6973704597 |
| 10 | GGPN | C4 | 30.1916038994 | 16.8093127739 | 46.6488627007 |
| 10 | GGPN | N2 | 33.4045774522 | 17.1494931978 | 45.4135688243 |
| 10 | GGPN | H21 | 33.9366682952 | 17.2608322702 | 46.2406977192 |
| 10 | GGPN | H22 | 33.8257239015 | 17.2442948717 | 44.5070686889 |
| 10 | GGPN | N3 | 31.5306777423 | 16.9475016973 | 46.7368220530 |
| 10 | GGPN | C2 | 32.1011940237 | 16.9629979022 | 45.5370282743 |
| 10 | GGPN | N1 | 31.3927198839 | 16.8837580944 | 44.3681680033 |
| 10 | GGPN | H1 | 31.9087845796 | 16.8460473613 | 43.5036053946 |
| 10 | GGPN | C6 | 30.0142183397 | 16.7618863690 | 44.2535048777 |

| | | | | | |
|---|---|---|---|---|---|
| 10 | GGPN | O6 | 29.4592912408 | 16.7066894646 | 43.1532840389 |
| 10 | GGPN | C5 | 29.3959598962 | 16.7631874944 | 45.5239060649 |
| 10 | GGPN | N7 | 28.0563108505 | 16.6651875654 | 45.8657523343 |
| 10 | GGPN | C8 | 28.0628235113 | 16.6194082186 | 47.1698611251 |
| 10 | GGPN | H8 | 27.1733512361 | 16.5652019543 | 47.7991589449 |
| 11 | GCPN | C | 32.1532110807 | 25.0433676352 | 49.2497628007 |
| 11 | GCPN | O1' | 31.8536776547 | 26.1683294278 | 49.6300769812 |
| 11 | GCPN | C2' | 31.5230377154 | 23.8146981823 | 49.7960532772 |
| 11 | GCPN | H2' | 30.4556452792 | 23.8744481044 | 49.6517273830 |
| 11 | GCPN | H2'' | 31.7949518288 | 23.8005230635 | 50.8402503153 |
| 11 | GCPN | N2' | 32.0228399889 | 22.5881731177 | 49.1550798962 |
| 11 | GCPN | C5' | 33.0226731628 | 21.8335099592 | 49.8958854057 |
| 11 | GCPN | H5' | 33.7701055681 | 22.5620592795 | 50.1772403653 |
| 11 | GCPN | H5'' | 33.4384578103 | 21.0648797590 | 49.2673221234 |
| 11 | GCPN | N | 31.3037016668 | 20.4026454784 | 50.9087682797 |
| 11 | GCPN | H1' | 31.3781401499 | 19.5978303446 | 50.3316891520 |
| 11 | GCPN | C6' | 32.4850816177 | 21.1744023606 | 51.1828788520 |
| 11 | GCPN | H6' | 32.2274010017 | 21.9730042485 | 51.8660534574 |
| 11 | GCPN | C6'' | 33.5418521522 | 20.3334372511 | 51.9488668260 |
| 11 | GCPN | H6'' | 34.0542672124 | 20.9492412365 | 52.7185741992 |
| 11 | GCPN | H6''' | 34.3119655452 | 19.9786137102 | 51.2358754060 |
| 11 | GCPN | O7' | 32.8481988624 | 19.0877748998 | 52.5677349840 |
| 11 | GCPN | C8' | 33.1776607191 | 18.8537425116 | 53.9339683110 |
| 11 | GCPN | H8' | 33.7970736478 | 19.6724191389 | 54.3517698343 |
| 11 | GCPN | H8'' | 33.7958577491 | 17.9298928758 | 53.9780058097 |
| 11 | GCPN | C9' | 31.9279746767 | 18.6527347526 | 54.8269428643 |
| 11 | GCPN | H9' | 31.0835309585 | 18.2696035359 | 54.2368933799 |
| 11 | GCPN | H9'' | 31.5470990691 | 19.6215064802 | 55.2156884182 |
| 11 | GCPN | O10' | 32.1741312583 | 17.7063210246 | 55.8643193715 |
| 11 | GCPN | C11' | 30.9549290596 | 17.1890467742 | 56.3985314435 |
| 11 | GCPN | H11' | 30.2029897983 | 17.1084157795 | 55.5968124291 |
| 11 | GCPN | H11'' | 30.5079627176 | 17.8873661419 | 57.1345462477 |
| 11 | GCPN | C12' | 31.1521576820 | 15.8114818043 | 57.0498163634 |
| 11 | GCPN | H12' | 31.7820467805 | 15.9051304515 | 57.9605487421 |
| 11 | GCPN | H12'' | 31.6693859117 | 15.1291449968 | 56.3386939061 |
| 11 | GCPN | O13' | 29.8733743193 | 15.2895135573 | 57.3882671685 |
| 11 | GCPN | H13' | 30.0037579999 | 14.4776495743 | 57.8911185167 |
| 11 | GCPN | C3' | 31.3744712733 | 22.1283571645 | 48.0418615461 |
| 11 | GCPN | O3' | 30.6000643262 | 22.8633078749 | 47.4265495254 |
| 11 | GCPN | C4' | 31.5803945347 | 20.6854578805 | 47.5165949809 |
| 11 | GCPN | H4' | 31.1505719359 | 19.9978814598 | 48.2692917976 |
| 11 | GCPN | H4'' | 32.6633659468 | 20.4579685096 | 47.4486840701 |
| 11 | GCPN | N1 | 30.8926234954 | 20.4456773087 | 46.2367406637 |
| 11 | GCPN | C6 | 29.5338429509 | 20.2601478639 | 46.1833255669 |
| 11 | GCPN | H6 | 29.0159472942 | 20.2373323382 | 47.1476538438 |
| 11 | GCPN | C5 | 28.9009993213 | 20.0993805340 | 45.0118169061 |
| 11 | GCPN | H5 | 27.8246725563 | 19.9562240837 | 44.9495935656 |
| 11 | GCPN | C2 | 31.6290720421 | 20.5499819248 | 45.0518569059 |
| 11 | GCPN | O2 | 32.8357076955 | 20.8474411757 | 45.1076702940 |
| 11 | GCPN | N3 | 31.0145674090 | 20.3375719849 | 43.8681609122 |
| 11 | GCPN | C4 | 29.6846460581 | 20.1214881581 | 43.8193754836 |
| 11 | GCPN | N4 | 29.0961774986 | 19.9243536696 | 42.6344334906 |
| 11 | GCPN | H41 | 29.6607126268 | 19.8272751248 | 41.8098770016 |
| 11 | GCPN | H42 | 28.1552808803 | 19.6066502223 | 42.5829165102 |
| 12 | GAPN | C | 32.9617208064 | 29.0355273317 | 45.3821483825 |
| 12 | GAPN | O1' | 33.0479543306 | 30.2134808602 | 45.7064392154 |
| 12 | GAPN | C2' | 32.5648576281 | 27.9734687977 | 46.3356527661 |
| 12 | GAPN | H2' | 31.5084977217 | 28.0620816944 | 46.5401801946 |
| 12 | GAPN | H2'' | 33.1631490502 | 28.2018027710 | 47.1972050363 |
| 12 | GAPN | N2' | 32.8881622790 | 26.5999912809 | 45.8931641353 |
| 12 | GAPN | C5' | 34.0925274237 | 25.9509586996 | 46.4303215363 |
| 12 | GAPN | H5' | 34.9240577986 | 26.5989845130 | 46.2210533094 |
| 12 | GAPN | H5'' | 34.2468875742 | 24.9760417023 | 45.9952728205 |
| 12 | GAPN | N | 33.0923760061 | 24.8168179699 | 48.3311142653 |
| 12 | GAPN | H1' | 33.1516060988 | 23.8965426310 | 47.9663037606 |
| 12 | GAPN | C6' | 34.0707776030 | 25.7794554238 | 47.9405065052 |
| 12 | GAPN | H6' | 33.7801218761 | 26.7391615354 | 48.3366765531 |
| 12 | GAPN | C6'' | 35.4582779261 | 25.5155048664 | 48.5564479507 |
| 12 | GAPN | H6'' | 36.2786018891 | 25.6582114272 | 47.8226415530 |
| 12 | GAPN | H6''' | 35.4925064327 | 24.4726519324 | 48.9428485068 |
| 12 | GAPN | O7' | 35.6346631700 | 26.5263824941 | 49.6885260009 |
| 12 | GAPN | C8' | 36.0828252661 | 25.9489107233 | 50.8982712518 |
| 12 | GAPN | H8' | 37.1647601251 | 25.7063042145 | 50.8215214266 |

| | | | | | |
|---|---|---|---|---|---|
| 12 | GAPN | H8'' | 35.5062099918 | 25.0321047133 | 51.1528629781 |
| 12 | GAPN | C9' | 35.8481645677 | 27.0129564478 | 51.9670636164 |
| 12 | GAPN | H9' | 34.7526938454 | 27.1361512555 | 52.1260014657 |
| 12 | GAPN | H9'' | 36.2575440728 | 27.9808867339 | 51.5981354356 |
| 12 | GAPN | O10' | 36.4797604165 | 26.6714646563 | 53.1853373044 |
| 12 | GAPN | C11' | 36.3218869513 | 27.7203333712 | 54.1299700022 |
| 12 | GAPN | H11' | 35.2412309547 | 27.8794760084 | 54.3439985756 |
| 12 | GAPN | H11'' | 36.7409895787 | 28.6686700503 | 53.7230358219 |
| 12 | GAPN | C12' | 37.0573364621 | 27.3519657949 | 55.4183164250 |
| 12 | GAPN | H12' | 38.1364961212 | 27.2003629632 | 55.1878697691 |
| 12 | GAPN | H12'' | 36.6363108083 | 26.4007935481 | 55.8156906577 |
| 12 | GAPN | O13' | 36.8940627881 | 28.4013943604 | 56.3631488809 |
| 12 | GAPN | H13' | 37.3646237994 | 28.1464761171 | 57.1624205221 |
| 12 | GAPN | C3' | 31.9644621719 | 25.9574613118 | 45.1211431516 |
| 12 | GAPN | O3' | 31.0391588754 | 26.5685396285 | 44.5820584036 |
| 12 | GAPN | C4' | 32.0905013686 | 24.4512644305 | 44.8834713712 |
| 12 | GAPN | H4' | 32.0156778264 | 23.9204890042 | 45.8472456182 |
| 12 | GAPN | H4'' | 33.0826355802 | 24.2416551581 | 44.4341732236 |
| 12 | GAPN | N9 | 31.0678787416 | 24.0031163423 | 43.9463048906 |
| 12 | GAPN | C5 | 30.0486980083 | 23.4855038536 | 42.0841218483 |
| 12 | GAPN | N7 | 29.1298969208 | 23.2632258942 | 43.1002852146 |
| 12 | GAPN | C8 | 29.7787994656 | 23.5890513231 | 44.1906738051 |
| 12 | GAPN | H8 | 29.3752666785 | 23.5359241667 | 45.2050469668 |
| 12 | GAPN | N1 | 31.0962163380 | 23.6273341494 | 39.9817681037 |
| 12 | GAPN | C2 | 32.1595677504 | 24.1303970532 | 40.6086211034 |
| 12 | GAPN | H2 | 33.0095989722 | 24.3828839208 | 39.9652582877 |
| 12 | GAPN | N3 | 32.3395020668 | 24.3235764841 | 41.9155995520 |
| 12 | GAPN | C4 | 31.2356263463 | 23.9655046423 | 42.5943967499 |
| 12 | GAPN | C6 | 29.9945054775 | 23.2885084136 | 40.6941629471 |
| 12 | GAPN | N6 | 28.9213758778 | 22.7748345248 | 40.0680900246 |
| 12 | GAPN | H61 | 28.0804526272 | 22.5752984322 | 40.5619926125 |
| 12 | GAPN | H62 | 28.9695667969 | 22.5359263930 | 39.0912477012 |
| 13 | GTPN | C | 33.1442186590 | 32.3530949123 | 41.0302097002 |
| 13 | GTPN | O1' | 32.8217835477 | 33.5130287289 | 41.2496082257 |
| 13 | GTPN | C2' | 32.9778555129 | 31.3114090608 | 42.0595740945 |
| 13 | GTPN | H2' | 32.0321436567 | 31.4793322979 | 42.5562682276 |
| 13 | GTPN | H2'' | 33.8031414058 | 31.5387204656 | 42.7019302105 |
| 13 | GTPN | N2' | 33.0894235366 | 29.9052819368 | 41.6359388954 |
| 13 | GTPN | C5' | 34.2977037161 | 29.1785387705 | 42.0163382193 |
| 13 | GTPN | H5' | 35.1105757224 | 29.7320922205 | 41.5948835824 |
| 13 | GTPN | H5'' | 34.3089958051 | 28.1685638109 | 41.6463834636 |
| 13 | GTPN | N | 33.2824073545 | 28.6130623688 | 44.1663195832 |
| 13 | GTPN | H1' | 32.9201162737 | 27.7362870456 | 43.8706702786 |
| 13 | GTPN | C6' | 34.4472409607 | 29.1433596587 | 43.5368223637 |
| 13 | GTPN | H6' | 34.5634107028 | 30.1788146208 | 43.8248467127 |
| 13 | GTPN | C6'' | 35.6933340992 | 28.4762350519 | 44.1237940923 |
| 13 | GTPN | H6'' | 36.5022620091 | 28.3633908537 | 43.3896140104 |
| 13 | GTPN | H6''' | 35.4254603202 | 27.4605402865 | 44.4699882830 |
| 13 | GTPN | O7' | 36.0908051408 | 29.3928007070 | 45.2999985027 |
| 13 | GTPN | C8' | 37.0825951798 | 28.8952262250 | 46.1685596136 |
| 13 | GTPN | H8' | 37.8258700161 | 28.2988194254 | 45.6084795472 |
| 13 | GTPN | H8'' | 36.6288929577 | 28.2736847356 | 46.9724878457 |
| 13 | GTPN | C9' | 37.7269361066 | 30.1352408310 | 46.7916520531 |
| 13 | GTPN | H9' | 36.9540733492 | 30.6986200739 | 47.3653173838 |
| 13 | GTPN | H9'' | 38.1087293601 | 30.8040372725 | 45.9876337550 |
| 13 | GTPN | O10' | 38.7883237260 | 29.7518628676 | 47.6445206411 |
| 13 | GTPN | C11' | 39.2736828672 | 30.8622719521 | 48.3863697795 |
| 13 | GTPN | H11' | 38.4331473144 | 31.3711574241 | 48.9115756778 |
| 13 | GTPN | H11'' | 39.7622872927 | 31.5975828603 | 47.7089658443 |
| 13 | GTPN | C12' | 40.2784553866 | 30.3507632235 | 49.4194311413 |
| 13 | GTPN | H12' | 41.1370889573 | 29.8773387454 | 48.8915461379 |
| 13 | GTPN | H12'' | 39.7793622219 | 29.5829255708 | 50.0543793328 |
| 13 | GTPN | O13' | 40.7225578301 | 31.4388222789 | 50.2181428746 |
| 13 | GTPN | H13' | 41.3008121937 | 31.0800275431 | 50.8983232174 |
| 13 | GTPN | C3' | 31.9375191256 | 29.2785622939 | 41.2751097090 |
| 13 | GTPN | O3' | 30.8908399070 | 29.9029272144 | 41.1241296010 |
| 13 | GTPN | C4' | 31.9837368728 | 27.7580096009 | 41.0664647444 |
| 13 | GTPN | H4' | 32.1914846601 | 27.2830887828 | 42.0482634595 |
| 13 | GTPN | H4'' | 32.8290337720 | 27.5251326819 | 40.3886886178 |
| 13 | GTPN | N1 | 30.7372629333 | 27.1892039531 | 40.5331497095 |
| 13 | GTPN | C6 | 29.6951072431 | 26.9013946444 | 41.3576009842 |
| 13 | GTPN | H6 | 29.8620351354 | 27.1308680366 | 42.4134291273 |
| 13 | GTPN | C2 | 30.6654489332 | 27.1045018247 | 39.1391187853 |

| | | | | | |
|---|---|---|---|---|---|
| 13 | GTPN | O2 | 31.5014633178 | 27.4672684549 | 38.4058764519 |
| 13 | GTPN | N3 | 29.4615446130 | 26.5330163790 | 38.7418009126 |
| 13 | GTPN | H3 | 29.4034148159 | 26.4199562439 | 37.7336497910 |
| 13 | GTPN | C4 | 28.3939450830 | 26.1537605891 | 39.5021194483 |
| 13 | GTPN | O4 | 27.4100091939 | 25.6248825023 | 38.9666707308 |
| 13 | GTPN | C5 | 28.5325513867 | 26.3869771160 | 40.9180422783 |
| 13 | GTPN | C5M | 27.4108561120 | 26.0496632347 | 41.8322069677 |
| 13 | GTPN | H51 | 27.6713403471 | 26.2585768874 | 42.8943981587 |
| 13 | GTPN | H52 | 27.1579486420 | 24.9679693825 | 41.7515483595 |
| 13 | GTPN | H53 | 26.5036310594 | 26.6410492932 | 41.5737040125 |
| 14 | GGPN | C | 31.0196961128 | 35.6344883905 | 37.6027748668 |
| 14 | GGPN | O1' | 30.8258365217 | 36.8339731554 | 37.7598604960 |
| 14 | GGPN | C2' | 31.6665521649 | 34.7666808928 | 38.6435531054 |
| 14 | GGPN | H2' | 30.9954052305 | 34.6671714492 | 39.4880806918 |
| 14 | GGPN | H2'' | 32.5820011433 | 35.2614231960 | 38.9289755369 |
| 14 | GGPN | N2' | 31.9875659130 | 33.4224197275 | 38.1037616414 |
| 14 | GGPN | C5' | 33.3744284017 | 33.1047253925 | 37.7483486305 |
| 14 | GGPN | H5' | 33.7407155487 | 33.9389601543 | 37.1727929599 |
| 14 | GGPN | H5'' | 33.4127151004 | 32.1972815220 | 37.1602758579 |
| 14 | GGPN | N | 33.7281581305 | 31.9907891981 | 39.8870837362 |
| 14 | GGPN | H1' | 33.9028130989 | 31.0252591327 | 39.7152501778 |
| 14 | GGPN | C6' | 34.2966711681 | 32.9419188818 | 38.9744603038 |
| 14 | GGPN | H6' | 34.3565692702 | 33.9155986619 | 39.4598656292 |
| 14 | GGPN | C6'' | 35.7663368599 | 32.5689051780 | 38.6125207187 |
| 14 | GGPN | H6'' | 36.4326157351 | 33.4429942646 | 38.7640382305 |
| 14 | GGPN | H6''' | 35.8592683117 | 32.2740562428 | 37.5478565662 |
| 14 | GGPN | O7' | 36.2048055837 | 31.3655033470 | 39.4932897978 |
| 14 | GGPN | C8' | 37.4590632542 | 31.5548668069 | 40.1447037137 |
| 14 | GGPN | H8' | 37.6173546153 | 32.6149595320 | 40.4375234132 |
| 14 | GGPN | H8'' | 38.2872246392 | 31.2301175930 | 39.4765584016 |
| 14 | GGPN | C9' | 37.4777966199 | 30.6869774181 | 41.4078520138 |
| 14 | GGPN | H9' | 37.1051439165 | 29.6812412650 | 41.1113992454 |
| 14 | GGPN | H9'' | 36.7755912081 | 31.1189467756 | 42.1590914905 |
| 14 | GGPN | O10' | 38.7699058266 | 30.5308845919 | 41.9760113795 |
| 14 | GGPN | C11' | 38.7408496399 | 29.4736505722 | 42.9236054962 |
| 14 | GGPN | H11' | 38.3244449770 | 28.5662690432 | 42.4253467758 |
| 14 | GGPN | H11'' | 38.0841053932 | 29.7743775622 | 43.7654290490 |
| 14 | GGPN | C12' | 40.1006847444 | 29.0606196094 | 43.4935580166 |
| 14 | GGPN | H12' | 40.5151378843 | 29.8676710009 | 44.1360573963 |
| 14 | GGPN | H12'' | 40.7953127922 | 28.8796267711 | 42.6446066154 |
| 14 | GGPN | O13' | 39.9413663724 | 27.8564908898 | 44.2406292985 |
| 14 | GGPN | H13' | 39.7197560084 | 28.1066094344 | 45.1444257775 |
| 14 | GGPN | C3' | 30.9910412889 | 32.4913747722 | 38.1307827164 |
| 14 | GGPN | O3' | 29.8287000665 | 32.8444856134 | 38.3557547366 |
| 14 | GGPN | C4' | 31.2521282177 | 31.0073281046 | 37.8490323436 |
| 14 | GGPN | H4' | 31.8172999707 | 30.5756605320 | 38.6947584619 |
| 14 | GGPN | H4'' | 31.8413120932 | 30.8942433743 | 36.9180530529 |
| 14 | GGPN | N9 | 29.9747423122 | 30.3140633460 | 37.7078846274 |
| 14 | GGPN | C4 | 29.4159998806 | 29.8957197088 | 36.5304951738 |
| 14 | GGPN | N2 | 29.4328563680 | 29.7484214280 | 33.0721424376 |
| 14 | GGPN | H21 | 30.2815678354 | 30.2064784982 | 32.8421691011 |
| 14 | GGPN | H22 | 28.8052388721 | 29.4525396127 | 32.3447363383 |
| 14 | GGPN | N3 | 29.9391034425 | 30.0638802360 | 35.2983553556 |
| 14 | GGPN | C2 | 29.1288293039 | 29.6022276841 | 34.3502907403 |
| 14 | GGPN | N1 | 27.9292699276 | 28.9928414242 | 34.6152967019 |
| 14 | GGPN | H1 | 27.3895023692 | 28.6404913076 | 33.8403145945 |
| 14 | GGPN | C6 | 27.3811188837 | 28.7850766777 | 35.8733202841 |
| 14 | GGPN | O6 | 26.2868751471 | 28.2287686323 | 36.0048841299 |
| 14 | GGPN | C5 | 28.2090122951 | 29.3224117859 | 36.8884719132 |
| 14 | GGPN | N7 | 27.9940927340 | 29.4241771232 | 38.2532359242 |
| 14 | GGPN | C8 | 29.0657126586 | 30.0254589953 | 38.7014453994 |
| 14 | GGPN | H8 | 29.2378461322 | 30.3228011676 | 39.7370144431 |
| 15 | GCPN | C | 27.0367775367 | 36.8547381836 | 32.7137895195 |
| 15 | GCPN | O1' | 26.6769750157 | 37.9239248859 | 32.2404714528 |
| 15 | GCPN | C2' | 27.8985675805 | 36.8543408244 | 33.9528506802 |
| 15 | GCPN | H2' | 27.3148882489 | 37.2820640182 | 34.7523883750 |
| 15 | GCPN | H2'' | 28.7018171710 | 37.5166760023 | 33.6724506920 |
| 15 | GCPN | N2' | 28.5098363705 | 35.6038470677 | 34.4348664270 |
| 15 | GCPN | C5' | 29.8755153799 | 35.3305967449 | 34.0394335094 |
| 15 | GCPN | H5' | 30.1614135760 | 36.1326736499 | 33.3808718665 |
| 15 | GCPN | H5'' | 29.8547608198 | 34.3982875921 | 33.5043306241 |
| 15 | GCPN | N | 30.6898224293 | 34.9575853183 | 36.4991722896 |
| 15 | GCPN | H1' | 30.5025914367 | 34.0125060971 | 36.7406390699 |

| | | | | | |
|---|---|---|---|---|---|
| 15 | GCPN | C6' | 30.9994970411 | 35.2140041884 | 35.1121133509 |
| 15 | GCPN | H6' | 31.2592189511 | 34.1795602456 | 34.9601519432 |
| 15 | GCPN | C6'' | 32.3372600231 | 35.8988527899 | 34.6953532972 |
| 15 | GCPN | H6'' | 32.2164814544 | 36.7362419115 | 33.9772070121 |
| 15 | GCPN | H6''' | 32.9947358948 | 35.1337512902 | 34.2397318051 |
| 15 | GCPN | O7' | 33.0383254933 | 36.4357394616 | 35.9000244942 |
| 15 | GCPN | C8' | 34.4361318409 | 36.3991001243 | 35.7783436323 |
| 15 | GCPN | H8' | 34.7916950421 | 37.1147976581 | 35.0085307140 |
| 15 | GCPN | H8'' | 34.7965749675 | 35.3751154001 | 35.5335576672 |
| 15 | GCPN | C9' | 34.8012153206 | 36.7857868474 | 37.1920993550 |
| 15 | GCPN | H9' | 34.2150691601 | 36.1506484112 | 37.8943169107 |
| 15 | GCPN | H9'' | 34.5014728893 | 37.8423292365 | 37.3784428137 |
| 15 | GCPN | O10' | 36.1664744457 | 36.5979759644 | 37.4628829052 |
| 15 | GCPN | C11' | 36.3482664476 | 36.5942887419 | 38.8640008217 |
| 15 | GCPN | H11' | 35.9646499906 | 35.6407237184 | 39.2879725274 |
| 15 | GCPN | H11'' | 35.7719635120 | 37.4309657275 | 39.3214251390 |
| 15 | GCPN | C12' | 37.8317721272 | 36.7418988190 | 39.1540982279 |
| 15 | GCPN | H12' | 38.2147010495 | 37.6590939404 | 38.6501106813 |
| 15 | GCPN | H12'' | 38.3805900903 | 35.8628267253 | 38.7472252987 |
| 15 | GCPN | O13' | 38.0079432150 | 36.8250543054 | 40.5594605842 |
| 15 | GCPN | H13' | 37.5182595596 | 37.5964026097 | 40.8635133876 |
| 15 | GCPN | C3' | 27.7386729322 | 34.6952017532 | 35.0891324530 |
| 15 | GCPN | O3' | 26.6288975506 | 35.0031238780 | 35.5241945679 |
| 15 | GCPN | C4' | 28.2005990918 | 33.2284871176 | 35.2546939678 |
| 15 | GCPN | H4' | 28.8702024512 | 33.1720375351 | 36.1284026435 |
| 15 | GCPN | H4'' | 28.7667573873 | 32.8903618591 | 34.3649956330 |
| 15 | GCPN | N1 | 27.0318310956 | 32.3679720497 | 35.4667220419 |
| 15 | GCPN | C6 | 26.5631813949 | 32.0988327144 | 36.7257900706 |
| 15 | GCPN | H6 | 27.1875824736 | 32.4489602972 | 37.5540322399 |
| 15 | GCPN | C5 | 25.4447971822 | 31.3827077344 | 36.9050710425 |
| 15 | GCPN | H5 | 25.0628488537 | 31.1307786221 | 37.8909476821 |
| 15 | GCPN | C2 | 26.3004735326 | 31.9782027714 | 34.3403606265 |
| 15 | GCPN | O2 | 26.6807192733 | 32.3289514946 | 33.2077301889 |
| 15 | GCPN | N3 | 25.1941568972 | 31.2290358294 | 34.5119230840 |
| 15 | GCPN | C4 | 24.7486988925 | 30.9363794061 | 35.7457088826 |
| 15 | GCPN | N4 | 23.6336461583 | 30.2198631079 | 35.8825531989 |
| 15 | GCPN | H41 | 23.2637319418 | 29.8083975522 | 35.0385092859 |
| 15 | GCPN | H42 | 23.4757867544 | 29.7030995183 | 36.7156620273 |
| 16 | GCPN | C | 21.7991762155 | 36.7105152505 | 29.8655231323 |
| 16 | GCPN | C2' | 23.1131591921 | 37.0913033108 | 30.5636606961 |
| 16 | GCPN | H2' | 22.9216779292 | 37.9630333223 | 31.1669804533 |
| 16 | GCPN | H2'' | 23.7955839325 | 37.3192497475 | 29.7588440078 |
| 16 | GCPN | N2' | 23.7401497070 | 36.0641377825 | 31.4091488356 |
| 16 | GCPN | C5' | 24.6642508960 | 35.1692329028 | 30.7337919609 |
| 16 | GCPN | H5' | 24.3373957084 | 35.1634625611 | 29.7084421811 |
| 16 | GCPN | H5'' | 24.5643043497 | 34.1775121018 | 31.1392087572 |
| 16 | GCPN | N | 26.7009938938 | 35.6950086896 | 32.1191918129 |
| 16 | GCPN | H1' | 26.9081413238 | 34.8315336698 | 32.5717506661 |
| 16 | GCPN | C6' | 26.1589913348 | 35.6208421431 | 30.7688408417 |
| 16 | GCPN | H6' | 26.1708890869 | 36.6147678396 | 30.3227248042 |
| 16 | GCPN | C6'' | 27.0577246102 | 34.7443693384 | 29.8347949054 |
| 16 | GCPN | H6'' | 27.2296725541 | 35.2381538656 | 28.8572248577 |
| 16 | GCPN | H6''' | 26.5652797677 | 33.7758056939 | 29.6280356647 |
| 16 | GCPN | O7' | 28.3905248092 | 34.4354294362 | 30.5491127311 |
| 16 | GCPN | C8' | 29.5539874304 | 34.4865191185 | 29.7260892325 |
| 16 | GCPN | H8' | 29.5050164054 | 35.3115944334 | 28.9842548886 |
| 16 | GCPN | H8'' | 29.7293736011 | 33.5177605236 | 29.2092397930 |
| 16 | GCPN | C9' | 30.6492504115 | 34.7600938911 | 30.7599204063 |
| 16 | GCPN | H9' | 30.4570916565 | 34.0461490908 | 31.5913391542 |
| 16 | GCPN | H9'' | 30.4809639104 | 35.7944065394 | 31.1390571771 |
| 16 | GCPN | O10' | 32.0140046879 | 34.6186802868 | 30.3990628421 |
| 16 | GCPN | C11' | 32.7787926491 | 34.8238926995 | 31.5830980445 |
| 16 | GCPN | H11' | 32.4399131005 | 34.0915883124 | 32.3520772862 |
| 16 | GCPN | H11'' | 32.5561466996 | 35.8460909462 | 31.9628077839 |
| 16 | GCPN | C12' | 34.3057249213 | 34.6905747320 | 31.4587191518 |
| 16 | GCPN | H12' | 34.6843614633 | 35.4144091392 | 30.7046838773 |
| 16 | GCPN | H12'' | 34.5681972722 | 33.6587770065 | 31.1377168313 |
| 16 | GCPN | O13' | 34.8826759998 | 34.9654228098 | 32.7347217009 |
| 16 | GCPN | H13' | 35.8409169979 | 35.0093765474 | 32.6356464945 |
| 16 | GCPN | C3' | 23.4593710522 | 36.0087373922 | 32.7473917162 |
| 16 | GCPN | O3' | 22.7632658122 | 36.8465623529 | 33.3303972997 |
| 16 | GCPN | C4' | 24.0311498636 | 34.8897922863 | 33.6699758028 |
| 16 | GCPN | H4' | 24.5720256594 | 35.4558519608 | 34.4520856303 |

| | | | | | |
|---|---|---|---|---|---|
| 16 | GCPN | H4'' | 24.7814522462 | 34.2639396709 | 33.1649595920 |
| 16 | GCPN | N1 | 23.0369089558 | 34.0026595655 | 34.2885062911 |
| 16 | GCPN | C6 | 22.8113113692 | 33.9825456601 | 35.6416155244 |
| 16 | GCPN | H6 | 23.3976767044 | 34.6937954368 | 36.2272543581 |
| 16 | GCPN | C5 | 21.9190274752 | 33.1347262373 | 36.1838521130 |
| 16 | GCPN | H5 | 21.7230387914 | 33.0806060944 | 37.2510796904 |
| 16 | GCPN | C2 | 22.4146118993 | 33.0623818493 | 33.4650482437 |
| 16 | GCPN | O2 | 22.7376168449 | 32.9777522200 | 32.2730150544 |
| 16 | GCPN | N3 | 21.4884153414 | 32.2432790751 | 33.9933649074 |
| 16 | GCPN | C4 | 21.2136092040 | 32.2663001038 | 35.3001220250 |
| 16 | GCPN | N4 | 20.2677438759 | 31.4458054273 | 35.7587524312 |
| 16 | GCPN | H41 | 19.8375647824 | 30.8364164525 | 35.0745801778 |
| 16 | GCPN | H42 | 20.0963440621 | 31.2740321717 | 36.7186916486 |
| 16 | GCPN | OT1 | 21.2862046139 | 35.5745780449 | 30.0569537915 |
| 16 | GCPN | OT2 | 21.3062964832 | 37.5770968764 | 29.0925300272 |

## Input deck for model compound optimization.

```
%chk=gpna-set1.chk
%nprocshared=20
%mem=150GB
#p opt=(calcfc,noeigen) freq mp2/6-31+g(d) pop=(mk,dipole) density=all
nosym

Gamma modified diPNA optimization. AT15Dec2020

0 1
 N                  0.00000000    0.00000000    0.00000000
 C                  0.00000000    0.00000000    1.45160000
 C                 -1.40343939    0.24746423    2.00784399
 N                 -2.23593237   -0.79331283    1.43186468
 C                 -2.07245003   -2.14113734    1.97685238
 C                  0.39317057   -1.08022725   -0.73003711
 C                  0.34418409   -0.94563800   -2.24940121
 N                 -0.52340578    0.10220676   -2.76314461
 C                 -0.07902617    1.49381655   -2.74146641
 C                 -3.14005152   -0.67683076    0.45942432
 C                 -3.19354021    0.64976328   -0.28125056
 O                 -3.81960828   -1.63726830    0.07003141
 C                 -1.76111643   -0.26824723   -3.26248240
 C                 -2.57484855    0.82212528   -3.92756524
 O                 -2.16569832   -1.43410106   -3.19882058
 O                  0.77785341   -2.13713467   -0.21680858
 H                 -0.67324390    2.09732815   -2.04175147
 H                  0.96216437    1.51856506   -2.41251258
 H                 -0.13597376    1.97264612   -3.72379614
 H                 -2.08305470    1.17863792   -4.83948411
 H                 -2.73057991    1.68398585   -3.27132177
 H                 -3.54118840    0.39043427   -4.18945117
 H                 -0.27597028   -1.80845898   -2.52568534
 H                  0.94533660   -0.67105525   -3.11807388
 H                 -0.30163686    0.82874047   -0.50382388
 H                 -0.80287698   -0.69482205    1.73361564
 H                 -1.75953648    1.23602863    1.70979008
 H                 -1.34440111    0.24842065    3.10665868
 H                 -3.50223575    1.47626833    0.36747162
 H                 -3.92585416    0.52968911   -1.08057838
 H                 -2.22845319    0.90913982   -0.73008361
 H                 -1.99558574   -1.90775181    3.29254739
 H                 -1.49413911   -2.71783099    1.95377767
 H                 -3.24205851   -2.55381898    2.18842580
 C                  1.25265492   -0.78197982    1.88859338
 H                  2.04828755   -0.59591637    1.19776120
 H                  1.03194532   -1.82884287    1.90487313
 O                  1.64747888   -0.35768928    3.19587743
 C                  2.96621767   -0.83524805    3.47474233
 H                  3.65794295   -0.39426363    2.78775698
 H                  3.23794033   -0.56892918    4.47481286
 H                  2.98951790   -1.89988540    3.37031822

D 8 9 13 7 F
D 4 5 3 10 F
D 1 2 6 25 F
D 6 1 7 16 F
D 25 1 6 16 F
```

## Input deck for model compound dihedral scan.

```
%chk=gpna-set1-scan-rev.chk
%nprocshared=16
%mem=350GB
#p opt=(calcfc,modredundant,noeigen) mp2/6-31+g(d) nosymm

Gamma modified diPNA linkage scan. AT28Dec2020

0 1
N                    0.00949  -1.26915  -0.03637
C                   -0.06146  -1.13159   1.40786
C                   -0.89324   0.12077   1.77551
N                   -2.06242   0.20915   0.91392
C                   -3.03658  -0.87358   1.01139
C                    0.64146  -0.40451  -0.86638
C                    0.54916  -0.80605  -2.3336
N                   -0.81249  -0.5327   -2.80549
C                   -1.07516   0.77761  -3.40193
C                   -2.31177   1.22042   0.03032
C                   -1.44257   2.46024   0.08631
O                   -3.25322   1.13984  -0.787
C                   -1.75506  -1.5061   -2.66488
C                   -3.13456  -1.23138  -3.20652
O                   -1.48894  -2.57966  -2.07482
O                    1.24367   0.62071  -0.49729
H                   -2.09511   1.09432  -3.18478
H                   -0.39891   1.50357  -2.94407
H                   -0.90362   0.76464  -4.48572
H                   -3.10799  -0.84962  -4.23159
H                   -3.63598  -0.49289  -2.56999
H                   -3.68978  -2.16981  -3.1807
H                    0.76677  -1.86927  -2.47446
H                    1.2553   -0.19978  -2.9056
H                   -0.44189  -2.068    -0.48806
H                   -0.58648  -2.02371   1.76922
H                   -0.27535   1.00946   1.6456
H                   -1.19748   0.05891   2.82726
H                   -1.44201   2.89962   1.08961
H                   -1.87248   3.17817  -0.6139
H                   -0.40661   2.25067  -0.19586
H                   -3.03664  -1.2489    2.03973
H                   -2.80504  -1.69765   0.3249
H                   -4.02441  -0.48468   0.7593
C                    1.31536  -1.10517   2.0573
H                    1.90357  -0.262     1.67232
H                    1.8498   -2.04303   1.8374
O                    1.09781  -0.96791   3.45915
C                    2.32994  -0.91003   4.17339
H                    2.92884  -0.04906   3.84854
H                    2.07506  -0.8036    5.22835
H                    2.91091  -1.8304    4.02648


D 1 2 35 38 S 24 -15.000000
D 8 9 13 7 F
D 10 3 5 4 F
D 1 2 6 25 F
D 6 1 7 16 F
D 25 1 6 16 F
```

**NAMD configuration file for minimization and equilibration of miniPEG-modified 2KVJ.**

```
#############################################################
## JOB DESCRIPTION                                         ##
#############################################################

# minimization/equilibration of 2KVJ
# in a box of water


#############################################################
## ADJUSTABLE PARAMETERS                                   ##
#############################################################

structure          2kvj-wb.psf
coordinates        2kvj-wb.pdb

set temperature    310
set outputname     2kvj-eq
set inputname      2kvj-wb ;# only need to edit this in one place!
firsttimestep      0


#############################################################
## SIMULATION PARAMETERS                                   ##
#############################################################

# Input
paraTypeCharmm     on
parameters          par_all36_prot.prm
parameters          par_all36_carb.prm
parameters          par_all36_na.prm
parameters          par_all35_ethers.prm
parameters          par_all36_cgenff.prm
parameters          par_all36_lipid.prm
parameters          LH-toppar_all36_na_modifications-AT.str
Parameters          toppar_water_ions_namd.str
temperature         $temperature


# Force-Field Parameters
exclude             scaled1-4
1-4scaling          1.0
cutoff              12.0
switching           on
switchdist          10.0
pairlistdist        14.0


# Integrator Parameters
timestep            2.0  ;# 2fs/step
rigidBonds          all  ;# needed for 2fs steps
nonbondedFreq       1
fullElectFrequency  2
stepspercycle       10


# Constant Temperature Control
langevin            on    ;# do langevin dynamics
langevinDamping     1     ;# damping coefficient (gamma) of 1/ps
langevinTemp        $temperature
langevinHydrogen    off    ;# don't couple langevin bath to hydrogens


# Periodic Boundary Conditions
cellBasisVector1    61.17   0.0    0.0
cellBasisVector2     0.0   60.0   0.0
cellBasisVector3     0.0    0.0   76.0
cellOrigin          30.16  23.46  39.01

wrapAll             on
```

```
# PME (for full-system periodic electrostatics)
PME                 yes
PMEGridSpacing      1.0

#manual grid definition
#PMEGridSizeX        45
#PMEGridSizeY        45
#PMEGridSizeZ        48


# Constant Pressure Control (variable volume)
useGroupPressure      yes ;# needed for rigidBonds
useFlexibleCell       no
useConstantArea       no

langevinPiston        on
langevinPistonTarget  1.01325 ;#  in bar -> 1 atm
langevinPistonPeriod  100.0
langevinPistonDecay   50.0
langevinPistonTemp    $temperature


# Output
outputName          $outputname

restartfreq         5000     ;# 5000steps = every 10ps
dcdfreq             5000
xstFreq             5000
outputEnergies      5000
outputPressure      5000


#############################################################
## EXTRA PARAMETERS                                        ##
#############################################################


#############################################################
## EXECUTION SCRIPT                                        ##
#############################################################

# Minimization
minimize            1000
reinitvels          $temperature

run 50000000 ;# 100ns
```

**NAMD configuration file for a 200-nanosecond production run of miniPEG-modified 2KVJ.**

```
#############################################################
## JOB DESCRIPTION                                         ##
#############################################################

# 200 ns production run of 2KVJ
# in a box of water


#############################################################
## ADJUSTABLE PARAMETERS                                   ##
#############################################################

structure          2kvj-wb.psf
coordinates        2kvj-wb.pdb

set temperature    310
set outputname     2kvj-prod1
set inputname      2kvj-eq ;# only need to edit this in one place!
## continuing a run##
binCoordinates     $inputname.coor ;# coordinates from last run (binary)
binVelocities      $inputname.vel ;# velocities from last run (binary)
extendedSystem     $inputname.xsc ;# cell dimensions from last run

firsttimestep      0


#############################################################
## SIMULATION PARAMETERS                                   ##
#############################################################

# Input
paraTypeCharmm     on
parameters          par_all36_prot.prm
parameters          par_all36_carb.prm
parameters          par_all36_na.prm
parameters          par_all35_ethers.prm
parameters          par_all36_cgenff.prm
parameters          par_all36_lipid.prm
parameters          LH-toppar_all36_na_modifications-AT.str
Parameters          toppar_water_ions_namd.str
#temperature         $temperature


# Force-Field Parameters
exclude             scaled1-4
1-4scaling          1.0
cutoff              12.0
switching           on
switchdist          10.0
pairlistdist        14.0


# Integrator Parameters
timestep            2.0  ;# 2fs/step
rigidBonds          all  ;# needed for 2fs steps
nonbondedFreq       1
fullElectFrequency  2
stepspercycle       10


# Constant Temperature Control
langevin            on    ;# do langevin dynamics
langevinDamping     1     ;# damping coefficient (gamma) of 1/ps
langevinTemp        $temperature
langevinHydrogen    off    ;# don't couple langevin bath to hydrogens


# Periodic Boundary Conditions
#cellBasisVector1    57.68   0.0    0.0
```

```
#cellBasisVector2     0.0   65.75   0.0
#cellBasisVector3     0.0    0.0   60.46
#cellOrigin           0.09  -0.01   0.23

wrapAll             on


# PME (for full-system periodic electrostatics)
PME                 yes
PMEGridSpacing      1.0

#manual grid definition
#PMEGridSizeX        45
#PMEGridSizeY        45
#PMEGridSizeZ        48


# Constant Pressure Control (variable volume)
useGroupPressure      yes ;# needed for rigidBonds
useFlexibleCell       no
useConstantArea       no

langevinPiston        on
langevinPistonTarget  1.01325 ;#  in bar -> 1 atm
langevinPistonPeriod  100.0
langevinPistonDecay   50.0
langevinPistonTemp    $temperature


# Output
outputName          $outputname

restartfreq         5000     ;# 5000steps = every 10ps
dcdfreq             5000     ;# saving out = 100 ps
xstFreq             5000
outputEnergies      5000
outputPressure      5000


#############################################################
## EXTRA PARAMETERS                                        ##
#############################################################


#############################################################
## EXECUTION SCRIPT                                        ##
#############################################################

# Minimization
#minimize            1000
#reinitvels          $temperature

run 100000000 ;# 200ns 2000 frames
```

References

(1) Jasiński, M.; Feig, M.; Trylska, J. Improved Force Fields for Peptide Nucleic Acids with Optimized Backbone Torsion Parameters. *J. Chem. Theory Comput.* **2018**, *14* (7), 3603–3620.

(2) McGibbon, R. T.; Beauchamp, K. A.; Harrigan, M. P.; Klein, C.; Swails, J. M.; Hernández, C. X.; Schwantes, C. R.; Wang, L.-P. P.; Lane, T. J.; Pande, V. S. MDTraj: A Modern Open Library for the Analysis of Molecular Dynamics Trajectories. *Biophys. J.* **2015**, *109* (8), 1528–1532.

(3) Pedregosa, F.; Varoquaux, G.; Gramfort, A.; Michel, V.; Thirion, B.; Grisel, O.; Blondel, M.; Prettenhofer, P.; Weiss, R.; Dubourg, V.; et al. Scikit-Learn: Machine Learning in Python. *J. Mach. Learn. Res.* **2011**, *12* (85), 2825–2830.

(4) He, W.; Crawford, M. J.; Rapireddy, S.; Madrid, M.; Gil, R. R.; Ly, D. H.; Achim, C. The Structure of a γ-Modified Peptide Nucleic Acid Duplex. *Mol. Biosyst.* **2010**, *6* (9), 1619.

(5) Wishart, D. S.; Knox, C.; Guo, A. C.; Shrivastava, S.; Hassanali, M.; Stothard, P.; Chang, Z.; Woolsey, J. DrugBank: A Comprehensive Resource for in Silico Drug Discovery and Exploration. *Nucleic Acids Res.* **2006**, *34* (Database issue), D668-72.

(6) Bloomfield, V. A.; Crothers, D. M.; Jr, Tinoco, I. Nucleic Acid Structures from Diffraction Methods. In *Nucleic Acids: Structures, Properties, and Functions*; University Science Books: Sausalito, California, California, 2000; pp 79–110.
